# The use of RNA-based treatments in the field of cancer immunotherapy

**DOI:** 10.1186/s12943-023-01807-w

**Published:** 2023-07-07

**Authors:** Mohammad Chehelgerdi, Matin Chehelgerdi

**Affiliations:** 1 Novin Genome (NG) Lab, Research and Development Center for Biotechnology, Shahrekord, Iran; 2 Young Researchers and Elite Club, Shahrekord Branch, Islamic Azad University, Shahrekord, Iran

**Keywords:** mRNA vaccines, Cancer immunotherapy, Messenger RNA, Therapeutic use, Clinical trials, Technological advancements, Infectious diseases, Animal models

## Abstract

Over the past several decades, mRNA vaccines have evolved from a theoretical concept to a clinical reality. These vaccines offer several advantages over traditional vaccine techniques, including their high potency, rapid development, low-cost manufacturing, and safe administration. However, until recently, concerns over the instability and inefficient distribution of mRNA in vivo have limited their utility. Fortunately, recent technological advancements have mostly resolved these concerns, resulting in the development of numerous mRNA vaccination platforms for infectious diseases and various types of cancer. These platforms have shown promising outcomes in both animal models and humans. This study highlights the potential of mRNA vaccines as a promising alternative approach to conventional vaccine techniques and cancer treatment. This review article aims to provide a thorough and detailed examination of mRNA vaccines, including their mechanisms of action and potential applications in cancer immunotherapy. Additionally, the article will analyze the current state of mRNA vaccine technology and highlight future directions for the development and implementation of this promising vaccine platform as a mainstream therapeutic option. The review will also discuss potential challenges and limitations of mRNA vaccines, such as their stability and in vivo distribution, and suggest ways to overcome these issues. By providing a comprehensive overview and critical analysis of mRNA vaccines, this review aims to contribute to the advancement of this innovative approach to cancer treatment.

## Introduction

Cancer immunotherapies have gained significant attention and popularity in recent years, significant advancements have been made in cancer immunotherapies with the FDA approval of checkpoint blockade modulators (such as pembrolizumab in 2014 and nivolumab in 2015) and CAR-T cell immunotherapies (like tisagenlecleucel in 2017 and axicabtagene ciloleucel in 2018) [1]. These immunotherapies work by enhancing the immune system's ability to recognize and destroy cancer cells, offering a promising alternative to traditional cancer treatments. The approval of these treatments highlights the potential of cancer immunotherapy as a novel and effective approach to cancer treatment [2].

The primary goal of cancer immunotherapies is to enhance the host's anti-tumor immunity and modify the tumor's suppressive microenvironment. By doing so, these therapies aim to inhibit the growth of the patient's tumor and prolong their lifespan [2]. Through the stimulation of the immune system, cancer immunotherapies have shown potential to induce long-term remission and offer a durable treatment option for cancer patients. Additionally, cancer immunotherapy may also have fewer side effects than traditional cancer treatments, which can lead to an improved quality of life for patients [3].

Cancer vaccines have emerged as a promising alternative approach to cancer immunotherapy, with potential applications in cancer prevention and therapy. Unlike traditional vaccines that are used to prevent infectious diseases, cancer vaccines are designed to stimulate the immune system to recognize and attack cancer cells. Cancer vaccines may be used as a preventative measure in individuals at high risk for developing certain types of cancer, or as a therapeutic option to treat existing cancer. The development of cancer vaccines has the potential to revolutionize cancer treatment by offering a more targeted and personalized approach, with fewer side effects than conventional cancer treatments. The application of cancer vaccines in both cancer prevention and therapy highlights their potential as a promising tool in the fight against cancer [2]. Vaccinations against tumor-associated or tumor-specific antigens (TAAs or TSAs) have shown promise in targeting and destroying cancer cells that overexpress certain antigens, leading to a long-lasting therapeutic response. TAAs and TSAs are specific molecules expressed by cancer cells that are not found on normal cells, making them a unique and attractive target for cancer immunotherapy. By vaccinating individuals with cancer-specific antigens, the immune system can be trained to recognize and destroy cancer cells. This targeted approach has the potential to induce a long-lasting immune response, providing a durable and effective treatment option for cancer patients. The ability to specifically target cancer cells that overexpress certain antigens highlights the potential of vaccinations against TAAs or TSAs in cancer immunotherapy [4].

Immunologic memory, a property of the immune system, plays a crucial role in the effectiveness of cancer vaccines. This memory allows the immune system to recognize and attack cancer cells even after the initial exposure to a cancer vaccine. Unlike other types of immunotherapies, cancer vaccines offer a specific, non-toxic, and well-tolerated therapy option. By targeting cancer-specific antigens, cancer vaccines have the potential to induce a targeted immune response, reducing the risk of adverse effects and toxicities associated with traditional cancer treatments. The specific nature of cancer vaccines allows for a personalized approach to cancer treatment, targeting the specific antigens expressed by a patient's cancer cells. This personalized approach, along with the non-toxic and well-tolerated nature of cancer vaccines, highlights their potential as a valuable tool in cancer immunotherapy [3]. Despite significant research efforts, the clinical translation of cancer vaccines into effective medicines has been challenging for decades. One of the primary reasons for this challenge is the highly variable nature of tumor antigens, making it difficult to identify specific targets for cancer vaccines. Additionally, the immune response generated by cancer vaccines has often been insufficient to produce a therapeutic effect. These factors have hindered the development of effective cancer vaccines and limited their potential impact on cancer treatment. Despite these challenges, ongoing research and advancements in cancer immunotherapy hold promise for the future development of effective cancer vaccines. By identifying more specific targets for cancer vaccines and developing more potent immune responses, researchers may be able to overcome these hurdles and harness the full potential of cancer vaccines in cancer treatment [5].

This is the case despite the fact that there have been significant attempts to generate cancer vaccines. Despite the fact that the human papillomavirus (HPV) is responsible for 70% of cervical cancers and the hepatitis B virus may cause liver cancer, the Food and Drug Administration in the United States has only recently licenced two prophylactic vaccines. More encouragingly, the first therapeutic cancer vaccine, PROVENGE (sipuleucel-T), was licensed by the U.S. FDA [3] which was designed to treat hormone-resistant prostate cancer. In the treatment of a broad range of solid and metastatic cancers, clinical trials are now examining a wide variety of customised cancer vaccines in conjunction with checkpoint blockade modulators or cytokine therapies, with positive results [3, 5]. Cancer vaccines may be classified into the following four groups: those that are based on tumour cells or immune cells; those that are based on peptides; those that are based on viral vectors; and those that are based on nucleic acids [5]. There are several reasons why vaccines that are constructed using nucleic acids (DNA or RNA) have a great deal of potential. The first advantage of nucleic acid vaccines is that they can deliver multiple antigens all at once, covering a wider range of TAAs or somatic tumour alterations, and increasing the possibility of overcoming vaccine resistance by inducing a humoral and cell-mediated immune response [1]. Covering a wider range of TAAs or somatic tumour alterations is the second advantage of nucleic acid vaccines [6]. Second, nucleic acid vaccines are less constrained by the human HLA types and are more likely to induce a larger T cell response because they may encode full-length tumour antigens and enable antigen presenting cells (APCs) to present or cross-present several epitopes with both class I and II patient-specific HLA [7]. This is because nucleic acid vaccines can encode full-length tumour antigens and enable APCs to present or cross-present several epitopes [8]. Nucleic acid vaccines, such as mRNA or DNA vaccines, have the potential to encode a broad range of tumor antigens [5]. Unlike traditional protein-based vaccines, which typically target a limited number of specific antigens, nucleic acid vaccines can be designed to produce a variety of antigens simultaneously. This characteristic allows for a broader immune response against diverse tumor-associated antigens [6]. For example, in preclinical studies, researchers have developed mRNA vaccines encoding multiple tumor antigens, including neoantigens specific to individual patients, resulting in enhanced antitumor immune responses [5].

HLA (human leukocyte antigen) molecules play a critical role in presenting antigens to the immune system [9]. However, the genetic diversity of HLA types across individuals can pose a challenge for vaccine development, as a vaccine targeting one HLA type may not be effective for individuals with different HLA types [10]. Nucleic acid vaccines offer advantages in this regard. By encoding the antigen directly in the mRNA or DNA sequence, nucleic acid vaccines can bypass the need for HLA matching. The produced antigen is processed by the recipient's cells, leading to the presentation of peptides on the cell surface in a manner that is independent of the individual's HLA type [8]. This ability to generate a broader immune response regardless of HLA type has been demonstrated in several studies [10]. For instance, in a clinical trial evaluating an mRNA-based cancer vaccine, personalized neoantigens were shown to induce immune responses across various HLA types, suggesting the potential for broad applicability [9].

Nucleic acid vaccines are risk-free for use in both preventive and therapeutic contexts since they do not transmit infections and their production does not include any protein or virus-derived contaminations [6]. In recent years, mRNA vaccine has emerged as a potentially useful alternative to DNA vaccine for using in the prevention of infectious illnesses and the treatment of cancer [11]. As contrast to DNA, the use of mRNA as a cancer vaccine approach has a number of advantages, including the following: Once RNA has been taken up into the cytoplasm, the antigen (or antigens) of interest may be translated from mRNA in a single step in cells that are dividing as well as in cells that are not dividing [5]. mRNA vaccines, in contrast to DNA vaccines, often have higher rates and levels of protein synthesis [2]. This is due to the fact that mRNA vaccines cannot integrate into the genome sequence, meaning that they are not susceptible to insertional mutagenesis [1]. The feasibility of creating an mRNA vaccine was first reported in the year 1990, when it was discovered that in vitro transcription (IVT) mRNA could be effectively generated in mouse skeletal muscle cells by the process of direct injection into animals [6]. It is possible that worries over mRNA instability, poor in vivo transport, and highly intrinsic innate immunogenicity contributed to the fact that this first attempt did not result in extensive study on the production of mRNA vaccines [7]. mRNA vaccination has become a more practical choice as a result of significant technological developments that have taken place over the last several decades [11]. The RNA may be made more resistant to RNases, more stable, and more translation-friendly by making various modifications to the mRNA backbone and the untranslated regions [12]. mRNA products are now accessible without double-stranded contaminations as a result of developments in purifying procedures [13]. This helps to reduce the non-specific activation of the body's innate immune system [7]. The incorporation of messenger RNA (mRNA) into delivery vehicles, including as lipid nanoparticles (LNPs), polymers, and peptides, has led to an improvement in the distribution of mRNA in living organisms [8]. Finally, mRNAs have been discovered to be useful in IVT procedures in a broad manner [6]. Scale-up manufacturing has progressed to the point where mRNA vaccines provide substantial advantages over traditional immunisation approaches [4].

These advantages include decreased production costs and the possibility for wider use. In terms of cancer treatment, clinical trials have mostly concentrated on non-replicating mRNAs up to this point [1]. Self-amplifying mRNAs, also known as SAM, have garnered a lot of attention and are now being investigated for potential use in the treatment of cancer and infectious illnesses [6]. This is due to the fact that SAM are more cost-effective in the long term and have a lower impact per dosage [7]. More than twenty immunotherapies based on mRNA have progressed to the clinical trial stage, and the outcomes of these trials have been promising in the treatment of solid tumours [6, 7]. Furthermore, mRNA vaccines provide a considerable edge over anti-cancer immunotherapies when it comes to preventing the spread of the coronavirus infection over the globe [6]. Since the FDA in the United States has given its approval to two mRNA-based vaccines, one from Pfizer-BioNTech and one from Moderna, for emergency use in preventing COVID-19, the mRNA vaccine field will encompass a dramatic increase in market value and attract widespread interest in both cancer and infectious disease applications [2, 6, 8]. According to the findings, cancer immunotherapies might benefit from the use of mRNA vaccines in order to overcome certain obstacles.

This review article covers a range of topics related to mRNA vaccines in cancer immunotherapy. It begins with a discussion of basic mRNA vaccine pharmacology and recent advances in mRNA vaccine technology. The article then examines the optimization of mRNA translation and stability, modulation of immunogenicity, and progress in mRNA vaccine delivery. Various delivery methods are discussed, including ex vivo loading of DCs, injection of naked mRNA in vivo, physical delivery methods in vivo, protamine, and cationic lipid and polymer-based delivery. The review covers the development of mRNA cancer vaccines, including DC mRNA cancer vaccines and direct injection of mRNA cancer vaccines. It also highlights therapeutic considerations and challenges, good manufacturing practice production, and regulatory aspects of mRNA vaccines. Strategies to improve mRNA translation efficiency and overcome innate immunogenicity are examined, including the modification of the five-prime cap, optimization of untranslated regions, codon optimization of open reading frames, poly(a) tail modification, nucleoside modified mRNA, and purification of IVT-mRNA. The article also discusses the immunogenicity of mRNA and paradoxical effects in cancer immunotherapy, as well as self-amplifying mRNA vaccines, their structure, advantages, and deliveries. The review covers the delivery of mRNA cancer vaccines, including the rationale for lipid nanoparticles to maximize delivery efficiency and immunogenicity, mechanistic studies, and additional functional modifications of LNPs, LNP mRNA vaccine from formulation to manufacturing, polymer-based mRNA delivery systems, peptide-based mRNA delivery systems, and other formulations used in mRNA delivery. The article also examines the injection routes of mRNA cancer vaccines and provides a clinical overview of mRNA cancer vaccines. Finally, the review discusses mRNA encoding immunostimulants, mRNA vaccine encoding tumor-associated antigens, mRNA vaccine encoding neoantigen, personalized vaccines, and concludes with future perspectives on the development of RNA-based treatments in cancer immunotherapy.

## Cancer immunotherapies

Cancer immunotherapy is a revolutionary approach to treating cancer that harnesses the power of the immune system to recognize and destroy cancer cells [14]. The immune system plays a crucial role in detecting and eliminating abnormal cells in the body, including cancer cells [15]. However, cancer cells have developed various mechanisms to evade the immune system and continue to grow unchecked [14]. Immunotherapy aims to enhance and activate the body's immune response against cancer cells, helping the immune system to recognize and eliminate them effectively [16].

### Immune checkpoint inhibitors

Immune checkpoint inhibitors are a type of cancer immunotherapy that target molecules known as checkpoints on immune cells [17]. These checkpoints act as regulators or "brakes" on the immune system, preventing excessive immune responses that can lead to autoimmune reactions [16]. One of the well-known checkpoint molecules is called programmed cell death protein 1 (PD-1). It is expressed on the surface of certain immune cells, including T cells, which play a crucial role in recognizing and eliminating cancer cells [18]. Another checkpoint molecule is cytotoxic T-lymphocyte-associated protein 4 (CTLA-4), which is primarily found on the surface of regulatory T cells [19]. Cancer cells often exploit these checkpoint molecules to evade immune detection and attack. They can express ligands (such as PD-L1) that bind to the checkpoints on immune cells, sending inhibitory signals that dampen the immune response. By doing so, cancer cells can avoid being targeted and destroyed by the immune system [19].

Immune checkpoint inhibitors work by blocking these inhibitory signals, thereby unleashing the immune system's ability to recognize and attack cancer cells. Specifically, these inhibitors bind to either PD-1 or CTLA-4, preventing the cancer cell's ligands from interacting with the checkpoint molecules [20]. As a result, the immune response is reinvigorated, and immune cells, particularly T cells, can better recognize and eliminate cancer cells. By removing these "brakes" on the immune system, checkpoint inhibitors enhance the host's anti-tumor immunity. They allow immune cells to infiltrate tumors more effectively, recognize cancer-specific antigens, and mount a robust immune response against the tumor [19]. This can lead to tumor shrinkage and improved outcomes for cancer patients. It's important to note that checkpoint inhibitors are used in the treatment of various types of cancer, including melanoma, lung cancer, kidney cancer, bladder cancer, and others. They have shown significant success in some patients, with durable responses and improved survival rates. However, their effectiveness can vary depending on the type of cancer and individual patient factors [20].

### CAR-T cell therapy

CAR-T cell therapy is an innovative form of cancer immunotherapy that involves modifying a patient's own T cells to enhance their ability to recognize and attack cancer cells [21]. The process begins by collecting the patient's T cells from their blood. These T cells are then genetically engineered to express a CAR on their surface. The CAR is designed to recognize a specific antigen present on the surface of cancer cells [22]. Once the CAR-T cells are generated in the laboratory, they are infused back into the patient's body. The modified CAR-T cells can now specifically target and bind to cancer cells expressing the targeted antigen, initiating a potent immune response against the tumor. This therapy has shown remarkable success in treating certain types of blood cancers, such as leukemia and lymphoma, where the targeted antigen is abundantly expressed [23].

CAR-T cell therapy represents a personalized and highly targeted approach that harnesses the power of the patient's immune system to fight cancer. Once infused back into the patient, the CAR-T cells multiply and persist in the body, allowing for a sustained anti-tumor response [24]. The CAR-T cells have the ability to recognize and eliminate cancer cells throughout the body, including in hard-to-reach areas. This makes CAR-T cell therapy particularly effective against cancers that have spread or have been resistant to other treatments. One of the key advantages of CAR-T cell therapy is its specificity [24]. The CAR is designed to target a specific antigen present on cancer cells, minimizing damage to healthy cells. This targeted approach reduces the risk of off-target side effects commonly associated with traditional cancer treatments like chemotherapy and radiation [25]. Despite its success, CAR-T cell therapy does have potential side effects. The activation of the immune system can lead to an excessive immune response, known as cytokine release syndrome (CRS). CRS can cause flu-like symptoms, fever, low blood pressure, and in severe cases, organ damage. Another potential side effect is neurotoxicity, which can lead to confusion, seizures, and other neurological symptoms [26]. However, medical professionals closely monitor patients receiving CAR-T cell therapy to manage and mitigate these potential side effects.

CAR-T cell therapy has revolutionized the field of cancer treatment, particularly for certain types of blood cancers [27]. It has demonstrated remarkable efficacy in inducing long-term remissions and even cures in some patients. Ongoing research and clinical trials are exploring the application of CAR-T cell therapy to other types of cancer, with the hope of expanding its benefits to a wider range of patients [25]. Additionally, CAR-T cell therapy has shown promising results in pediatric patients with relapsed or refractory cancers [24]. Children with acute lymphoblastic leukemia who have not responded to standard treatments have achieved significant remissions with CAR-T cell therapy. This breakthrough therapy has provided a new treatment option for young patients who previously had limited options [21].

CAR-T cell therapy is continually evolving and improving. Researchers are exploring ways to enhance its effectiveness and reduce side effects. One area of focus is the development of "second-generation" and "third-generation" CARs that incorporate additional signaling domains to enhance CAR-T cell activation and persistence [27]. These advancements aim to further improve the anti-tumor response and potentially broaden the applicability of CAR-T cell therapy to other types of cancer. Moreover, efforts are underway to overcome challenges related to solid tumors, which have proven more complex to target with CAR-T cell therapy compared to blood cancers. Strategies such as combining CAR-T cell therapy with other treatments, including immune checkpoint inhibitors or CAR-T cells targeting multiple antigens, are being explored to improve outcomes in solid tumors [26].

### Tumor-infiltrating lymphocyte (TIL) therapy

Tumor-infiltrating lymphocyte (TIL) therapy is a form of cancer immunotherapy that harnesses the power of a patient's own immune system to fight against cancer [28]. In TIL therapy, immune cells called lymphocytes are isolated from a tumor sample obtained from the patient. These lymphocytes, which have infiltrated the tumor, are then expanded and activated in the laboratory [29]. Once a sufficient number of TILs have been generated, they are infused back into the patient's body [30]. The goal of TIL therapy is to enhance the host's anti-tumor immune response by providing a larger population of activated T cells that can specifically recognize and target cancer cells. By reintroducing these modified TILs, the therapy aims to create a more potent immune response against the tumor [29].

TIL therapy also has the potential to modify the tumor microenvironment by increasing the infiltration of immune cells into the tumor, thereby creating a more hostile environment for cancer cells and improving the overall anti-tumor immune response [31]. TIL therapy offers a promising approach to treating cancer by leveraging the patient's own immune system to specifically target and eliminate cancer cells [32]. Upon infusion, the expanded TILs migrate to the tumor site, where they engage with cancer cells through the recognition of tumor-specific antigens. This interaction activates the TILs, leading to the release of cytotoxic molecules and the secretion of immune-stimulating cytokines. The cytotoxic molecules, such as perforin and granzymes, enable the TILs to directly attack and kill cancer cells [32]. Additionally, the secreted cytokines help recruit and activate other immune cells, further enhancing the anti-tumor immune response.

TIL therapy not only focuses on the direct elimination of cancer cells but also aims to modify the tumor microenvironment [33]. Tumors often create an immunosuppressive environment that inhibits immune cell function and allows the cancer cells to evade immune detection. However, the introduction of activated TILs can disrupt this immune suppression by promoting immune cell infiltration and altering the balance of immune cell types within the tumor [32]. This shift in the tumor microenvironment can create a more favorable setting for anti-tumor immune responses to occur. It's important to note that TIL therapy is still an area of active research, and its effectiveness can vary depending on several factors, including the type and stage of cancer, the quality and quantity of TILs, and the overall immune status of the patient [32].

Ongoing studies are focused on optimizing TIL expansion techniques, improving the selection of tumor-specific TILs, and exploring combination therapies to enhance the efficacy of TIL-based treatments. In addition to its potential as a standalone therapy, TIL therapy is also being investigated in combination with other cancer treatments [32]. For example, researchers are exploring the use of TIL therapy alongside immune checkpoint inhibitors to further enhance the anti-tumor immune response. Immune checkpoint inhibitors can alleviate the brakes on the immune system, allowing TILs to exert their full potential in targeting cancer cells [34]. Moreover, ongoing efforts are being made to overcome some of the challenges associated with TIL therapy [32].

One such challenge is the limited availability of TILs from some tumor types or patients with low TIL infiltration. Researchers are exploring strategies to generate and expand TILs from small tumor samples or using techniques such as genetic engineering to improve TIL functionality [32]. Another area of interest is the development of personalized TIL therapy, where TILs are specifically tailored to target the unique antigens present in an individual patient's tumor. This approach involves identifying the specific antigens expressed by the patient's tumor and selecting or engineering TILs that can recognize and attack those antigens [32]. Personalized TIL therapy has shown promising results in early clinical trials and may improve treatment efficacy by targeting tumor-specific antigens.

As research in TIL therapy progresses, it holds the potential to become an integral part of the cancer treatment landscape [32]. By harnessing the power of the immune system, TIL therapy offers a targeted and personalized approach to combat cancer, potentially leading to improved outcomes and better quality of life for patients. Continued advancements and clinical studies will provide further insights into the optimal use and potential of TIL therapy in the fight against cancer [31].

### Therapeutic vaccines

Therapeutic vaccines can be developed using different strategies. One approach is to use tumor-specific antigens derived from the patient's own tumor cells [35]. These antigens are unique to the cancer cells and not present in healthy cells, making them ideal targets for the immune system. By presenting these tumor-specific antigens to the immune system, the therapeutic vaccine helps train immune cells to recognize and attack cancer cells specifically. Another strategy involves using immune-stimulating molecules called adjuvants [36]. Adjuvants are included in the vaccine formulation to enhance the immune response. They can activate immune cells, promote antigen presentation, and improve the overall effectiveness of the vaccine [37]. Adjuvants can be designed to trigger specific immune pathways or amplify immune responses against cancer cells [37].

Therapeutic vaccines are typically administered through injections, either subcutaneously or intramuscularly. The vaccination process may involve multiple doses over a period of time to optimize the immune response [38]. In some cases, the vaccines may be combined with other immunotherapies or conventional treatments like chemotherapy or radiation therapy to enhance their effectiveness.

Therapeutic vaccines offer several advantages in the field of cancer immunotherapy [39]. One major advantage is their potential for personalized medicine. Each patient's tumor is unique, and therapeutic vaccines can be tailored to target specific antigens present in their cancer cells. This personalized approach can enhance the vaccine's effectiveness by focusing on the individual's specific tumor characteristics [40]. Furthermore, therapeutic vaccines have the potential to induce immune memory. This means that even after the initial treatment, the immune system may retain the ability to recognize and respond to cancer cells if they reappear. This immune memory could provide long-term protection against cancer recurrence, offering a durable and sustained therapeutic effect [38]. Additionally, therapeutic vaccines are generally well-tolerated with manageable side effects. They do not typically cause the severe adverse reactions associated with traditional cancer treatments such as chemotherapy or radiation therapy [41]. This makes therapeutic vaccines an attractive option for patients who are unable to tolerate or have completed standard treatments and are seeking alternative therapies [38]. However, challenges remain in the development and implementation of therapeutic vaccines. One hurdle is identifying the most appropriate tumor antigens to target, as cancer cells can have a complex and heterogeneous antigen profile [37]. Additionally, tumors employ various mechanisms to evade immune detection and suppress the immune response, which can limit the effectiveness of therapeutic vaccines [36]. Overcoming these immunosuppressive mechanisms is an active area of research to improve the efficacy of therapeutic vaccines. Therapeutic vaccines hold great promise in harnessing the power of the immune system to target and eliminate cancer cells [35]. Their personalized nature, potential for immune memory, and relatively favorable side effect profile make them a compelling avenue for cancer treatment. As research advances and our understanding of the immune response to cancer deepens, therapeutic vaccines are likely to play an increasingly important role in the broader landscape of cancer immunotherapy [38].

### Adoptive cell transfer

Adoptive cell transfer is a cancer immunotherapy approach that involves transferring immune cells, such as TILs or genetically modified T cells, into the patient [42]. The process begins by isolating immune cells from the patient's tumor, which often contains TILs that have already recognized the cancer cells [43]. These TILs are then expanded and activated in the laboratory to enhance their anti-tumor capabilities. In some cases, genetic modifications may be made to these cells to improve their effectiveness or to introduce specific receptors that recognize tumor antigens [44]. Once the cells have been prepared, they are infused back into the patient, where they can seek out and attack cancer cells more effectively than the patient's own immune system alone [45]. By transferring these highly specialized immune cells, adoptive cell transfer aims to bolster the host's anti-tumor immunity and modify the tumor microenvironment [46]. The transferred cells can infiltrate the tumor, recognize cancer cells more efficiently, and mount a targeted immune response, leading to tumor regression. Additionally, the presence of these activated immune cells can influence the tumor microenvironment by promoting immune cell infiltration and altering the balance of immune cell types within the tumor, creating a more hostile environment for cancer cells [47].

Adoptive cell transfer holds promise as a powerful tool in cancer treatment, harnessing the potential of the immune system to fight against cancer. Through adoptive cell transfer, the immune cells that are transferred into the patient are specifically selected and engineered to recognize and target cancer cells [48]. This approach can overcome some of the limitations of the patient's own immune system, which may not have been able to effectively recognize or eliminate the cancer cells on its own. One example of adoptive cell transfer is CAR-T cell therapy, where T cells are genetically modified to express chimeric antigen receptors (CARs) on their surface [49]. These CARs are designed to recognize specific tumor antigens, enabling the T cells to specifically target cancer cells [50]. Once infused into the patient, these engineered CAR-T cells can multiply and persist in the body, continuously searching for and attacking cancer cells. Another example is the use of TILs, which are immune cells that have naturally infiltrated the tumor [49]. TILs are isolated from the tumor, expanded in the laboratory, and then reinfused back into the patient. These TILs, already primed to recognize cancer cells, can exert a potent anti-tumor immune response [48]. By transferring a large number of activated TILs, the immune response against the tumor is enhanced, leading to tumor regression. Adoptive cell transfer not only enhances the host's anti-tumor immunity but also has the potential to modify the tumor microenvironment [50]. The transferred immune cells can secrete cytokines and other signaling molecules that can recruit additional immune cells to the tumor site [49]. This immune cell infiltration can lead to changes in the tumor microenvironment, such as increased presence of effector immune cells and reduced suppressive factors. These modifications create a more immune-favorable environment, allowing for better immune surveillance and targeting of the cancer cells [45]. Adoptive cell transfer is a promising cancer immunotherapy that leverages the power of engineered or expanded immune cells to enhance the host's immune response against cancer [44]. By specifically targeting cancer cells and modifying the tumor microenvironment, adoptive cell transfer holds great potential in improving outcomes for patients with various types of cancer. Ongoing research and advancements in this field are continually refining and expanding the applications of adoptive cell transfer in cancer treatment [48].

## Basic mRNA vaccine pharmacology

**Fig. 1:**
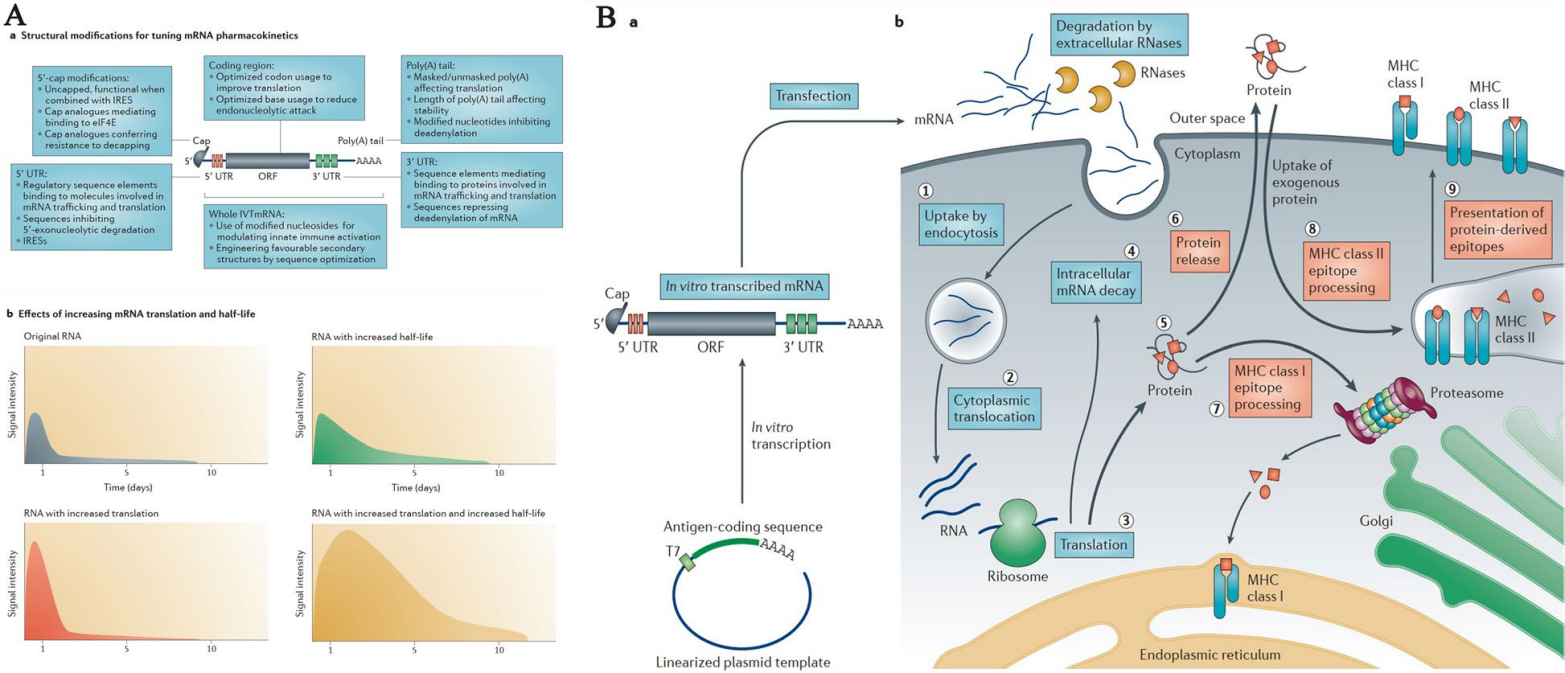
**A** Adjusting mRNA Medicine Dosage Pharmacokinetics. a) Crucial structural components of in vitro transcribed (IVT) mRNA and approaches for their alterations. b) Based on the individual or combined use of these elements (such as modification of caps, UTRs, or poly(A) tails), the protein product's expression duration and kinetic profile can be controlled and optimized. eIF4E represents eukaryotic translation initiation factor 4E; IRES refers to the internal ribosome entry site; and ORF denotes open reading frame. Reprinted from [51] with permission from Springer Nature. **B** Fundamentals of mRNA-based antigen pharmacology. a) A linear DNA plasmid containing the antigen-encoding sequence is employed for in vitro transcription. The transcribed mRNA consists of the cap, 5′ and 3′ UTRs, the open reading frame (ORF), and the poly(A) tail, which influence the mRNA's translational activity and stability once introduced into cells. b) Step 1: A portion of the foreign mRNA avoids degradation by common RNases and is taken up by cell-specific mechanisms (such as macropinocytosis in immature dendritic cells) into endosomal pathways. Step 2: The release of mRNA into the cytoplasm is not entirely understood. Step 3: Host cell protein synthesis machinery translates the mRNA. mRNA translation's rate-limiting step involves eukaryotic eIF4E binding to the cap structure. The formation of circular structures and active translation result from mRNA binding to ribosomes, eIF4E, eIF4G, and poly(A)-binding protein. Step 4: Exonucleases catalyze the termination of translation via mRNA degradation. Decapping enzymes D CP1, DCP2, and DCPS hydrolyze the cap, followed by the digestion of residual mRNA by 5′–3′ exoribonuclease 1 (XRN1). Degradation might be delayed if mRNA is silenced and located within cytoplasmic processing bodies. Alternatively, exosomal endonucleolytic cleavage of mRNA may take place. Various mechanisms control the breakdown of aberrant mRNA (such as mRNA with a premature stop codon). Step 5: The translated protein undergoes post-translational modifications based on the host cell's characteristics. The synthesized protein can then function within the cell it was produced in. Step 6: Alternatively, the protein is secreted and can function through autocrine, paracrine, or endocrine pathways. Step 7: For immunotherapeutic mRNA application, the protein must be broken down into antigenic peptide epitopes. These peptides are loaded onto major MHC molecules, which present the antigens to immune effector cells. Proteasomes degrade cytoplasmic proteins, which are then transported to the endoplasmic reticulum and loaded onto MHC class I molecules for presentation to CD8 + cytotoxic T lymphocytes. Almost all cells express MHC class I molecules. Step 8: In antigen-presenting cells, the protein must be directed to MHC class II loading compartments to obtain T cell assistance for a stronger, lasting immune response. This can be achieved by incorporating routing signal-encoding sequences into the mRNA. Additionally, DCs can process and load exogenous antigens onto MHC class I molecules through a mechanism called cross-priming. Step 9: Antigens derived from the protein can be displayed on the cell surface by both MHC class I and MHC class II molecules, enabling the immune system to recognize and respond to them accordingly. Reprinted from [51] with permission from Springer Nature

The translation of protein-encoding DNA into mRNA is the first step in the synthesis of proteins by ribosomes in the cytoplasm. Figure 1-A illustrates the process of adjusting mRNA medicine dosage pharmacokinetics through the manipulation of crucial structural components in IVT mRNA. By altering elements such as the cap structure, untranslated regions (UTRs), and polyadenylated (poly(A)) tails, researchers can effectively control and optimize the expression duration and kinetic profile of the protein product. This approach involves modulating the interaction of eukaryotic translation initiation factor 4E (eIF4E) with the mRNA cap, leveraging the internal ribosome entry site (IRES) for alternative translation initiation, and adjusting the open reading frame (ORF) to fine-tune protein production. As a result, this method offers a promising avenue for the development of personalized mRNA medicine with enhanced efficacy and reduced side effects.

Both non-replicating and virally produced, self-amplifying RNA are now being investigated as vaccine candidates: non-replicating RNA and self-amplifying RNA. The antigen of interest and the 5′ and 3′ UTRs are encoded by conventional mRNA-based vaccines, whereas self-amplifying RNAs encode not only the antigen of interest but also the viral replication machinery, which allows for intracellular RNA amplification and abundant protein expression. RNA polymerase (T7, T3, or Sp6) is used to transcribe the linear DNA template into mRNA, which is then used to transcribe the mRNA in vitro. The final product should include an open reading frame that encodes the protein of interest, flanking UTRs, a 5′ cap, and a poly(A) tail, if possible. As a result, the mRNA has been made to look and behave like fully processed mature mRNA molecules seen in the cytoplasm of eukaryotic cells in the natural world. Unprocessed mRNA is rapidly destroyed by extracellular RNases and does not undergo effective internalization. The result has been the development of a large number of different in vitro and in vivo transfection agents that aid in the absorption of mRNA into cells while also protecting it from destruction.

Once the mRNA reaches the cytosol, the cellular translation machinery begins to generate protein, which is then subjected to post-translational modifications, culminating in a correctly folded and completely functioning protein. As previously stated, this aspect of mRNA pharmacology is especially helpful for vaccinations and protein replacement treatments that need the delivery of cytosolic or transmembrane proteins to the appropriate cellular compartments in order to be effective. In the end, IVT mRNA is destroyed by natural physiological processes, lowering the risk of toxicity from metabolites. Figure 1-B illustrates the fundamentals of mRNA-based antigen pharmacology. The process begins with in vitro transcription of a linear DNA plasmid containing the antigen-encoding sequence, producing an mRNA molecule with various components influencing its translational activity and stability. The foreign mRNA then enters cells through specific mechanisms, such as macropinocytosis in immature dendritic cells, and undergoes several steps, including cytoplasmic release, translation by host cell machinery, and degradation. The rate-limiting step of mRNA translation involves eukaryotic translation initiation factors (eIF4E and eIF4G) and poly(A)-binding protein, which facilitate the formation of circular structures for active translation.

Following translation, the synthesized protein undergoes post-translational modifications and may either function within the producing cell or be secreted to act through autocrine, paracrine, or endocrine pathways. In immunotherapeutic applications, the protein is degraded into antigenic peptide epitopes and loaded onto major MHC molecules to elicit immune responses. MHC class I and class II molecules present antigens to immune effector cells, with class II molecules typically requiring additional routing signals encoded into the mRNA. Dendritic cells (DCs) play a crucial role in this process by processing exogenous antigens and cross-priming. Ultimately, the immune system recognizes and responds to the antigens presented on the cell surface by both MHC class I and II molecules.

## Recent advances in mRNA vaccine technology

In recent years, a number of mRNA vaccine platforms have been generated and confirmed in investigations of immunogenicity and effectiveness [53]. Through the use of genetic engineering, synthesized mRNA is more readily translated than ever before [54]. Table 1 provides a summary of the different types of mRNA cancer vaccines and mentioned several categories of mRNA cancer vaccines. There is a wide array of mRNA cancer vaccines under development, each with distinct mechanisms of action and unique advantages and disadvantages [55]. These vaccines range from DC mRNA cancer vaccines, which involve the ex vivo loading of patient-derived DCs, to direct injection of mRNA into the tumor or surrounding tissue [56]. Some mRNA cancer vaccines encode for specific tumor-associated antigens or neoantigens, while others utilize self-amplifying RNA vectors or lipid nanoparticles for improved delivery. Other strategies include combining mRNA with adjuvants, immune checkpoint inhibitors, gene editing tools, or novel delivery systems to enhance the immune response against cancer cells [11]. Despite the diversity of approaches, mRNA cancer vaccines generally face challenges in terms of stability, immunogenicity, and manufacturing complexity. Additionally, while many of these vaccines have shown promise in preclinical studies, their efficacy in early clinical trials remains limited. Nonetheless, these innovative treatments hold great potential in the fight against cancer and warrant further research and development.

**Table 1:** The different types of mRNA cancer vaccines

mRNA Cancer Vaccine Type	Mechanism of Action	Advantages	Disadvantages	Immunogenicity	Efficacy	Safety	Stability	Reference
DC mRNA cancer vaccines	Direct ex vivo loading of patient-derived DCs	Highly personalized, high antigen expression, long-lasting immune response	Requires specialized personnel and equipment, complex manufacturing, short half-life in vivo	High	High in preclinical studies, moderate in early clinical trials	Generally safe, rare cases of autoimmune response	Requires refrigeration or cryopreservation, short shelf life in vivo	[55]
Direct injection of mRNA cancer vaccines	Direct injection of mRNA into the tumor or surrounding tissue	Simplicity, low cost, no requirement for specialized personnel or equipment	Low antigen expression, limited potential for systemic immune response, requires multiple injections	Low to moderate	Moderate in preclinical studies, limited in early clinical trials	Generally safe, rare cases of autoimmune response	Requires refrigeration or cryopreservation, short half-life in vivo	[56]
mRNA cancer vaccines encoding immunostimulants	mRNA encoding for cytokines or other immune system activators	Potent immune stimulation, potential for systemic response	Requires identification and optimization of appropriate immunostimulant, potential for excessive inflammation	High	Moderate in preclinical studies, limited in early clinical trials	Generally safe, rare cases of autoimmune response	Requires refrigeration or cryopreservation, short half-life in vivo	[11]
mRNA cancer vaccines encoding tumor-associated antigens	mRNA encoding for specific tumor-associated antigens	Highly personalized, specific targeting of tumor cells, potential for long-lasting immune response	Requires identification and optimization of appropriate antigen, limited potential for systemic response	Moderate to high	Moderate in preclinical studies, limited in early clinical trials	Generally safe, rare cases of autoimmune response	Requires refrigeration or cryopreservation, short half-life in vivo	[57]
mRNA cancer vaccines encoding neoantigens	mRNA encoding for patient-specific neoantigens	Highly personalized, specific targeting of tumor cells, potential for long-lasting immune response	Requires identification and optimization of appropriate neoantigen, limited potential for systemic response	High	Limited in preclinical studies and early clinical trials	Generally safe, rare cases of autoimmune response	Requires refrigeration or cryopreservation, short half-life in vivo	[58]
Self-amplifying mRNA cancer vaccines	mRNA encoding for self-amplifying RNA vectors	High antigen expression, potential for long-lasting immune response	Complex manufacturing, short half-life in vivo, potential for excessive inflammation	High	Moderate in preclinical studies, limited in early clinical trials	Generally safe, rare cases of autoimmune response	Requires refrigeration or cryopreservation, short half-life in vivo	[59]
Lipid nanoparticle (LNP) mRNA cancer vaccines	mRNA encapsulated in lipid nanoparticles for delivery	High antigen expression, potential for systemic immune response, potential for enhanced cellular uptake	Complex manufacturing, potential for toxicity or adverse reactions to LNP, potential for immune recognition and clearance	High	Moderate in preclinical studies, limited in early clinical trials	Generally safe, rare cases of autoimmune response	Requires refrigeration or cryopreservation, short half-life in vivo	[59]
Peptide-based mRNA cancer vaccines	mRNA encoding for specific peptides	Highly specific targeting of tumor cells, low potential for toxicity or adverse reactions, potential for systemic immune response	Limited potential for long-lasting immune response, requires identification and optimization of appropriate peptide	Low to moderate	Limited in preclinical studies and early clinical trials	Generally safe, rare cases of autoimmune response	Requires refrigeration or cryopreservation, short half-life in vivo	[60]
Adjuvant-assisted mRNA cancer vaccines	mRNA combined with adjuvants to enhance immune response	Potential for potent immune stimulation, potential for systemic response	Requires identification and optimization of appropriate adjuvant, potential for excessive inflammation or toxicity	High	Limited in preclinical studies and early clinical trials	Generally safe, rare cases of autoimmune response	Requires refrigeration or cryopreservation, short half-life in vivo	[61]
Non-replicating mRNA cancer vaccines	mRNA encoding for non-replicating viral antigens	Highly specific targeting of tumor cells, potential for long-lasting immune response	Requires identification and optimization of appropriate antigen, limited potential for systemic response	Moderate to high	Limited in preclinical studies and early clinical trials	Generally safe, rare cases of autoimmune response	Requires refrigeration or cryopreservation, short half-life in vivo	[62]
Nanoparticle-assisted mRNA cancer vaccines	mRNA combined with nanoparticle delivery systems for enhanced uptake	High antigen expression, potential for systemic immune response, enhanced cellular uptake	Complex manufacturing, potential for toxicity or adverse reactions to nanoparticle, potential for immune recognition and clearance	High	Moderate in preclinical studies, limited in early clinical trials	Generally safe, rare cases of autoimmune response	Requires refrigeration or cryopreservation, short half-life in vivo	[63]
mRNA cancer vaccines with checkpoint inhibitors	mRNA encoding for checkpoint inhibitors to enhance anti-tumor immune response	Potential for enhanced immune response, specific targeting of tumor cells	Limited potential for long-lasting immune response, potential for excessive inflammation or toxicity	High	Limited in preclinical studies and early clinical trials	Generally safe, rare cases of autoimmune response	Requires refrigeration or cryopreservation, short half-life in vivo	[64]
mRNA cancer vaccines with CAR-T cells	mRNA encoding for CAR-T cells for targeted immune response	Highly specific targeting of tumor cells, potential for long-lasting immune response	Complex manufacturing, potential for toxicity or adverse reactions to CAR-T cells, potential for excessive inflammation or toxicity	High	Limited in preclinical studies and early clinical trials	Generally safe, rare cases of autoimmune response	Requires refrigeration or cryopreservation, short half-life in vivo	[11]
mRNA cancer vaccines with gene editing tools	mRNA encoding for gene editing tools for targeted modification of tumor cells	Highly specific targeting of tumor cells, potential for long-lasting immune response	Requires identification and optimization of appropriate gene editing tools, potential for off-target effects	High	Limited in preclinical studies and early clinical trials	Generally safe, rare cases of autoimmune response	Requires refrigeration or cryopreservation, short half-life in vivo	[65]
mRNA cancer vaccines with adjuvant-loaded exosomes	mRNA combined with exosomes carrying adjuvants for enhanced immune response	High antigen expression, potential for systemic immune response, potential for long-lasting immune response	Requires optimization of appropriate exosome-adjuvant combination, potential for excessive inflammation or toxicity	High	Limited in preclinical studies and early clinical trials	Generally safe, rare cases of autoimmune response	Requires refrigeration or cryopreservation, short half-life in vivo	[66]
mRNA cancer vaccines with oncolytic viruses	mRNA encoding for oncolytic viruses for targeted destruction of tumor cells	Highly specific targeting of tumor cells, potential for long-lasting immune response	Requires identification and optimization of appropriate oncolytic virus, potential for off-target effects, potential for excessive inflammation or toxicity	High	Limited in preclinical studies and early clinical trials	Generally safe, rare cases of autoimmune response	Requires refrigeration or cryopreservation, short half-life in vivo	[67]
mRNA cancer vaccines with chimeric antigen receptor (CAR) mRNA	mRNA encoding for CARs for targeted immune response	Highly specific targeting of tumor cells, potential for long-lasting immune response	Complex manufacturing, potential for toxicity or adverse reactions to CARs, potential for excessive inflammation or toxicity	High	Limited in preclinical studies and early clinical trials	Generally safe, rare cases of autoimmune response	Requires refrigeration or cryopreservation, short half-life in vivo	[11]
mRNA cancer vaccines with bispecific T cell engagers (BiTEs)	mRNA encoding for BiTEs for targeted immune response	Highly specific targeting of tumor cells, potential for long-lasting immune response	Complex manufacturing, potential for toxicity or adverse reactions to BiTEs, potential for excessive inflammation or toxicity	High	Limited in preclinical studies and early clinical trials	Generally safe, rare cases of autoimmune response	Requires refrigeration or cryopreservation, short half-life in vivo	[68]
mRNA cancer vaccines with multiple antigens	mRNA encoding for multiple tumor-associated antigens	Potentially higher response rates, potential for broad targeting of tumor cells	Requires identification and optimization of appropriate antigens, potential for autoimmune response	High	Limited in preclinical studies and early clinical trials	Generally safe, rare cases of autoimmune response	Requires refrigeration or cryopreservation, short half-life in vivo	[69]
mRNA cancer vaccines with personalized neoantigens	mRNA encoding for personalized neoantigens specific to patient's tumor	Highly specific targeting of tumor cells, potential for long-lasting immune response	Requires sequencing and analysis of patient's tumor, potential for autoimmune response, complex manufacturing	High	Limited in preclinical studies and early clinical trials	Generally safe, rare cases of autoimmune response	Requires refrigeration or cryopreservation, short half-life in vivo	[70]
mRNA cancer vaccines with cytokine encoding mRNA	mRNA encoding for cytokines to enhance anti-tumor immune response	Potential for enhanced immune response, specific targeting of tumor cells	Limited potential for long-lasting immune response, potential for excessive inflammation or toxicity	High	Limited in preclinical studies and early clinical trials	Generally safe, rare cases of autoimmune response	Requires refrigeration or cryopreservation, short half-life in vivo	[71]
mRNA cancer vaccines with immune checkpoint inhibitors	mRNA encoding for immune checkpoint inhibitors to enhance anti-tumor immune response	Potential for enhanced immune response, specific targeting of tumor cells	Limited potential for long-lasting immune response, potential for excessive inflammation or toxicity	High	Limited in preclinical studies and early clinical trials	Generally safe, rare cases of autoimmune response	Requires refrigeration or cryopreservation, short half-life in vivo	[2]
mRNA cancer vaccines with immune-modulatory agents	mRNA encoding for immune-modulatory agents to enhance anti-tumor immune response	Potential for enhanced immune response, specific targeting of tumor cells	Limited potential for long-lasting immune response, potential for excessive inflammation or toxicity	High	Limited in preclinical studies and early clinical trials	Generally safe, rare cases of autoimmune response	Requires refrigeration or cryopreservation, short half-life in vivo	[69]
mRNA cancer vaccines with immunomodulatory genes	mRNA encoding for immunomodulatory genes to enhance anti-tumor immune response	Potential for enhanced immune response, specific targeting of tumor cells	Limited potential for long-lasting immune response, potential for excessive inflammation or toxicity	High	Limited in preclinical studies and early clinical trials	Generally safe, rare cases of autoimmune response	Requires refrigeration or cryopreservation, short half-life in vivo	[67]
mRNA cancer vaccines with tumor-derived extracellular vesicles	mRNA combined with extracellular vesicles derived from tumor cells to enhance immune response	High antigen expression, potential for systemic immune response, potential for long-lasting immune response	Requires optimization of appropriate extracellular vesicle-mRNA combination, potential for excessive inflammation or toxicity	High	Limited in preclinical studies and early clinical trials	Generally safe, rare cases of autoimmune response	Requires refrigeration or cryopreservation, short half-life in vivo	[72]
mRNA cancer vaccines with RNA sensors	mRNA encoding for RNA sensors to activate innate immune response and enhance anti-tumor immune response	Potential for enhanced immune response, specific targeting of tumor cells	Limited potential for long-lasting immune response, potential for excessive inflammation or toxicity	High	Limited in preclinical studies and early clinical trials	Generally safe, rare cases of autoimmune response	Requires refrigeration or cryopreservation, short half-life in vivo	[73]
mRNA cancer vaccines with novel delivery systems	mRNA combined with novel delivery systems for enhanced stability and efficient delivery	Potential for enhanced immune response, specific targeting of tumor cells, increased stability and efficiency of mRNA delivery	Requires optimization of appropriate delivery system, potential for toxicity or adverse reactions to delivery system	High	Limited in preclinical studies and early clinical trials	Generally safe, rare cases of autoimmune response	Requires refrigeration or cryopreservation, short half-life in vivo	[74]

**Fig. 2:**
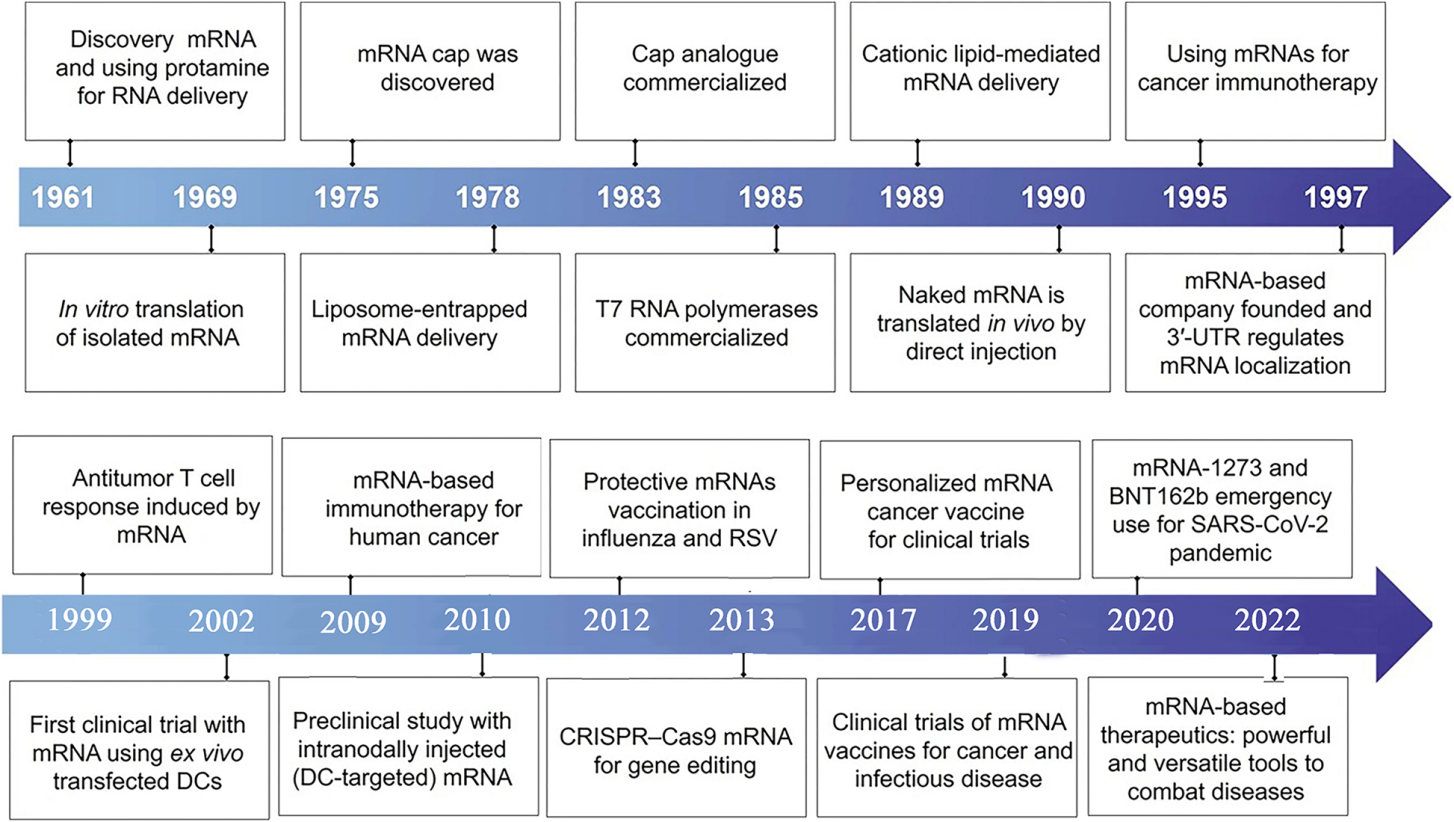
Essential breakthroughs and progress in mRNA-based treatment development. The creation of mRNA-based treatments can be split into three primary phases. Phase 1 (1961–1990) involves mRNA discovery, in vitro synthesis, and the construction of nucleic acid delivery systems, including mRNA identification, protamine uses for RNA delivery, in vitro mRNA translation, mRNA cap discovery, liposome-trapped mRNA delivery, commercialization of cap analogs and T7 RNA polymerases, cationic lipid-mediated mRNA delivery, and in vivo translation of naked mRNA through direct injection. Phase 2 (1990–2019) encompasses the accumulation of knowledge through numerous attempts and diverse applications, particularly protein replacement therapies and vaccination strategies for cancer and infectious diseases, such as mRNA-based cancer immunotherapy, founding of an mRNA-based company, 3′-UTR regulation of mRNA localization, antitumor T cell response triggered by mRNA, first clinical trial with mRNA using ex vivo transfected DCs, mRNA-based immunotherapy for human cancer, preclinical study with intranodally injected DC-targeted mRNA, protective mRNA vaccinations for influenza and respiratory syncytial virus, CRISPR-Cas9 mRNA for gene editing, and personalized mRNA cancer vaccines for clinical trials. Phase 3 (2019-present) sees mRNA-based therapeutics emerging as a disruptive technology, providing powerful and versatile tools for treating diseases, including clinical trials of mRNA vaccines for cancer and infectious diseases, as well as the emergency use of mRNA-1273 and BNT162b for the SARS-CoV-2 pandemic. Reprinted from [52] with permission from Springer Nature

The development of highly effective and non-toxic RNA carriers has allowed for the expression of antigens in vivo to be extended in certain circumstances [75]. Novel adjuvants are used in certain vaccination formulations, whereas others produce robust immune responses even in the absence of well-established adjuvants [76]. The significant advancements in these areas of mRNA engineering, as well as their implications for vaccination effectiveness, are summarized in the following section [53]. Figure 2 highlights the essential breakthroughs and progress in mRNA-based treatment development, which can be divided into three main phases.

### Optimization of mRNA translation and stability

**Fig. 3:**
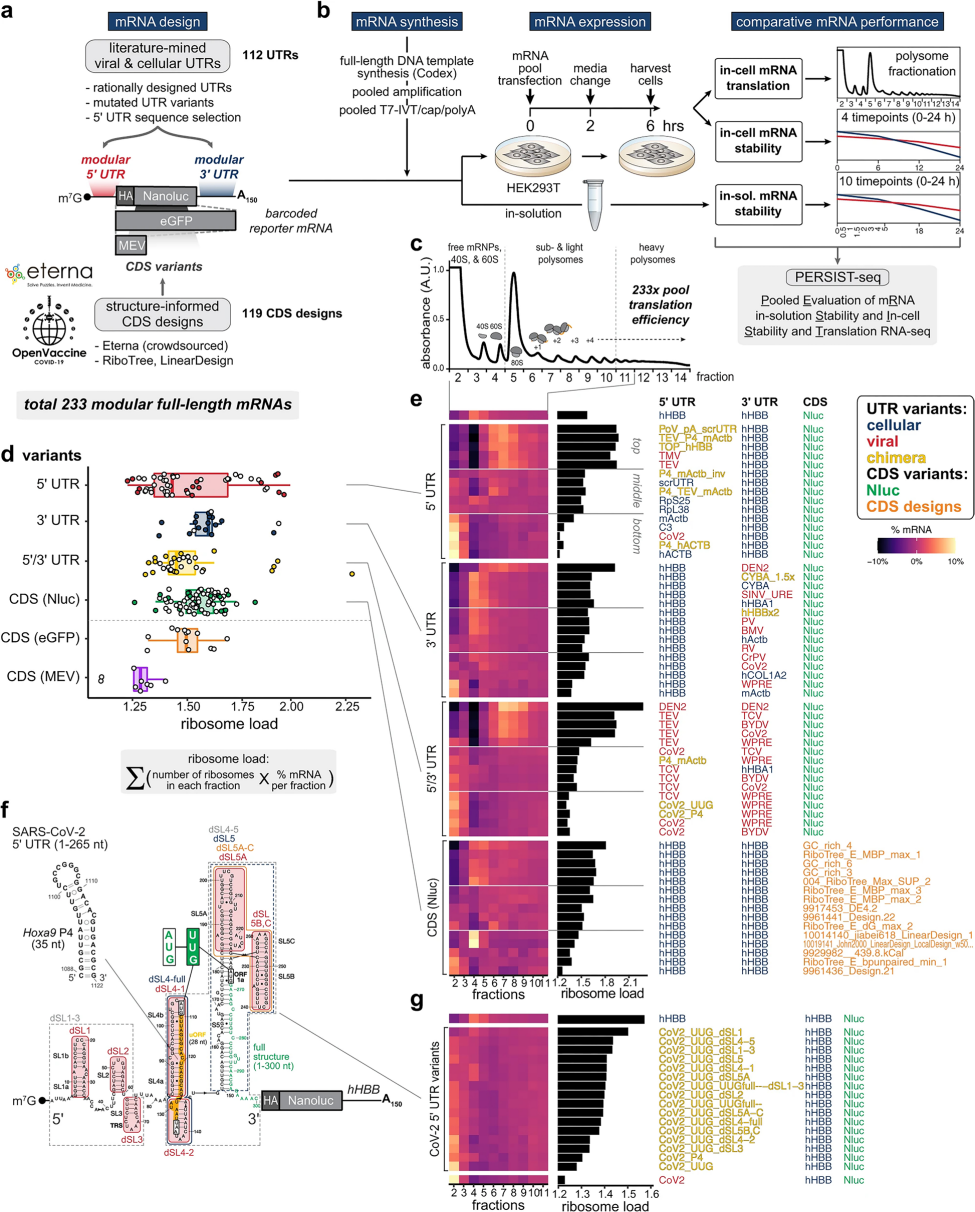
PERSIST-seq summary and representative ribosome load findings. **a** Schematic representation of the mRNA optimization process. 5′ and 3′ UTRs sourced from literature and rational design were merged with Eterna and algorithmically created coding sequences. All sequences underwent simultaneous experimental evaluation for in-solution and in-cell stability, as well as ribosome load. Unique 6–9 nt barcodes in the 3′ UTR of the mRNA design enabled tag counting via short-read sequencing. **b** Experimental layout for assessing in-solution and in-cell stability and ribosome load concurrently. mRNAs were in vitro transcribed, 5′ capped, and polyadenylated in a pooled format prior to HEK293T cell transfection or in-solution degradation exposure. Cells were then collected for sucrose gradient fractionation or in-cell degradation examination. **c** Polysome trace from a 233-mRNA pool transfected into HEK293T cells. **d** 5′ UTR variations exhibit greater variability in average ribosome load per construct, as determined by polysome sequencing. The ribosome load formula is provided. Box hinges display 25% quantile, median, and 75% quantile from left to right, while whiskers indicate lower or upper hinge ± 1.5 × interquartile range. **e** Heatmaps of polysome profiles for top, middle, and bottom five mRNA designs (based on ribosome load) from each design category. **f** SARS-CoV-2 5′ UTR secondary structure model, with highlighted mutations and substitutions. **g** Heatmaps of SARS-CoV-2 5′ UTR variant polysome profiles, sorted by ribosome load. Reprinted from [87] with permission from Springer Nature

Stability and translation of mRNA are significantly influenced by the 5′ and 3′ UTRs that surround the coding sequence, both of which are key issues for vaccine development [77]. Regulation sequences may be obtained from viral or Eukaryotic genes, and they have been shown to significantly prolong the half-life and boost the production of therapeutic mRNAs [78]. A 5′ cap structure is necessary for effective protein synthesis from mRNA [79]. A 5′ cap structure is required to successfully generate protein from mRNA [79]. Depending on the application, 5′ caps may be inserted during or after transcription using a vaccinia virus capping enzyme or synthetic cap or anti-reverse cap analogues [80]. To ensure that mRNA is translated and stable, a suitable length of poly(A) must be added to it, either directly from the encoding DNA template or by using poly(A) polymerase [81]. The codons utilized also affect protein translation [82]. It is typical practice to replace unusual codons with common synonymous codons that have abundant cognate tRNA in the cytosol to increase protein synthesis from mRNA, although this paradigm has been questioned [83]. Enriching G:C composition in sequences has been shown to increase steady-state mRNA levels in vitro and protein expression in vivo [84]. While it is possible to positively modulate protein expression by modifying codon composition or nucleosides, it is also possible to negatively modulate mRNA secondary structure, translation kinetics and accuracy, simultaneous protein folding kinetics and accuracy, and expression of cryptic T cell epitopes present in alternative reading frames30 [85]. These factors may all affect the magnitude and specificity of the immune response [86]. Figure 3 presents a comprehensive summary of the PERSIST-seq approach, as well as key findings on ribosome load for various mRNA designs. The process begins with a schematic representation of mRNA optimization (Fig. 3-a), where 5′ and 3′ UTRs are combined with Eterna-based and algorithmically generated coding sequences. All mRNA sequences are experimentally evaluated for in-solution and in-cell stability, along with ribosome load, using unique 6–9 nt barcodes for tag counting through short-read sequencing. The experimental layout (Fig. 3-b) demonstrates the parallel assessment of in-solution and in-cell stability and ribosome load, with mRNAs synthesized and prepared in a pooled format before HEK293T cell transfection or in-solution degradation exposure. Polysome traces from a 233-mRNA pool (Fig. 3-c) show the effect of UTR variations on ribosome load, revealing greater variability in average load per construct for 5′ UTR variations (Fig. 3-d). The ribosome load formula is provided alongside box hinges and whiskers illustrating data distribution. Heatmaps (Fig. 3-e) display polysome profiles for the top, middle, and bottom five mRNA designs across design categories, while the SARS-CoV-2 5′ UTR secondary structure model (Fig. 3-f) highlights mutations and substitutions. Finally, heatmaps of SARS-CoV-2 5′ UTR variant polysome profiles (Fig. 3-g) are sorted by ribosome load, offering valuable insight into the impact of design optimization on ribosome efficiency.

### Modulation of immunogenicity

Exogenous mRNA may be recognized by the innate immune system at several levels, including the cell surface, the endosome, and the cytosol [88]; as a result, the innate immune system is extremely immunostimulatory [88]. This characteristic of mRNA might either be helpful or detrimental to therapeutic endeavors, depending on the circumstances [89]. Because it may increase DC maturation, which in turn enhances T and B cell immunological responses, it has the potential to be effective as an adjuvant for vaccination [90]. On the other hand, the reduction of antigen expression could be a collateral consequence of the innate immune system detecting mRNA [91]. In recent years, more clarity has been given to the seemingly contradicting effects of innate immune sensing on distinct mRNA vaccination types [91]; nonetheless, there is still a great deal of work to be done in this area.

Purifying IVT mRNA, adding various nucleosides, and complexing the mRNA with other carrier molecules are all possible ways to modify the immunostimulatory profile of the mRNA, as was discovered in recent study [65]. It is possible for enzymatically generated mRNA samples to include double-stranded RNA contaminants, also known as dsRNA [92]. These contaminants are by-products of the IVT process [65]. Pathogen-associated molecular patterns, or pathogen-associated molecular patterns (PAMPs), such as double-stranded RNA (dsRNA), mimic viral genomes and replication intermediates [93]. Pattern recognition receptors, which are located in a variety of different cellular sites, are responsible for detecting PAMPs [94]. In response to the detection of IVT mRNA that is contaminated with dsRNA, both protein kinase R (also known as EIF2AK2) and 2′-5′-oligoadenylate synthetase (OAS) are activated [95]. This results in the inhibition of translation as well as the destruction of cellular mRNA and ribosomal RNA [96]. Some researchers demonstrated, with the use of chromatographic methods such as reverse-phase fast protein liquid chromatography (FPLC) or high-performance liquid chromatography, that contaminating dsRNA could be efficiently removed from IVT mRNA [97]. It has been proven that purification by FPLC may significantly increase the amount of protein that can be synthesized from IVT mRNA in primary human DCs by as much as a factor of 1,000 [98]. Therefore, it would seem that proper purification of IVT mRNA is necessary for adequate protein (immunogen) production in DCs in order to avoid unnecessary activation of the innate immune system [99]. When exogenous single-stranded mRNA molecules are introduced into cells, they function as a PAMP in a manner similar to that of dsRNA contaminants [100]. Endosomal sensors known as Toll-like receptor 7 (TLR7) and TLR8 are responsible for the generation of type I interferon when they detect single-stranded oligoribonucleotides and the products of their breakdown [101]. Importantly, it was revealed that type I interferon signaling48 may be inhibited by integrating naturally occurring chemically modified nucleosides such as pseudouridine and 1-methylpseudouridine [87]. This was a significant finding. Nucleoside modification is another factor that may, to a certain degree, impede dsRNA species recognition. According to the findings of some researchers, the translation efficiency of nucleoside-modified mRNA is much higher than that of unmodified mRNA both in vitro, particularly in primary DCs, and in vivo in mice [102]. It is important to highlight that DCs were only able to create the highest quantities of protein when the mRNA had been FPLC-purified as well as nucleoside-modified [103]. Recent research into the mechanisms behind innate immune sensing and methods for mitigating the potentially detrimental effects it may have may be responsible, at least in part, for the surge in interest in mRNA-based immunizations and protein replacement therapies [104].

According to the findings of a study that was carried out by some researchers, sequence-optimized, HPLC-purified, unmodified mRNA produced greater amounts of protein in HeLa cells and in mice than its nucleoside-modified counterpart did [105]. In addition, some researchers demonstrated that nucleoside-modified mRNA leads to far less robust protein synthesis than unmodified, non-HPLC-purified mRNA does in HeLa cells, yet both types of mRNA lead to a similar quantity of protein creation in mice [106]. Differences in RNA sequence optimization, the stringency of mRNA purification to exclude dsRNA contaminants, and the degree of innate immune sensing in the targeted cell types may be to blame for the unresolved discrepancies between the findings obtained by some researchers [107]. It is possible that the inclusion of an adjuvant, which increases the immunostimulatory properties of mRNA, might improve the efficiency of some kinds of mRNA vaccination [108]. Some of these approaches make use of traditional adjuvants, while others are considered to be more cutting-edge and leverage on the immunogenicity of mRNA or its potential to encode immune-modulatory proteins [109]. It has been shown that the incorporation of self-replicating RNA vaccines into cationic nanoemulsions, using as their foundation the FDA-approved MF59 (Novartis) adjuvant, results in increased immunogenicity and effectiveness [110]. CD70, CD40 ligand (CD40L), and constitutively active TLR4 are the three immune activator proteins that are combined in the TriMix technique, a powerful adjuvant that mixes the mRNAs that code for them [111]. TriMix mRNA was proven to be more immunogenic than unmodified, unpurified mRNA in several cancer vaccination tests, and this effect was most clearly associated to improved DC maturation and cytotoxic T lymphocyte (CTL) responses [112]. It has been proven that the kind of mRNA carrier utilized and the size of the mRNA-carrier complex both have an effect on the cytokine profile that is produced as a result of the injection of mRNA [113]. For example, the carrier is what provides the adjuvant effect for the RNActive (CureVac AG) immunization platform [113]. RNA complexed with protamine, which is a polycationic peptide, works as an adjuvant by increasing TLR7 signaling and is utilized to make the antigen [114]. The naked, sequence-optimized mRNA is used to produce the antigen [114]. Positive immune responses have been shown in a number of different preclinical animal investigations that used this vaccine formulation to protect against viral diseases as well as cancer [115]. A newly published piece of study has provided mechanistic insight into the adjuvanticity of RNActive vaccines in mice in vivo and human cells in vitro, respectively [116]. The intradermal vaccination led to a high activation of TLR7 in both mice and humans, as well as TLR8 in humans[117]. This immunization also led to the production of type I interferon, as well as pro-inflammatory cytokines and chemokines [118]. In a similar vein, it was shown that RNAdjuvant (CureVac AG), which is an unmodified, single-stranded RNA that is stabilized by a cationic carrier peptide, had adjuvant effect in the context of vaccines that do not include messenger RNA [119].

### Progress in mRNA vaccine delivery

**Fig. 4:**
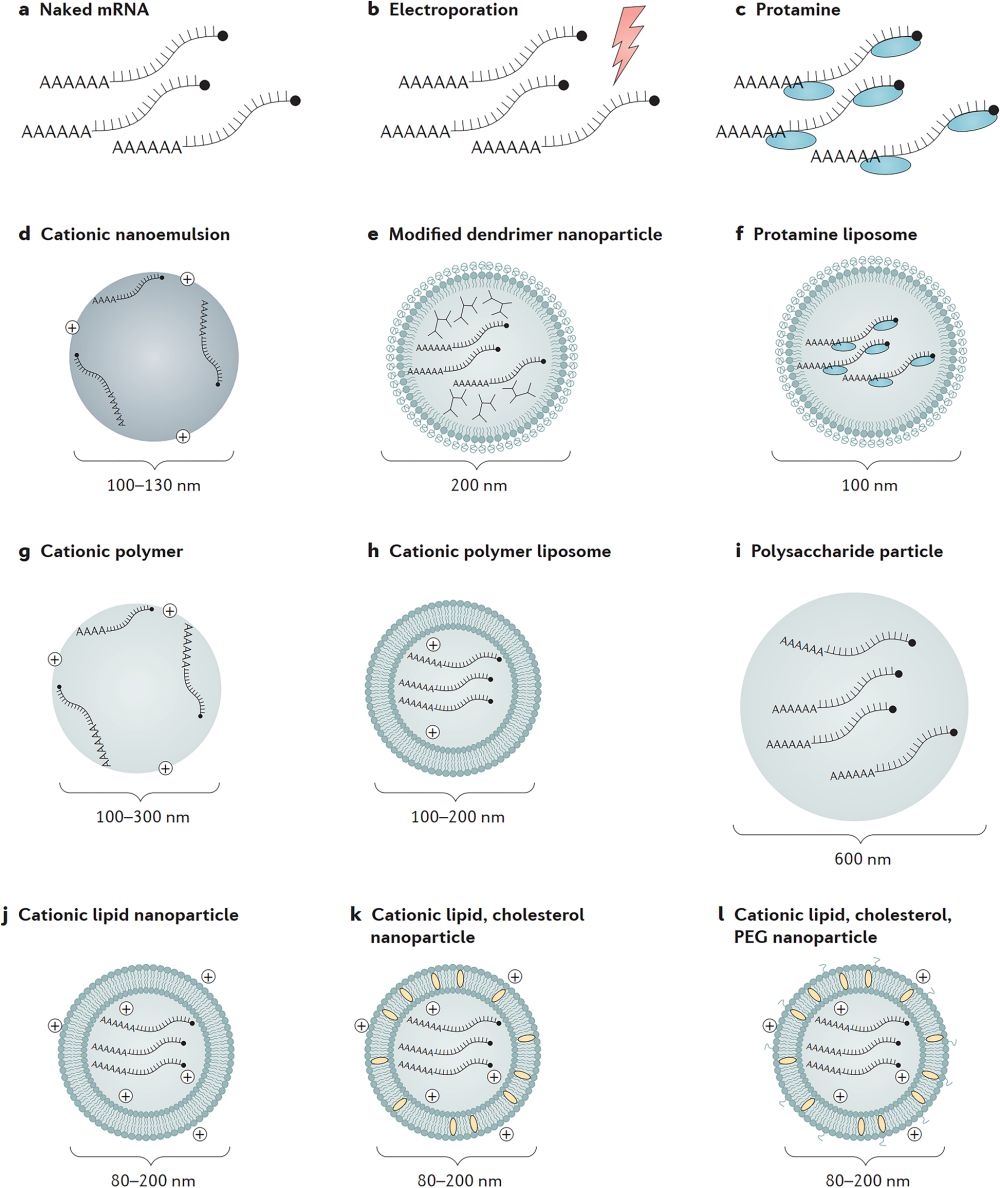
Various delivery methods and carrier molecules for mRNA vaccines, along with the typical diameters of the particulate complexes: **a** Naked mRNA: mRNA without any carrier or delivery system. **b**) Naked mRNA with in vivo electroporation: mRNA is introduced into cells by applying an electric field to facilitate uptake. **c** Protamine-complexed mRNA: mRNA is complexed with protamine, a cationic peptide, to improve stability and cellular uptake. **d** mRNA in cationic nanoemulsion: mRNA is associated with a positively charged oil-in-water cationic nanoemulsion to enhance delivery. **e** mRNA with dendrimer and PEG-lipid: mRNA is associated with a chemically modified dendrimer and complexed with polyethylene glycol (PEG)-lipid for improved delivery and reduced immunogenicity. **f** Protamine-complexed mRNA in a PEG-lipid nanoparticle: mRNA is complexed with protamine and encapsulated in a PEG-lipid nanoparticle for enhanced stability and delivery. **g** mRNA with polyethylenimine (PEI): mRNA is associated with a cationic polymer like PEI to improve delivery and transfection efficiency. **h** mRNA with PEI and lipid component: mRNA is associated with PEI and a lipid component for improved delivery and reduced immunogenicity. **i** mRNA in a polysaccharide particle or gel: mRNA is associated with a polysaccharide, such as chitosan, to form a particle or gel for improved stability and delivery. **j** mRNA in cationic lipid nanoparticle: mRNA is encapsulated in a cationic lipid nanoparticle (e.g., DOTAP or DOPE lipids) for enhanced stability and cellular uptake. **k** mRNA complexed with cationic lipids and cholesterol: mRNA is complexed with cationic lipids and cholesterol for improved stability and delivery. **l** mRNA complexed with cationic lipids, cholesterol, and PEG-lipid: mRNA is complexed with cationic lipids, cholesterol, and PEG-lipid for enhanced stability, delivery, and reduced immunogenicity. Reprinted from [126] with permission from Springer Nature

**Fig. 5:**
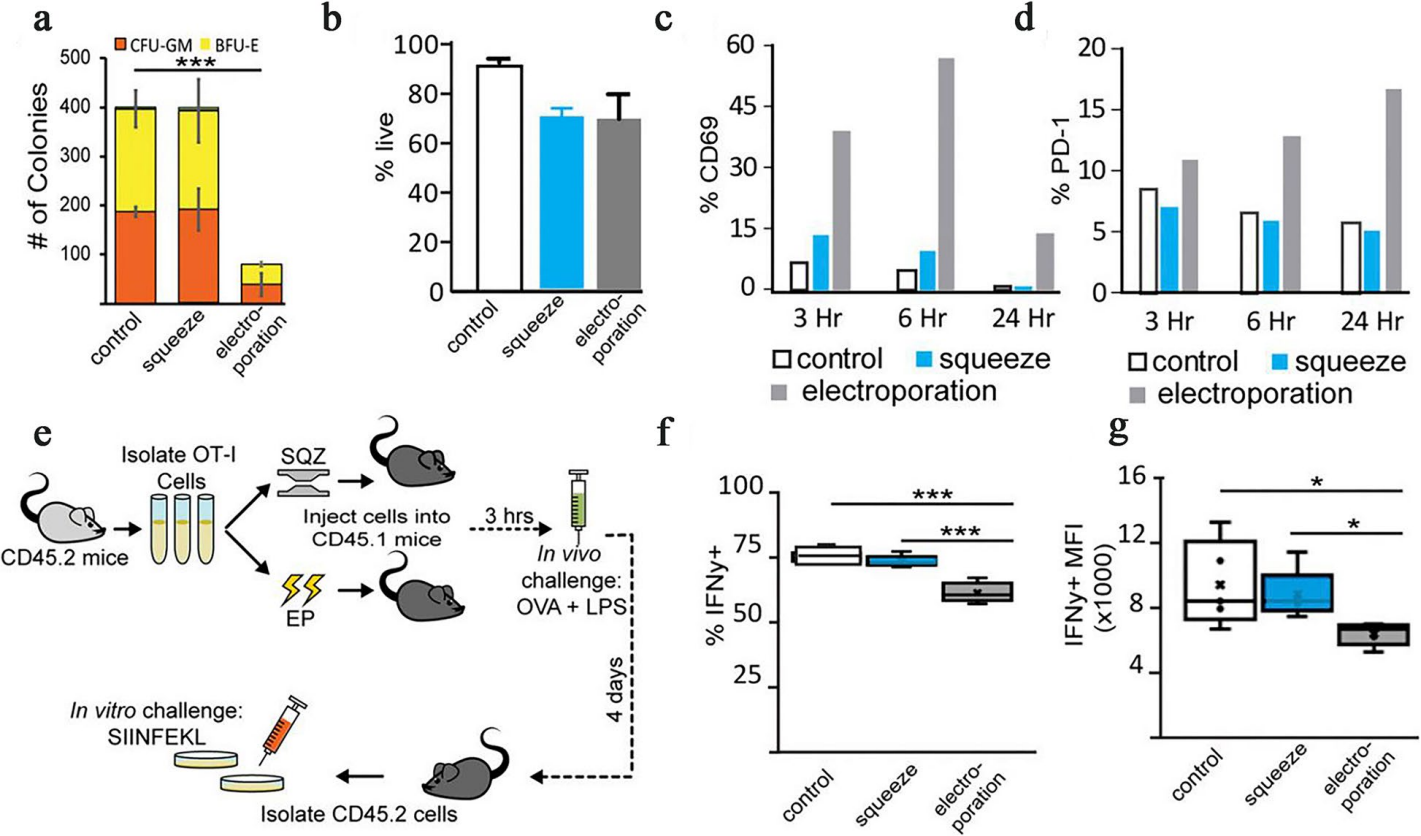
The impact of different intracellular delivery methods on in vitro and in vivo functionality. **a** In vitro colony-forming assays are used to compare the differentiation potential of electroporated and squeezed human CD34 + HSCs into CFU-GM and BFU-E colonies over a 2-week period. **b** The viability of mouse T cells after undergoing squeeze and electroporation is displayed. **c** and **d** The proportion of CD3 + mouse T cells expressing PD-1 or CD69 activation after squeeze, electroporation, or no treatment (control) is presented over time. **e** A diagram illustrates the experimental method for evaluating the effects of delivery methods on T cell activation. **f** and **g** On day 4, after re-exposure to OVA, CD45.2 + /CD8 + /IFN-γ + T cells were stained intracellularly for IFN-γ. Reprinted from [127] with permission from the Proceedings of the National Academy of Sciences

In order to achieve therapeutic relevance, effective mRNA distribution in living organisms is required [120]. It is necessary for the mRNA to pass through the lipid barrier that separates the cytoplasm from the rest of the cell in order for the translation of exogenous mRNA into a functional protein to take place [121]. The mechanisms by which cells appear to take up mRNA appear to differ depending on the lineage of the cells, and the physicochemical properties of mRNA complexes can have a significant impact on the transport of the complexes into cells as well as their subsequent location within tissues [122]. There have, up until this point, been documented two basic strategies for the administration of mRNA vaccines [123]. Direct parenteral injection of mRNA with or without a carrier, followed by first ex vivo loading of mRNA into DCs and then subsequent re-infusion of the transfected cells [55]. Figure 4 illustrates a variety of delivery methods and carrier molecules for mRNA vaccines, each with distinct particulate complex diameters. Naked mRNA (Fig. 4-a) lacks a carrier or delivery system, while in vivo electroporation (Fig. 4-b) uses an electric field to facilitate cellular uptake of naked mRNA. Protamine-complexed mRNA (Fig. 4-c) combines mRNA with the cationic peptide protamine for increased stability and uptake. Cationic nanoemulsion (Fig. 4-d) associates mRNA with a positively charged oil-in-water emulsion, while dendrimer and PEG-lipid complexes (Fig. 4-e) provide improved delivery and reduced immunogenicity. Protamine-complexed mRNA in PEG-lipid nanoparticles (Fig. 4-f) offer enhanced stability and delivery. Polyethylenimine (PEI) (Fig. 4-g) and PEI with lipid component (Fig. 4-h) improve delivery and transfection efficiency. Polysaccharide particles or gels (Fig. 4-i) use materials like chitosan for stability and delivery. Cationic lipid nanoparticles (Fig. 4-j), cationic lipids and cholesterol complexes (Fig. 4-k), and cationic lipids, cholesterol, and PEG-lipid complexes (Fig. 4-l) all serve to optimize stability, delivery, and reduced immunogenicity. Ex vivo DC loading is an expensive and time-consuming way of vaccination that enables precise control of the cellular target, transfection effectiveness, and other cellular properties [124]. Figure 5 demonstrates the impact of different intracellular delivery methods, specifically electroporation and cell squeezing, on the in vitro and in vivo functionality of cells. In an in vitro colony-forming assay (Fig. 5-A), the differentiation potential of human CD34 + hematopoietic stem cells (HSCs) subjected to these methods was assessed by comparing the growth of CFU-GM and BFU-E colonies over a two-week period. Moreover, the viability of mouse T cells following both delivery methods was analyzed (Fig. 5-B). In panels Fig. 5-C and D, the proportion of CD3 + mouse T cells expressing PD-1 or CD69 activation markers after electroporation, cell squeezing, or no treatment (control) was monitored over time. To evaluate the effects of these delivery methods on T cell activation, an experimental method is illustrated in panel Fig. 5-E. On day 4 post-re-exposure to the OVA antigen, CD45.2 + /CD8 + /IFN-γ + T cells were stained intracellularly for IFN-γ, as shown in panels Fig-F and G. Overall, this figure highlights the potential consequences of different intracellular delivery methods on cell functionality and responsiveness. However, this method has the advantage of being able to manage these cellular factors [124]. Even though considerable progress has been made in the field, cell-type-specific delivery that is accurate and efficient cannot yet be achieved with direct injection of mRNA [125]. Table 2 highlights the various characteristics of mRNA cancer vaccine delivery methods. In vivo injection of naked mRNA involves direct injection into the patient, with the antigen being expressed by host cells.

**Table 2:** Characteristics of mRNA cancer vaccine delivery methods

Delivery Method	Description	Mechanism of Action	Advantages	Disadvantages	Safety Concerns	Clinical Development Status	Potential Applications	Reference
In vivo injection of naked mRNA	Direct injection of mRNA into the patient	Expression of the antigen by host cells	Simple and low cost	Low transfection efficiency and immunogenicity	Inflammation at the injection site	Preclinical and early clinical trials	Melanoma, prostate cancer, infectious diseases	[61]
Lipid nanoparticles (LNPs)	mRNA encapsulated in a lipid nanoparticle for delivery	Facilitate cellular uptake and mRNA release	High transfection efficiency and immunogenicity	Potential toxicity and accumulation in liver	Immune response to the lipid components	Clinical trials ongoing	Various cancer types, infectious diseases	[128]
Electroporation	Electrical pulse applied to cells to increase permeability	Enhance mRNA delivery and uptake	High transfection efficiency and immunogenicity	Pain and muscle contractions	Electrode burn and tissue damage	Early clinical trials	Melanoma, breast cancer, head and neck cancer	[129]
Dendritic cell loading	mRNA loaded into dendritic cells (DCs) for antigen presentation	Increase antigen presentation and T cell activation	Efficient and targeted delivery	Complex and costly production process	DC maturation and activation	Preclinical and early clinical trials	Various cancer types	[130]
Polymeric nanoparticles	mRNA encapsulated in a polymeric nanoparticle for delivery	Facilitate cellular uptake and mRNA release	Biodegradable and biocompatible	Lower transfection efficiency than LNPs	Potential toxicity and accumulation in liver	Preclinical trials	Various cancer types, infectious diseases	[131]
Protamine-condensed mRNA	mRNA condensed with protamine for delivery	Facilitate cellular uptake and mRNA release	Efficient and low cost	High toxicity and immunogenicity	Non-specific activation of immune cells	Preclinical trials	Various cancer types, infectious diseases	[132]
mRNA-coated gold nanoparticles	mRNA adsorbed onto gold nanoparticles for delivery	Facilitate cellular uptake and mRNA release	Efficient and targeted delivery	Potential toxicity and accumulation in liver	Gold nanoparticles may activate immune cells	Preclinical trials	Various cancer types, infectious diseases	[133]
In vitro transcribed mRNA-loaded exosomes	mRNA loaded into exosomes for delivery	Facilitate cellular uptake and mRNA release	Targeted delivery and high stability	Lower transfection efficiency than LNPs	Immunogenicity of the exosomes	Preclinical trials	Various cancer types	[66]
Synthetic polymeric vectors	mRNA encapsulated in a synthetic polymeric vector for delivery	Facilitate cellular uptake and mRNA release	Biocompatible and biodegradable	Lower transfection efficiency than LNPs	Potential toxicity and accumulation in liver	Preclinical trials	Various cancer types	[134]
Self-amplifying mRNA vaccines	mRNA encoding self-replicating RNA for delivery	Amplification of mRNA expression in vivo	High immunogenicity and long-term antigen expression	Potential toxicity and long-term safety concerns	Immune response to the viral components	Early clinical trials	Various cancer types	[2]
mRNA-carrying oncolytic viruses	mRNA loaded into oncolytic viruses for delivery	Selective replication in cancer cells and antigen presentation	Tumor-specific delivery and amplification of mRNA expression	Potential toxicity and systemic spread	Immune response to the viral components	Preclinical trials	Various cancer types	[135]
mRNA-nanocomplexes	mRNA encapsulated in a nanoparticle for delivery	Facilitate cellular uptake and mRNA release	Efficient and targeted delivery	Potential toxicity and accumulation in liver	Immune response to the delivery vehicle	Preclinical trials	Various cancer types	[136]
mRNA-loaded hydrogels	mRNA embedded in a hydrogel matrix for delivery	Facilitate cellular uptake and mRNA release	Sustained release and targeted delivery	Lower transfection efficiency than LNPs	Inflammation and fibrosis at the injection site	Preclinical trials	Various cancer types	[2]
mRNA-loaded microneedles	mRNA coated on microneedles for dermal delivery	Facilitate cellular uptake and mRNA release	Simple and painless administration	Limited to dermal applications	Risk of skin irritation and infection	Preclinical trials	Various cancer types, infectious diseases	[137]
mRNA-electrospun fibers	mRNA encapsulated in electrospun fibers for delivery	Facilitate cellular uptake and mRNA release	Sustained release and targeted delivery	Limited to topical applications	Biocompatibility and toxicity concerns	Preclinical trials	Various cancer types, infectious diseases	[138]
mRNA delivery via ultrasound-targeted microbubble destruction	mRNA delivered to the site of interest using ultrasound and microbubbles	Increased cellular uptake and gene expression at the site of interest	Targeted delivery, non-invasive	Limited to superficial tumors, small area of effect	Safety of microbubbles	Preclinical trials	Various cancer types	[139]
mRNA delivery via magnetofection	mRNA complexed with magnetic particles and delivered using a magnetic field	Enhanced cellular uptake and transfection	Targeted delivery, non-invasive	Limited to superficial tumors, small area of effect	Safety of magnetic particles	Preclinical trials	Various cancer types	[140]
mRNA delivery via nanoparticles with tumor-penetrating peptides	mRNA encapsulated in nanoparticles functionalized with tumor-penetrating peptides	Facilitates the penetration of mRNA-containing nanoparticles into tumor tissue	Enhanced tumor-targeting, high transfection efficiency	Limited to solid tumors	Safety of nanoparticles	Preclinical trials	Solid tumors	[141]
mRNA delivery via bacterial vectors	mRNA loaded into attenuated bacteria and delivered to tumor site	Amplifies antigen presentation and tumor-specific immune response	Enhanced immunogenicity, targeted delivery	Potential for bacterial infection and immune response	Safety of bacterial vectors	Preclinical trials	Various cancer types	[142]
mRNA delivery via hyaluronan nanogels	mRNA encapsulated in hyaluronan-based nanogels and delivered to the site of interest	Increased cellular uptake and transfection at the site of interest	Targeted delivery, non-toxic	Limited to superficial tumors, small area of effect	Safety of nanogels	Preclinical trials	Various cancer types	[143]
mRNA delivery via bioresponsive polymeric nanoparticles	mRNA encapsulated in polymeric nanoparticles designed to degrade in response to specific stimuli	Facilitates site-specific mRNA delivery	Targeted delivery, non-toxic	Limited to tumors that can be targeted by specific stimuli	Safety of nanoparticles	Preclinical trials	Various cancer types	[144]
mRNA delivery via tissue engineering scaffolds	mRNA incorporated into tissue engineering scaffolds and delivered to the site of interest	Enhanced cellular uptake and transfection at the site of interest	Site-specific delivery, potentially long-term antigen expression	Limited to solid tumors and tissue-engineered sites	Safety of scaffolds	Preclinical trials	Various cancer types, tissue engineering	[145]
mRNA delivery via extracellular vesicles	mRNA encapsulated in extracellular vesicles derived from a patient's own cells and delivered to the site of interest	Enhanced tumor-targeting, high transfection efficiency	Targeted delivery, non-toxic	Limited to solid tumors	Safety of extracellular vesicles	Preclinical trials	Solid tumors	[146]
mRNA delivery via in vivo electroporation	mRNA delivered to tissue via electroporation	Facilitates cellular uptake and gene expression	Targeted delivery, non-toxic	Limited to specific tissue types and requires specialized equipment	Risk of tissue damage from electroporation	Preclinical trials, some clinical trials	Various cancer types	[147]
mRNA delivery via dissolvable microneedle arrays	mRNA coated onto dissolvable microneedles and applied to skin	Facilitates cellular uptake and gene expression in skin	Targeted delivery, non-invasive	Limited to skin and superficial tumors	Safety of microneedles	Preclinical trials	Skin cancers	[148]
mRNA delivery via laser ablation	mRNA delivered to tissue via laser ablation	Facilitates cellular uptake and gene expression	Targeted delivery, non-toxic	Limited to specific tissue types and requires specialized equipment	Risk of tissue damage from laser ablation	Preclinical trials	Various cancer types	[149]
mRNA delivery via microfluidic chips	mRNA delivered to cells via microfluidic chips	Facilitates cellular uptake and gene expression	Targeted delivery, precise control over flow rates and concentrations	Limited to specific cell types and requires specialized equipment	Safety of microfluidic chips	Preclinical trials	Various cancer types	[150]
mRNA delivery via electrospray	mRNA delivered to tissue via electrospray	Facilitates cellular uptake and gene expression	Targeted delivery, non-toxic	Limited to specific tissue types and requires specialized equipment	Safety of electrospray	Preclinical trials	Various cancer types	[151]
mRNA delivery via cell-penetrating peptides	mRNA complexed with cell-penetrating peptides and delivered to cells	Facilitates cellular uptake and gene expression	Targeted delivery, non-toxic	Limited to specific cell types	Safety of cell-penetrating peptides	Preclinical trials	Various cancer types	[152]
mRNA delivery via gene gun	mRNA coated onto gold particles and delivered to tissue via gene gun	Facilitates cellular uptake and gene expression	Targeted delivery, non-toxic	Limited to specific tissue types and requires specialized equipment	Risk of tissue damage from gene gun	Preclinical trials	Various cancer types	[153]
mRNA delivery via polymeric carriers	mRNA complexed with biodegradable polymeric carriers and delivered to tissue	Facilitates cellular uptake and gene expression	Targeted delivery, non-toxic, controlled release	Limited to specific tissue types and requires specialized equipment	Safety of polymeric carriers	Preclinical trials	Various cancer types	[154]
mRNA delivery via lipoplexes	mRNA complexed with lipids and delivered to cells	Facilitates cellular uptake and gene expression	Non-toxic, efficient	Limited to specific cell types	Safety of lipids	Preclinical trials	Various cancer types	[155]
mRNA delivery via dendrimers	mRNA complexed with dendrimers and delivered to cells	Facilitates cellular uptake and gene expression	Non-toxic, efficient	Limited to specific cell types	Safety of dendrimers	Preclinical trials	Various cancer types	[156]
mRNA delivery via gold nanoparticles	mRNA complexed with gold nanoparticles and delivered to cells	Facilitates cellular uptake and gene expression	Non-toxic, efficient	Limited to specific cell types	Safety of gold nanoparticles	Preclinical trials	Various cancer types	[133]
mRNA delivery via viral vectors	mRNA loaded into viral vectors and delivered to cells	Facilitates cellular uptake and gene expression	High transfection efficiency, targeted delivery	Risk of immune response and viral integration	Safety of viral vectors	Preclinical trials	Various cancer types	[157]
mRNA delivery via cell-based vehicles	mRNA loaded into various cell types and delivered to target tissues	Facilitates cellular uptake and gene expression	Non-toxic, potential for targeting and controlled release	Limited to specific cell types and requires specialized equipment	Safety of cell-based vehicles	Preclinical trials	Various cancer types	[158]
mRNA delivery via exosomes	mRNA encapsulated in exosomes and delivered to target tissues	Facilitates cellular uptake and gene expression	Non-toxic, potential for targeting and controlled release	Limited to specific tissues	Safety of exosomes	Preclinical trials	Various cancer types	[159]
mRNA delivery via ribonucleoprotein complexes	mRNA complexed with ribonucleoproteins and delivered to cells	Facilitates cellular uptake and gene expression	Non-toxic, efficient	Limited to specific cell types	Safety of ribonucleoproteins	Preclinical trials	Various cancer types	[147]
mRNA delivery via inorganic nanoparticles	mRNA complexed with inorganic nanoparticles and delivered to cells	Facilitates cellular uptake and gene expression	Non-toxic, efficient, targeted delivery	Limited to specific cell types	Safety of inorganic nanoparticles	Preclinical trials	Various cancer types	[147]
mRNA delivery via sonoporation	mRNA delivered to tissue via ultrasound-mediated sonoporation	Facilitates cellular uptake and gene expression	Non-invasive, targeted delivery	Limited to specific tissue types and requires specialized equipment	Risk of tissue damage from sonoporation	Preclinical trials	Various cancer types	[160]
mRNA delivery via gas-filled microbubbles	mRNA delivered to tissue via microbubble-assisted ultrasound	Facilitates cellular uptake and gene expression	Non-invasive, targeted delivery	Limited to specific tissue types and requires specialized equipment	Safety of microbubbles	Preclinical trials	Various cancer types	[161]
mRNA delivery via electrical fields	mRNA delivered to cells via electrical fields	Facilitates cellular uptake and gene expression	Non-toxic, efficient	Limited to specific cell types and requires specialized equipment	Safety of electrical fields	Preclinical trials	Various cancer types	[162]
mRNA delivery via bacterial vectors	mRNA loaded into bacterial vectors and delivered to cells	Facilitates cellular uptake and gene expression	Targeted delivery, high transfection efficiency	Risk of immune response and bacterial infection	Safety of bacterial vectors	Preclinical trials	Various cancer types	[147]
mRNA delivery via CRISPR-Cas systems	mRNA encoding CRISPR-Cas system delivered to cells	Facilitates targeted gene editing	Precise, efficient	Limited to specific cell types and requires specialized equipment	Safety of CRISPR-Cas system	Preclinical trials	Various cancer types	[163]
mRNA delivery via cell-penetrating antibodies	mRNA complexed with cell-penetrating antibodies and delivered to cells	Facilitates cellular uptake and gene expression	Non-toxic, potential for targeting and controlled release	Limited to specific cell types	Safety of cell-penetrating antibodies	Preclinical trials	Various cancer types	[164]
mRNA delivery via non-viral vectors	mRNA complexed with non-viral vectors and delivered to cells	Facilitates cellular uptake and gene expression	Non-toxic, efficient, potential for targeted delivery	Limited to specific cell types	Safety of non-viral vectors	Preclinical trials	Various cancer types	[165]

#### Ex vivo loading of DCs

DCs are unparalleled in their ability to deliver antigens to T cells [166]. Adaptive immunity is initiated when APCs take in and proteolytically digest antigens, then present them on major histocompatibility complexes (MHCs) of the class I and class II kind to helper T cells (CD8 + and CD4 + T cells) [167]. The DCs' ability to transmit intact antigen to B cells and so stimulate an antibody response is another important function [168]. DCs also respond well to mRNA transfection [168].

Considering these characteristics, DCs are a potentially effective in vivo and ex vivo target for mRNA vaccine transfection [169]. While it has been established that DCs ingest naked mRNA through a number of endocytic mechanisms, electroporation is routinely employed to boost transfection effectiveness ex vivo by forcing mRNA molecules through membrane pores generated by a high-voltage pulse and into the cytoplasm [170]. This method of mRNA administration has become widely used due to its high transfection effectiveness and lack of a carrier molecule [171]. DCs are re-infused into a patient undergoing autologous immunization after being pre-activated with mRNA in vitro [172]. Because they induce a cell-mediated immune response, most DC vaccines that have been loaded ex vivo have been used to treat cancer [172].

#### Injection of naked mRNA in vivo

In vivo vaccines using naked mRNA have been shown to be effective, particularly when administered by intradermal or intranodal injections, both of which preferentially target antigen-presenting cells [173]. Recent study has revealed that immunizing patient’s numerous times with unmodified mRNA encoding tumor-associated neoantigens boosts progression-free survival and generates robust T cell responses [174]. Some researchers were the first to use tailored cancer vaccines including neoepitope mRNA [175].

High-throughput sequencing is used to identify each somatic mutation in a patient's tumor sample [176]. The term "mutanome" is used to describe this phenomenon [177]. In addition to allowing for the rational construction of neoepitope cancer vaccines on an individual basis, this approach has the added benefit of focusing on non-self-antigen specificities that central tolerance mechanisms shouldn't destroy [178]. Recent advances have established proof of concept in the following fields: Scientists found that a sizable fraction of non-synonymous cancer mutations were immunogenic when delivered by messenger RNA, and that CD4 + T cells were the preeminent population capable of recognizing these abnormalities [179]. Using this information, they devised a computational method for predicting vaccine immunogens that are confined to major MHC class II [180].

Tumor growth was inhibited in animal models of B16-F10 melanoma and CT26 colon cancer when mRNA vaccines encoding these neoepitopes were administered [58]. Some researchers recently conducted a clinical trial in which 13 patients with metastatic melanoma were given customized neoepitope-based mRNA vaccinations [30]. The high rate of somatic mutations and subsequent neoepitopes in melanoma makes it a distinct subtype of the malignancy [181]. They immunized people against 10 different neoepitopes by injecting naked mRNA into their noses [182]. After several months of follow-up, a low incidence of metastatic disease was seen, and CD4 + T cell responses were found against the bulk of the neoepitopes [183]. It is worth noting that a study with a similar methodology, but using synthetic peptides as the immunogens instead of mRNA, also yielded similar results [183]. All of these recent clinical trials support the idea that the personalized vaccine technique may have some application [184].

#### Physical delivery methods in vivo

On occasion, the breaching of the cell membrane and the enhancement of the efficient uptake of mRNA in vivo have been accomplished through the use of physical methods [185]. In order to express mRNA in tissues that is complexed with gold particles, microprojectile technology, also known as a "gene gun," has been utilized [186]. The gene gun has been shown to be an efficient method of RNA delivery and immunization in mouse models; however, there is a dearth of data about the gene gun's usefulness in either large animals or people at this time [187]. The immunogenicity of a vaccination that was based on non-replicating mRNA was not improved by in vivo electroporation [188]; nevertheless, one research found that the absorption of therapeutic RNA was improved [188]. When adopting physical methods, there is a possibility of increased cell death and decreased access to the cells or tissues of interest [189]. However, lipid or polymer-based nanoparticles have recently acquired favor as effective and adaptable delivery vehicles [190]. This trend is expected to continue in the near future.

#### Protamine

In spite of the fact that protamine, a cationic peptide, has been demonstrated to protect mRNA from degradation by serum RNases, protamine-complexed mRNA alone demonstrated limited protein expression and efficacy in a cancer vaccine model [191]. This could have been the result of an overly tight association between protamine and mRNA [191]. This issue was the impetus behind the development of the RNActive vaccination platform, which employs RNA that has been modified with protamine purely for the purpose of acting as an immune activator and not as an expression vector [192].

#### Cationic lipid and polymer-based delivery

Although there are commercially available highly efficient mRNA transfection reagents that are based on cationic lipids or polymers and work effectively in a large number of primary cells and cancer cell lines, these reagents frequently exhibit either limited efficacy in vivo or a high level of toxicity [56]. TransIT-mRNA (manufactured by Mirus Bio LLC) and Lipofectamine are two examples (Invitrogen) [193]. In a number of recent studies, the tremendous progress that has been made in the development of complexing reagents that are similarly designed for use in vivo that is both safe and successful has been discussed [194]. In recent years, dendrimers and other cationic lipids and polymers have emerged as preferred techniques for the delivery of mRNA [133]. For almost a decade, researchers have used small interfering RNA (siRNA) as a delivery vehicle in the mRNA region, and their efforts have undoubtedly paid off [195].mRNA distribution using lipid nanoparticles is quickly becoming one of the most promising and widely used technologies of LNPs [196]. LNPs are composed of four primary components [197]: an ionizable cationic lipid that promotes self-assembly into virus-sized (100 nm) particles and allows endosomal release of mRNA to the cytoplasm [197]; lipid-linked polyethylene glycol (PEG) that increases the half-life of formulations [198]; cholesterol, which acts as a stabilizing agent; and naturally occurring phospholipids that support lipid bilayer structure [199]. All of these components work together Although LNPs have been found to be effective instruments for the in vivo administration of siRNAs in a number of studies, it was not until recently that it was discovered that they may also be used to deliver larger RNAs as well as traditional, non-replicating mRNA21 [199]. This discovery was made despite the fact that LNPs have been found to be effective instruments for the in vivo administration of siRNA. Although it has been demonstrated that intradermal, intramuscular, and subcutaneous administration can produce prolonged protein expression at the site of injection, systemically delivered mRNA-LNP complexes primarily target the liver due to the binding of apolipoprotein E and the subsequent receptor-mediated uptake by hepatocytes [200]. This is the case even though these administration routes have been shown to produce prolonged protein expression at the site of injection. Neither artificial liposomes nor exosomes that occur naturally have had their processes for mRNA escape into the cytoplasm completely deciphered [156]. More research into this subject area is likely to be very beneficial to the field of therapeutic RNA delivery.

One strategy to vary the amount of in vivo protein synthesis as well as its duration is by changing the route through which mRNA-LNP vaccines are delivered to the body [201]. In the experiment, the half-life of mRNA-encoded firefly luciferase was roughly threefold longer after intradermal injection than after intravenous delivery, demonstrating that intramuscular and intradermal delivery of mRNA-LNPs result in more persistent protein expression than systemic delivery routes [202]. It is likely that the rapidity with which mRNA-LNPs are generated can be advantageous for inducing immune responses [202]. Recent research has found that high levels of antibody titers, as well as B cells from the germinal center (GC) and TFH cells, are driven by prolonged antigen availability during vaccination [203]. This process may have contributed to the efficiency of intramuscular and intradermally given nucleoside-modified mRNA-LNP vaccines [203]. Vaccines have been proven to be successful only if they stimulate a specific population of immune cells termed TFH cells, which are necessary for eliciting powerful and lasting neutralizing antibody responses, especially against viruses that escape humoral immunity [204]. Any advancement in our knowledge of the kinetics of the GC reaction and the differentiation of the TFH cell will undoubtedly assist the design of future vaccines.

## mRNA cancer vaccines

**Fig. 6:**
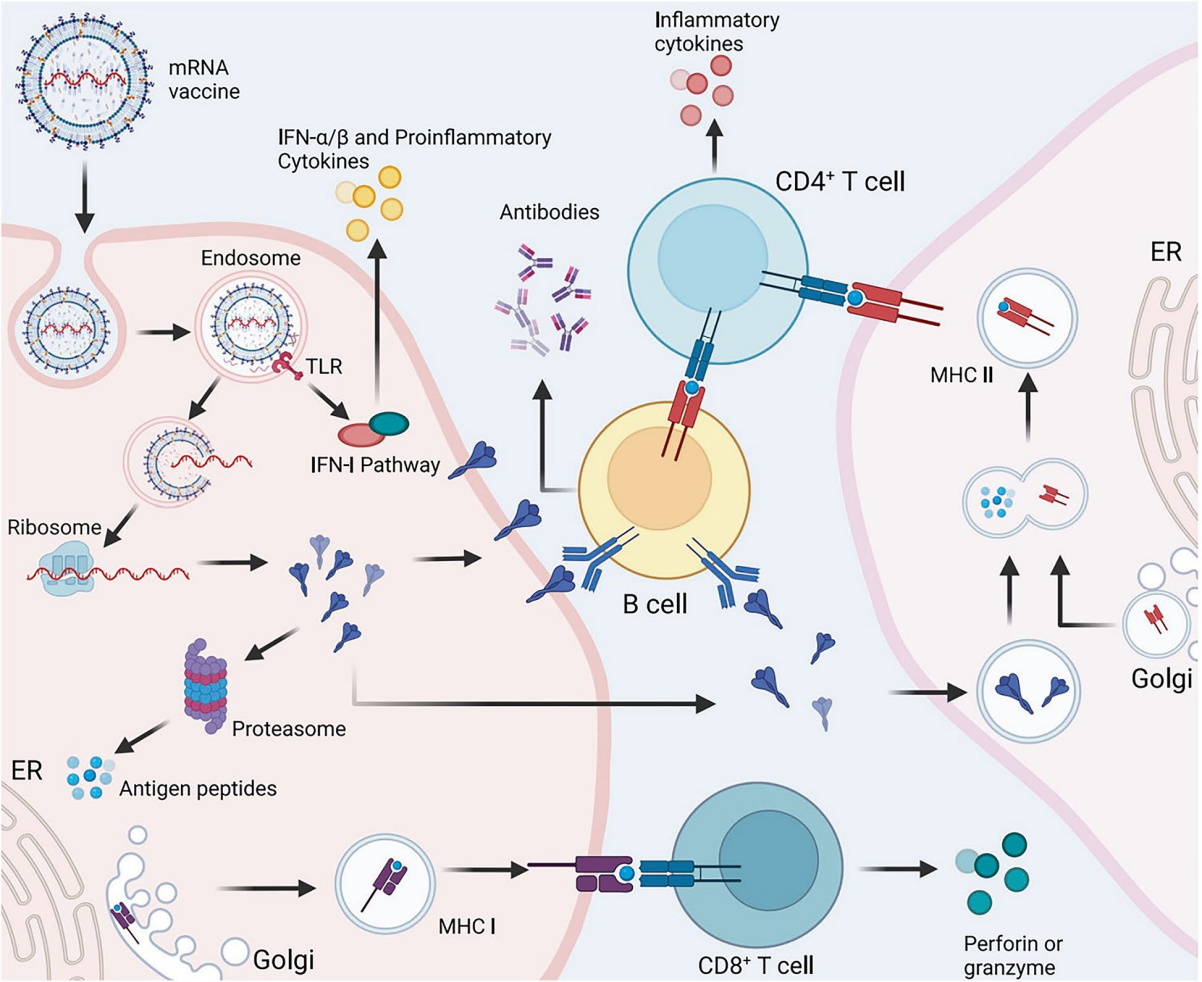
In an mRNA vaccine, the mRNA is taken up by cells through endocytosis and subsequently released from the endosome to be converted into proteins by ribosomes. These proteins can activate the immune system in two primary ways: i) the proteins are broken down by proteasomes into peptides that are then displayed as antigens on the cell surface by MHC class I molecules, which bind to the TCR and activate CD8 + T cells to destroy infected cells by releasing perforin and granzyme; ii) proteins secreted externally are taken up by APCs and broken down into peptides that are then displayed on the cell surface by MHC class II molecules for recognition by CD4 + T cells, which can activate both cellular immune responses by producing cytokines and humoral immune responses by co-activating B cells. Additionally, single-stranded RNA and double-stranded RNA in mRNA vaccines bind to TLR in the endosome to activate antiviral innate immune responses through the production of type-I interferon (IFN-I), which leads to the induction of numerous IFN-1-stimulated genes involved in antiviral innate immunity, a process known as the self-adjuvant effect of sequence-engineered mRNA. Reprinted from [205] with permission from Springer Nature

Cancer vaccines based on messenger RNA have been the subject of recent, in-depth last investigations [2]. Figure 6 illustrates the process of how mRNA vaccines work to activate the immune system. When the mRNA vaccine is introduced into the body, it is taken up by cells through endocytosis and released from the endosome. Ribosomes then convert the mRNA into proteins that stimulate the immune system in two main ways: i) proteasomes break down the proteins into peptides, which are displayed as antigens on the cell surface by MHC class I molecules, subsequently activating CD8 + T cells that release perforin and granzyme to destroy infected cells; ii) proteins secreted externally are absorbed by APCs and broken down into peptides, which are displayed on the cell surface by MHC class II molecules, allowing recognition by CD4 + T cells, which in turn activate cellular immune responses by producing cytokines and humoral immune responses by co-activating B cells. Furthermore, single-stranded RNA and double-stranded RNA in mRNA vaccines bind to TLR in the endosome, initiating antiviral innate immune responses through the production of type-I interferon (IFN-I). This leads to the induction of numerous IFN-I-stimulated genes involved in antiviral innate immunity, a phenomenon referred to as the self-adjuvant effect of sequence-engineered mRNA.

Cancer vaccines and other forms of immunotherapy represent promising new approaches in the war against the disease [206]. Tumor-associated antigens, such as growth-associated factors or antigens that are unique to malignant cells as a result of somatic mutation, can be used in the development of cancer vaccines [207]. Targeting either these neoantigens or the neoepitopes they are composed of, human mRNA vaccines have been created [58]. Most cancer vaccines are designed for therapeutic use rather than prevention [2]; they function by eliciting cell-mediated responses (such as CTLs) that can remove or greatly reduce tumor burden [208]. The earliest proof-of-concept studies proposing and presenting evidence for the feasibility of RNA cancer vaccines were published more than two decades ago [209]. Numerous studies on both animals and humans have since confirmed that mRNA vaccinations are highly effective against cancer [2].

### Tumor cell-based vaccines

Tumor cell-based vaccines are a type of cancer vaccine that involves using whole tumor cells to stimulate an immune response against cancer [210]. These vaccines are designed to target the unique antigens expressed by tumor cells, which can elicit an immune response specifically directed against the cancer cells [211].

Autologous Tumor Cell-Based Vaccines: Autologous tumor cell-based vaccines are personalized vaccines created from the patient's own tumor cells [210]. A small sample of the patient's tumor is obtained, and tumor cells are isolated and processed in the laboratory [178]. These tumor cells may be modified or treated to enhance their immunogenicity and ability to trigger an immune response [210]. For example, they can be genetically engineered to express molecules that stimulate the immune system or fused with immune-stimulating substances [212]. The modified tumor cells are then reintroduced into the patient through vaccination. The idea behind autologous tumor cell-based vaccines is to create a vaccine that contains a broad spectrum of antigens unique to the patient's tumor [213]. By using the patient's own tumor cells, the vaccine aims to activate the patient's immune system against the specific antigens present in their cancer cells, potentially leading to a targeted immune response against the tumor [214].

Allogeneic tumor cell-based vaccines are developed using tumor cells obtained from a donor or cell lines derived from tumor tissues. These cells are not specific to the patient receiving the vaccine [213]. Allogeneic vaccines may contain a mixture of tumor cell lines from different patients or may be derived from well-characterized tumor cell lines established in the laboratory. Allogeneic vaccines offer the advantage of being readily available, as they can be produced in large quantities and stored for future use [215]. They can also provide a broader range of tumor antigens compared to autologous vaccines since they may represent a variety of tumor types and genetic variations [212]. However, there is a risk of immune rejection or immune tolerance to the allogeneic tumor cells, which may limit their effectiveness [215]. Both autologous and allogeneic tumor cell-based vaccines are being investigated in clinical trials and research studies [213]. These vaccines represent an approach that harnesses the patient's immune system to recognize and attack the unique antigens present on their tumor cells [212]. By stimulating an immune response against these antigens, tumor cell-based vaccines aim to target and destroy cancer cells while sparing healthy cells [215].

### Peptide-based vaccines

Peptide-based vaccines are a type of cancer vaccine that utilizes small protein fragments called peptides to trigger an immune response against cancer cells [210]. These peptides are derived from tumor-specific antigens, which are unique proteins expressed by cancer cells [216]. The process of developing a peptide-based vaccine begins with identifying specific antigens that are associated with the tumor cells of interest. These antigens can be identified through various methods, such as analyzing the proteins expressed by cancer cells or studying the immune response of cancer patients [217]. Once the tumor-specific antigens are identified, the corresponding peptides are synthesized or produced using recombinant DNA technology. These peptides are designed to mimic the antigens and contain specific regions that can stimulate an immune response [216].

Peptide-based vaccines are typically administered through injection, either subcutaneously or intramuscularly. Upon injection, the peptides are presented to immune cells called antigen-presenting cells (APCs) such as dendritic cells. APCs engulf the peptides and process them internally [218]. The processed peptides are then presented on the surface of APCs in complex with major histocompatibility complex (MHC) molecules, forming MHC-peptide complexes [216]. The MHC-peptide complexes on the surface of APCs act as signals to alert and activate immune cells, especially CTLs or CD8 + T cells, which are crucial for eliminating cancer cells [210].

CTLs recognize the MHC-peptide complexes as foreign or abnormal, indicating the presence of cancer cells displaying those specific antigens. This triggers the activation of CTLs, leading to their proliferation and the release of immune molecules such as cytokines and perforins [217]. The activated CTLs can then migrate to tumor sites, recognize cancer cells expressing the targeted antigens, and eliminate them through various mechanisms, including inducing cell death (apoptosis) or activating other components of the immune system to attack the cancer cells [212].

Peptide-based vaccines offer several advantages. They are highly specific because they target tumor-specific antigens, minimizing the risk of off-target effects. Peptides can be synthesized relatively easily and can be modified to enhance their stability and immunogenicity [210]. Additionally, peptide-based vaccines are generally well-tolerated and have a favorable safety profile [216]. However, there are challenges associated with peptide-based vaccines [219]. Peptides alone may not efficiently stimulate a strong immune response, so adjuvants or immune-stimulating molecules are often included to enhance the vaccine's efficacy [216]. Another challenge is the potential for tumor cells to escape immune recognition by downregulating the expression of targeted antigens or undergoing genetic mutations that alter the antigenic profile [220]. Ongoing research and advancements in peptide synthesis, adjuvant design, and personalized medicine are continuously improving the effectiveness of peptide-based vaccines. They are being investigated as both standalone therapies and in combination with other immunotherapeutic approaches to enhance immune responses against cancer cells and improve patient outcomes [221].

### Viral vector-based vaccines

Viral vector-based vaccines are a type of vaccine that uses modified viruses as carriers or vectors to deliver specific antigens into the body [222]. These viruses are typically genetically engineered to be safe and non-replicative, meaning they cannot cause disease in the vaccinated individual. Instead, they serve as delivery vehicles to introduce selected antigens to the immune system and stimulate an immune response [222].

The viral vectors used in these vaccines are often derived from naturally occurring viruses, such as adenoviruses or lentiviruses. These viruses have the ability to infect cells and deliver their genetic material [223]. However, in viral vector-based vaccines, the viral genes responsible for replication and causing disease are removed or inactivated, ensuring that the vector cannot replicate in the body and cause harm [223]. To create a viral vector-based vaccine, scientists modify the viral vector by inserting a gene that encodes the desired antigen. This antigen is typically a protein or a part of a pathogen, such as a viral protein or a tumor-specific antigen [224]. Once the modified viral vector is administered to a person, it enters cells and releases the genetic material encoding the antigen. The cells then use this genetic information to produce the antigen, which is presented to the immune system [224].

The immune system recognizes the antigen as foreign and mounts an immune response against it. This response includes the activation of immune cells, such as T cells and B cells, which are essential for eliminating the targeted antigen [225]. The immune system also generates memory cells that remember the antigen, providing long-term protection against future encounters with the actual pathogen or cancer cells expressing the specific antigen.

Viral vector-based vaccines offer several advantages [226]. They have the ability to deliver genetic material encoding complex antigens, making them suitable for generating robust immune responses [223]. These vaccines can also be designed to target specific cell types or tissues, further enhancing their effectiveness [222]. Additionally, viral vectors can stimulate both cellular and humoral immune responses, involving different components of the immune system [223]. Viral vector-based vaccines have been successfully utilized in various vaccine development efforts, including the development of COVID-19 vaccines [226]. These vaccines have shown promising results in stimulating immune responses and providing protection against targeted pathogens or tumor cells. Ongoing research continues to explore and optimize viral vector-based vaccines for a wide range of diseases, including cancer, infectious diseases, and genetic disorders [223].

### Nucleic acid-based vaccines

Nucleic acid-based vaccines represent a promising approach in the field of immunization, offering a unique strategy to induce protective immune responses against various diseases, including cancer [227]. These vaccines utilize genetic material, either in the form of DNA or RNA, to encode specific antigens that are targeted for immune recognition [228]. Nucleic acid-based vaccines have garnered significant attention due to their ability to mimic natural infection processes and stimulate robust and long-lasting immune responses [229].

DNA-based vaccines are designed by inserting the gene encoding the desired antigen into a circular DNA plasmid. This plasmid is then administered directly into the body through intramuscular or intradermal injection [230]. Once inside the cells, the DNA plasmid is taken up by the nucleus, where the antigen gene is transcribed into mRNA. The mRNA then undergoes translation in the cytoplasm, leading to the synthesis of the target antigen within the host cells. This process mimics the natural viral infection cycle, but without causing disease [231]. The newly produced antigen is subsequently presented on the surface of the host cells, triggering an immune response that includes the activation of APCs, such as dendritic cells [232]. These APCs process and present the antigen to T cells, thereby initiating a specific immune response against the antigen-expressing cells.

RNA-based vaccines, on the other hand, directly utilize mRNA molecules encoding the desired antigens [231]. These mRNA vaccines can be synthesized in the laboratory and then encapsulated within lipid nanoparticles for efficient delivery into the cells. Upon administration, the lipid nanoparticles protect the mRNA from degradation and facilitate its entry into host cells [227]. Once inside the cells, the mRNA is translated into the target antigen, triggering an immune response similar to DNA-based vaccines [228]. RNA-based vaccines offer several advantages, such as ease of design and production, rapid manufacturing process, and flexibility to incorporate modifications to enhance antigen expression or stability [231]. Both DNA-based and RNA-based vaccines have shown great potential in cancer immunotherapy. They enable the expression of tumor-specific antigens within the patient's own cells, leading to the presentation of these antigens to the immune system [230]. This process activates immune cells, including cytotoxic T cells, NK cells, and B cells, which work together to target and destroy cancer cells expressing the specific antigen [228]. In addition to directly targeting tumor cells, nucleic acid-based vaccines can also stimulate an immune response against other components of the tumor microenvironment, such as stromal cells or immune-suppressive cells, thereby promoting a comprehensive anti-tumor response [231]. Moreover, nucleic acid-based vaccines offer several advantages over traditional vaccine approaches. Firstly, they have the potential to elicit both humoral (antibody-mediated) and cellular (T cell-mediated) immune responses, making them suitable for combating various pathogens and diseases, including those that require both arms of the immune system for protection [227]. Secondly, nucleic acid-based vaccines can be rapidly developed and manufactured, as they do not rely on the time-consuming process of growing live attenuated pathogens or producing recombinant proteins. This characteristic makes them particularly valuable in the context of emerging infectious diseases or rapidly evolving pathogens [230]. Thirdly, nucleic acid-based vaccines are highly flexible and adaptable, allowing for the incorporation of multiple antigens or modifications to optimize their efficacy. This flexibility is particularly advantageous in the case of cancer vaccines, where targeting multiple tumor-specific antigens or incorporating immune-stimulating adjuvants can enhance therapeutic outcomes [223].

### DC mRNA cancer vaccines

The use of DCs in cancer immunotherapy made sense because of their pivotal function in initiating antigen-specific immune responses [233]. Some researchers described how DCs electroporated with mRNA might trigger strong immune responses against tumor antigens [234]. Stimulating DCs with ovalbumin (OVA)-encoding mRNA or tumor-derived RNAs dampened the immune response in OVA-expressing and other mice models of melanoma [235].

Immune regulatory proteins can be found in abundance in mRNA-encoded adjuvants, which can increase the potency of DC cancer vaccines [236]. Multiple studies have revealed that electroporation of DCs with mRNAs expressing co-stimulatory molecules like CD83, tumour necrosis factor receptor superfamily member 4, greatly increases their immune stimulating activity [237]. However, DC activity can also be modulated by a variety of substances linked with trafficking and by pro-inflammatory cytokines encoded on messenger RNA [237].

Electroporation of antigen-encoding mRNA or mRNAs is possible in conjunction with the adjuvant cocktail TriMix, which includes mRNA-encoded CD70, CD40L, and constitutively active TLR4 [111]. This formulation has been shown to be effective in a number of preclinical studies by increasing DC activation and altering the phenotype of CD4 + T cells to make them more like TH1 cells rather than T regulatory cells [238]. A total of 27% of patients with stages III or IV melanoma who were treated with DCs loaded with mRNA expressing melanoma-associated antigens and TriMix adjuvant experienced tumor regression [239].

Trials using DC immunization have been conducted for a variety of cancers, including prostate, lung, brain, and pancreatic cancers [239]. It is a new approach to combine mRNA electroporation of DCs with standard chemotherapeutic medicines or immune checkpoint inhibitors [240]. Patients in stages III and IV of the disease were given a combination of the monoclonal antibody ipilimumab, which targets CTL antigen 4, and DCs loaded with mRNA encoding melanoma-associated antigens and TriMix [239]. After receiving this treatment, the majority of patients with recurrent or refractory melanoma reported a substantial reduction in tumour size [239].

### Direct injection of mRNA cancer vaccines

**Fig. 7:**
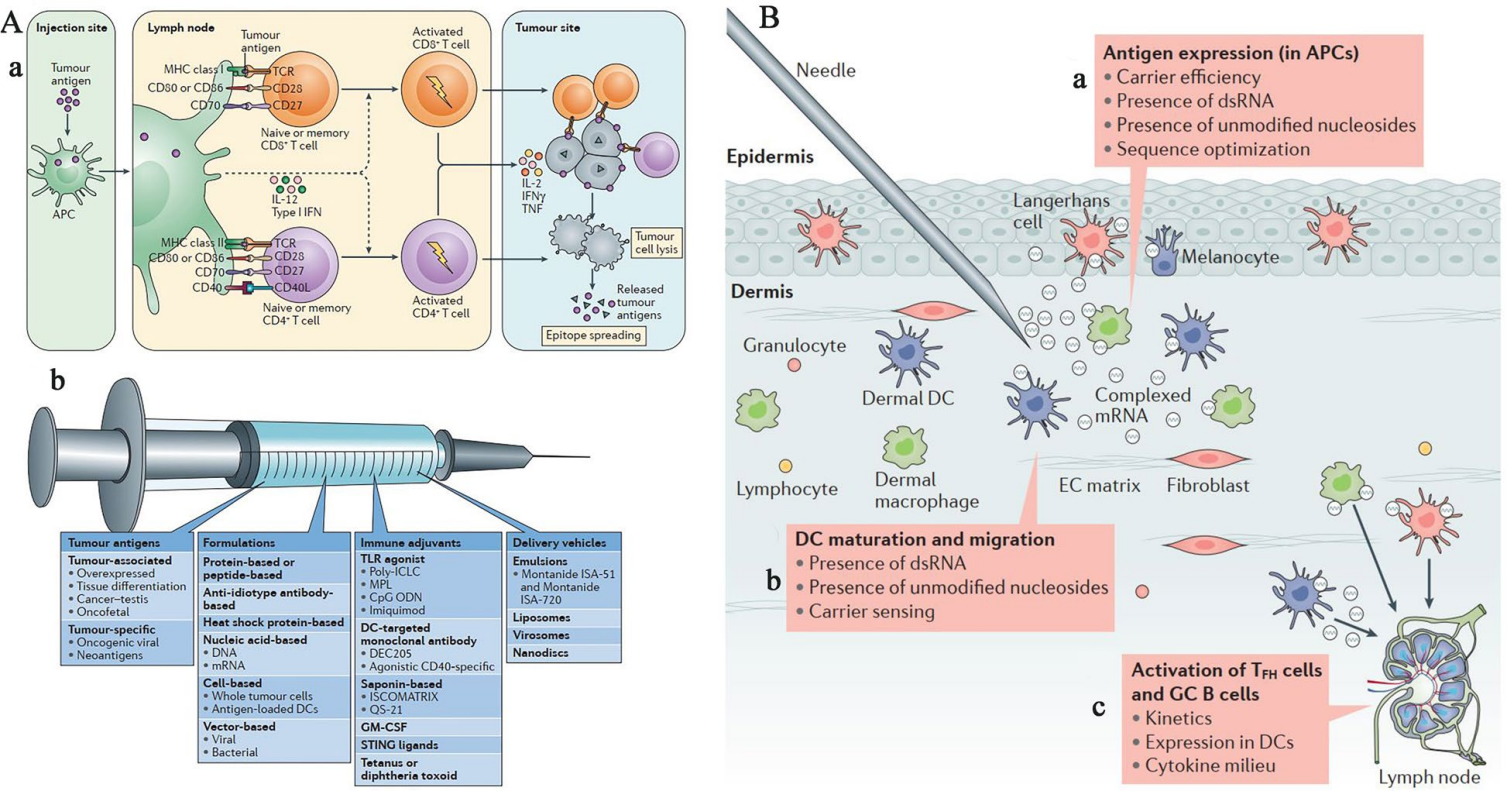
**A** Key elements and processes of an effective cancer vaccine. a The tumor antigen presentation process involves several steps. Initially, APCs such as DCs encounter antigens at the injection site or have antigens externally loaded onto them before injection, as in the case of DC vaccines. Antigen-loaded APCs then travel through the lymphatic system to the draining lymph nodes, where T cell activation primarily takes place. In the lymph node, mature DCs present tumor-derived peptides on MHC class I and II molecules to CD8 + and CD4 + T cells, both of naïve and memory types. The development of tumor-specific T cell responses is facilitated by delivering a costimulatory "signal 2" to T cells through interactions like CD80-CD28, CD86-CD28, CD70-CD27, and CD40-CD40 ligand (CD40L). Costimulation is enhanced by IL-12 and type I interferons (IFNs) produced by DCs. These interactions collectively support the generation and expansion of activated tumor-specific CD4 + and CD8 + T cell populations. CD4 + and CD8 + T cells migrate to the tumor site, and upon recognizing their specific antigens, they can destroy tumor cells through cytotoxicity and effector cytokine production, such as IFNγ and tumor necrosis factor (TNF). Consequently, lysed tumor cells release tumor antigens, which can be captured, processed, and presented by APCs to induce polyclonal T cell responses, thus increasing the antigenic variety of the anti-tumor immune response and leading to epitope spreading. b Cancer vaccines consist of four main components: tumor antigens, formulations, immune adjuvants, and delivery vehicles. Abbreviations: CpG ODN, CpG oligodeoxynucleotide; GM-CSF, granulocyte–macrophage colony-stimulating factor; MPL, monophosphoryl lipid A; poly-ICLC, polyinosinic–polycytidylic acid with polylysine and carboxymethylcellulose; STING, stimulator of interferon genes protein; TCR, T cell receptor; TLR, Toll-like receptor. Reprinted from [241] with permission from Springer Nature. **B** Key factors in the efficacy of directly injected mRNA vaccines. The effectiveness of an injected mRNA vaccine depends on several factors: the amount of antigen expression in professional APCs, which is affected by the carrier's efficiency, the presence of PAMPs such as double-stranded RNA (dsRNA) or unmodified nucleosides, and the optimization of the RNA sequence (including codon usage, G:C content, and 5' and 3' UTRs); the maturation and migration of DCs to secondary lymphoid tissues, which is enhanced by PAMPs; and the vaccine's capacity to stimulate strong T follicular helper (TFH) cell and germinal center (GC) B cell responses, an aspect that is not yet well-understood. An intradermal injection is provided as an illustration. EC refers to extracellular. Reprinted from [126] with permission from Springer Nature

The manner in which messenger RNA vaccines are delivered can have a substantial impact on the degree to which they are successful [69]. Figure 7-A illustrates the key elements and processes of an effective cancer vaccine, focusing on the tumor antigen presentation process and the vaccine components.

In the antigen presentation process, APCs like DCs encounter or receive externally-loaded antigens before injection. These antigen-loaded APCs travel through the lymphatic system to draining lymph nodes, where T cell activation primarily occurs. Mature DCs present tumor-derived peptides on MHC class I and II molecules to CD8 + and CD4 + T cells, with costimulatory "signal 2" interactions enhancing the development of tumor-specific T cell responses. IL-12 and type I interferons produced by DCs further augment costimulation. These interactions result in the generation and expansion of activated tumor-specific CD4 + and CD8 + T cell populations, which can then migrate to the tumor site and destroy tumor cells.

The lysed tumor cells release antigens that induce polyclonal T cell responses, increasing the antigenic variety of the anti-tumor immune response and leading to epitope spreading. Cancer vaccines are composed of four main components: tumor antigens, formulations, immune adjuvants, and delivery vehicles. These components work together to facilitate a robust and targeted immune response against cancer cells. In the process of developing mRNA cancer vaccines, both conventional and nonconventional administration systems (intralipid) have been utilized [242]. Conventional administration systems include intradermal, intramuscular, subcutaneous, and intranasal [242]. Nonconventional administration systems include intralipid (intranodal, intravenous, intrasplenic or intratumoural) [243]. Table 3 outlines the different types of mRNA vaccines for direct injection, each with their own unique set of advantages and disadvantages.

**Table 3:** Different types of mRNA vaccines for direct injection

mRNA Vaccine Type	Advantages	Disadvantages	Immunogenicity	Efficacy	Safety	Stability	Dosage	Manufacturing Complexity	Clinical Trials	Reference
Naked mRNA vaccine	Easy to manufacture and administer; low cost	Inefficient delivery and translation; low immunogenicity	Moderate	Moderate	Good	Short half-life; degradation by RNases	High	Low	Phase I/II clinical trials	[244]
Lipid nanoparticle (LNP) mRNA vaccine	Efficient delivery and translation; high immunogenicity	Expensive to manufacture; potential for toxicity	High	High	Good	Long half-life; stability in vivo	Low	Moderate	Phase III clinical trials	[245]
Adjuvant-assisted mRNA vaccine	Improved immunogenicity; low cost	Limited clinical data; potential for toxicity	High	High	Good	Short half-life; degradation by RNases	Moderate	Low	Early phase clinical trials	[246]
Self-amplifying mRNA vaccine	Low dose required; high immunogenicity	Limited clinical data; potential for toxicity	High	High	Good	Long half-life; stability in vivo	Low	High	Preclinical studies	[247]

Naked mRNA vaccines are easy to manufacture and administer, and are relatively low-cost. However, they suffer from inefficient delivery and translation, leading to low immunogenicity. These vaccines have moderate efficacy and good safety, but their stability is hindered by a short half-life and susceptibility to degradation by RNases. Naked mRNA vaccines require a high dosage and have low manufacturing complexity, with Phase I/II clinical trials serving as references. An unconventional technique of delivering vaccines that uses intranasal administration of naked mRNA has been shown to be successful [41]. If mRNA is injected directly into secondary lymphoid tissue, antigen-presenting cells are able to skip the necessity for DC migration and be directed straight to the site of T cell activation [248]. Numerous studies have demonstrated that naked mRNA delivered via intranasal injection is selectively taken up by DCs and has the ability to generate potent antitumor T cell responses, either for therapeutic or preventative purposes [249]. Results from an earlier trial were found to be comparable when intrasplenic administration was used [249]. It was discovered in a number of trials that it was possible to increase immune responses to intranasal mRNA vaccination by coadministering the DC-activating protein FMS-related tyrosine kinase 3 ligand, also known as FLT3L [250]. This was accomplished by giving the vaccine at the same time [250].

In mice, mRNAs encoding tumor-associated antigens were delivered intranasally using the TriMix adjuvant [56]. This resulted in powerful antigen-specific CTL responses and the suppression of a wide array of tumor types [229]. According to a recent study, the intranasal injection of mRNA encoding the E7 protein of HPV 16 with TriMix was found to increase the frequency of CD8 + T cells invading tumors [251]. This was confirmed to be the case after the injection [251]. Clinical trials using intranodally injected naked mRNA encoding tumour-associated antigens are being conducted on patients with advanced melanoma (NCT01684241) and patients with hepatocellular carcinoma (NCT01684242) (EudraCT: 2012–005572-34) [252].

DCs electroporated with mRNA encoding the melanoma-associated antigens tyrosinase or gp100 and TriMix and administered intranasally to patients with advanced melanoma evoked modest antitumor responses, according to the findings of one investigation [253]. Intranasal vaccination is advantageous since it does not require the use of needles and is not intrusive [254]. As a result, the rate at which DCs take up antigens is increased [254]. In animal models of preventative and therapeutic tumors, employing the OVA-expressing E.G7-OVA T lymphoblastic cell line, intranasal administration of mRNA complexed with Stemfect (Stemgent) LNPs was found to be effective in delaying the start of tumor growth and improving survival rates [255]. There is reason for optimism regarding the use of intratumorally mRNA immunization as a therapy option because it can promptly and selectively activate tumor-resident T cells [57]. These vaccines often make use of immune stimulatory substances in order to promote tumor-specific immunity locally [256]. This is done rather than adding mRNAs that encode for antigens that are linked with tumors [256]. Utilizing the inherently immunogenic properties of mRNA, earlier research demonstrated that mRNA expressing a gene that is unrelated to the process of carcinogenesis (GLB1) could decrease the formation of tumors and provide protection in a mouse model of glioblastoma [257].

In a more recent study, researchers found that intratumor delivery of mRNA encoding an engineered cytokine based on interferon- (IFN) fused to a transforming growth factor- (TGF) antagonist increased the cytolytic capacity of CD8 + T cells and modestly slowed the growth of tumors in OVA-expressing lymphoma or lung carcinoma mouse models [258]. These findings were made possible by the fact that the TGF antagonist was fused to the interferon Additionally, it has been demonstrated in a number of mouse models that intratumor delivery of TriMix mRNA that does not contain tumor-associated antigens activates CD8 + DCs and tumor-specific T lymphocytes, which in turn results in a delay in the growth of the tumor [259]. This has been accomplished by inhibiting the production of tumor-associated antigens [259].

Systemic distribution of mRNA vaccines is unusual because of concerns over aggregation with serum proteins and rapid extracellular mRNA degradation [260]; as a result, packaging mRNAs into carrier molecules is necessary [261]. As was been discussed in further detail above, the absorption of mRNA, the pace at which it is translated into proteins, and its safety against destruction by RNases are all issues that have been addressed through the creation of a wide variety of delivery formulations [261]. Following the systemic delivery of mRNA vaccines, the biodistribution of these agents presents yet another formidable obstacle [260].

When numerous cationic LNP-based complexing agents are administered intravenously, the majority of the traffic is directed to the liver, which may not be in the best condition for DC activation [262]. Recent research has led to the discovery of a strategy for the effective DC-specific targeting of mRNA vaccines after their delivery via the systemic route [233]. An mRNA-lipoplex delivery platform, also known as an mRNA-liposome complex, was developed by using cationic lipids and neutral helper lipids [263]. It was discovered that the ratio of lipids to mRNA, and consequently the net charge of the particles, has a significant impact on the biodistribution of the vaccine [57].

A positively charged lipid particle's major target was the lung, while a negatively charged particle's primary target was DCs in secondary lymphoid organs and the bone marrow [264]. The negatively charged particle stimulated immune responses against tumor-specific antigens, which led to considerable tumor regression in several mouse models [265]. As a result of the absence of toxicity in rats and apes, clinical trials to investigate the efficacy of this approach in the treatment of metastatic melanoma and triple-negative breast cancer are currently being carried out NCT02410733 and NCT02316457 [266]. Figure 7-B highlights the critical factors influencing the effectiveness of directly injected mRNA vaccines. The efficacy of these vaccines is contingent upon several aspects, including the level of antigen expression in professional antigen-presenting cells (APCs). This expression is influenced by the carrier's efficiency, the presence of pathogen-associated molecular patterns (PAMPs) like double-stranded RNA (dsRNA) or unmodified nucleosides, and the optimization of the RNA sequence, which encompasses codon usage, G:C content, and 5' and 3' UTRs. Additionally, the maturation and migration of DCs to secondary lymphoid tissues play a crucial role, with PAMPs serving to enhance this process. Finally, the vaccine's ability to stimulate robust T follicular helper (TFH) cell and germinal center (GC) B cell responses is essential, though this area warrants further investigation. Figure 7 exemplifies these factors through an intradermal injection, with "EC" representing the extracellular components involved.

## Therapeutic considerations and challenges

### Good manufacturing practice production

mRNA is synthesized in vitro utilizing recombinant enzymes, ribonucleotide triphosphates (NTPs), and a DNA template [267]. Due to its high reaction yield and convenience of usage, mRNA can be made in a compact GMP facility [268]. Length of RNA, nucleotide and capping chemistry, and product purification govern the production process, which is sequence-independent [269]. However, extreme length may provide problems [269]. This technology is well-suited for rapid response to new infectious diseases since it can create any encoded protein immunogen [270]. All of the enzymes and reaction components needed for GMP manufacture of mRNA are available from commercial providers as synthesized chemicals or bacterially produced, animal component-free reagents [271]. GMP-grade traceable plasmid DNA, phage polymerases, capping enzymes, and NTPs are available [272]. Other ingredients are pricey or unavailable [272]. As mRNA therapy production increases up, more cost-effective GMP source materials may become available [273]. GMP mRNA production begins with DNA template synthesis and continues with enzymatic IVT [274]; this is the same multistep approach used for research-scale synthesis, with extra tests to assure safety and potency [274]. Depending on the mRNA construct and chemistry involved, this process may need small alterations for changing nucleosides, capping techniques, or the removal of a template [275]. By linearizing Escherichia coli template plasmid DNA with a restriction enzyme, runoff transcripts with a 3′-terminal poly(A) tract are synthesized [276]. A bacteriophage-derived DNA-dependent RNA polymerase produces mRNA from NTPs (such as T7, SP6, or T3) [277]. DNase destroys template DNA [277]. To aid in vivo translation, mRNA is capped enzymatically or chemically [275]. mRNA synthesis may yield 2 g l1 of full-length mRNA under ideal conditions. After being synthesized, mRNA is purified to remove any residual enzymes, nucleotides, DNA, or RNA [276]. Derivatized microbeads in batch or column forms are more practical for large-scale purification in the clinic [278]. dsRNA must be removed from various mRNA platforms to prevent interferon-dependent translation inhibition [279]. Reverse-phase FPLC has attained laboratory-scale success, and scalable aqueous purification approaches are being studied [280]. Once mRNA is sterile-filtered and stored in a final buffer, vials can be filled for clinical usage [279]. RNA can be degraded chemically and enzymatically [276].

To prevent mRNA instability, formulation buffers may include antioxidants and chelators [276]. Developing messenger RNA pharmaceuticals [281]. Vaccines are usually stored at 70 °C, but researchers are experimenting on warmer formulations [282]. Stable formulations at room temperature or refrigeration can be made [283]. The RNActive platform can be lyophilized and stored at 5–25 °C for 3 years and 40 °C for 6 months [283]. A separate study found that freeze-dried naked mRNA lasts at least 10 months in the refrigerator [284]. Packaging mRNA in nanoparticles or adding RNase inhibitors can boost product stability [285]. At least 6 months of stability has been demonstrated for lipid-encapsulated mRNA (Arbutus Biopharma, personal communication), but longer-term unfrozen preservation has not been documented [285].

### Regulatory aspects

Neither the FDA nor the European Medicines Agency (EMA) have established any concrete rules concerning mRNA vaccine products as of yet [224]. As can be seen by the increasing number of clinical trials carried out under the control of the EMA and the FDA, the regulatory bodies have given their stamp of approval to the methods that have been proposed by various organisations to demonstrate that products are safe and suitable for testing in humans [286]. Given that mRNA is a type of genetic immunogen that is used in vaccinations, it is reasonable to anticipate that the principles that have been established for DNA vaccines and gene therapy vectors will be able to be applied to mRNA with only minor adjustments to account for its particular qualities [287]. Some researchers highlight, in their examination of EMA guidelines for RNA vaccines, how the laws for preventative and therapeutic uses of RNA vaccines differ substantially [288]. Regardless of the precise classification under the guidelines that are currently in place, there are similarities between the assertions made in these guidance documents and the findings of recently published clinical studies. These similarities can be found in both sets of materials [289]. A recent article that was published on an mRNA vaccine for the influenza virus includes data that indicate biodistribution and durability in mice, protection from sickness in a relevant animal model (ferrets), and immunogenicity, local reactogenicity, and toxicity in humans [230]. As mRNA products continue to garner more attention in the vaccination industry, it is expected that specialized guidance will be developed to describe the requirements to develop and investigate new mRNA vaccines [287].

### Safety

The mRNA vaccines have gained significant attention and recognition in recent years due to their remarkable potential in combating infectious diseases [290]. They offer a comprehensive approach to understanding mRNA synthesis, production, stability, regulatory considerations, and safety aspects [291].

One of the notable advantages of mRNA vaccines is their safety profile. Unlike traditional vaccines that may contain attenuated or inactivated pathogens, mRNA vaccines do not require the introduction of live organisms into the body [292]. Instead, they utilize the body's own cellular machinery to produce specific viral or pathogenic proteins, triggering an immune response. This mechanism significantly reduces the risk of infection or adverse effects associated with live attenuated vaccines [9]. Additionally, the production of mRNA vaccines is relatively convenient compared to conventional vaccines. Once the genetic sequence of the pathogen is identified, the mRNA can be synthesized in the laboratory using well-established techniques. This flexibility allows for a faster response to emerging infectious diseases, as the production process can be rapidly scaled up [293]. However, it is important to acknowledge that there are still safety considerations and ongoing research associated with mRNA vaccines. One concern is the potential for unwanted immune responses or off-target effects. While extensive preclinical and clinical testing is conducted to ensure safety, ongoing surveillance and evaluation are necessary to monitor any long-term effects or rare adverse events [294]. Regulatory considerations also play a crucial role in the development and approval of mRNA vaccines. Regulatory agencies worldwide assess the safety, efficacy, and quality of these vaccines through rigorous evaluation processes before granting authorization for public use [9]. Furthermore, mRNA vaccines have the potential for rapid development and deployment during outbreaks or pandemics [295]. The production process for mRNA vaccines can be quickly adjusted to target new variants or strains of pathogens. This adaptability is particularly valuable in the face of emerging infectious diseases, where timely response is crucial [290]. Moreover, mRNA vaccines are well-suited for large-scale manufacturing. The production of mRNA does not require the complex and time-consuming processes involved in traditional vaccine production, such as growing large quantities of the pathogen in cell cultures [292]. Instead, mRNA can be synthesized using readily available materials and standardized laboratory techniques. This streamlined production process allows for increased vaccine availability and accessibility, particularly in global health emergencies [293]. Another advantage of mRNA vaccines is their potential to elicit a strong and durable immune response. By delivering genetic instructions directly to cells, mRNA vaccines can stimulate both the humoral immune response (involving antibody production) and the cellular immune response (involving T cells). This dual immune activation can contribute to robust and long-lasting protection against the targeted pathogen [290]. It is worth noting that mRNA vaccines have undergone rigorous testing and regulatory scrutiny to ensure their safety and efficacy [292]. Clinical trials involving tens of thousands of participants have demonstrated their effectiveness in preventing disease and reducing severe outcomes [293]. These trials have also shown a favorable safety profile, with most adverse reactions being mild and transient. While mRNA vaccines have exhibited great promise, ongoing research and evaluation remain important [294]. Further studies are necessary to monitor the long-term safety, effectiveness, and durability of immune responses generated by mRNA vaccines. Additionally, efforts are underway to address vaccine hesitancy and ensure equitable distribution to populations worldwide [292]. Because vaccinations for prevention are meant to be administered to otherwise healthy persons, stringent safety criteria must be adhered [290]. When compared to other platforms for vaccination, such as live viruses, viral vectors, inactivated viruses, and subunit protein vaccines, the generation of mRNA does not require the use of harmful chemicals or cell cultures that could become contaminated with adventitious viruses [291]. This is in contrast to other vaccination platforms, such as live viruses, viral vectors, inactivated viruses, and subunit protein vaccines [291]. In addition, because mRNA processing takes place so quickly, there are less opportunities for the entry of bacteria that could cause contamination [296]. In those who have received vaccination, the mRNA does not face possible dangers such as infection or the incorporation of the vector into the DNA of the host cell [291]. For the reasons that were just discussed, mRNA vaccines have been considered to be a safe vaccine choice.

Several different mRNA vaccines have been shown to be safe and well tolerated after going through clinical studies ranging from phase I to phase IIb of testing [291]. On the other hand, recent human trials employing a range of mRNA platforms have showed reactions ranging from modest to severe at the injection site or across the body [292]. These responses have been seen. Local and systemic inflammation, the biodistribution and persistence of expressed immunogen, stimulation of auto-reactive antibodies, and the potential toxic effects of any non-native nucleotides and delivery system components are all potential safety concerns that are likely to be evaluated in future preclinical and clinical studies [292].

Inflammation can occur both locally and systemically [293]. Several different mRNA-based vaccine platforms have the potential to induce intense type I interferon responses [297]. These responses have been related to inflammation and even autoimmune diseases [297]. Consequently, if people who are at a higher risk of autoimmune reactions are identified in advance, it may be possible to take adequate precautions prior to giving an mRNA vaccine [9]. During mRNA immunization, the presence of extracellular RNA presents yet another opportunity for damage to occur [295]. Because naked RNA located outside of cells can improve the permeability of closely packed endothelial cells, oedema may be induced, at least in part, by this property of naked RNA [295]. Extracellular RNA, according to the findings of yet another study, had a role in the coagulation of blood as well as the formation of pathogenic thrombi [294]. Because of this, it will be required to conduct continuous safety studies when new mRNA techniques and delivery systems are evaluated in wider patient groups.

### The role of adjuvants in enhancing the stability and efficacy of mRNA vaccines

Adjuvants play a crucial role in enhancing the stability and efficacy of mRNA vaccines [74]. mRNA vaccines contain fragile mRNA molecules that encode the instructions for cells to produce viral or antigenic proteins, triggering an immune response [298]. However, mRNA is inherently unstable and can be rapidly degraded by enzymes in the body and easily cleared from the system [299]. To overcome these challenges, adjuvants are included in mRNA vaccines to protect and stabilize the mRNA molecules, thereby increasing their potency and duration of action [299].

#### Protecting mRNA from degradation

Adjuvants are substances that are often used in vaccines to enhance the immune response generated by the vaccine [300]. While their primary role is to improve the efficacy of vaccines, adjuvants can also play a crucial role in protecting mRNA from degradation in mRNA-based vaccines. mRNA is a fragile molecule that can be easily degraded by enzymes called nucleases present in the body [301]. Adjuvants can provide a protective shield around the mRNA, shielding it from these enzymes and increasing its stability. By preserving the integrity of the mRNA, adjuvants help ensure that the desired genetic information encoded in the mRNA is effectively delivered to cells, promoting the production of the intended protein and triggering a robust immune response [302]. This protection is essential for the success and durability of mRNA-based vaccines, allowing them to effectively elicit the desired immune response and contribute to effective immunization against infectious diseases [301].

#### Enhancing cellular uptake

One important aspect of their function is their ability to enhance cellular uptake, which refers to the process by which cells internalize and take up external substances or particles [303]. Adjuvants can facilitate cellular uptake by several mechanisms [304]. Firstly, they can improve the stability and solubility of the vaccine or drug molecules, ensuring their effective delivery to the target cells [304]. This is particularly important for hydrophobic or poorly soluble compounds that might otherwise be rapidly cleared or degraded in the body. Secondly, adjuvants can enhance the recognition and binding of the vaccine or drug molecules to specific receptors on the surface of target cells. By promoting receptor-ligand interactions, adjuvants increase the likelihood of cellular uptake, as these interactions trigger internalization pathways within the cells. Moreover, adjuvants can stimulate the immune system and induce an inflammatory response at the injection site [305]. This local immune activation leads to the recruitment of immune cells, such as macrophages and DCs, to the site of administration. These immune cells play a crucial role in phagocytosis, the process by which they engulf and internalize foreign substances [306]. By promoting phagocytosis, adjuvants enhance the cellular uptake of vaccines or drugs, ultimately leading to an improved immune response or therapeutic effect. Adjuvants play a vital role in enhancing cellular uptake by improving the stability and solubility of vaccine or drug molecules, facilitating their binding to cell surface receptors, and promoting phagocytosis through immune activation [307]. These mechanisms collectively contribute to the effectiveness of vaccines and drug delivery systems, ultimately leading to improved therapeutic outcomes [308].

#### Promoting immune activation

Adjuvants are substances that are often recommended for use in mRNA vaccines to enhance their effectiveness by promoting immune activation [309]. mRNA vaccines work by introducing a small piece of genetic material, called messenger RNA (mRNA), into the body. This mRNA encodes instructions for the production of a viral protein, which triggers an immune response and leads to the development of immunity against the targeted pathogen [310]. However, mRNA vaccines alone may not always elicit a robust immune response. That's where adjuvants come into play. Adjuvants are added to the vaccine formulation to enhance the immune response, making it stronger, more durable, and potentially more specific. These substances can stimulate the innate immune system, which serves as the first line of defense, and enhance the adaptive immune response, which involves the production of specific antibodies and memory cells [311]. Adjuvants work by activating certain receptors on immune cells, such as DCs, macrophages, and B cells. This activation leads to the release of signaling molecules, such as cytokines, which promote the recruitment and activation of other immune cells [311, 312]. Adjuvants can also enhance the uptake and processing of the mRNA vaccine by antigen-presenting cells, which improves the presentation of viral proteins to immune cells and increases the likelihood of a robust immune response [313]. By promoting immune activation, adjuvants help to optimize the effectiveness of mRNA vaccines, leading to stronger and longer-lasting immunity against the targeted pathogen [314]. These adjuvants undergo rigorous testing to ensure their safety and efficacy before being incorporated into vaccine formulations [315]. The development and utilization of adjuvants in mRNA vaccines represent an important strategy to enhance the potency and effectiveness of these innovative vaccines, ultimately contributing to the protection of individuals and populations against infectious diseases [316].

#### Modulating immune response

Adjuvants can influence the type and strength of the immune response elicited by the mRNA vaccine [317]. Different adjuvants can bias the immune response towards specific types, such as a Th1 (cell-mediated) or Th2 (antibody-mediated) response [318]. This modulation is crucial for tailoring the vaccine's effectiveness to combat specific diseases or pathogens [319].

#### Prolonging antigen presentation

Adjuvants can extend the presentation of the antigen (viral or antigenic protein) derived from the mRNA vaccine [320]. They help in promoting antigen persistence and slow down the clearance of the antigen from the injection site, allowing for a more prolonged and robust immune response [321].

### Applications of mRNA vaccines in preventive and therapeutic contexts

The mRNA vaccines are administered to individuals who are at risk of being exposed to a specific pathogen, such as a virus or bacteria [311, 312]. The mRNA vaccines provide instructions to the cells in the body to produce a harmless piece of the pathogen, typically a protein or antigen, which then stimulates an immune response. This immune response includes the production of antibodies and activation of immune cells specific to the pathogen [304]. If the individual is later exposed to the actual pathogen, their immune system can recognize and respond to it more effectively, preventing infection or reducing its severity. mRNA vaccines have been successfully developed for various infectious diseases, including COVID-19, influenza, and others [319]. The mRNA vaccines are used as a treatment option for individuals who have already contracted a specific disease, such as cancer or certain viral infections. The therapeutic mRNA vaccines work by delivering genetic instructions to the cells, directing them to produce specific proteins that are characteristic of the disease [321]. These proteins can be tumor-specific antigens in the case of cancer or viral proteins in the case of viral infections. By producing these disease-specific proteins, the immune system is stimulated to recognize and mount an immune response against the diseased cells or viruses [315]. Therapeutic mRNA vaccines hold promise in cancer treatment as they can potentially enhance the body's ability to target and eliminate cancer cells [304].

### Safety and tolerability of mRNA vaccines

The mRNA vaccines have gained recognition for being largely risk-free and well-tolerated [321]. There are a few reasons for this. Firstly, mRNA vaccines do not contain live viruses or pathogens, eliminating the risk of developing the disease they aim to prevent or treat [318]. Instead, they consist of a small piece of genetic material that encodes a specific protein. Secondly, mRNA vaccines are transient, meaning that once the genetic material is taken up by cells and the protein is produced, the mRNA quickly degrades and is eliminated from the body [313]. This temporary nature ensures that there is no long-term genetic alteration. Additionally, mRNA vaccines do not integrate into the host genome, further minimizing any potential long-term risks. Moreover, extensive clinical trials and rigorous safety evaluations are conducted before their approval, ensuring that they meet strict safety standards [321]. Adverse reactions to mRNA vaccines are typically mild and temporary, such as pain at the injection site, fatigue, or fever, which are common with most vaccines [304]. Furthermore, the technology behind mRNA vaccines has been extensively studied and refined over many years, providing a solid foundation for their safety and tolerability [321]. The mRNA used in vaccines is carefully engineered and optimized to enhance stability and reduce any potential side effects. Modern mRNA vaccines also benefit from lipid nanoparticle delivery systems, which help protect the mRNA and facilitate its efficient uptake by cells [313]. Another key factor contributing to the safety of mRNA vaccines is the stringent regulatory processes and rigorous testing they undergo before approval [304]. These vaccines undergo comprehensive preclinical studies in animals to evaluate their efficacy and safety. Subsequently, they progress through multiple phases of clinical trials involving thousands of human participants, where their safety, immunogenicity, and efficacy are thoroughly assessed [321]. Additionally, the extensive post-approval monitoring and surveillance systems enable the rapid detection and investigation of any potential adverse events. This ongoing monitoring ensures that the safety profile of mRNA vaccines is continuously evaluated and any rare or unexpected side effects can be promptly addressed [313]. The collective evidence from clinical trials, real-world data, and the successful deployment of mRNA vaccines in millions of individuals supports their remarkable safety and tolerability [313]. The benefits of mRNA vaccines in preventing serious illnesses, hospitalizations, and deaths far outweigh the minimal risks associated with them. Continuous monitoring and research efforts further contribute to improving our understanding of these vaccines and ensuring their ongoing safety [321].

## Strategies to improve mRNA translation efficiency and overcome the innate immunogenicity

**Fig. 8:**
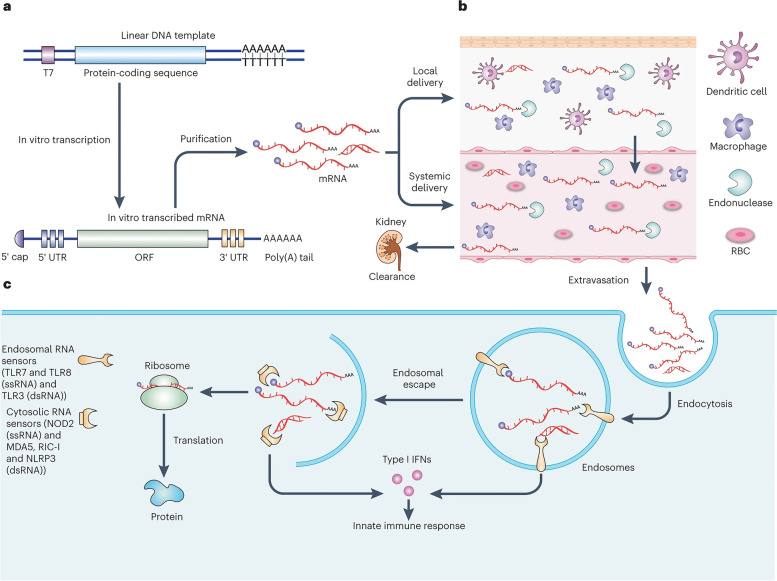
Obstacles in the clinical application of mRNA. **a** In vitro production of therapeutic mRNA involves a linear DNA template and RNA polymerase (T7), followed by purification; the mRNA consists of a 5′ cap, a 5′ UTR, an ORF that encodes the target protein, a 3′ UTR, and a poly(A) tail. **b**) Upon local or systemic administration, mRNA encounters various extracellular hurdles such as rapid breakdown by prevalent nucleases, removal through macrophage phagocytosis, and elimination via renal filtration. **c** A portion of mRNA that escapes from blood vessels can be taken up by cells. The majority of these internalized mRNAs get confined in endosomes and can be identified by endosomal and cytosolic RNA sensors, which ultimately decrease the mRNA's translation and stability. Enhancing the 5′ cap can boost the binding efficiency of cytoplasmic mRNAs to ribosomes, consequently increasing mRNA translation efficiency. Although the endosomal escape of bare and unaltered mRNA is difficult, it can be facilitated by employing mRNA carriers. Reprinted from [322] with permission from Springer Nature

Figure 8 illustrates the various obstacles encountered during the clinical application of mRNA therapies. The production of therapeutic mRNA in vitro includes the use of a linear DNA template and T7 RNA polymerase, followed by purification. The resulting mRNA is composed of a 5′ cap, a 5′ UTR, an ORF encoding the target protein, a 3′ UTR, and a poly(A) tail. When administered locally or systemically, mRNA faces numerous extracellular challenges, such as degradation by nucleases, clearance by macrophage phagocytosis, and elimination through renal filtration. Some mRNA molecules that evade these hurdles manage to enter cells, but the majority end up sequestered in endosomes. Here, they are detected by endosomal and cytosolic RNA sensors, which negatively affect mRNA translation and stability. Enhancing the 5′ cap can improve ribosome binding efficiency and thus increase mRNA translation. While endosomal escape remains a challenge for unmodified mRNA, the use of specialized carriers can facilitate this critical step and improve the overall effectiveness of mRNA-based therapies. Table 4 outlines various strategies for improving mRNA translation efficiency, which include modifications to mRNA structures and utilizing novel methods for mRNA delivery. Some of these strategies include five-prime cap (5' Cap) modification, optimization of UTRs, codon optimization, and poly(A) tail modification, among others. These approaches aim to enhance mRNA stability, translation efficiency, and immunogenicity while maintaining safety and efficacy. The use of exogenous factors, nanoparticle-based delivery systems, and viral vectors are also explored as methods for improving mRNA translation. Combining multiple strategies may result in synergistic effects, leading to increased overall efficiency. While these methods show promise, some disadvantages include increased costs, time-consuming synthesis processes, and potential off-target effects or safety concerns. Nonetheless, these innovative approaches pave the way for the development of more effective mRNA-based therapeutics and vaccines in the future.

**Table 4:** Strategies for improving mRNA translation efficiency

Strategy	Advantages	Disadvantages	Immunogenicity	Efficacy	Safety	Stability	Description	Reference
Five-prime cap (5' Cap) modification	Increase translation efficiency, reduce innate immune response	Expensive and time-consuming to synthesize	Low to moderate	High	Safe	Stable	Addition of a modified 5' cap to the mRNA molecule to improve translation efficiency	[323]
Optimization of UTRs	Improve translation initiation and elongation	May not work for all mRNA sequences	Low to moderate	High	Safe	Stable	Alteration of the 5' and 3' untranslated regions to optimize mRNA translation	[324]
Codon optimization of open reading frame (ORF)	Improve translation efficiency by selecting optimal codons for the target protein	May not be effective for all mRNA sequences, codon optimization can be difficult for complex proteins	Low to moderate	High	Safe	Stable	Modification of the mRNA sequence to include codons that are optimal for the translation of the target protein	[325]
Poly(A) tail modification	Increase translation efficiency and stability of mRNA	Can be time-consuming and costly to synthesize	Low to moderate	High	Safe	Stable	Addition of a modified poly(A) tail to the mRNA to improve stability and translation efficiency	[326]
Nucleoside-modified mRNA	Increase stability and translational efficiency of mRNA	May increase immunogenicity	Low to moderate	High	Safe	Stable	Substitution of natural nucleotides with modified nucleotides to enhance stability and translation efficiency of the mRNA	[327]
Purification of in vitro transcribed mRNA (IVT-mRNA)	Improve purity and quality of mRNA for translation	Can be time-consuming and expensive	Low to moderate	High	Safe	Stable	Purification of mRNA using chromatography to remove impurities and increase mRNA quality	[328]
Utilizing the impact of type I IFN	Enhance the immunostimulatory effect of mRNA vaccines	Can be difficult to predict the effect of IFN on specific mRNA vaccines	Low to moderate	High	Safe	Stable	Addition of type I IFN to mRNA vaccines to increase their immunostimulatory effect	[329]
Type 1 cap (Type 1 Cap-0, Type 1 Cap-1)	Improved stability and increased translation efficiency	Can be difficult to synthesize and may increase cost	Low to moderate	High	Safe	Stable	Type 1 Cap-0 and Type 1 Cap-1 modifications of the mRNA cap structure to enhance stability and translation efficiency	[330]
Modification of regulatory elements	Increase mRNA stability and translation	Can be difficult to predict the effect on specific mRNA vaccines	Low to moderate	High	Safe	Stable	Modification of regulatory elements such as miRNA binding sites, AU-rich elements, or splice sites to enhance mRNA stability and translation	[331]
Cell-specific targeting of mRNA	Increase mRNA uptake by specific cells and improve translation	May require additional modifications or targeting agents	Low to moderate	High	Safe	Stable	Incorporation of targeting moieties such as aptamers or antibodies to improve cell-specific uptake and translation of mRNA	[332]
Combination of strategies	Potential for synergistic effects and increased efficacy	May increase complexity and cost of mRNA synthesis	Low to moderate	High	Safe	Stable	Combination of two or more strategies to enhance mRNA translation efficiency and efficacy	[333]
Use of modified ribonucleoside analogs	Improve stability and translational efficiency	May increase cost of synthesis	Low to moderate	High	Safe	Stable	Substitution of ribonucleosides with modified analogs to increase mRNA stability and translation efficiency	[334]
Use of optimized mRNA 3' UTRs	Improve translation efficiency and mRNA stability	May not work for all mRNA sequences	Low to moderate	High	Safe	Stable	Modification of the 3' untranslated region (UTR) to improve mRNA stability and translation efficiency	[77]
Use of small molecule modulators	Improve mRNA translation through regulation of key signaling pathways	May have off-target effects and safety concerns	Low to moderate	High	Safe	Stable	Identification of small molecules that regulate signaling pathways involved in mRNA translation, to improve efficiency and stability	[335]
Use of exogenous factors	Improve mRNA stability and translation efficiency through the addition of exogenous factors	May be difficult to identify optimal factors and may increase cost of synthesis	Low to moderate	High	Safe	Stable	Addition of exogenous factors such as chaperones or translation initiation factors to improve mRNA stability and translation efficiency	[336]
Use of self-amplifying mRNA	Increase mRNA stability and translation efficiency through self-amplification	May require additional modifications and increased cost of synthesis	Low to moderate	High	Safe	Stable	Incorporation of self-amplifying mRNA sequences to improve mRNA stability and translation efficiency	[337]
Use of ribosome engineering	Improve mRNA translation by optimizing the function of ribosomes	May require additional modifications and increase the complexity of mRNA synthesis	Low to moderate	High	Safe	Stable	Modification of ribosomes to improve translation efficiency and efficacy of mRNA vaccines	[338]
Use of circular mRNA	Improve mRNA stability and translational efficiency	May require additional modifications and increase the cost of mRNA synthesis	Low to moderate	High	Safe	Stable	Circularization of mRNA to increase its stability and translational efficiency	[339]
Use of microRNA-mediated control	Improve mRNA stability and translation through regulation by microRNAs	May require additional modifications and increase the complexity of mRNA synthesis	Low to moderate	High	Safe	Stable	Incorporation of microRNA binding sites into the mRNA to regulate its stability and translation efficiency	[340]
Use of non-coding RNAs	Improve mRNA stability and translation through regulation by non-coding RNAs	May require additional modifications and increase the complexity of mRNA synthesis	Low to moderate	High	Safe	Stable	Incorporation of non-coding RNAs such as long non-coding RNAs or circular RNAs to regulate mRNA stability and translation efficiency	[341]
Use of modified mRNA 5' cap structures	Improve mRNA stability and translation efficiency	May increase the complexity and cost of mRNA synthesis	Low to moderate	High	Safe	Stable	Modification of the mRNA 5' cap structure to improve its stability and translation efficiency	[342]
Use of riboswitches	Improve mRNA translation by regulating its stability and translation efficiency through riboswitches	May require additional modifications and increase the complexity of mRNA synthesis	Low to moderate	High	Safe	Stable	Incorporation of riboswitches into the mRNA to regulate its stability and translation efficiency	[343]
Use of viral vectors for mRNA delivery	Increase mRNA stability and improve its delivery to target cells	May increase safety concerns and require additional modifications for clinical use	Low to moderate	High	Safe	Stable	Use of viral vectors for mRNA delivery to increase its stability and improve its delivery to target cells	[344]
Use of protease-resistant mRNA	Improve mRNA stability and translation efficiency by increasing resistance to degradation	May require additional modifications and increase the complexity of mRNA synthesis	Low to moderate	High	Safe	Stable	Modification of the mRNA to increase its resistance to proteases and improve its stability and translation efficiency	[345]
Use of hybrid mRNA molecules	Improve mRNA stability and translation efficiency by incorporating features of different mRNA types	May require additional modifications and increase the complexity of mRNA synthesis	Low to moderate	High	Safe	Stable	Hybridization of different types of mRNA to improve their stability and translation efficiency	[346]
Use of non-natural amino acids	Improve mRNA translation by incorporating non-natural amino acids	May require additional modifications and increase the complexity of mRNA synthesis	Low to moderate	High	Safe	Stable	Incorporation of non-natural amino acids into the mRNA to improve its translation efficiency	[347]
Use of nanoparticle-based delivery systems	Improve mRNA stability and delivery to target cells through the use of nanoparticle-based delivery systems	May require additional modifications and increase the complexity of mRNA synthesis	Low to moderate	High	Safe	Stable	Use of nanoparticle-based delivery systems for mRNA to improve its stability and delivery to target cells	[285]
Use of RNA–protein complexes	Improve mRNA stability and translation efficiency by incorporating RNA–protein complexes	May require additional modifications and increase the complexity of mRNA synthesis	Low to moderate	High	Safe	Stable	Incorporation of RNA–protein complexes into the mRNA to improve its stability and translation efficiency	[348]
Use of mRNA fragments	Improve mRNA stability and translation efficiency by using shorter mRNA fragments	May require additional modifications and increase the complexity of mRNA synthesis	Low to moderate	High	Safe	Stable	Use of shorter mRNA fragments to improve their stability and translation efficiency	[349]
Use of RNA editing	Improve mRNA translation by editing the mRNA sequence to optimize its translation	May require additional modifications and increase the complexity of mRNA synthesis	Low to moderate	High	Safe	Stable	Editing of the mRNA sequence to improve its translation efficiency and efficacy	[120]
Use of RNA interference (RNAi)	Improve mRNA translation and stability through gene silencing by RNAi	May require additional modifications and increase the complexity of mRNA synthesis	Low to moderate	High	Safe	Stable	Use of RNAi to silence genes that interfere with mRNA stability and translation	[156]
Use of translational enhancers	Improve mRNA translation by incorporating translational enhancers	May require additional modifications and increase the complexity of mRNA synthesis	Low to moderate	High	Safe	Stable	Incorporation of translational enhancers to improve mRNA translation efficiency	[350]
Use of RNA-binding proteins	Improve mRNA stability and translation efficiency by incorporating RNA-binding proteins	May require additional modifications and increase the complexity of mRNA synthesis	Low to moderate	High	Safe	Stable	Incorporation of RNA-binding proteins to improve mRNA stability and translation efficiency	[351]
Use of modified nucleotides	Improve mRNA stability and translation efficiency by incorporating modified nucleotides	May require additional modifications and increase the complexity of mRNA synthesis	Low to moderate	High	Safe	Stable	Incorporation of modified nucleotides to improve mRNA stability and translation efficiency	[120]
Use of RNA aptamers	Improve mRNA stability and translation efficiency by incorporating RNA aptamers	May require additional modifications and increase the complexity of mRNA synthesis	Low to moderate	High	Safe	Stable	Incorporation of RNA aptamers to improve mRNA stability and translation efficiency	[152]
Use of RNA secondary structures	Improve mRNA stability and translation efficiency by incorporating RNA secondary structures	May require additional modifications and increase the complexity of mRNA synthesis	Low to moderate	High	Safe	Stable	Incorporation of RNA secondary structures to improve mRNA stability and translation efficiency	[352]
Use of optimized UTRs	Improve mRNA translation by optimizing UTRs for efficient translation	May require additional modifications and increase the complexity of mRNA synthesis	Low to moderate	High	Safe	Stable	Optimization of UTRs to improve mRNA translation efficiency	[77]
Use of modified poly(A) tails	Improve mRNA stability and translation efficiency by incorporating modified poly(A) tails	May require additional modifications and increase the complexity of mRNA synthesis	Low to moderate	High	Safe	Stable	Incorporation of modified poly(A) tails to improve mRNA stability and translation efficiency	[326]
Use of codon optimization	Improve mRNA translation by optimizing codon usage for efficient translation	May require additional modifications and increase the complexity of mRNA synthesis	Low to moderate	High	Safe	Stable	Optimization of codon usage to improve mRNA translation efficiency	[353]
Use of 5’ cap modification	Improve mRNA stability and translation efficiency by modifying the 5’ cap structure	May require additional modifications and increase the complexity of mRNA synthesis	Low to moderate	High	Safe	Stable	Modification of the 5’ cap structure to improve mRNA stability and translation efficiency	[354]
Use of synthetic mRNAs	Generate synthetic mRNAs for improved translation and stability	May require additional modifications and increase the complexity of mRNA synthesis	Low to moderate	High	Safe	Stable	Use of synthetic mRNAs for improved translation and stability	[162]
Use of in vitro transcription (IVT)	Improve mRNA quality and yield through IVT	May require additional modifications and increase the complexity of mRNA synthesis	Low to moderate	High	Safe	Stable	Use of IVT for improved mRNA quality and yield	[98]
Use of type I interferons (IFNs)	Enhance mRNA immunogenicity and efficacy through the use of type I IFNs	May increase immunogenicity and require additional modifications and increase the complexity of mRNA synthesis	High	High	Safe	Stable	Use of type I IFNs to enhance mRNA immunogenicity and efficacy	[104]
Use of lipid nanoparticles	Improve mRNA stability and delivery to target cells through the use of lipid nanoparticles	May require additional modifications and increase the complexity of mRNA synthesis	Low to moderate	High	Safe	Stable	Use of lipid nanoparticles for mRNA delivery to improve its stability and delivery to target cells	[355]

### Five-prime cap (5’Cap) modification

IVT mRNAs, which mimic the eukaryotic mRNA, usually have a N7-methylated guanosine added to the first 5′ nucleotide through a 5′, 5′-triphosphate bridge for efficient translation in the eukaryotic system [356]. This 5′ m7G cap or m7Gppp- is typically referred to as “Cap 0” [354]. The 5′ cap recruits the eukaryotic eIF4E to facilitate ribosome recognition and translation initiation [356]. Both enzymatic and chemical strategies are applied for mRNA 5′ capping [357]. The most widely used in vitro post-translational capping enzymatic method is the Vaccinia capping system, which is based on the Vaccinia virus Capping Enzyme (VCE) [358]. The VCE consists of 2 subunits (D1 and D12) [358]. The D1 subunit possesses triphosphatase, guanylyl transferase, and methyltransferase activity, all of which are essential for adding a complete Cap 0 structure, while D12 plays a valid role in activating D1 [359]. Vaccinia capping system provides a near 100% capping efficiency with proper orientation, but efficient expression and purification for VCE are required for large scale capped RNA production [360].

Besides the enzymatic post-translational capping methods, chemical capping methods add cap analogs co-transcriptionally [361]. However, regular cap analog added during IVT (co-transcriptional process) can be reversely incorporated into the mRNA sequence [362]. Therefore, approximately one third of mRNA molecules are not properly methylated, with free phosphate hanging at the 5′ location, leading to low efficiency of downstream mRNA translation [362]. To prevent reverse incorporation, anti-reverse cap analogs (ARCA) have been developed [230]. ARCA is methylated at the C3 position (closer to m7G) to ensure the addition of a nucleotide only at the non-methylated guanosine during IVT [363]. ARCA capped mRNA increases and prolongs protein expression in vitro [230]. To inhibit de-capping of the corresponding mRNA and increase binding affinity to eIF4E, ARCA have been further modified within the triphosphate linkage, either through a bridging oxygen (e.g. (methylenebis) phosphonate and imidodiphosphate) or a non-bridging oxygen (e.g. phosphorothioate and phosphorselenoate) [364]. Remaining limitations of ARCA caps are: (1) Relatively low capping efficiency (60–80%); (2) Cap-0 structure is formed after capping; (3) Cap contains an unnatural O’methyl group in the C3 position that can be recognized as exogeneous motif; (4) mRNA transcript must start with guanine (G) [365].

5’cap can be added enzymatically after IVT to achieve 100% capping efficiency with a natural unmodified cap structure [366]. However, the process is costly and suffers from batch-to-batch variability. A next generation co-transcriptional cap analog, CleanCap™, was developed in 2018 to overcome the issues associated with ARCA [366]. CleanCap™ utilized an initiating capped trimer to yield a natural unmodified cap structure with increased capping efficiency to nearly 90–99% [367]. Uncapped (5’ppp or 5’pp) or abnormally capped (Cap-0) mRNAs can be recognized by PRRs, such RIG-1 and IFIT, triggering type I IFN, blocking mRNA translation [368]. Therefore, a natural Cap-1 structure is preferred [369]. Cap1 structure can be enzymatically added by guanylyl transferase and 2′-O-methyltransferases or through the co-transcriptional CleanCap™ technology [369]. To further avoid recognition by the innate immune system, capped-IVT mRNAs should be treated with phosphatases to remove uncapped phosphate, preventing PRR-mediated sensing and destruction of mRNA translation [370].

### Optimization of untranslated regions

UTRs can impact mRNA degradation rate and translation efficiency through interacting with RNA binding proteins [79]. 5′ UTR sequence can be optimized to enhance the stability of mRNA and accuracy of translation [79]. Firstly, avoid the presence of start codon (AUG), and non-canonical start codons (CUG) in the 5′ UTR, as these codons may disturb the normal translation process of ORF [371]. Secondly, avoid the presence of highly stable secondary structures, which can prevent ribosome recruitment and codon recognition [371]. Thirdly, shorter 5’UTR may be introduced as previous studies have shown that this type of 5’UTR is more conducive to mRNA translation process [372]. Ultimately, bioinformatics tool can be used to predict mRNA translation efficiency according to 5’UTR sequence. α-globin and β-globin from *Xenopus laevis* or humans contain translation and stability regulatory elements, and are commonly used as the 3′ UTR of IVT mRNA [373]. To further improve RNA stability, AU- and GU-enriched sequences can be introduced [374]. Moreover, transcription efficiency might be improved by adding 3’UTR sequence twice in tandem [374]. Overall, UTR performance is dependent on species, cell type, and cell state [375]. One needs to understand the pharmacology in the targeted cells to allow better design of UTRs of the therapeutic mRNA vaccines [376].

### Codon optimization of Open Reading Frame (ORF)

Optimization of G and cytosine (C) content in the ORF can be applied to regulate the translation elongation rate [377]. Uridine depletion is another codon optimization strategy that can directly be linked to an increased GC content [378]. Uridine-rich regions can be recognized by RIG-I, and its activation may lead to abolishing of protein expression [379]. Moreover, the sequence can be optimized to have the same ratio of every codon found naturally in highly expressed proteins in the targeted cells or to use the best pairs of codons that are commonly seen in these highly expressed proteins [379]. In addition, codons with higher tRNA abundance are usually used to replace rare codons in ORF to increase the translation rate [380]. Lastly, highly stable secondary structures and hairpin loops should be avoided in the ORF [380]. However, high translation rate is not all beneficial, as some proteins require a low translation rate to correctly and effectively fold [381]. Therefore, codon optimizations in the ORF should be carefully monitored to ensure moderate translation rate and high translation accuracy [382]. Some researchers demonstrated that sequence engineered but chemical unmodified mRNA is fully suited for use in mRNA therapies, and the protein expression level was even higher than chemically modified but without codon optimized mRNA, indicating the importance of codon optimization in improving mRNA expression efficiency [383].

### Poly (a) tail modification

Poly(A) sequence can slow down the degradation process of RNA exonuclease, increase RNA stability, and enhance translation efficiency [384]. A suitable length of Poly(A) is crucial [385]. Commonly used Poly(A) is 250 units in length, but different cells may have different preferences [385]. For example, the optimal length of poly(A) in human monocyte-derived DCs are 120–150 nucleotides, in human primary T cells are 300 nucleotides [172]. Moreover, Poly (A) binding protein (PABP) can interact with 5’cap through translational initiation factors, such as eIF4G and eIF4E, forming a close-loop to impact mRNA structure [386]. Recent study by Some researchers found that shorter poly(A) sequence could promote this closed-loop structure for efficient translation [387]. Therefore, future studies should evaluate the role of poly-A size in kinetic expression of IVT-mRNA antigen [387].

### Nucleoside modified mRNA

Another method to improve mRNA stability, translation efficiency and mRNA vaccine potency is to modify mRNA transcripts with alternative nucleotides [352]. Pseudouridine (Ψ), 1-methylpseudouridine (m1Ψ), and 5-methylcytidine (m5C) are used to replace the natural uridine and cytidine, and thus to remove intracellular signaling triggers for PKR and RIG-I, leading to enhanced antigen expression [388]. Some researchers have found that altering nucleosides in the mRNA’s structure (e.g., 5mC or Ψ) can substantially reduce innate immune activation and increase translational capacity of mRNA [11]. Post-transcriptional epigenomic RNA modifications can also be a powerful approach for improving mRNA translation and evading innate immune response [11]. Some researchers reported that post-transcriptional RNA modification with N4-aceylcytidine (ac4C) enhanced mRNA translation in vitro and in vivo [389]. Moreover, the function of post-translational epigenomic modifications in DC activation has been demonstrated by mettl3, an RNA methyl transferase which mediates mRNA m6A methylation and induces DC activation [390].

### Purification of IVT-mRNA

**Fig. 9:**
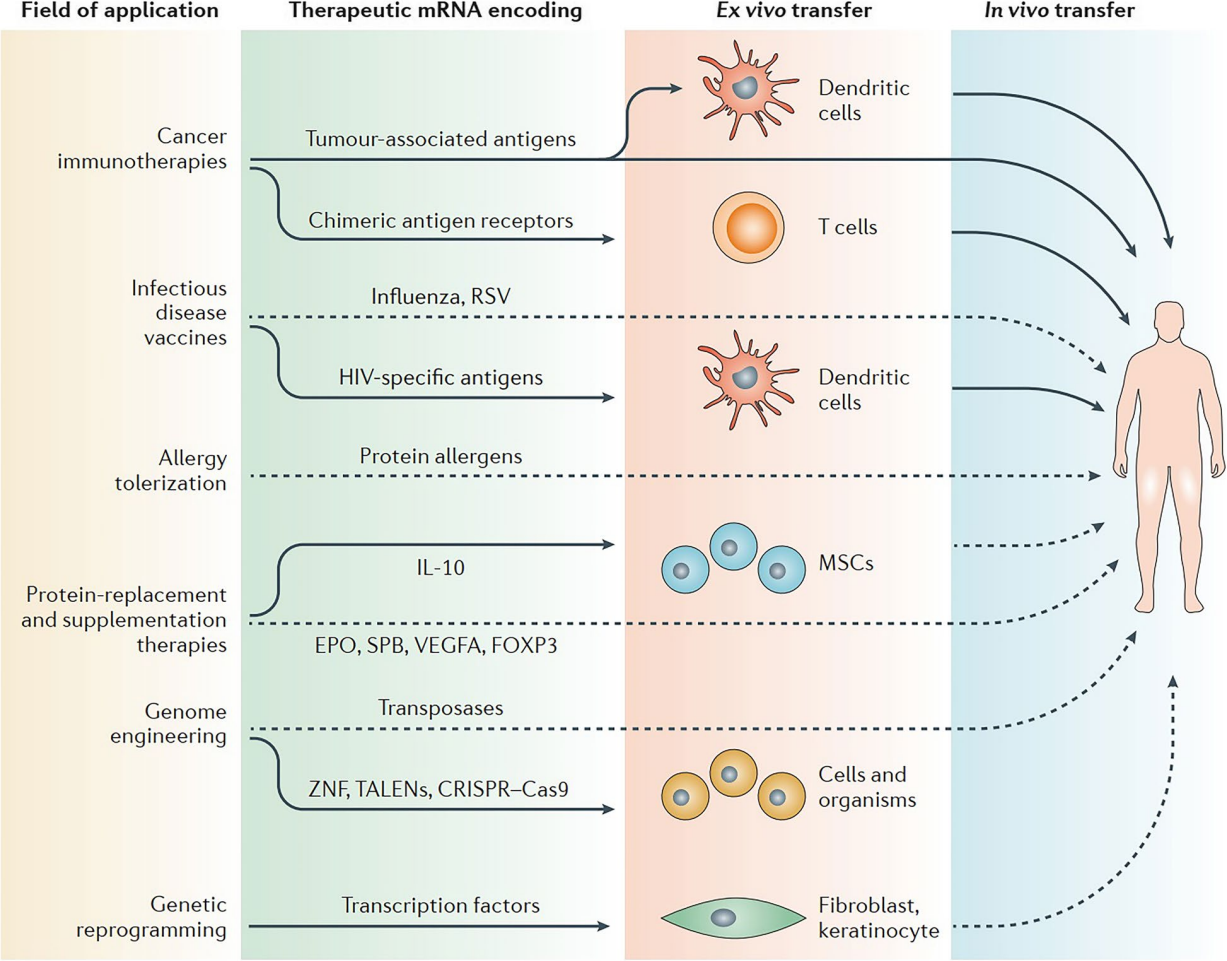
Possible therapeutic uses of IVT mRNA. Solid arrows in the right-hand column signify clinical applications, while dotted arrows indicate preclinical applications. Reprinted from [51] with permission from Springer Nature

As mentioned in Sect. "Cancer immunotherapies", phage polymerase in IVT can yield multiple contaminants, including short RNAs generated from abortive initiation event and dsRNA produced by self-complementary 3′ extension [387]. These RNA contaminants can activate intracellular PPRs, including PKR, MDA-5, OAS etc. and lead to abolish of mRNA translation and activation of innate immunity [391]. Some researchers have demonstrated that the removal of these RNA contaminants result in mRNA that does not induce IFNs and inflammatory cytokines, ultimately leading to10- to 1000-fold increase in protein production in human primary DCs [392]. dsRNA species can be reduced during IVT by decreasing Mg^2+^ concentration or by producing RNA at elevated temperature [387]. A more complete and scalable removal of dsRNA was performed by high-pressure liquid chromatography (HPLC) [393]. However, HPLC purification of mRNA is usually high cost and low yield (< 50%) [353]. Recently, a fast and cheap purification method has been reported by some researchers. The method utilized the selective binding of dsRNA to a cellulose powder in ethanol containing buffer combined with fast protein liquid chromatography (FPLC) to remove up to 90% of dsRNA [328]. Another way to completely get rid of dsRNA contaminants is through solid phase synthesis of mRNA rather than IVT [394]. For instance, some researchers have synthesized RNA fragments up to ~ 70 nucleotides using the solid phase method [395]. The RNA fragments were then ligated to become full length mRNA. This process is scalable and completely avoids the formation of dsRNA [395]. Figure 9 illustrates the potential therapeutic uses of in vitro transcribed (IVT) mRNA in various clinical and preclinical applications.

### Utilizing the impact of type I IFN for improved mRNA vaccination

As mentioned earlier, type I IFN shows paradoxical impact on the immune response of mRNA cancer vaccine [64]. Several studies have demonstrated that increased innate immune stimulation driven by mRNA and delivery system modifications are not necessary for increased immunogenicity [2]. Other studies indicated that enhanced immune responses via combination with alternative adjuvants are required for mRNA vaccines to achieve the targeted anti-tumor therapeutic outcome and improved patients’ survival [396].

Some researchers have reported mRNA pulsed with a palmitic acid-modified TLR7/8 agonist R484 markedly improved the MHC class I presentation of OVA mRNA derived antigen in APCs, subsequently induced a more effective adaptive immune response in a tumor bearing mouse model as compared to mRNA vaccine without the adjuvant [37]. Moreover, the RNActive® vaccine platform developed by CureVac AG used RNA/protamine complex as an adjuvant to activate TLR7/8, induce Th1 T cell response [397]. Enhanced antitumor immunity was achieved when dosing RNA/protamine adjuvant with the naked, unmodified mRNA encoding antigens [398]. In addition to using TLR agonists as adjuvants, stimulator of interferon genes (STING) agonists has been recently applied as immunomodulators for combination with mRNA and peptide vaccines [399]. Some researchers have shown that loading of mRNA cancer vaccines into LNPs with intrinsic STING-IFN activation function produced a potent and prolonged CD8^+^ T cells response [2]. Improved antitumor efficacies were observed in three cancer models with the addition of STING activating lipids [400].

Recently, a combination of pro-inflammatory cytokines and chemokines have also been exploited to boost the antitumor immunity of mRNA vaccines in both pre-clinical and clinical studies [37]. In one clinical study, a DC-based mRNA vaccination composed of a mixture of TAAs were administrated together with DCs electroporated with mRNA encoding CD70, CD40 ligand (CD40L), and constitutively active TLR4 (TriMix) [401]. The combination therapy resulted in an encouraging rate of tumor responses in patients with stage III or IV melanoma [402]. Costimulatory molecules CD70 and CD40L, together with active TLR4, play crucial roles in the activation of DCs and priming of CD8^+^ T cell responses [111]. The cytokine cocktails are not only used to prime DC and T cell functions, but can also be dosed intratumorally to reshape the tumor microenvironments [403]. For instance, intratumorally injection of mRNA-encoding cytokines IL-23, IL-36Ƴ, and T cell co-stimulatory OX40L can overcome the suppressive tumor environment and produce effective systemic antitumor immunity [404]. Studies in combination of adjuvants with mRNA vaccines are blooming, but this strategy should be used with caution as it could be counterproductive and paradoxical, especially when using immune-stimulatory molecules that have tight interactions with type I IFN and the innate immunity pathway [405].

### Immunogenicity of mRNA and paradoxical effects in cancer immunotherapy

Innate immune response is usually activated by host immune system through detecting exogeneous motifs called PAMPs via the pattern recognition receptors (PRRs) [406]. These receptors are particularly highly expressed in APCs, the major target cell population of mRNA cancer vaccines [2]. Exogeneous IVT mRNA is intrinsically immunostimulatory, as it is recognized by a variety of cell surface, endosome and cytosolic PRRs [370]. Recognition of IVT mRNA inside the endosome is mainly mediated by toll-like receptor (TLR)-7 and − 8 (one type of PRRs), subsequently activates the myeloid differentiation marker 88 (MyD88) pathway, leading to activation of Type-1 interferon (IFN) pathways and secretion of proinflammatory cytokines [355]. In the cytosol, these exogeneous mRNAs are sensed by other PRR families, including retinoic acid-inducible gene-I-like (RIG-I-like) receptors, oligoadenylate synthetase (OAS) receptors, and RNA-dependent protein kinase (PKR) [406].

**Fig. 10:**
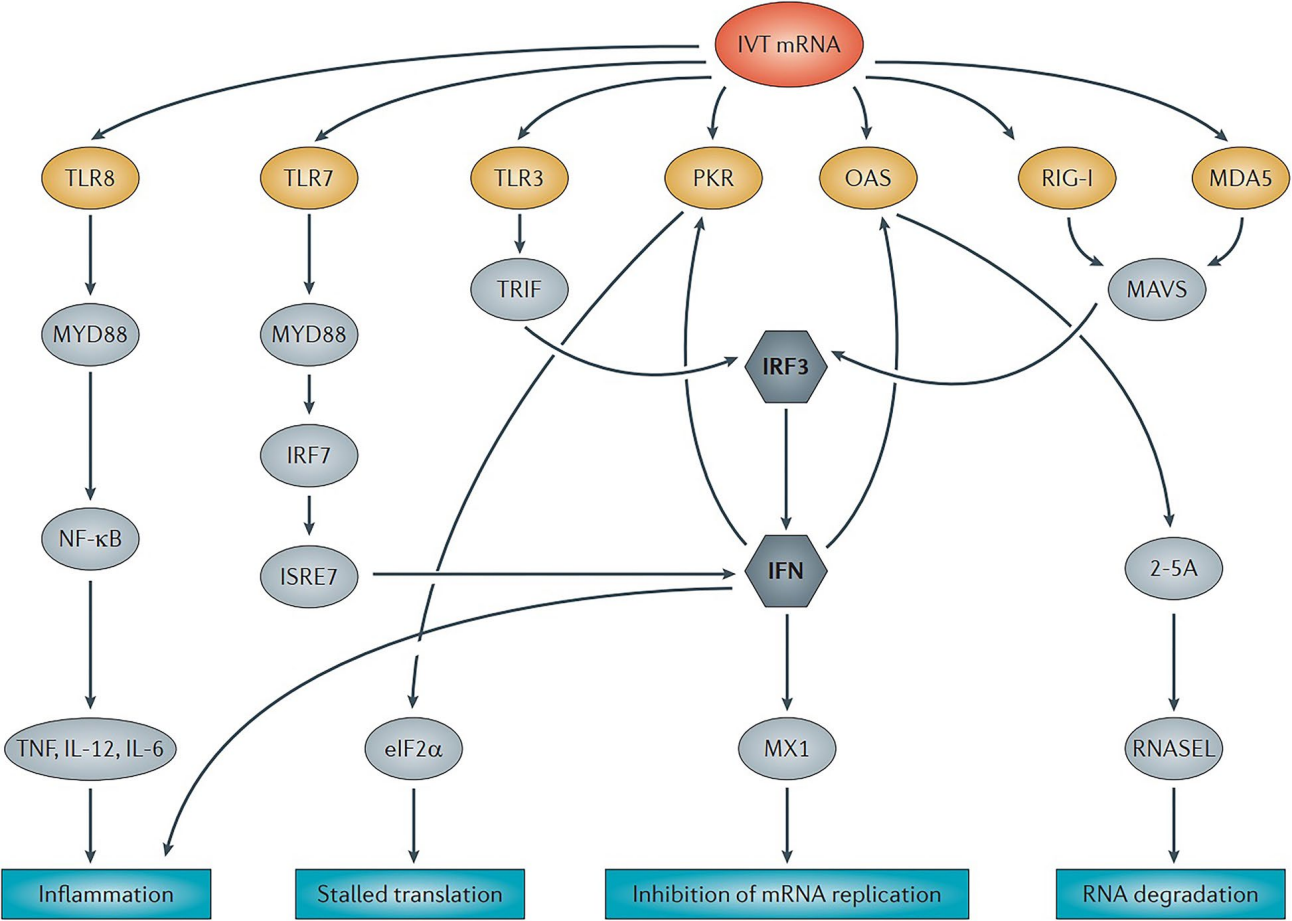
Inflammatory Reactions to Artificial mRNA. In vitro transcribed (IVT) mRNA is identified by a variety of endosomal innate immune receptors, including Toll-like receptors 3, 7, and 8 (TLR3, TLR7, TLR8), as well as cytoplasmic innate immune receptors like protein kinase RNA-activated (PKR), retinoic acid-inducible gene I protein (RIG-I), melanoma differentiation-associated protein 5 (MDA5), and 2′–5′-oligoadenylate synthase (OAS). These pathways signal to produce inflammation connected with type 1 interferon (IFN), tumor necrosis factor (TNF), interleukin-6 (IL-6), IL-12, and the initiation of various transcriptional programs. Collectively, these elements generate a pro-inflammatory environment conducive to triggering specific immune responses. Furthermore, downstream consequences such as eukaryotic translation initiation factor 2α (eIF2α) phosphorylation-induced translation slowdown, increased RNA degradation due to ribonuclease L (RNASEL) overexpression, and self-amplifying mRNA replication inhibition are significant for the pharmacokinetics and pharmacodynamics of IVT mRNA. Reprinted from [51] with permission from Springer Nature

These PRRs can sense different RNAs, including dsRNA and single stranded RNA (ssRNA), blocking mRNA translation as reviewed elsewhere [407]. The activation of multiple PRRs and production of type I IFN can be paradoxically beneficial or detrimental for anti-cancer immunotherapy [408]. It is potentially beneficial for vaccination since, in some cases, activation of type I IFN pathways drives APC activation and maturation, promotes antigen presentation, and elicits robust adaptive immune responses [405]. However, innate immune sensing of RNAs may be associated with inhibition of antigen expression, and thus dampen immune response [408]. Specifically, phage RNA polymerases produce unwanted dsRNA during IVT that can activate innate immunity via PKR, OAS, TLR-3, MDA-5 (one type of RIG-I like receptors) [409]. Once the PKR is activated, the eukaryotic initiation factor (eIF)-2 can be phosphorylated, blocking mRNA translation [410]. Moreover, the dsRNA activates RNase L upon binding to OAS, causing degradation of the exogenous RNAs [410]. Ultimately, binding of dsRNA with MDA-5 and TLR-3 can activate Type I IFN, eliciting several other genes that inhibit the translation of mRNA [411]. Besides the dsRNA impurities, improperly designed mRNA structure may also activate PRRs like MDA-5 and PKR, abolishing antigen expression [410]. Figure 10 illustrates the inflammatory reactions triggered by artificial in vitro transcribed (IVT) mRNA. This mRNA is recognized by several endosomal innate immune receptors, such as Toll-like receptors 3, 7, and 8 (TLR3, TLR7, TLR8), and cytoplasmic innate immune receptors, including protein kinase RNA-activated (PKR), retinoic acid-inducible gene I protein (RIG-I), melanoma differentiation-associated protein 5 (MDA5), and 2′–5′-oligoadenylate synthase (OAS). These receptor pathways then activate signaling processes that result in the production of inflammation-associated molecules like type 1 interferon (IFN), tumor necrosis factor (TNF), interleukin-6 (IL-6), and IL-12, initiating various transcriptional programs. The combined effect of these components creates a pro-inflammatory environment that facilitates the activation of specific immune responses. Additionally, downstream effects such as eukaryotic translation initiation factor 2α (eIF2α) phosphorylation-induced translation slowdown, increased RNA degradation due to ribonuclease L (RNASEL) overexpression, and self-amplifying mRNA replication inhibition play crucial roles in determining the pharmacokinetics and pharmacodynamics of IVT mRNA.

**Fig. 11:**
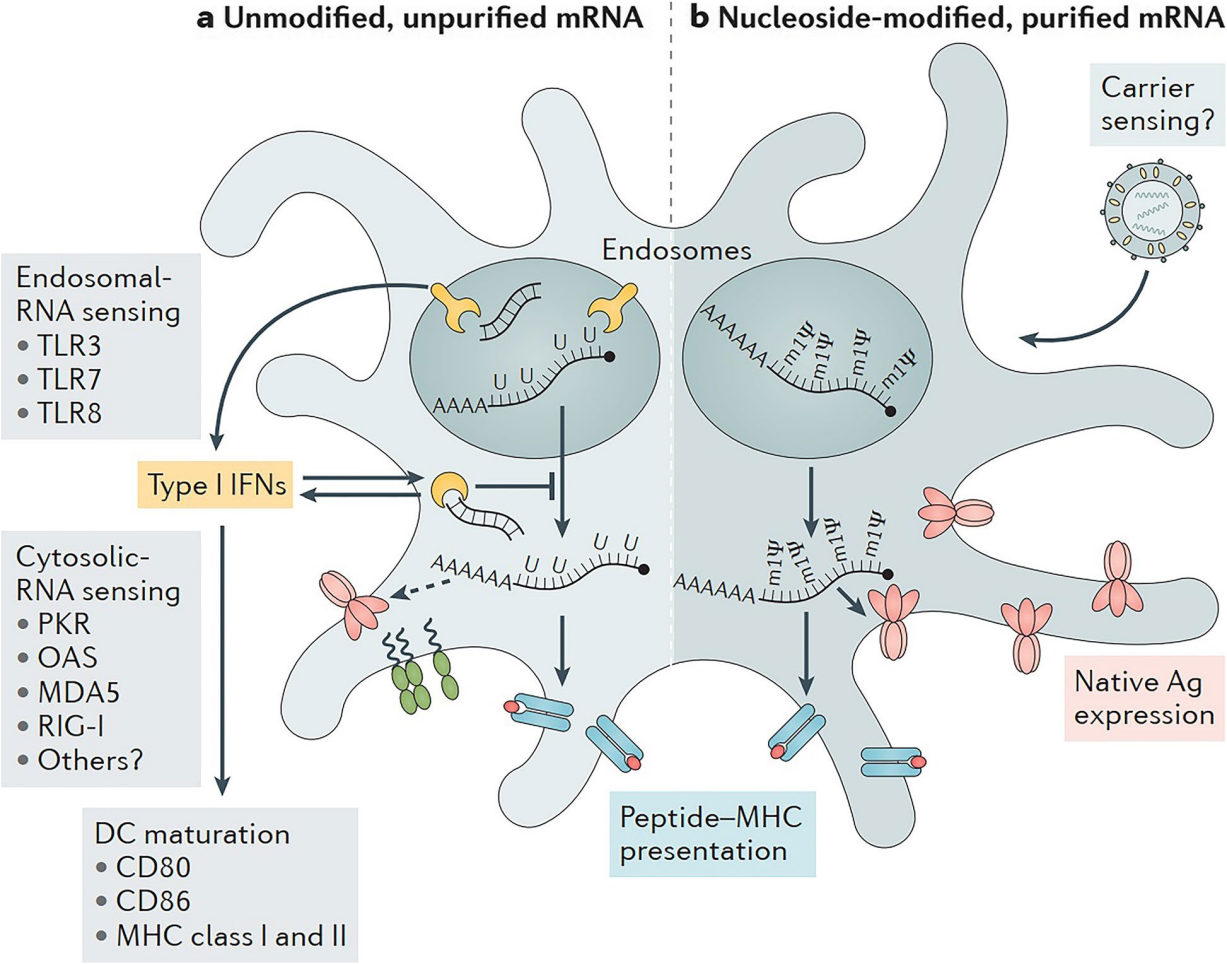
Natural immune detection of mRNA vaccines. DC recognizes two types of mRNA vaccines, with RNA sensors in yellow, antigens in red, DC maturation elements in green, and peptide-MHC complexes in light blue and red; a sample lipid nanoparticle carrier is depicted in the top right corner. A non-comprehensive list of major known RNA sensors responsible for identifying double-stranded and unaltered single-stranded RNAs is provided. Unaltered, unrefined (part a) and nucleoside-altered, FPLC-purified (part b) mRNAs are chosen to demonstrate two types of mRNA vaccines where known mRNA sensing mechanisms are either present or absent. The dotted arrow signifies diminished antigen expression. Ag stands for antigen; PKR for interferon-induced, double-stranded RNA-activated protein kinase; MDA5 for interferon-induced helicase C domain-containing protein 1 (also called IFIH1); IFN for interferon; m1Ψ for 1-methylpseudouridine; OAS for 2'-5'-oligoadenylate synthetase; and TLR for Toll-like receptor. Reprinted from [126] with permission from Springer Nature

The paradoxical impact of Type I IFNs activation is not only restricted to antigen expression, but also shown on CD^+^ 8 T cell activation [2]. The dual effect of Type I IFNs on CD8^+^ T cell immunity have been extensively reviewed elsewhere. In brief, the stimulatory or inhibitory actions of type I IFNs on CD^+^ 8 T cell activation is likely to be dependent on the timing and kinetics between activation of IFNAR signaling and TCR signaling, which may be further impacted by the routes of administration of mRNA cancer vaccines [412]. For instance, several studies have shown that type I IFNs can potentially promote CD8^+^ T cell response to systemic mRNA vaccination [413]. One hypothesis is that, intravenous (IV) delivery of mRNA (typically delivered by cationic lipoplex) is expressed in splenic DCs, where antigen expression and presentation take place simultaneously, with TCR signaling preceding or coinciding with IFNAR signaling [412]. In contrast, type I IFNs can potentially interfere with topical (ID or SC) mRNA vaccination where antigen expression happens locally in the injection site, but antigen presentation takes place in the secondary lymphoid organs, with IFNAR signaling precedes TCR signaling [240]. However, this IFNAR/TCR signaling theory is still debating, since other research groups have observed the opposite effects from local administration of mRNA vaccines [412]. Therefore, the purity of mRNA products, the modification of mRNA sequence, the design of delivery system and administration routes need to be tuned to properly active the innate immunity to initiate the adaptive immune response, simultaneously, averting the toxic overactivations that inhibit antigen protein expression and immune response [408]. Figure 11 illustrates the natural immune detection of mRNA vaccines by DCs.

## Self-amplifying mRNA vaccine, structure, advantages and deliveries

Another RNA vaccine platform that holds the promise to maximize the magnitude and length of antigen production is SAM [226]. SAMs are originated from positive-single stranded mRNA viruses, most commonly from alphaviruses such as Sindbis and Semliki-Forest viruses [62]. The structural protein encoding genes of respective alphavirus that enable the formation of infectious viral particles have been replaced with gene encoding the antigen(s) of interest, whereas the RNA replication machinery remains [414]. Specifically, the viral RNA-dependent RNA polymerase (known as replicase) and the non-structural proteins were retained to assemble into the multi-enzyme replicase complex to direct cytoplasmic amplification of RNA [415]. SAM can self-amplify over time (up to 2 months) and consequently inducing more potent and persistent immune responses owing to the integrity of the viral replication machinery [226]. The SAM platform precedes other nonreplicating mRNA vaccine platforms in that it allows for a huge amount of antigen production in an extended period of time from a remarkable low dose vaccination [416]. Some researchers reported that the IM injection of Sindbis virus-derived SAM led to a ten-fold increase in antigen expression ratio and eight-day extension of expression (from 2 to 10 days) than non-replicating mRNA [417, 418].

Early investigation of SAM is direct injection of SAM packaged into viral replication particles (VRP) [419]. VRPs are potent vaccines in mice, non-human primates and humans However, the replicated VRP structural proteins may induce non-specific immunogenicity and toxicity [420]. To decrease the infectious concern of viral components, a propagation-defective type of VRPs was generated [421]. The capsid and envelope proteins of the modified VRP are encoded in trans as defective helper constructs during production [419]. Only the RNA can further amplify after internalization, whereas other part of VRPs lack the ability to form infectious viral particles [422]. Nowadays, complete synthetic SAM produced after IVT can be directly used as RNA-based vaccine, removing the potential safety concerns of the viral components [423].

Since SAM is a huge and negatively charged molecule (~ 9500 nt), a delivery system is needed for its effective cellular uptake and protection from enzymatic degradation [416]. Over the past few years, substantial efforts have been made to identify a suitable delivery vehicle for IVT SAM [226]. Medium-length cationic polymer polyethylenimine (PEI) was adopted by some researchers to deliver the long SAM, from which they have shown that 64-fold less dose of SAM achieved the equivalent immunity to the non-replicating mRNA [416]. To decrease the potential toxicity from non-degradable cationic polymer, a bio-reducible, linear cationic polymer called “pABOL” was developed to deliver SAM [424]. Some researchers demonstrated pABOL enhanced protein expression via both IM and intradermal (ID) injection [424].

Some researchers presented a new vaccine platform based on self-amplifying RNA encapsulated in synthetic LNPs [425, 426]. The LNP platform protected SAM from enzymatic degradation, allowed for efficient gene delivery after IM injection [426]. Proof of concept was demonstrated in a model of respiratory syncytial virus (RSV) infection [427]. To further improve transgene expression and immunity of SAM vaccines, several approaches have been attempted: Some researchers have reported the co-administration of GM-CSF expressing RNA with SAM to improve the potency against a lethal influenza virus challenge in mice [226]. Moreover, some researchers evaluated different cationic lipid formulations including liposomes, LNPs, polymeric nanoparticles and emulsions to encapsulate rabies virus glycoprotein G (SAM-RVG), and noticed that DOTAP containing polymeric nanoparticles and LNPs were the most potent in triggering humoral and cellular immunity [428]. Lastly, SAM has been truncated into two transcripts (smaller in size) to address the concerns of inefficient delivery [429]. Some researchers have separated SAM encoding the antigen of interest from the replicase activity [62]. The replicase activity is provided in trans using a co-transfected RNA [62]. These two compartment SAM demonstrated 10–100-fold higher trans replicon expression than the whole-set counterpart [62]. The doses of influenza hemagglutinin antigen-encoding RNA in this platform were as low as 50 ng to induce neutralizing antibodies [430].

Clinical applications of SAM (delivered by VRPs and LNPs) in the prevention of infectious disease are promising, which have been extensively reviewed elsewhere. However, the applications of SAM in cancer vaccine are mainly limited to preclinical studies, with only two clinical trials ongoing using VRP delivered antigens against colorectal cancers [431]. The clinical and immunological benefits of SAM are still debating [431]. One major consideration that restricts SAM applications is the intrinsic PAMP natures, which makes it difficult to modulate the inflammatory profile, potentially limiting repeated dosing anti-tumor therapies [432].

## Delivery of mRNA cancer vaccine

Table 5 provides an extensive list of physical delivery methods for mRNA cancer vaccines, each with its own set of advantages and disadvantages. Some methods, such as electroporation, laser microporation, and magnetofection, offer high immunogenicity and efficacy, but may cause discomfort, require specialized equipment, or have limited depth of penetration. Non-invasive options like sonoporation, needle-free injection, and ultrasound-mediated delivery offer more comfortable experiences for patients but face their own limitations, such as penetration depth and equipment requirements. Biodegradable and hyaluronic acid-based nanoparticles are highly stable and offer targeted delivery, but require optimization for size and charge. Additionally, viral vector-mediated delivery provides high transfection efficiency, but raises concerns related to immune response and toxicity. Overall, each delivery method presents a unique balance of immunogenicity, efficacy, safety, and stability, emphasizing the need for continued research and development in this field.

**Table 5:** The different types of physical delivery methods for mRNA cancer vaccines

Delivery Method	Advantages	Disadvantages	Immunogenicity	Efficacy	Safety	Stability	Reference
Electroporation (EP)	Enhances uptake and expression of mRNA, can be used with a variety of tissues	Can cause discomfort and pain, may damage surrounding tissue or cells	High	High	Moderate	Unstable	[228]
Sonoporation	Non-invasive, highly specific targeting, can be used with a variety of tissues	Limited penetration depth, requires specialized equipment	Moderate	High	High	Stable	[433]
Laser microporation	Precise and controlled delivery, can be used with a variety of tissues	Limited scale-up capabilities, may require specialized equipment or expertise	High	High	High	Stable	[434]
Jet injection	Rapid and efficient delivery, can be used with a variety of tissues	May cause inflammation or tissue damage, limited depth of penetration	High	Moderate	Moderate	Stable	[435]
Needle-free injection	Non-invasive, easy to use	Limited depth of penetration, may cause tissue damage or inflammation	Moderate	Moderate	High	Stable	[436]
Gold particle bombardment	Can deliver to specific cells, easy to use	Limited scale-up capabilities, may cause tissue damage or inflammation	High	Moderate	Moderate	Stable	[437]
Magnetofection	Can deliver to specific cells or tissues, non-invasive	Limited depth of penetration, may require specialized equipment or expertise	High	High	High	Stable	[438]
Microprojection array	Precise and controlled delivery, can be used with a variety of tissues	Limited scale-up capabilities, may cause tissue damage or inflammation	High	High	High	Stable	[439]
Biodegradable nanoparticle	Targeted delivery, can be used with a variety of tissues	Requires optimization of size and charge for effective delivery, possible toxicity	High	High	High	Stable	[440]
Hyaluronic acid-based nanoparticle	Targeted delivery, can be used with a variety of tissues	Requires optimization of size and charge for effective delivery, limited scale-up capabilities	High	High	High	Stable	[143]
Cell-penetrating peptide	Efficient delivery to cells and tissues, low toxicity	Limited stability, may cause inflammation or tissue damage	High	Moderate	Moderate	Unstable	[441]
Hydrodynamic delivery	Rapid and efficient delivery, can be used with a variety of tissues	Limited depth of penetration, can cause tissue damage	High	High	High	Unstable	[442]
Ultrasound-mediated delivery	Non-invasive, can target specific cells and tissues	Limited depth of penetration, may require specialized equipment or expertise	Moderate	High	High	Stable	[160]
Viral vector-mediated delivery	High transfection efficiency, can be used with a variety of tissues	Limited scale-up capabilities, potential for immune response or toxicity	High	High	Low	Stable	[443]
Liposome-mediated delivery	Targeted delivery, low toxicity, easy to prepare	Limited scale-up capabilities, may cause inflammation or tissue damage	Moderate	Moderate	High	Stable	[198]
Calcium phosphate nanoparticle	Efficient delivery to cells and tissues, low toxicity	Limited scale-up capabilities, may cause inflammation or tissue damage	High	High	High	Stable	[444]
Magnet-assisted transfection (MAT)	Non-invasive, can target specific cells and tissues, low toxicity	Limited depth of penetration, requires specialized equipment	High	High	High	Stable	[445]
Photochemical internalization (PCI)	Targeted delivery, low toxicity, can enhance immunogenicity	Limited scale-up capabilities, requires specialized equipment	High	High	High	Stable	[446]
Cyclic dinucleotide-based delivery	Can enhance immune response, low toxicity	Limited scale-up capabilities, may cause inflammation or tissue damage	High	Moderate	Moderate	Stable	[447]
Nanoparticles with antigen presenting cells (APCs)	Efficient delivery to APCs, can enhance immune response	Requires optimization of size and charge for effective delivery, limited scale-up capabilities	High	High	High	Stable	[448]
Gene gun delivery	Precise and controlled delivery, can be used with a variety of tissues	Limited scale-up capabilities, may cause tissue damage or inflammation	High	High	High	Stable	[437]
Inorganic nanoparticle-mediated delivery	Targeted delivery, high loading capacity	Limited scale-up capabilities, requires optimization of size and charge for effective delivery	High	High	High	Stable	[449]
Topical delivery	Non-invasive, easy to use	Limited depth of penetration, may cause skin irritation or inflammation	Moderate	Moderate	High	Stable	[450]
Inhalation delivery	Non-invasive, can target specific cells and tissues in the respiratory tract	Limited depth of penetration, may cause respiratory irritation or inflammation	Moderate	Moderate	High	Stable	[451]
Acoustofluidic delivery	Non-invasive, can be used with a variety of tissues, can achieve high transfection efficiency	Requires specialized equipment, limited scale-up capabilities	High	High	High	Stable	[452]
In vivo electroporation	Efficient delivery, can be used with a variety of tissues	Can cause tissue damage, may require specialized equipment or expertise	High	High	High	Stable	[453]
Nanoparticle-laden microbubbles	Non-invasive, can achieve high transfection efficiency	Limited scale-up capabilities, requires specialized equipment	High	High	High	Stable	[454]
Supramolecular nanostructures	Targeted delivery, high loading capacity, can be used with a variety of tissues	Requires optimization of size and charge for effective delivery, limited scale-up capabilities	High	High	High	Stable	[455]
Magnetic field-guided delivery	Precise and controlled delivery, non-invasive	Limited depth of penetration, requires specialized equipment	High	High	High	Stable	[456]
Polymer-based microneedles	Non-invasive, easy to use	Limited depth of penetration, requires specialized equipment or expertise	Moderate	Moderate	High	Stable	[457]
Gold nanorod-mediated delivery	Targeted delivery, can achieve high transfection efficiency	Limited scale-up capabilities, requires optimization of size and charge for effective delivery	High	High	High	Stable	[458]
Dendritic cell-mediated delivery	Efficient delivery to dendritic cells, can enhance immune response	Requires specialized equipment or expertise, limited scale-up capabilities	High	High	High	Stable	[72]
Cell-penetrating peptides (CPPs)	Non-invasive, can be used with a variety of tissues	Limited scale-up capabilities, may have low transfection efficiency	Moderate	Moderate	High	Stable	[459]
Ultrasound-mediated delivery	Non-invasive, can achieve high transfection efficiency	Limited scale-up capabilities, requires specialized equipment	High	High	High	Stable	[460]
Bioreducible lipid nanoparticles	Targeted delivery, can enhance immune response, can achieve high transfection efficiency	Requires optimization of size and charge for effective delivery, limited scale-up capabilities	High	High	High	Stable	[424]
Surface-modified nanoparticles	Targeted delivery, can enhance immune response, can achieve high transfection efficiency	Requires optimization of size and charge for effective delivery, limited scale-up capabilities	High	High	High	Stable	[461]
Viral vectors (e.g. adenovirus, lentivirus)	Efficient delivery, high transfection efficiency	Safety concerns (e.g., integration, immunogenicity), limited scale-up capabilities, complex manufacturing	High	High	Variable	Stable	[344]
Cell-based delivery	Efficient delivery, can enhance immune response	Requires specialized equipment or expertise, may have limited scale-up capabilities, safety concerns (e.g., tumorigenicity)	High	High	Variable	Stable	[462]

Various viral, non-viral, and cell-based vehicles have been developed to increase the delivery efficiency of mRNA cancer vaccines [2]. Viral and cell-based vehicles have been extensively reviewed elsewhere and are not discussed in detail here. The comparison of these delivery systems is essential to identify the most effective and efficient way of delivering mRNA cancer vaccines. For example, some delivery systems may be more effective in delivering mRNA to certain types of cells, while others may be better at eliciting a specific immune response. By comparing the different delivery systems, researchers can choose the most suitable system for their mRNA cancer vaccine, taking into account the specific requirements of their study, such as the type of cancer being targeted, the desired immune response, and the mode of administration. Table 6 provides a comprehensive comparison of various mRNA cancer vaccine delivery systems, each with its own set of advantages, disadvantages, and factors affecting immunogenicity, efficacy, safety, and stability.

**Table 6:** The comparison of various mRNA cancer vaccine delivery systems

Delivery System	Advantages	Disadvantages	Immunogenicity	Efficacy	Safety	Stability	Reference
Lipid nanoparticles (LNPs)	High delivery efficiency, increased cellular uptake, and low toxicity	Prone to degradation, manufacturing costs, potential for immune response	Potentially high immunogenicity, excellent antigen expression	Vary depending on the specific cancer target and delivery method	Generally safe with limited adverse effects reported	Susceptible to degradation and require specialized storage conditions	[2]
Cationic polymers	Low cost, easy to produce, and highly customizable	Relatively low transfection efficiency and potential for toxicity	Lower immunogenicity than LNPs, but highly dependent on the specific polymer used	Highly dependent on cancer target and delivery method	Generally safe, but potential for toxicity and adverse immune responses	Vary depending on the specific polymer used	[121]
Peptide-based delivery	Highly customizable, and potential for targeted delivery	Low transfection efficiency and potential for toxicity	Potentially high immunogenicity, but dependent on the specific peptide used	Highly dependent on the cancer target and peptide delivery method	Generally safe, but potential for toxicity and adverse immune responses	Susceptible to degradation and require specialized storage conditions	[74]
In vivo electroporation (EP)	High transfection efficiency and targeted delivery	Limited depth of delivery, potential for pain or discomfort during injection, and potential for immune response	Potentially high immunogenicity, but highly dependent on the specific EP conditions and mRNA target	Highly dependent on cancer target and delivery method, but may have potential for both local and systemic effects	Generally safe, but potential for toxicity and adverse immune responses	Susceptible to degradation and require specialized storage conditions	[123]
Physical delivery methods (e.g., laser microporation, sonoporation)	Highly targeted delivery and relatively non-invasive compared to injection	Limited depth of delivery and potential for immune response	Highly dependent on the specific method and cancer target	Vary depending on the specific method and cancer target	Generally safe, but potential for toxicity and adverse immune responses	Vary depending on the specific method and cancer target	[111]
Gold nanoparticles	Targeted delivery and potential for imaging and therapeutic applications	May have potential for toxicity and immune response	May have lower immunogenicity compared to other delivery methods, but highly dependent on the specific conditions and mRNA target	Highly dependent on cancer target and delivery method, but may have potential for both local and systemic effects	Generally safe, but potential for toxicity and adverse immune responses	Vary depending on the specific method and cancer target	[133]
Biodegradable microspheres	Prolonged release and targeted delivery	Limited depth of delivery and potential for immune response	Potentially high immunogenicity, but highly dependent on the specific microsphere and mRNA target	Highly dependent on cancer target and delivery method	Generally safe, but potential for toxicity and adverse immune responses	Vary depending on the specific microsphere and cancer target	[161]
Cell-based vaccines	May have potential for enhanced efficacy and long-term immunity	May be challenging to produce and standardize, potential for immune response, and limited shelf-life	Highly dependent on the specific cell-based vaccine and mRNA target	Highly dependent on cancer target and delivery method	Generally safe, but potential for toxicity and adverse immune responses	Vary depending on the specific vaccine and cancer target	[123]
Proteins and peptides	High specificity and potential for targeted delivery	Low transfection efficiency and potential for toxicity	Potentially high immunogenicity, but highly dependent on the specific protein or peptide used	Highly dependent on cancer target and delivery method	Generally safe, but potential for toxicity and adverse immune responses	Vary depending on the specific protein or peptide and cancer target	[123]
Electrospray	High encapsulation efficiency, tunable particle size and morphology, and high antigenicity	Potential for mRNA degradation and low transfection efficiency	Potentially high immunogenicity, but dependent on the specific conditions and mRNA target	Highly dependent on cancer target and delivery method, but may have potential for both local and systemic effects	Generally safe, but potential for toxicity and adverse immune responses	Susceptible to degradation and require specialized storage conditions	[135]
RNA-lipoplexes	High delivery efficiency and low toxicity	Potentially low immunogenicity and low transfection efficiency	Potentially low immunogenicity and low transfection efficiency	Highly dependent on cancer target and delivery method	Generally safe, but potential for toxicity and adverse immune responses	Susceptible to degradation and require specialized storage conditions	[121]
mRNA-coated gold nanorods	Targeted delivery, potential for enhanced photothermal therapy, and reduced toxicity	Potentially low immunogenicity and low transfection efficiency	Potentially low immunogenicity and low transfection efficiency	Highly dependent on cancer target and delivery method	Generally safe, but potential for toxicity and adverse immune responses	Susceptible to degradation and require specialized storage conditions	[133]
Nanocarriers (e.g., dendrimers, cyclodextrins)	High encapsulation efficiency and low toxicity	May have potential for immune response, potential for mRNA degradation, and limited efficacy	Potentially high immunogenicity, but dependent on the specific carrier and mRNA target	Highly dependent on cancer target and delivery method	Generally safe, but potential for toxicity and adverse immune responses	Susceptible to degradation and require specialized storage conditions	[463]
Lipoplex-like nanoparticle	High encapsulation efficiency, low toxicity, and enhanced efficacy	May have potential for immune response and limited stability	Potentially high immunogenicity, but dependent on the specific nanoparticle and mRNA target	Highly dependent on cancer target and delivery method	Generally safe, but potential for toxicity and adverse immune responses	Susceptible to degradation and require specialized storage conditions	[121]
Lipid-coated gold nanorods	Targeted delivery, potential for enhanced photothermal therapy, and reduced toxicity	May have potential for immune response and limited stability	Potentially high immunogenicity, but dependent on the specific lipid and mRNA target	Highly dependent on cancer target and delivery method	Generally safe, but potential for toxicity and adverse immune responses	Susceptible to degradation and require specialized storage conditions	[464]
Polyplexes (e.g., polyethyleneimine, chitosan)	High transfection efficiency and low toxicity	May have potential for immune response and limited stability	Potentially high immunogenicity, but dependent on the specific polyplex and mRNA target	Highly dependent on cancer target and delivery method	Generally safe, but potential for toxicity and adverse immune responses	Susceptible to degradation and require specialized storage conditions	[448]
Virus-like particles (VLPs)	High immunogenicity and antigenicity, potential for multivalent display of antigens	May have potential for immune response and limited stability	Highly dependent on the specific VLP and mRNA target	Highly dependent on cancer target and delivery method	Generally safe, but potential for toxicity and adverse immune responses	Susceptible to degradation and require specialized storage conditions	[295]
Inorganic nanoparticles (e.g., calcium phosphate, zinc oxide)	High stability and biocompatibility, potential for targeted delivery	May have potential for immune response and limited efficacy	Potentially high immunogenicity, but dependent on the specific nanoparticle and mRNA target	Highly dependent on cancer target and delivery method	Generally safe, but potential for toxicity and adverse immune responses	Susceptible to degradation and require specialized storage conditions	[465]
Cell-penetrating peptides	High transfection efficiency and low toxicity	Limited potential for targeted delivery and potential for immune response	Potentially high immunogenicity, but dependent on the specific peptide and mRNA target	Highly dependent on cancer target and delivery method	Generally safe, but potential for toxicity and adverse immune responses	Susceptible to degradation and require specialized storage conditions	[451]
PEGylated lipid nanoparticles	Improved delivery efficiency and circulation time, reduced toxicity	Potential for immunogenicity and limited specificity	Potentially high immunogenicity, but dependent on the specific formulation and mRNA target	Highly dependent on cancer target and delivery method	Generally safe, but potential for toxicity and adverse immune responses	Susceptible to degradation and require specialized storage conditions	[466]
Magnetofection	Targeted delivery and potential for enhanced efficacy	Limited potential for immune response, potential for toxicity and adverse immune responses	Potentially low immunogenicity, but dependent on the specific method and mRNA target	Highly dependent on cancer target and delivery method	Generally safe, but potential for toxicity and adverse immune responses	Susceptible to degradation and require specialized storage conditions	[438]
mRNA-loaded extracellular vesicles	High stability and biocompatibility, potential for targeted delivery and sustained release	Limited potential for immune response, low transfection efficiency	Potentially low immunogenicity, but dependent on the specific vesicle and mRNA target	Highly dependent on cancer target and delivery method	Generally safe, but potential for toxicity and adverse immune responses	Susceptible to degradation and require specialized storage conditions	[467]
CRISPR-Cas9 system	Highly targeted delivery and potential for gene editing	Potential for off-target effects, limited specificity and efficiency	Potentially high immunogenicity, but dependent on the specific system and mRNA target	Highly dependent on cancer target and delivery method	Potential for toxicity and adverse immune responses	Susceptible to degradation and require specialized storage conditions	[468]
mRNA-protein conjugates	Enhanced immunogenicity and stability, potential for multivalent display of antigens	Limited potential for targeted delivery, low transfection efficiency	Potentially high immunogenicity, but dependent on the specific conjugate and mRNA target	Highly dependent on cancer target and delivery method	Generally safe, but potential for toxicity and adverse immune responses	Susceptible to degradation and require specialized storage conditions	[149]
Dendrimer-based delivery systems	High transfection efficiency, improved stability and biocompatibility, and potential for targeted delivery	Potential for immune response, limited specificity, and potential for toxicity and adverse immune responses	Potentially high immunogenicity, but dependent on the specific dendrimer and mRNA target	Highly dependent on cancer target and delivery method	Generally safe, but potential for toxicity and adverse immune responses	Susceptible to degradation and require specialized storage conditions	[91]
In-vivo electroporation	High transfection efficiency and potential for targeted delivery	Limited specificity, potential for immune response, and potential for toxicity and adverse immune responses	Potentially high immunogenicity, but dependent on the specific method and mRNA target	Highly dependent on cancer target and delivery method	Generally safe, but potential for toxicity and adverse immune responses	Susceptible to degradation and require specialized storage conditions	[453]
Intra-lymphatic injection	Potential for targeted delivery and enhanced immune response	Limited potential for systemic effects, limited data on safety and efficacy	Potentially high immunogenicity, but dependent on the specific method and mRNA target	Highly dependent on cancer target and delivery method	Generally safe, but potential for toxicity and adverse immune responses	Susceptible to degradation and require specialized storage conditions	[61]
Tumor-targeting aptamers	Potential for targeted delivery and reduced toxicity	Limited specificity and potential for immune response	Potentially high immunogenicity, but dependent on the specific aptamer and mRNA target	Highly dependent on cancer target and delivery method	Generally safe, but potential for toxicity and adverse immune responses	Susceptible to degradation and require specialized storage conditions	[469]
Non-viral nanocarriers (e.g., carbon nanotubes, mesoporous silica nanoparticles)	High stability and potential for targeted delivery	Limited data on safety and efficacy, potential for toxicity and adverse immune responses	Potentially high immunogenicity, but dependent on the specific nanocarrier and mRNA target	Highly dependent on cancer target and delivery method	Generally safe, but potential for toxicity and adverse immune responses	Susceptible to degradation and require specialized storage conditions	[470]

LNPs are known for their high delivery efficiency and low toxicity, while cationic polymers offer low cost and customizability. Peptide-based delivery systems boast a high level of customization and potential for targeted delivery. Other methods, such as in vivo electroporation, physical delivery methods like laser microporation and sonoporation, and the use of gold nanoparticles, provide targeted delivery with varying levels of immunogenicity and efficacy. Biodegradable microspheres, cell-based vaccines, and protein and peptide-based systems have shown promising results in certain cancer targets, while electrospray and RNA-lipoplexes provide high antigenicity and low toxicity, respectively. Novel methods like mRNA-coated gold nanorods and nanocarriers offer targeted delivery and reduced toxicity. Collectively, these delivery systems offer unique advantages and challenges in the fight against cancer, with their success largely dependent on the specific cancer target and delivery method employed.

Ex vivo DC mRNA allows for the loading of DCs with tumor-specific antigens but involves complex and costly procedures. In vivo naked mRNA is simple and easy to administer but has limited efficacy due to degradation in the body. Lipid nanoparticles and polymer-based delivery systems offer high efficiency of mRNA delivery, with the former being more efficient. Peptide-based delivery offers highly specific targeting of cancer cells, but limited clinical data and validation are available. Self-amplifying mRNA (SAM) requires lower doses for efficacy, while in vivo electroporation increases cellular uptake, reducing the need for delivery vehicles. Jet injection and hydrodynamic delivery are simple and easy to administer, but their efficacy is limited due to mRNA degradation. Physical delivery methods can achieve high mRNA delivery efficiency but may cause tissue damage or be limited by tissue barriers. In vitro transcription allows for large-scale production of mRNA but requires high cost and complex procedures. The safety and stability of these delivery systems are generally considered acceptable, but there are variations in efficacy and immunogenicity among them. Table 7 provides a comprehensive comparison of different delivery systems for mRNA cancer vaccines.

**Table 7:** Comparison of different delivery systems for mRNA cancer vaccines

Delivery System	Advantages	Disadvantages	Target Cells	Efficacy	Immunogenicity	Safety	Stability	Reference
Ex vivo DC mRNA	- Allows for the loading of DCs with tumor-specific antigens	- Complex and costly procedure	Dendritic cells	Promising in preclinical studies	Low immunogenicity	Generally considered safe	mRNA needs to be stored at low temperatures and protected from degradation	[471]
In vivo naked mRNA	- Simple and easy to administer	- Limited efficacy due to degradation in the body	Cancer cells and surrounding tissue	Low	Low to moderate	Generally considered safe	mRNA is unstable and easily degraded in the body	[472]
Lipid nanoparticles	- High efficiency of mRNA delivery	- Potential toxicity and immune response to the delivery vehicle	Cancer cells and surrounding tissue	High	Moderate to high	Generally considered safe	mRNA is stable and protected from degradation	[128]
Polymer-based delivery	- Biocompatible and biodegradable	- Less efficient compared to lipid nanoparticles	Cancer cells and surrounding tissue	Moderate	Moderate to high	Generally considered safe	mRNA is stable and protected from degradation	[443]
Peptide-based delivery	- Highly specific targeting of cancer cells	- Limited clinical data and validation	Cancer cells and surrounding tissue	Low to moderate	Low to moderate	Generally considered safe	mRNA is stable and protected from degradation	[473]
Self-amplifying mRNA (SAM)	- Requires lower doses of mRNA for efficacy	- Potentially higher toxicity due to longer persistence	Cancer cells and surrounding tissue	High	Moderate to high	Generally considered safe	SAM vaccines are more stable than non-SAM vaccines	[62]
In vivo electroporation	- Increases cellular uptake and reduces the need for delivery vehicles	- May cause pain, discomfort and tissue damage	Cancer cells and surrounding tissue	Moderate to high	Moderate to high	Generally considered safe	mRNA is unstable and easily degraded in the body	[135]
Jet injection	- Simple and easy to administer	- Limited efficacy due to degradation in the body	Cancer cells and surrounding tissue	Low	Low to moderate	Generally considered safe	mRNA is unstable and easily degraded in the body	[435]
Hydrodynamic delivery	- Rapid and efficient mRNA delivery	- Limited clinical data and validation	Liver cells and surrounding tissue	Moderate	Moderate	Generally considered safe	mRNA is unstable and easily degraded in the body	[474]
Physical delivery methods	- Can achieve high mRNA delivery efficiency	- May cause tissue damage or be limited by tissue barriers	Cancer cells and surrounding tissue	Varies	Varies	Generally considered safe	mRNA stability and degradation depend on the delivery method	[475]
In vitro transcription	- Allows for the large-scale production of mRNA	- High cost and complex procedures	Cancer cells and surrounding tissue	Promising in preclinical studies	Moderate to high	Generally considered safe	mRNA needs to be stored at low temperatures and protected from degradation	[170]

### Ionizable lipid nanoparticles-based mRNA delivery system

Ionizable lipid nanoparticles (LNPs) are a type of lipid-based delivery system used for the delivery of mRNA in mRNA vaccines. They are composed of a lipid bilayer that encapsulates the mRNA payload, protecting it from degradation and facilitating its delivery into target cells. The term "ionizable" refers to the presence of ionizable lipid components within the nanoparticle structure. The lipid component forms the core structure of the nanoparticle and provides stability. It is usually a mixture of different lipids, including ionizable cationic lipids, neutral lipids, and cholesterol. The ionizable cationic lipids play a crucial role in endosomal escape and efficient intracellular delivery of mRNA.

The mRNA encoding the desired antigen or therapeutic protein is encapsulated within the lipid bilayer of the nanoparticle. The mRNA is synthesized in vitro and then complexed with the lipid components to form the LNP. Polyethylene glycol (PEG) is often included in the LNP formulation to provide stability and prevent aggregation of nanoparticles. PEGylation also helps to prolong the circulation time of LNPs in the body, enhancing their chances of reaching the target cells. In some cases, LNPs can be modified with targeting ligands on their surface to facilitate specific binding to target cells or tissues. This modification enhances the specificity and efficiency of LNP delivery to the desired cells, improving vaccine efficacy.

The ionizable LNPs exploit the natural process of endocytosis, where cells engulf extracellular materials, to deliver the mRNA payload into the target cells. After the LNPs are taken up by cells, they are internalized into endosomes. The ionizable cationic lipids within the LNPs interact with the negatively charged endosomal membrane, leading to disruption of the endosomal structure and release of the mRNA payload into the cytoplasm. Once inside the cytoplasm, the mRNA is available for translation by the cellular machinery to produce the desired protein or antigen. The protein or antigen is then presented to the immune system, triggering an immune response and the production of specific antibodies or activated T cells. Ionizable lipid nanoparticles have gained significant attention in the field of mRNA vaccines due to their ability to efficiently deliver mRNA into cells, resulting in high protein expression levels and potent immune responses. They have been successfully used in the development of mRNA-based COVID-19 vaccines, such as the Pfizer-BioNTech and Moderna vaccines. The versatility and effectiveness of ionizable LNPs make them a promising tool for the delivery of mRNA-based therapeutics and cancer vaccines.

### Rationale for lipid nanoparticles to maximize deliver efficiency and immunogenicity

There are various types of lipid nanoparticles employed for mRNA vaccine delivery, each with their unique advantages and disadvantages. PEGylated lipids offer increased circulation time and reduced toxicity but suffer from poor transfection efficiency and manufacturing challenges. Cationic lipids boast good transfection efficiency and easy manufacturing but can be toxic and unstable. Neutral lipids provide high stability and low toxicity, while pH-sensitive lipids enable endosomal escape in acidic environments, but both have limited transfection efficiency. Ionizable lipids have high transfection efficiency and stability but are also associated with toxicity and off-target effects. Multi-component lipids, pro-nano liposomes, dual-function polymer-lipid nanoparticles, and targeted lipid nanoparticles offer enhanced stability, reduced toxicity, and improved transfection efficiency, albeit at the cost of complex manufacturing processes and potential off-target effects. Researchers continue to explore and develop these lipid nanoparticles to optimize the delivery of mRNA vaccines, aiming to maximize efficacy, safety, and stability while minimizing disadvantages and off-target effects.

LNPs are a type of delivery system that encapsulate the mRNA in a protective lipid coat to improve its stability and delivery efficiency (Table 8). LNPs, which were originally designed to deliver siRNAs, have been recently applied for the delivery of mRNA and present as the most clinical-translatable non-viral delivery vehicles [476]. LNPs are mainly composed of an ionizable amino-lipid-like molecule, a helper phospholipid, cholesterol, and lipid-anchored polyethylene glycol (PEG) [196]. The ionizable lipid is an amphipathic structure with a hydrophilic headgroup containing one or multiple ionizable amines, hydrocarbon chains capable of promoting self-assembly, and a linker that connects the headgroups with hydrocarbon chains [477]. The ionizable lipid is designed to acquire positive charges by protonation of the free amines at low pH for two main purposes: (1) during the preparation of LNPs, the positively charged lipids can facilitate encapsulation of the negatively charged mRNA via electrostatic interaction [121]; (2) in the acidic endosomal microenvironment upon intracellular delivery of LNPs, the positively charged lipid could interact with the ionic endosomal membrane, facilitating membrane fusion and destabilization, leading to release of mRNA from both LNPs and endosome [478]. At the physiological pH, the ionizable lipid remains neutral, improving stability and decreasing systemic toxicity [479]. Representative ionizable lipids include: Dlin-DMA, DLin-KC2-DMA, and DLin-MC3-DMA, which were synthesized based on rational design [480]; C12–200, and cKK-E12, which were screened by high throughput screenings of combinatorial libraries [275]; next-generation ionizable lipids, including DLin-MC3-DMA derivative L319 (Alnylam and AlCana Technologies), C12–200 and cKK-E12 derivatives (Anderson’s group), COVID-19 vaccine lipid ALC-0315 and SM-102, TT3 and biodegradable derivative FTT5 (Dong’s group), vitamin derived lipid ssPalmE and VcLNP, A9 (Acuitas), L5 (Moderna), A18 Lipid, ATX Lipid (LUNAR® composition, Arcturus) and LP01 (Intellia Therapeutics), which were mostly biodegradable [481]. Besides ionizable lipid(s), phospholipid (i.e. 1,2-dioleoyl-sn-glycero-3-phosphoethanolamine (DOPE), 1,2-distearoyl-sn-glycero-3-phosphocholine (DSPC)) and cholesterol are incorporated to improve lipid bilayer stability, aid membrane fusion and endosomal escape [482]. The lipid-anchored PEG is incorporated to decrease macrophage-mediated clearance. More importantly, lipid-anchored PEG helps prevent particle aggregation and improve storage stability [483]. For cancer vaccine delivery, LNPs should be designed to protect mRNA from extracellular RNase degradation, and to deliver mRNA encoding antigens specifically to APCs, so to facilitate efficient antigen presentation, whilst not comprise mRNA translation [484]. In addition, the lipid excipients used to deliver mRNA should be metabolizable and cleared rapidly, thus decreasing the potential systemic toxicity elicited from the vehicles and to allow for repeatable dosing [485]. Ionizable lipids play crucial roles in fulfilling all these purposes [479]. Current optimization of ionizable lipids have been focused on modulating the head group, linker and alkyl chains to adjust the acid dissociation constant (pKa), fusogenic properties, and metabolic behaviors [486]. Acid dissociation constant (pKa) of the ionizable amino group is strongly correlated with in vivo efficacy and immunogenicity of mRNA [487]. The optimal pKa range for IV delivery of siRNAs and mRNAs are between 6.2–6.5 as screened and confirmed. Whereas some researchers recently reported that the recommended range of lipid pKa was 6.6–6.9 for IM injection of mRNA to induce optimal immunogenicity [488]. To achieve the targeted pKa, the head group of the ionizable lipid usually contains at least one tertiary amine or two amino groups apart [489]. Examples include ethanolamine headgroup in L5 lipid (pKa 6.56), dimethylamine headgroup in DLin-MC3-DMA (pKa 6.44), and 2-ethylpiperidin headgroup in A18 (pH 6.6) [490]. Although the weakly acidic headgroup of the ionizable lipids is an important feature for the success of the LNP, it may also contribute to the instability of the nanoparticles [491]. According to the package insert, both Pfizer/BioNTech and Moderna COVID-19 vaccines must be stored at ultralow temperature and should be discarded after less than a day at room temperature [492]. One hypothesis for the instability nature of LNPs is that the ionizable lipids are neutral and oil-like at storage pH (usually neutral), and thus they may not tend to stay at the interface at ambient temperature [493]. Besides lipid pKa, the molecular shape of the lipid may also impact mRNA expression efficiency [494]. The hypothesis commonly acknowledged in the field is that the ionizable lipid should adopt a “cone” shape once protonated in acidic environments to facilitate endosomal escape [495]. In principle, the “cone shape” ionizable lipid, which contains lipid tails with larger cross-sectional areas than the lipid headgroups, could pair with the anionic endosomal membranes (i.e., phosphatidylserine) to form non-bilayer hexagonal H_II_ phases, resulting in fusion and disintegration of the endosomal membrane [495]. Multiple structure–activity evaluations from the high throughput lipid libraries demonstrate that incorporation of double bonds in hydrocarbon alky chains (especially cis-alkenyl group, e.g., linoleyl chains in Dlin-MC3 (KC2)-DMA) can alter the orientation of the alkyl chains, thereby enhancing the potentials to generate non-bilayer structure [496]. Linoleic acid-derived tails have been widely applied to build various ionizable or cationic lipids [497]. For instance, Some researchers have introduced linoleic chains to the cKK-E12 based polyamine core via a ring opening reaction [498]. The linoleic acid derivative OF-2 showed more than twice higher level of erythropoietin (EPO) expression than the cKK-E12 counterpart when IV injecting the EPO mRNA containing LNPs [499]. Increasing the degree of unsaturation (including alkynyl group) in the lipid tails can further enhance the fusogenicity of the lipid, and improve endosomal escape [495]. However, stability of LNPs may be compromised [499]. Replacing alkene group with ester bond can also maintain the lipid “core shape” and the fusogenicity [500]. Finally, the alkyl chain length may also be correlated with fusogenicity [500]. Some researchers evaluated lipids with alkyl chain length varying from C8 to C18, and showed that lipids with 12–14 carbon atoms in the tail were optimal for delivery [501]. Structural changes in the headgroup-linker region also affect the ionization behavior of the headgroup and the orientation of the alkyl chains [500]. However, safety is another index needs to be considered for chronic indications like cancer [502]. Unfortunately, improvements in delivery vehicle potency do not always result in an enlargement of the therapeutic outcome because of the reductions in tolerated dose levels [503]. Although the U.S. FDA approved DLin-MC3-DMA lipid is well tolerated in several clinical studies, repeat dosing some of the ionizable lipid containing LNPs have shown elevated cytokine levels and increased immunogenicity [309]. A persistent theme in the development of delivery vehicles is to incorporate biodegradable design features as means to improve biocompatibility and decrease systemic off-target toxicity [504]. Ester linkages are widely used for enhancing the biodegradability of biomaterials, as it can be hydrolyzed enzymatically by esterase or lipase in tissues and intracellular compartments [505]. Cleavage of an ester linkage within the hydrophobic chain will generate more hydrophilic by-products, carboxylic acid and alcohol that can be readily eliminated, or further metabolized by natural mechanisms [505]. In the same time, the sp^2^-carbon of the ester group helps the lipid maintain the “cone shape” to destabilize the endosomal membrane [506]. Moreover, the carboxylic acid containing derivative after hydrolysis are likely to reverse the positive charge in the amino head group, and facilitate the release of mRNA from the vehicle [507]. For instance, L319 (DLin-MC3-DMA derivative), LP-01 and lipid 5 are reported to be cleared from the liver rapidly (half-life < 6 h) as compared to DLin-MC3-DMA (half-life > 50 h) [508]. However, primary ester linkages added to the lipid tail are also vulnerable to the esterase/lipase in the systemic circulation, with the potential of cleavage before delivering mRNA intracellularly, thus leading to compromised potency [509]. A balance between delivery efficiency and pharmacokinetics are a complex correlation between number/type/location of the ester bond(s) in the hydrocarbon tails, the type and structure of the headgroup and linker [510]. Subtle change could tip the balance to one end. For instance, a combination of secondary and primary esters in the ethanolamine featured L5 lipid can maintain a satisfactory balance between expression potency and clearance [509]. Replacing the alcohol functionality with dimethylamine in the head group or moving the primary ester closer to the nitrogen group all introduce loss of delivery efficiency [511]. In some cases, introducing of ester bond can modulate the expression of protein in different cell types [512]. For example, OF-Deg-Lin induced protein expression selectively in the B cells of the spleen [513]. Therefore, rational design of biodegradable lipids could offer better control over clearance rate and expression selectivity [512]. In addition to chemical modifications of the ionizable lipids, formulation of LNPs were also optimized to potentiate antigen expression and adaptive immune response [491]. Some researchers have used design of experiment (DOE) to investigate the impact of ionizable lipid ratios, the type of helper lipids on the mRNA delivery efficiency [514]. The researchers found out that incorporation of DOPE as the helper lipid into cKK-E12 LNP could improve mRNA but not siRNA expression [515]. The same group later evaluated the impact of lipid length, PEG molecular weight and mole percentage of lipid-anchored PEG in LNPs on the distribution patterns of the encapsulated siRNA in vivo [516]. The highest liver distribution was observed when 0.75% of C18-PEG1000 were incorporated into C12–200 LNP formulations [516]. Some researchers have evaluated the mRNA expression using LNP containing combinations of different ionizable lipids, and indicated that combining a protein binding ionizable lipids with a lipid of high fusogenicity could potentiate mRNA expression [517]. Organ specificity can also be tuned by modifying the lipid formulations [518]. For instance, some researchers figured out that decreasing the ratio of cationic lipid to DOPE in the mRNA loaded lipoplex could shift mRNA expression from the lungs towards spleen [519]. Based on this rationale, they have developed lipoplexes that systemic delivered mRNA vaccine to splenic DCs [519].

**Table 8:** The different types of lipid nanoparticles used in mRNA vaccine delivery

Lipid Nanoparticle Type	Advantages	Disadvantages	Immunogenicity	Efficacy	Safety	Stability	Mechanism of Action	Reference
PEGylated lipids	Increased circulation time, reduced toxicity	Poor transfection efficiency, difficult to manufacture	Low	Moderate	High	Stable, but can be affected by PEG cleavage	Membrane fusion and endosomal escape	[520]
Cationic lipids	Good transfection efficiency, easy to manufacture	Can be toxic, poor stability	High	High	Moderate	Can be unstable in solution	Electrostatic interactions with the cell membrane and endosomal escape	[466]
Neutral lipids	High stability, low toxicity	Poor transfection efficiency	Low	Low	High	Stable	Endosomal escape	[480]
pH-sensitive lipids	Endosomal escape in acidic environments, increased stability	Limited transfection efficiency, potential for off-target effects	High	Moderate	Moderate	Stable, but can be affected by pH changes	Endosomal escape in acidic environments	[521]
Ionizable lipids	High transfection efficiency, good stability	Can be toxic, potential for off-target effects	High	High	Moderate	Stable	Endosomal escape via proton sponge effect	[2]
Neutral pH-responsive lipids	Good transfection efficiency, endosomal escape in mild acidic conditions	Limited stability, potential for off-target effects	High	Moderate	Moderate	Stable, but can be affected by pH changes	Endosomal escape in mildly acidic environments	[135]
Charge-reversal lipids	High transfection efficiency, good stability, increased target specificity	Potential for off-target effects, poor scalability	High	High	Moderate	Stable	Electrostatic interactions with the cell membrane and endosomal escape	[298]
Multi-component lipids	Increased stability, reduced toxicity, improved transfection efficiency	Complex manufacturing process, can be expensive	High	High	High	Stable	Membrane fusion and endosomal escape	[522]
PEG-phospholipid conjugates	Improved pharmacokinetics, increased stability	Poor transfection efficiency, limited control over PEG density	Low	Low	High	Stable	Membrane fusion and endosomal escape	[523]
Ionizable cationic lipids	High transfection efficiency, low toxicity	Can be unstable, potential for off-target effects	High	High	Moderate	Stable	Endosomal escape via proton sponge effect and electrostatic interactions with the cell membrane	[262]
Pro-nano liposomes	High stability, good transfection efficiency, biodegradable	Complex manufacturing process, potential for off-target effects	Moderate	High	Moderate	Stable	Endosomal escape and membrane fusion	[485]
Dual-function polymer-lipid nanoparticles	High stability, improved transfection efficiency, reduced toxicity	Complex manufacturing process, limited understanding of mechanism	High	High	High	Stable	Endosomal escape and electrostatic interactions with the cell membrane	[524]
SiRNA-lipid nanoparticles	Good transfection efficiency, high stability, reduced toxicity	Limited application to siRNA delivery only	Low	High	High	Stable	Endosomal escape and electrostatic interactions with the cell membrane	[341]
Metal ion-mediated self-assembled lipid nanoparticles	High stability, good transfection efficiency	Limited understanding of mechanism, potential for toxicity	Low	Moderate	Moderate	Stable	Endosomal escape and membrane fusion	[525]
Charge-altering releasable transporters (CARTs)	High transfection efficiency, improved target specificity, reduced toxicity	Limited understanding of mechanism, potential for off-target effects	High	High	High	Stable	Endosomal escape and membrane fusion	[298]
Self-assembling RNA nanoliposomes	High stability, good transfection efficiency, low toxicity	Limited understanding of mechanism, potential for off-target effects	Low	High	High	Stable	Endosomal escape and membrane fusion	[442]
Peptide amphiphile nanomicelles	High stability, reduced toxicity, improved transfection efficiency	Limited understanding of mechanism, potential for off-target effects	Low	High	High	Stable	Endosomal escape and membrane fusion	[526]
pH-sensitive cationic liposomes	High transfection efficiency, improved stability, endosomal escape in mildly acidic environments	Limited understanding of mechanism, potential for off-target effects	High	High	Moderate	Stable	Endosomal escape in mildly acidic environments	[527]
Phospholipid-PEG nanoparticles	Improved pharmacokinetics, reduced toxicity, good stability	Limited control over size and charge, limited transfection efficiency	Low	Low	High	Stable	Membrane fusion and endosomal escape	[523]
Superparamagnetic iron oxide nanoparticles	Good stability, transfection efficiency, potential for simultaneous imaging and targeting	Potential for off-target effects, limited understanding of mechanism	Low	Moderate	Moderate	Stable	Endosomal escape and membrane fusion	[528]
pH-sensitive liposomes	High transfection efficiency, improved stability, endosomal escape in mildly acidic environments	Limited understanding of mechanism, potential for off-target effects	High	High	Moderate	Stable	Endosomal escape in mildly acidic environments	[307]
Lipid-like nanoparticles	Good stability, transfection efficiency, reduced toxicity	Limited understanding of mechanism, potential for off-target effects	Low	High	High	Stable	Endosomal escape and membrane fusion	[341]
Cationic lipid-polymer hybrid nanoparticles	High stability, improved transfection efficiency, reduced toxicity	Complex manufacturing process, potential for off-target effects	High	High	Moderate	Stable	Endosomal escape and electrostatic interactions with the cell membrane	[190]
PEGylated lipid nanoparticles	Improved pharmacokinetics, reduced toxicity, good stability	Limited control over size and charge, limited transfection efficiency	Low	Low	High	Stable	Membrane fusion and endosomal escape	[473]
Targeted lipid nanoparticles	Improved target specificity, high stability, good transfection efficiency	Complex manufacturing process, potential for off-target effects	High	High	Moderate	Stable	Endosomal escape and specific receptor-mediated endocytosis	[529]
Unilamellar liposomes	High transfection efficiency, good stability, reduced toxicity	Limited control over size and charge, potential for off-target effects	Moderate	High	High	Stable	Endosomal escape and membrane fusion	[459]
Cationic lipid-nucleic acid nanoparticles	High transfection efficiency, improved stability, reduced toxicity	Complex manufacturing process, potential for off-target effects	High	High	Moderate	Stable	Endosomal escape and electrostatic interactions with the cell membrane	[530]
Silica nanoparticles	Good stability, transfection efficiency, potential for simultaneous imaging and targeting	Limited understanding of mechanism, potential for toxicity	Low	Moderate	Moderate	Stable	Endosomal escape and membrane fusion	[531]
Lipopolyplex nanoparticles	High transfection efficiency, improved stability, reduced toxicity	Complex manufacturing process, potential for off-target effects	High	High	Moderate	Stable	Endosomal escape and electrostatic interactions with the cell membrane	[532]
Calcium phosphate nanoparticles	Good stability, potential for simultaneous imaging and targeting	Limited transfection efficiency, potential for toxicity	Low	Moderate	Moderate	Stable	Endosomal escape and membrane fusion	[444]

### Mechanistic studies and additional functional modifications of LNPs

The rationales and mechanisms behind LNP internalization, endosomal escape and organ/cell-selective delivery have been widely investigated by multiple groups using either siRNA or mRNA as the delivered molecules [533]. In brief, apolipoprotein E(ApoE) or albumin-based receptor mediated endocytosis and non-specific micropinocytosis are two major mechanisms responsible for the update of mRNA/siRNA loaded LNPs [534]. To improve the specific delivery of LNPs to APCs, targeting ligand was further added to modify the LNPs [534]. For instance, mannose-cholesterol conjugates (MPn-CHs) was added to LNPs post formulation preparation through click reaction with the PEG units [423]. The mannose modified LNPs were shown to improve the uptake of the particles in DCs through mannose receptor CD206 [423].

Insufficient release of mRNA/siRNA from endosomal compartment has been considered as the predominant obstacle that limits the expression of mRNA/siRNA delivered by LNPs [535]. Intracellular trafficking of LNP loaded siRNA/mRNA have been visualized using electron microscope (EM), high-dynamic range live-cell imaging confocal, single-molecule fluorescence in situ hybridization (FISH), etc. [536]. By directly detecting colloidal-gold particles conjugated to siRNAs using EM, Some researchers demonstrated that only 1–2% of siRNA delivered by DLin-MC3-DMA LNPs could escape from the endosomes into cytosols [537]. Moreover, the cytosolic release of siRNA/mRNA only occurs during a narrow window of time when the LNPs reside in early matured endosomes, as reported by some researchers [232]. Ionizable lipids or helper lipids with increased fusogenicity have been incorporated into LNPs to improve the endosomal escape of mRNA/siRNAs [538]. For instance, Moderna L5 LNPs showed sixfold higher rate of endosomal escape as compared to the DLlin-MC3-DMA LNPs [539].

**Fig. 12:**
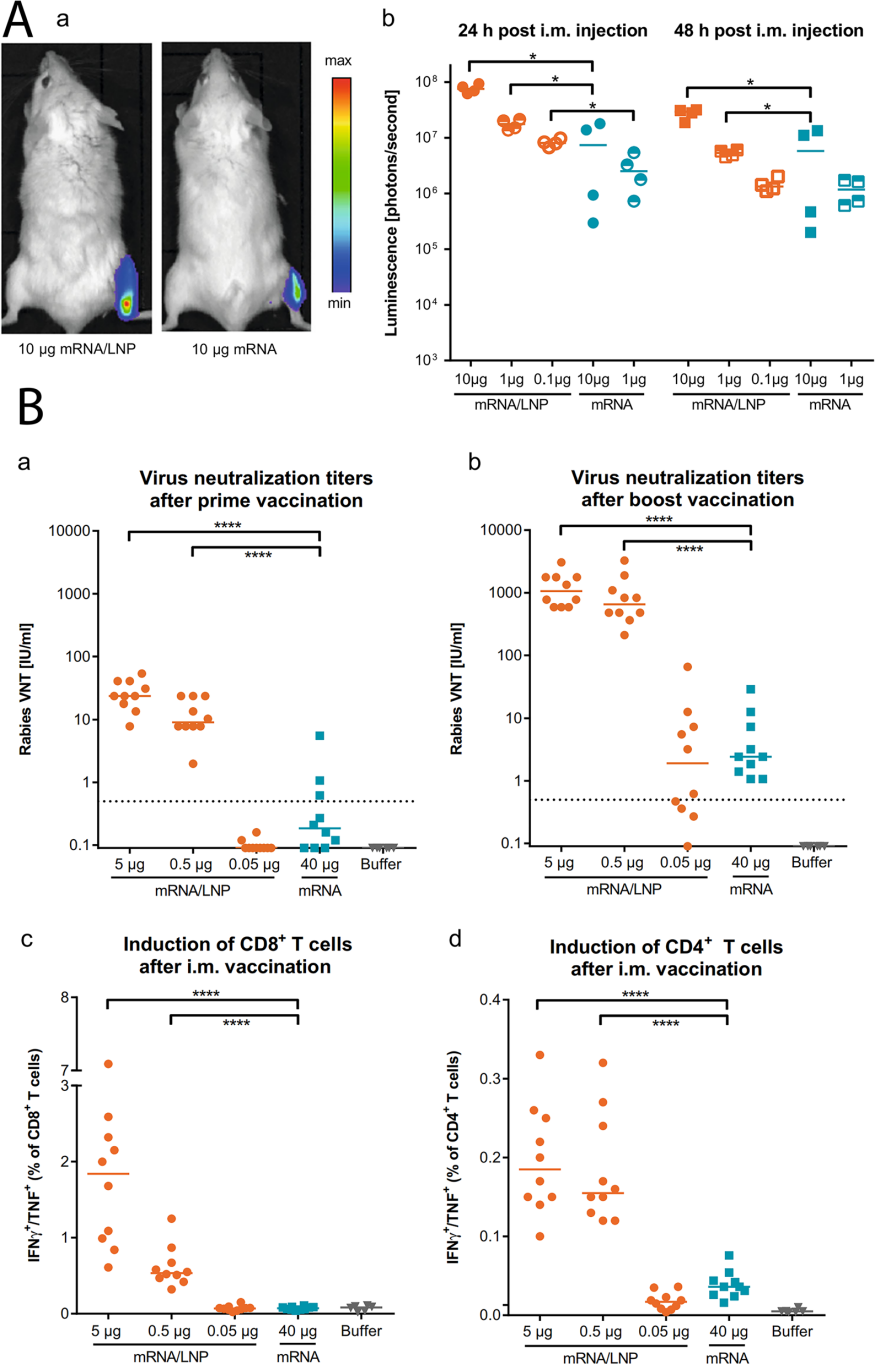
**A** LNP formulation boosts protein production in vivo following intramuscular administration. BALB/c mice (4 per group) received intramuscular injections of either non-formulated (mRNA) or LNP-formulated (mRNA/LNP) PpLuc mRNA. a) Luciferase expression was observed in vivo through optical imaging at 24- and 48-h post-injection. b) Luminescence was used to quantify luciferase expression. Data points from individual mice (represented as dots) and the median (depicted as solid lines) are shown for each group. **B** LNP-based mRNA vaccine triggers both humoral and cellular immune reactions in mice. BALB/c mice (10 per group) received intramuscular vaccinations on day 0 and day 21 using either non-formulated RABV-G mRNA (mRNA), LNP-encapsulated RABV-G mRNA (mRNA/LNP), or buffer. Rabies Virus Neutralization Tests (VNTs) were conducted on the serum three weeks post-initial vaccination (a) and two weeks post-boost vaccination (b). Two weeks after the boost vaccination, splenocytes were exposed to an overlapping peptide library encompassing the RABV-G protein. Antigen-specific, multifunctional (IFN-γ + /TNF +) CD8 + (c) and CD4 + (d) T cells were identified using intracellular cytokine staining. Data points represent individual mice, while the solid lines indicate median values for each group. The dashed line marks the generally accepted protective titer threshold of 0.5 IU/ml for rabies VNTs. Reprinted from [540] with permission from Springer Nature

The positive effect of lipid nanoparticle (LNP) formulation on protein production in vivo following intramuscular administration is demonstrated. Figure 12-A demonstrates the enhancement of protein production in vivo as a result of LNP formulation following intramuscular administration. In the study, BALB/c mice were divided into two groups of four, with one group receiving non-formulated PpLuc mRNA injections and the other receiving LNP-formulated PpLuc mRNA injections (mRNA/LNP). The luciferase expression, which serves as an indicator of protein production, was monitored through optical imaging at 24- and 48-h post-injection. The luminescence data was then used to quantify the expression levels. The results, represented by individual data points for each mouse and the median values shown as solid lines, clearly indicate that the LNP-formulated group exhibited significantly higher levels of luciferase expression compared to the non-formulated group, thus confirming the effectiveness of LNP formulation in boosting protein production in vivo.

The experiment involved BALB/c mice, divided into two groups of four. One group received intramuscular injections of non-formulated mRNA (mRNA), while the other group received LNP-formulated mRNA (mRNA/LNP) containing the PpLuc mRNA. Two time points, 24- and 48-h post-injection, were analyzed for luciferase expression using in vivo optical imaging (a). The luminescence was then quantified to measure the luciferase expression (b). The data points for individual mice are represented as dots, while the median for each group is shown as a solid line. Figure 12-A illustrates that LNP-formulated mRNA led to significantly enhanced protein production in comparison to non-formulated mRNA. The study involved three groups of BALB/c mice, each with 10 mice, which were vaccinated intramuscularly on day 0 and day 21 with either non-formulated RABV-G mRNA, LNP-encapsulated RABV-G mRNA (mRNA/LNP), or buffer. The figure presents Rabies Virus Neutralization Tests (VNTs) performed on the serum three weeks after the initial vaccination (a) and two weeks after the boost vaccination (b). Additionally, the figure shows antigen-specific, multifunctional (IFN-γ + /TNF +) CD8 + (c) and CD4 + (d) T cells identified using intracellular cytokine staining in splenocytes exposed to an overlapping peptide library encompassing the RABV-G protein, two weeks post-boost vaccination. Individual data points represent each mouse, while solid lines indicate the median values for each group. Figure 12-B demonstrates the efficacy of LNP-based mRNA vaccines in eliciting both humoral and cellular immune responses in mice. In the study, groups of BALB/c mice were given intramuscular vaccinations on day 0 and day 21, with either non-formulated RABV-G mRNA (mRNA), LNP-encapsulated RABV-G mRNA (mRNA/LNP), or buffer. Three weeks after the initial vaccination and two weeks after the boost vaccination, Rabies Virus Neutralization Tests (VNTs) were performed on the mice's serum to measure humoral immunity. Additionally, splenocytes from the mice were exposed to an overlapping peptide library covering the RABV-G protein, and antigen-specific, multifunctional (IFN-γ + /TNF +) CD8 + and CD4 + T cells were assessed through intracellular cytokine staining to gauge cellular immunity. Individual data points represent each mouse, while the solid lines indicate median values for each group. The dashed line signifies the generally accepted protective titer threshold of 0.5 IU/ml for rabies VNTs, highlighting the success of the LNP-based mRNA vaccine in triggering robust immune reactions in the test subjects.

Immunogenicity of the delivery materials were also evaluated and applied to boost immune response of the cancer vaccines [541]. Some researchers have developed a group of ionizable lipids containing cyclic amino head groups, isocyanide linker, and linoleic acid derived alkyl tails that provides adjuvant activities independent of the encapsulated mRNA [522]. The cyclic amino head and isocyanide linker of the lipids directly bound to STING (stimulator of interferon genes) protein and triggered the activation of Type I IFNs, leading to activation of humoral and cellular immune response [542].

### LNP mRNA vaccine from formulation to manufacturing

**Fig. 13:**
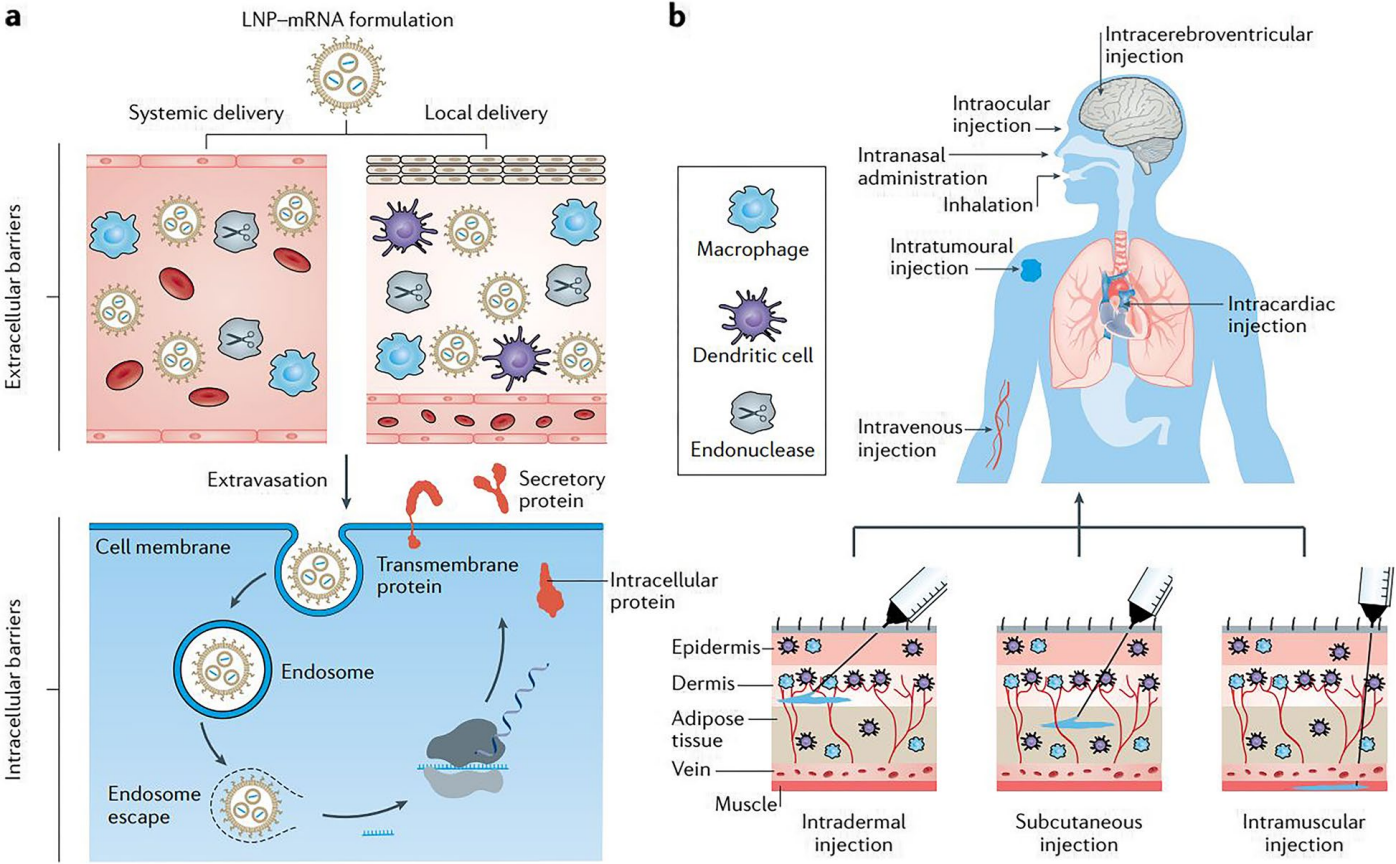
Obstacles in delivering and administering lipid nanoparticle-mRNA concoctions. **a** Natural hurdles faced by lipid nanoparticle-mRNA (LNP-mRNA) mixtures following systemic and localized distribution. **b** Methods of delivering LNP-mRNA compounds. Reprinted from [551] with permission from Springer Nature

Figure 13 illustrates the challenges and methods associated with delivering and administering lipid nanoparticle-mRNA (LNP-mRNA) concoctions. In part (Fig. 13-a), the figure highlights the natural obstacles faced by these mixtures after systemic and localized distribution. These hurdles include rapid clearance by the reticuloendothelial system, enzymatic degradation, immunogenicity, and endosomal entrapment, which impede the overall efficacy of LNP-mRNA therapies. Part (Fig. 13-b) of the figure outlines various delivery methods for LNP-mRNA compounds, such as intravenous, intramuscular, subcutaneous, and direct organ administration. Each method aims to overcome the natural barriers and maximize the therapeutic potential of LNP-mRNA treatments, enabling targeted gene expression and efficient cellular uptake for a range of clinical applications. The conventional benchtop formulation process for LNPs includes direct mixing, thin film, ethanol injection, which are usually labor intensive, lack of scalability and reproducibility [543]. More recently, great control was achieved over the mixing process when performed by T-junction mixing, microfluidic using microfluidic hydrodynamic focusing (MHF) or Staggered herringbone mixing (SHM) [544]. The rationales and advantages of each rapid mixing methods were summarized by some researchers elsewhere [545]. In brief, these chip-based microfluidic devices mix two laminar flows, the RNA-containing aqueous phase and the lipids-containing ethanol phase, through a confined microchannel equipped with chaotic mixers at a controlled speed, leading to rapid diffusion and self-assembly of mRNA-LNP at the interface [546]. High encapsulation efficiency (> 90%) and low polydispersity can be achieved by rapid laminar flow mixing [546]. The laminar flow rapid mixing method is scalable for GMP production of LNPs [547]. For instance, Precision NanoSystems team produced GMP microfluidic product of LNPs using the NanoAssemblr GMP system and a TrM (NxGen500) cartridge [548]. With the recent approval of two mRNA vaccines for prevention of COVID-19 from Pfizer/BioNTech and Moderna, rapid GMP manufacturing of COVID-19 vaccine (including mRNA and LNP manufacturing) are highly required [549]. For instance, BioNtech/Pfizer were committed to produce vaccines at 6 manufacturing sites to achieve 570 million doses for support dosing in 13 countries [550]. This further supports the feasibility of rapid production of mRNA vaccines to fulfill commercial requirement [59].

### Polymer-based mRNA delivery system

**Fig. 14:**
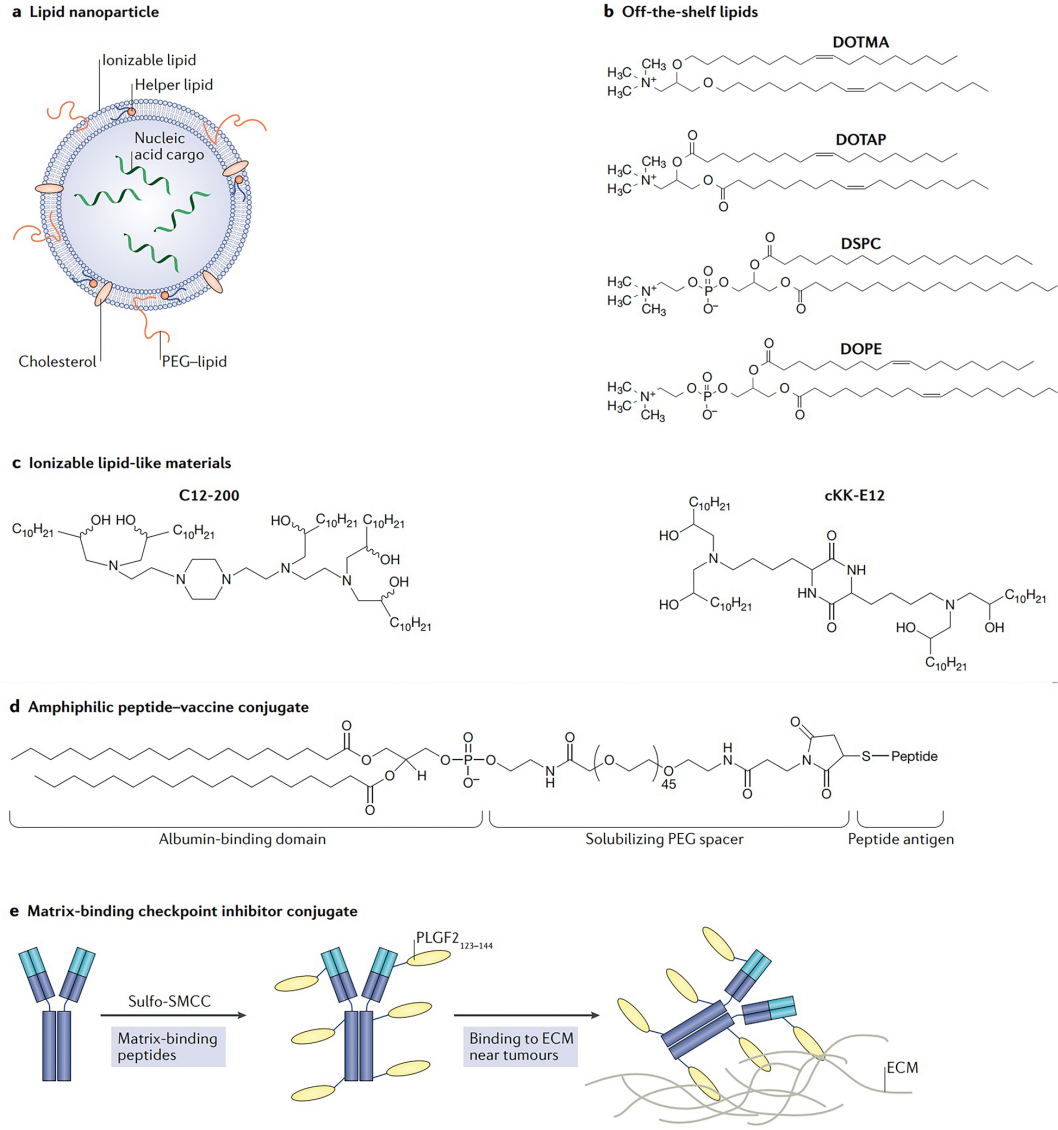
Nanoscale particles and complexes for cancer immunotherapy delivery systems. **a** Lipid nanoparticles usually comprise an ionizable lipid, a supporting lipid, cholesterol, and polyethylene glycol (PEG)-lipid. Nucleic acids are integrated into the nanoparticles' hydrophilic core. **b** The structures of ready-made lipids explored for nucleic acid delivery and, more recently, mRNA vaccines are depicted, including the structure of DOPE (1,2-dioleoyl-sn-glycero-3-phosphoethanolamine), a helper lipid that enhances lipid nanoparticle effectiveness. **c**) The structures of ionizable, lipid-like substances developed using combinatorial chemistry methods for better in vivo mRNA delivery and reduced toxicity. **d** The structure of an amphiphilic peptide-vaccine conjugate designed to attach to albumin in the bloodstream, enhancing lymph node delivery. **e** A matrix-binding checkpoint inhibitor conjugate with increased retention in the area surrounding the tumor to initiate an immune response. The checkpoint inhibitor is connected to a placental growth factor 2 (PLGF2) peptide using an amine-to-sulfhydryl linker. The PLGF2 peptide facilitates binding to proteins present in the extracellular matrix (ECM). Reprinted from [555] with permission from Springer Nature

Polyamines, dendrimers, biodegradable copolymers are commonly used polymer-based materials for mRNA delivery [552]. Polymer-based delivery systems tend to have lower purity due to high polydispersity, lower clearance rate due to large molecular weight, and worsen toxicity profile due to condensed charge density compared to synthetic LNPs, and they are not as clinically advanced for mRNA delivery as ionizable lipids [553]. Figure 14 illustrates various nanoscale particles and complexes utilized in cancer immunotherapy delivery systems To improve the tolerability and stability of the polymeric platforms, structural modifications, which include incorporating of lipid tails, hyperbranched groups and biodegradable moieties have been evaluated [554]. Polymer-based delivery systems are one of the methods used to deliver mRNA vaccines to cells (Table 9).

**Table 9:** The different types of polymer-based mRNA delivery systems

Polymer Type	Advantages	Disadvantages	Immunogenicity	Efficacy	Safety	Stability	Mechanism of Action	Reference
Polyethyleneimine (PEI)	High transfection efficiency	Cytotoxicity at high doses	Yes	Effective	Low	Good	Endosomal release	[556]
Poly(lactic-co-glycolic acid) (PLGA)	Biodegradable and biocompatible	Limited transfection efficiency	No	Moderate	High	Fair	Sustained release	[443]
Chitosan	Good biocompatibility and biodegradability	Limited transfection efficiency	Yes	Moderate	High	Fair	Endosomal release	[143]
Polyethyleneglycol (PEG)	Non-immunogenic and biocompatible	Poor transfection efficiency	No	Low	High	Good	Surface modification and cellular uptake	[557]
Poly(amidoamine) (PAA)	Good biocompatibility and efficient transfection	Cytotoxicity at high doses	Yes	Effective	Low	Good	Endosomal escape and cytoplasmic delivery	[558]
Poly(beta-amino esters) (PBAEs)	Good transfection efficiency and biocompatibility	Limited stability	Yes	Effective	Low	Fair	Endosomal release	[144]
Polyethyleneimine-polycaprolactone (PEI-PCL)	High transfection efficiency and stability	Potential cytotoxicity	Yes	Effective	Low	Good	Endosomal release	[556]
Poly(beta-amino ester)-polyethylene glycol (PBAE-PEG)	Good biocompatibility and efficient transfection	Limited stability	Yes	Effective	Low	Fair	Endosomal release and cytoplasmic delivery	[151]
Polyethylenimine-poly(lactic-co-glycolic acid) (PEI-PLGA)	High transfection efficiency and stability	Potential cytotoxicity and immunogenicity	Yes	Effective	Low	Good	Endosomal release	[559]
Dextran	Biocompatible and biodegradable	Limited transfection efficiency	No	Low	High	Fair	Sustained release	[560]
Polyethylenimine-polypropyleneimine (PEI-PPI)	High transfection efficiency and stability	Potential cytotoxicity and immunogenicity	Yes	Effective	Low	Good	Endosomal release	[561]
Poly-L-lysine (PLL)	Good transfection efficiency and biocompatibility	Limited stability and potential toxicity	Yes	Effective	Low	Fair	Endosomal release	[562]
Poly (amino acid) (PAA)	Biodegradable and biocompatible	Limited transfection efficiency and stability	No	Low	High	Fair	Sustained release	[563]
Polyhydroxyethylaspartamide (PHEA)	Good biocompatibility and stability	Limited transfection efficiency	No	Low	High	Good	Endosomal release and cytoplasmic delivery	[537]
Poly(N,N-dimethylaminoethyl methacrylate) (PDMAEMA)	High transfection efficiency and stability	Potential cytotoxicity and immunogenicity	Yes	Effective	Low	Good	Endosomal release	[564]
Poly(beta-thiopropionate) (PBT)	High transfection efficiency and stability	Limited data on biocompatibility	No	Effective	Low	Good	Endosomal release	[144]
Polypeptide	Biocompatible and biodegradable	Limited data on transfection efficiency	No	Low	High	Fair	Sustained release	[565]
Poly(glycidol) (PG)	Biocompatible and biodegradable	Limited data on transfection efficiency and stability	No	Low	High	Fair	Endosomal release and cytoplasmic delivery	[566]
Poly(amino acid)-polyethylene glycol (PAA-PEG)	Good biocompatibility and efficient transfection	Limited stability	Yes	Effective	Low	Fair	Endosomal release and cytoplasmic delivery	[151]
Polysaccharide	Biocompatible and biodegradable	Limited data on transfection efficiency and stability	No	Low	High	Fair	Sustained release	[542]
Poly(trimethylene carbonate) (PTMC)	Biodegradable and biocompatible	Limited data on transfection efficiency	No	Low	High	Fair	Sustained release	[567]
Poly(amidoamine)-polyethylene glycol (PAMAM-PEG)	High transfection efficiency and efficient endosomal escape	Potential cytotoxicity and immunogenicity	Yes	Effective	Low	Fair	Endosomal escape and cytoplasmic delivery	[568]
Poly(beta-amino ester)-polyethylene glycol (PBAE-PEG)	Good biocompatibility and efficient transfection	Limited stability	Yes	Effective	Low	Fair	Endosomal release and cytoplasmic delivery	[144]
Poly(2-(dimethylamino)ethyl methacrylate) (PDMA)	High transfection efficiency and efficient endosomal escape	Potential cytotoxicity and immunogenicity	Yes	Effective	Low	Good	Endosomal escape and cytoplasmic delivery	[564]
Poly(malic acid) (PMA)	Biodegradable and biocompatible	Limited transfection efficiency and stability	No	Low	High	Fair	Sustained release	[569]
Poly(propylene imine) dendrimers (PPI)	High transfection efficiency and efficient endosomal escape	Potential cytotoxicity and immunogenicity	Yes	Effective	Low	Good	Endosomal escape and cytoplasmic delivery	[570]
Poly(glutamic acid) (PGA)	Biodegradable and biocompatible	Limited transfection efficiency and stability	No	Low	High	Fair	Sustained release	[561]
Chitosan	Biodegradable and biocompatible	Limited transfection efficiency and stability	No	Low	High	Fair	Endosomal release	[143]
Poly(N-2-hydroxypropyl)methacrylamide (PHPMA)	Biodegradable and biocompatible	Limited transfection efficiency and stability	No	Low	High	Good	Endosomal release and cytoplasmic delivery	[571]
Poly(acrylic acid) (PAA)	Biocompatible and biodegradable	Limited data on transfection efficiency and stability	No	Low	High	Fair	Sustained release	[572]
Poly(glycolic acid) (PGA)	Biodegradable and biocompatible	Limited data on transfection efficiency and stability	No	Low	High	Fair	Sustained release	[561]
Polyurethane (PU)	Biocompatible and biodegradable	Limited data on transfection efficiency and stability	No	Low	High	Good	Endosomal release and cytoplasmic delivery	[573]
Poly(ethylene oxide) (PEO)	Good biocompatibility and efficient transfection	Limited stability	No	Effective	Low	Good	Endosomal release	[574]
Poly(N-vinylpyrrolidone) (PVP)	Good biocompatibility and efficient transfection	Limited stability	No	Effective	Low	Good	Endosomal release	[575]
Poly(beta-amino ester) (PBAE)	Efficient transfection and endosomal escape	Limited stability	Yes	Effective	Low	Fair	Endosomal escape and cytoplasmic delivery	[144]

Polymer-based mRNA delivery systems exhibit a range of characteristics, each with its own advantages and disadvantages. Polyethyleneimine (PEI) has high transfection efficiency but is cytotoxic at high doses. Poly(lactic-co-glycolic acid) (PLGA) is biodegradable and biocompatible but has limited transfection efficiency. Chitosan offers good biocompatibility and biodegradability but similarly suffers from limited transfection efficiency. Polyethyleneglycol (PEG) is non-immunogenic and biocompatible, though it has poor transfection efficiency. Various other polymers such as poly(amidoamine) (PAA), poly(beta-amino esters) (PBAEs), and poly-L-lysine (PLL) also have their unique sets of advantages and drawbacks, with some exhibiting high transfection efficiency but potential cytotoxicity or immunogenicity, while others have biodegradable and biocompatible properties but limited transfection efficiency or stability. The choice of polymer-based mRNA delivery system depends on the specific needs and requirements of the application, balancing factors such as immunogenicity, efficacy, safety, stability, and mechanism of action.

Polyethyleneimine (PEI) is one type of cationic polymer commonly used for nucleic acid delivery [562]. The commercial linear PEI derivative, jetPEI®, has already been used for mRNA in vivo/in vitro transfection [576]. A PEI formulation of SAM encoding the hemagglutinin antigens from influenza virus strains stimulated high antibody titer after IM vaccination in mice [577]. However, PEI is known with the severe systemic toxicity and low biodegradability due to the high charge density and molecular weight [578]. Low-molecular-weight PEI modified with fatty chains has been used for siRNA/mRNA delivery to reduce toxicity [578]. Polysaccharide and derivatives are another group of commonly used cationic polymers [579]. Some researchers have condensed SAM-encoding influenza virus hemagglutinin and nucleoprotein with chitosan, a commonly used polysaccharide excipient [580]. The researchers observed expression of antigens in DCs after SC injection of the particles [581]. Some researchers reported the use of polysaccharides derived from the microbial cell wall to form a flexible core–shell structure to encapsulate mRNA and promote DC activation in vivo [582]. Polyamidoamine (PAMAM) or polypropylenimine based dendrimer is another group of cationic polymer material used for mRNA delivery [530]. Some researchers developed fatty chain modified PAMAM dendrimers for delivery of siRNA systemically to lung endothelial [583, 584]. The same group later used the same delivery vehicle and delivered antigen-encoding SAMs [2]. The researchers showed that the single dose, adjuvant free IM delivered SAM protected mice from lethal challenge of Ebola, H1N1 influenza, Toxoplasma gondii, respectively [225]. Some researchers utilized a modified PAMAM dendrimers, PLGA and ceramide PEG to formulate polymer-lipid hybrid nanoparticles to deliver phosphate and tensin homolog mRNA in vivo [133]. In a later study, some researchers utilized the same vehicle to deliver OVA mRNA vaccine together with a fatty acid modified TLR7/8 agonist C16-R848, and showed the combination formulation could boost a strong antitumor immunogenicity [585]. Biodegradable polymers were developed to increase the clearance while decrease the charge induced toxicity of the delivery vehicles [586]. Poly (beta-amino) esters (PBAEs) are biodegradable polymers used for siRNA/mRNA delivery [587]. Some researchers co-formulated PBAEs with PEG-lipid to improve serum stability and increase mRNA delivery efficiency [588]. Besides adding lipid to the PBAE formulations, hyperbranched PBAEs were utilized to stabilized the formulation and to deliver mRNA to lung endothelium via IV injection, and to lung epithelium vial inhalation [495]. Other biodegradable polymers have been designed to achieve lower toxicity and selective delivery of mRNA to different organs [587]. Some researchers demonstrated that biodegradable amino polyesters (APEs), synthesized using ring-opening polymerization of various lactones, were capable of tissue-selective mRNA delivery [589]. Moreover, bio-reducible poly (CBA-co-4-amino-1-butanol) (pABOL), developed by some researchers were used to deliver haemagglutinin-(HA-) encoding SAM in mice [262]. Charge altering polymers have also been explored for mRNA vaccine delivery. Some researchers developed a library of charge-altering releasable transports (CARTs) that utilized poly(carbonate)-β-(α-amino ester)s [590]. CARTs undergo dynamic change from an ester to amide rearrangement. As a result, the cationic poly α amino ester backbone is gradually changed into neutral small molecules (diketopiperazine), providing a mechanism for release of mRNA, and avoiding tolerability issues associated with persistent polycations [590]. The CART polymers facilitated mRNA transfection into lymphocytes including T cells [591].

### Peptide-based mRNA delivery system

The cationic peptide, protamine has been used in many early studies for the delivery of mRNA vaccines [592]. Protamine spontaneously condenses mRNA through electrostatic interaction, protecting the encapsulated mRNA from degradation by extracellular RNases [593]. The protamine-mRNA complexes can also function as adjuvant, activating TLR7/8 to elicit Th-1 type immune response [594]. However, protamine-mRNA complexes alone showed suboptimal translation efficiency, which might be due to an excessively tight interaction between protamine and mRNA [595]. This concern has been solved by a two-compartment formulation, RNActive®, developed by CureVac AG [596]. The researchers combined protamine-mRNA complexes (50%) with naked antigen-coding mRNA(s) (50%). The protamine complexes act only as adjuvant, while the nucleoside modified mRNA acts as antigen producer [595]. RNActive® encapsulating TAAs-encoding mRNAs are currently being evaluated in several phase I/II clinical trials treating multiple solid tumors [116]. Most RNActive® vaccines are well tolerated and immunogenic in patients, some of them have shown moderate antitumor efficacy [116].

Cationic cell-penetrating peptides (CPPs) can complex with RNA [597]. Although their cell-uptake mechanisms are not fully understood, it is hypothesized that CPPs may facilitate clustering of the negative charged glycosaminoglycans on the cell surface, and trigger micropinocytosis [597]. RALA peptide is an amphipathic arginine-rich CPP with positively charged arginine residues on one end and neutral leucine residues on the other [598]. Researchers indicated that the peptide condensed mRNA complexes enabled mRNA delivery and expression in DCs, subsequently eliciting potent cytolytic T cell responses after ID injection of the ex-vivo loaded DCs [451]. Furthermore, D-amino acid-based truncated protamine was fused with a short CPP called Xentry [459]. This fusion peptide with combined positive and cell penetrating features was used to deliver a cystic-fibrosis transmembrane regulator (CFTR) mRNA into several human epithelial cells in vitro [459]. Some researchers used cholesterol-modified cationic peptide DP7 with transmembrane structure and immunoadjuvant function to modify the DOTAP liposomes [599]. This DOTAP/DP7-C liposomes efficiently transferred mRNA into different type of DCs in vitro. Subcutaneous injection of neoantigen-encoding mRNA loaded in DOTAP/D7-C liposomes significantly inhibited the growth of LL2 [599]. Similarly, an alpha-helical cationic CPP “KALA” was combined with the vitamin E-scaffold (ssPalmE)-LNP to achieve higher protein expression and increased proinflammatory cytokines secretion in DCs, functioning as a potent ex vivo DCs-based RNA vaccine platform [600]. Besides positive charged CPP, negative charged GALA peptide has been used as a targeting ligand, that click onto LNPs/polyplexes to improve the cell penetration of mRNAs [529].

### Other formulations used in mRNA delivery

In additional to ionizable lipid composed LNP system, cationic lipid composed liposomes, lipoplexes and cationic emulsions (CNE) are the very first generation of carriers used for mRNA delivery both preclinically and in clinical trials [121]. DOTMA (1,2-di-O-octadecenyl-3-trimethylammonium propane) and DOTAP (1,2-dioleoyl-3-trimethylammonium-propane) are two most widely used cationic lipids [601]. These lipids remain positively charged at all physiological pH, and can easily condense anionic mRNA [601]. A combination of DOTMA/DOTAP with fusogenic helper lipid DOPE to form lipoplexes have been used by BioNTech in their Lipo-MERIT cancer vaccine platform [601]. The ratio of cationic lipid and DOPE can be tuned to selectively target splenic APCs for mRNA vaccine delivery [262]. Promising therapeutic outcome has been seen in several ongoing clinical trials treating metastatic melanoma (summarized in later section).

In addition, DOTAP containing cationic CNE, which is derived from the Novartis’s first FDA approval CNE MF-59 have been used for mRNA delivery [171]. For instance, cationic CNE was used by some researchers to encapsulate SAM [602]. The CNE was prepared by mixing an aqueous phase containing buffer and Tween 80 with an oil phase containing Sorbian tioleate (Span 80), DOTAP, and oil squalene [603]. The researchers have shown that the protein expression of mRNA delivered by the CNE through IM administration was similar to a viral vector [602]. The mRNA CNE vaccine was well tolerated and immunogenic in a variety of models [121]. DOTAP containing liposomes were also used as a shell for encapsulating mRNA in core–shell structures [171]. For instance, some researchers has developed lipid/calcium/phosphate (LCP) system using calcium phosphate as the core to condense mRNA, and PEGylated DOTAP/DOPE liposome as the shell [604]. The researchers delivered MUC-1 (TAA of the triple negative breast cancer) mRNA to 4 T1 breast cancer bearing mice, and observed potent antigen-specific T cell activation and improved antitumor efficacy [605]. Moreover, Lipid-Polymer-RNA lipopolyplexes (LPR), functionalized with a tri-antenna of α-d-mannopyranoside (triMN-LPR) can specifically bind to human and mouse DC, provide high induction of a local inflammatory response after ID injection [605]. Another LPR system consisting of poly (β-amino ester) polymer/mRNA core encapsulated into a 1,2-dioleoyl-sn-glycero-3-ethylphosphocholine/1,2-dioleoyl-sn-glycero-3-phosphatidyl-ethanolamine/1,2-distearoyl-sn-glycero-3-phosphoethanolamine-N-[amino(polyethyleneglycol)-2000] (DOPC/DOPE/DSPE-PEG) lipid shell was developed by some researchers to deliver mRNA into DC through micropinocytosis [60, 606]. Results shown that the LPR induced potent antigen response [607]. A similar LPR platform is currently being evaluated in phase I clinical trial carrying mRNA encoding neoantigens to treat metastatic melanoma by Stemirna Therapeutics [607]. In additional to non-viral deliver system, naked mRNA has been directly injected ID or intranodal as anti-cancer vaccine or ex vivo loaded into DCs for cancer vaccinations [608]. The naked mRNA vaccines and DC-based mRNA vaccines have been widely evaluated in clinical trials with some optimistic therapeutic outcome for cancer treatment [72]. However, the strategies are either suffered from insufficient antigen expression, complicated in vitro processing or batch to batch variabilities [72].

## Injection routes mRNA cancer vaccines

Local injections, including IM, SC, ID, are the commonly used injection routes for mRNA cancer vaccines [609]. Representative examples include: IM injection of PAMAM loaded OVA mRNA for melanoma treatment in mice, Moderna LNPs optimized for IM injection of mRNA vaccines, SC injection of peptide modified DOTAP liposomes, SC injection of LNPs with optimized lipid compositions and lipid structures for antitumor vaccinations, i.d. injection of LPR to boost anti-cancer immunity in multiple mouse models [610]. Table 10 outlines the different mRNA cancer vaccine injection routes, each with its unique advantages and disadvantages.

**Table 10:** The different mRNA cancer vaccine injection routes

Injection Route	Advantages	Disadvantages	Immunogenicity	Efficacy	Safety	Stability	Reference
Intramuscular (IM)	Simple to administer, induces systemic immune response	May require multiple doses, limited local response	Moderate to high	Effective in some cancers, variable results	Generally safe, mild side effects	Stable at refrigeration temperature, needs to be protected from light	[611]
Subcutaneous (SC)	Simple to administer, induces systemic immune response	May require multiple doses, limited local response	Moderate to high	Effective in some cancers, variable results	Generally safe, mild side effects	Stable at refrigeration temperature, needs to be protected from light	[56]
Intradermal (ID)	Induces strong local and systemic immune response, potential for dose-sparing	Limited quantity of mRNA can be delivered, may require specialized equipment	High	Effective in some cancers, variable results	Generally safe, mild side effects	Stable at refrigeration temperature, needs to be protected from light	[38]
Intravenous (IV)	Induces systemic immune response	Requires high dosage and specialized equipment, potential for non-specific immune response	Low to moderate	Effective in some cancers, variable results	Generally safe, mild to moderate side effects	Stable at ultra-low temperatures, short half-life in circulation	[56]
Intra-tumoral (IT)	Direct delivery to tumor site, potential for targeted response	May require specialized equipment, limited systemic immune response	High	Effective in some cancers, variable results	Generally safe, mild to moderate side effects	Stable at refrigeration temperature, needs to be protected from light	[56]

Intramuscular (IM) and subcutaneous (SC) injections are simple to administer and induce a systemic immune response, but they may require multiple doses and have limited local response. Both methods show moderate to high immunogenicity, efficacy in some cancers, and general safety with mild side effects. Intradermal (ID) injections induce a strong local and systemic immune response with potential for dose-sparing, although they may require specialized equipment. Intravenous (IV) injections can also induce systemic immune response, but they require high dosages and specialized equipment, which may lead to non-specific immune responses. Lastly, intra-tumoral (IT) injections allow direct delivery to the tumor site and have the potential for targeted response, but may also require specialized equipment and offer limited systemic immune response. All injection routes, except for intravenous, are stable at refrigeration temperature and need protection from light, while intravenous injections are stable at ultra-low temperatures and have a short half-life in circulation.

Intramuscular administration is often preferred due to the flexibility of injection volume, the ease of dosing and the lack of safety concern, with limited risk for adverse reactions at the site of injection, However, vaccine delivered to the skin as a highly immunocompetent site has long been considered a strategy to augment vaccine response [612]. Some researchers have investigated the impact of vaccination route (mainly IM and SC) on antigen trafficking and immune response in Rhesus Macaques using fluorescently labeled HIV-1 envelope glycoprotein trimers displayed on liposomes [613]. The researchers found that both SC and IM routes induced efficient immune cell infiltration, activation and antigen uptakes [613]. Though the immunogenicity is tightly restricted to the injection site, and antigen also transported to different lymph nodes depending on route, these early differences failed to convert into significant differences in the magnitude or quality of antigen-specific immune response [614]. Despite this, the expression level and inherent innate immunity of mRNA might be influenced by the routes of administration, subsequently leading to different intensity of immune response [614]. Using the most translatable carrier LNPs as an example, some researchers have evaluated the expression kinetics of nucleoside modified mRNA in mice through various routes of administration [615]. Their findings demonstrated that IM and ID delivery of mRNA LNPs resulted in the longest duration of mRNA translation (half-life > 20 h) followed by SC (half-life ~ 15 h) and IV (half-life ~ 7 h) [615]. Whereas, SC and IM showed higher protein expression level as compared to ID The differences in magnitude and length of protein expression from different routes of administration may directly impact the intensity of immunogenicity, which required detailed evaluations in the future studies [616]. As covered in Sect. "Basic mRNA vaccine pharmacology", the kinetics between TCR activation and IFN signaling can also be dependent on the route of mRNA administration, ultimately impacting the potency of T cell activation [617]. Based on this perspective, systemic mRNA vaccination through IV injection is more likely to promote a favorable CD8^+^ T cell response and circumvent the detrimental impact of mRNA inherent innate immunity [57]. As a result, vaccination through IV injection has been used by several researchers and companies [57]. However, one needs to concern about the potential systemic toxicity generated from IV vaccination [618]. Until now, SC and IM injections are still the two major injection routes for mRNA cancer vaccination in clinical trials, due to their less invasive nature; however, other injection routes, including intranasal, and intranodal have been widely studied for mRNA vaccine delivery [618].

## Clinical overview of mRNA cancer vaccines

Transfection of mRNA into DCs for adoptive transfer was the first mRNA based therapeutic cancer vaccine entering clinical trial [72]. Although DC-based mRNA vaccine therapeutics still account for majority of mRNA cancer vaccines in clinical trials, IVT mRNA-based immunotherapies delivered by non-viral vectors are extensively explored recently as a result of the promising antitumor outcomes collected from preclinical studies, with CureVac, BioNTech and Moderna as pioneers in the campaign [619].

A group of IVT mRNA-based immunotherapies investigated in clinical trials are mRNAs encoding immunostimulants which are injected intratumorally or intranodal to modify the suppressive tumor microenvironment [620]. These immunostimulants are not considered as cancer vaccines, but are usually co-administered with cancer vaccines or other immunotherapeutic agents (e.g. checkpoint blockade modulators) and act as adjuvants to augment humoral and cellular response [621].

Multiple IVT mRNA-based cancer vaccines are currently tested in clinical trials, either encoding personalized neoantigens, or a cocktail of TAAs [622]. Deliver systems for these mRNA-based cancer vaccines include lipid polyplexes, CNEs, LNPs or protamine [2]. Local injection, such as IM, SC and ID are major administration routes for mRNA vaccines in the clinical studies, whereas the BioNTech product, Lipid-MERIT (DOTAP (or DOTMA)/DOPE lipoplex as deliver system) is vaccinated intravenously [623]. As discussed earlier, the ratio between DOTAP and DOPE can be optimized to allow specific delivery of mRNA to splenic APCs, and induce potent antigen-specific response [624].mRNA vaccines have been applied to treat aggressive, less accessible and metastatic solid tumors, including non-small cell lung cancers (NSCLC), colorectal carcinoma (CRC), melanoma, etc. [625]. For early proof of concept studies, mRNA vaccine has also been tested in treating glioblastoma [626]. In most clinical trials, mRNA cancer vaccines are further combined with checkpoint modulators or cytokine cocktails to augment antitumor efficacy [627]. Although SAMs are an appealing alternative to mRNA-based vaccine due to their inherent self-amplifying property, clinical investigation for cancer applications is only limited to early evaluation of VRPs [2]. With the recent advancing of, specifically the discovery of neoantigens, development of personalized vaccines and checkpoint blockade modulators, numerous improvements have been done to demonstrate the viability of mRNA vaccines to combat cancer [628]. In this section, we will discuss mRNA applications as immunostimulants and cancer vaccines, compare the delivery of mRNA encoding TAAs and neoantigens, as well as discuss the advantages of personalized vaccines and combination immunotherapies with checkpoint blockade modulators.mRNA-based COVID-19 vaccine developed by Pfizer and BioNTech has undergone extensive clinical trials. The Phase 3 trial involved over 43,000 participants and demonstrated an efficacy of approximately 95% in preventing symptomatic COVID-19 infection. The trial results were published in the New England Journal of Medicine in December 2020 [629]. Another mRNA-based COVID-19 vaccine developed by Moderna also underwent rigorous clinical trials. In a Phase 3 trial involving approximately 30,000 participants, the vaccine demonstrated an efficacy of around 94.1% against symptomatic COVID-19 infection. The trial results were published in the New England Journal of Medicine in December 2020 [630]. mRNA-based immunotherapies have also shown promise in the field of cancer treatment. For example, a study published in Nature in 2017 reported the results of a Phase 1 clinical trial of an mRNA-based personalized cancer vaccine. The vaccine was tested in patients with melanoma, and it showed encouraging results in terms of inducing immune responses and promoting tumor regression [631]. An mRNA-based influenza vaccine has also been the subject of clinical trials. A study published in The New England Journal of Medicine in 2019 reported the results of a Phase 1 trial of an mRNA-based flu vaccine. The trial showed that the vaccine induced a robust immune response and was well-tolerated by participants [632]. Researchers have also explored the potential of mRNA-based vaccines for HIV. A study published in Science in 2020 described a Phase 1 trial of an mRNA vaccine candidate for HIV. The vaccine induced immune responses against HIV and was found to be safe and well-tolerated [633]. mRNA-based immunotherapies have also shown promise for other infectious diseases. For example, a study published in Nature Medicine in 2020 reported the results of a Phase 1 trial of an mRNA-based vaccine against the respiratory syncytial virus (RSV). The vaccine was found to be safe and induced a strong immune response in healthy adults [634]. mRNA-based immunotherapies have shown promise in the development of personalized cancer vaccines. A study published in Nature in 2021 reported the results of a Phase 2 trial of an mRNA vaccine for patients with high-risk melanoma. The vaccine was found to induce durable immune responses and resulted in an improvement in progression-free survival compared to standard of care [635]. Table 11 provides an overview of the clinical trial outcomes of mRNA cancer vaccines in various cancer types.

**Table 11:** Clinical trial outcomes of mRNA cancer vaccines in various cancer types

Vaccine name	Cancer type	Primary endpoint	Secondary endpoints	Response rate	Adverse events	Description	Novelty	References
mRNA-4157	Melanoma	Safety and dose	Tumor response rate, duration of response, survival, immune response	50% objective response rate (partial or complete response)	Mild to moderate injection site reactions, fever, fatigue	Intradermal injection of a personalized mRNA vaccine targeting neoantigens	Personalized vaccine targeting multiple neoantigens derived from each patient's tumor	[635]
CV8102	Squamous cell carcinoma	Safety and dose	Immune response, objective response rate	30% objective response rate, enhanced immune response	Mild to moderate injection site reactions, flu-like symptoms	Intratumoral injection of an mRNA vaccine encoding five tumor-associated antigens	Multi-antigen mRNA vaccine using TLR7 agonist as an adjuvant	[636]
VB10.NEO	Colorectal cancer	Safety and dose	Immune response, tumor response rate	71% of evaluable patients showed tumor regression or stable disease	Mild to moderate injection site reactions, fatigue, flu-like symptoms	Personalized mRNA vaccine encoding up to 20 neoantigens, delivered via intradermal injection	Personalized vaccine targeting unique neoantigens specific to each patient's tumor	[631]
GEN-1	Ovarian cancer	Safety and dose	Tumor response rate, progression-free survival, overall survival, immune response	65% objective response rate, prolonged progression-free survival and overall survival	Mild to moderate injection site reactions, flu-like symptoms	Intraperitoneal injection of an mRNA vaccine encoding IL-12	Combines IL-12 gene therapy with mRNA vaccine delivery to target tumor microenvironment	[637]
Lipo-MERIT	Merkel cell carcinoma	Safety and dose	Immune response, tumor response rate	60% objective response rate, enhanced immune response	Mild to moderate injection site reactions, flu-like symptoms	mRNA vaccine formulated with lipid nanoparticles, encoding a modified oncoprotein	First-in-human study of mRNA vaccine formulated with novel lipid nanoparticles	[638]
mRNA-2416	Solid tumors	Safety and dose	Tumor response rate, immune response	10% partial response, 32% stable disease	Mild to moderate injection site reactions, flu-like symptoms, elevated liver enzymes	Intratumoral injection of mRNA encoding OX40L and IL-23, a combination to enhance anti-tumor immune response	Combination of two immune modulators to enhance immune response	[639]
RO7198457	Breast cancer	Safety and dose	Immune response, tumor response rate	11.9% partial response, 52.4% stable disease	Mild to moderate injection site reactions, flu-like symptoms, elevated liver enzymes	Intravenous injection of an mRNA vaccine encoding six breast cancer-associated antigens	Multi-antigen mRNA vaccine using a novel lipid nanoparticle formulation	[640]
BNT111	Melanoma	Safety and dose	Tumor response rate, immune response	50% of evaluable patients achieved a clinical response	Mild to moderate injection site reactions, flu-like symptoms, elevated liver enzymes	Intradermal injection of a personalized mRNA vaccine targeting up to 20 neoantigens	Personalized vaccine using patient-specific neoantigens and a novel lipid nanoparticle formulation	[641]
Kael-GemVax	Head and neck cancer	Safety and efficacy	Tumor response rate, progression-free survival, overall survival, immune response	46% of evaluable patients had tumor regression or stable disease, prolonged survival compared to historical controls	Mild to moderate injection site reactions, flu-like symptoms	Intradermal injection of an mRNA vaccine encoding multiple TAAs, combined with a TLR7 agonist as an adjuvant	Multi-antigen mRNA vaccine using a TLR7 agonist to enhance immune response	[642]
CV8101	Prostate cancer	Safety and dose	Immune response, tumor response rate	23% PSA reduction, enhanced immune response	Mild to moderate injection site reactions, flu-like symptoms, elevated liver enzymes	Intradermal injection of an mRNA vaccine encoding prostate cancer-associated antigens, combined with a TLR7 agonist as an adjuvant	Multi-antigen mRNA vaccine using a TLR7 agonist to enhance immune response	[643]
LN-145	Cervical cancer	Safety and efficacy	Objective response rate, duration of response, progression-free survival	44% objective response rate, 95% disease control rate	Mild to moderate injection site reactions, flu-like symptoms, anemia	Autologous TILs (tumor-infiltrating lymphocytes) transduced with mRNA encoding IL-2 and CD137 (4-1BB), infused into the patient after lymphodepletion	TILs genetically modified with mRNA encoding immune-stimulating cytokines and co-stimulatory molecules	[637]
JPT-TET	Pancreatic cancer	Safety and dose	Immune response, tumor response rate	30% objective response rate, enhanced immune response	Mild to moderate injection site reactions, flu-like symptoms, fatigue	Intradermal injection of an mRNA vaccine encoding multiple neoantigens, with a focus on TET (tumor endothelial marker) antigens	Multi-antigen mRNA vaccine targeting TET antigens expressed on the tumor vasculature	[644]
GRT-C901	Lung cancer	Safety and dose	Immune response, tumor response rate	8% partial response, 52% stable disease	Mild to moderate injection site reactions, fatigue, flu-like symptoms	Intramuscular injection of a multi-antigen mRNA vaccine encoding shared tumor antigens, with a focus on KRAS G12D	Multi-antigen mRNA vaccine targeting shared tumor antigens, with a focus on KRAS G12D	[645]
mRNA-5671	Ovarian cancer	Safety and dose	Immune response, tumor response rate	21% objective response rate	Mild to moderate injection site reactions, flu-like symptoms, anemia	Intramuscular injection of a mRNA vaccine encoding a cancer testis antigen (CTA)	mRNA vaccine targeting CTA antigens expressed on ovarian cancer cells	[635]
TriMixDC-MEL	Melanoma	Safety and efficacy	Tumor response rate, progression-free survival, overall survival, immune response	46% objective response rate, prolonged progression-free survival and overall survival	Mild to moderate injection site reactions, flu-like symptoms, fatigue	Ex vivo loading of patient-derived dendritic cells (DCs) with mRNA encoding three immune-stimulating molecules (CD40L, caTLR4, CD70) and tumor-associated antigens	DCs genetically modified with mRNA encoding immune-stimulating cytokines and tumor antigens	[646]
CV8102	Cutaneous melanoma	Safety and efficacy	Immune response, tumor response rate, duration of response	20% objective response rate, 60% disease control rate	Mild to moderate injection site reactions, flu-like symptoms	Intradermal injection of a mRNA vaccine encoding melanoma-associated antigens and toll-like receptor 7 (TLR7) agonist	mRNA vaccine targeting melanoma-associated antigens and immune-stimulating TLR7 agonist	[635]
RO7198457	Advanced solid tumors	Safety and efficacy	Immune response, tumor response rate, progression-free survival, overall survival	8% partial response, 31% stable disease	Mild to moderate injection site reactions, fatigue, flu-like symptoms	Intramuscular injection of a mRNA vaccine encoding multiple neoantigens, with a focus on individual patient-specific neoantigens	Personalized mRNA vaccine targeting individual patient-specific neoantigens	[631]
CV9202	Prostate cancer	Safety and efficacy	Immune response, tumor response rate, progression-free survival, overall survival	12% objective response rate, prolonged progression-free survival and overall survival	Mild to moderate injection site reactions, fatigue, flu-like symptoms	Intramuscular injection of a mRNA vaccine encoding prostate-specific antigens and immune-stimulating toll-like receptor 7/8 (TLR7/8) agonist	mRNA vaccine targeting prostate-specific antigens and immune-stimulating TLR7/8 agonist	[647]
AGS-004	HIV-associated malignancies	Safety and efficacy	Immune response, tumor response rate, progression-free survival	50% reduction in viral load, increased immune response	Mild to moderate injection site reactions, flu-like symptoms	Ex vivo loading of patient-derived dendritic cells (DCs) with mRNA encoding autologous HIV antigens	DCs genetically modified with mRNA encoding HIV antigens	[648]
TriMixDC-ALL	Acute lymphoblastic leukemia	Safety and efficacy	Tumor response rate, progression-free survival, overall survival, immune response	84% overall response rate, prolonged progression-free survival and overall survival	Mild to moderate injection site reactions, flu-like symptoms, fatigue	Ex vivo loading of patient-derived dendritic cells (DCs) with mRNA encoding three immune-stimulating molecules (CD40L, caTLR4, CD70) and tumor-associated antigens	DCs genetically modified with mRNA encoding immune-stimulating cytokines and tumor antigens	[649]
mRNA-4157	Head and neck cancer	Safety and efficacy	Immune response, tumor response rate, progression-free survival, overall survival	18% objective response rate, prolonged progression-free survival and overall survival	Mild to moderate injection site reactions, flu-like symptoms	Intramuscular injection of a mRNA vaccine encoding four cancer-testis antigens and immune-stimulating toll-like receptor 7/8 (TLR7/8) agonist	mRNA vaccine targeting multiple cancer-testis antigens and immune-stimulating TLR7/8 agonist	[650]
AGS-003	Metastatic renal cell carcinoma	Overall survival	Progression-free survival, immune response	Improved overall survival	Mild to moderate injection site reactions, flu-like symptoms, fatigue	Ex vivo loading of patient-derived dendritic cells (DCs) with mRNA encoding autologous tumor-associated antigens and immune-stimulating CD40L	DCs genetically modified with mRNA encoding tumor antigens and immune-stimulating cytokines	[651]
RNActive	Advanced pancreatic cancer	Safety and efficacy	Immune response, tumor response rate, progression-free survival, overall survival	20% objective response rate, prolonged progression-free survival and overall survival	Mild to moderate injection site reactions, flu-like symptoms	Intradermal injection of a mRNA vaccine encoding four pancreatic cancer-associated antigens and immune-stimulating toll-like receptor 7/8 (TLR7/8) agonist	mRNA vaccine targeting multiple pancreatic cancer-associated antigens and immune-stimulating TLR7/8 agonist	[631]
BNT111	Advanced melanoma	Safety and efficacy	Immune response, tumor response rate, progression-free survival, overall survival	51% objective response rate, prolonged progression-free survival and overall survival	Mild to moderate injection site reactions, fatigue, flu-like symptoms	Intramuscular injection of a mRNA vaccine encoding four melanoma-associated antigens and an immune-stimulating toll-like receptor 7 (TLR7) agonist	mRNA vaccine targeting melanoma-associated antigens and immune-stimulating TLR7 agonist	[652]
mRNA-4157	Head and neck squamous cell carcinoma	Safety and efficacy	Immune response, tumor response rate, progression-free survival, overall survival	17% objective response rate, prolonged progression-free survival and overall survival	Mild to moderate injection site reactions, fatigue, flu-like symptoms	Intramuscular injection of a mRNA vaccine encoding four cancer/testis antigens (MAGEA3, NY-ESO-1, MAGEC1, and HPV16 E6/E7) and an immune-stimulating toll-like receptor 7 (TLR7) agonist	mRNA vaccine targeting cancer/testis antigens and immune-stimulating TLR7 agonist	[653]
CV9201	Non-small cell lung cancer	Safety and efficacy	Immune response, tumor response rate, progression-free survival, overall survival	25% objective response rate, prolonged progression-free survival and overall survival	Mild to moderate injection site reactions, flu-like symptoms	Intramuscular injection of a mRNA vaccine encoding six lung cancer-associated antigens and an immune-stimulating toll-like receptor 7/8 (TLR7/8) agonist	mRNA vaccine targeting lung cancer-associated antigens and immune-stimulating TLR7/8 agonist	[631]

## mRNA encoding immunostimulants

Immunostimulants are commonly cytokines or chemokines that induce APC maturation and activation, activate T-cell mediated immunity and adjust the dysfunctional immune tumor microenvironment [654]. Intra-tumoral, intranodal, ID and IVroutes of administration have been used dosing of mRNA encoding immunostimulants, with most evaluations are currently in Phase I/II to assess the tolerability as monotherapy or combination therapy with other moieties, including either PD-1/PD-L1 antibodies or cancer vaccines [655].

**Fig. 15:**
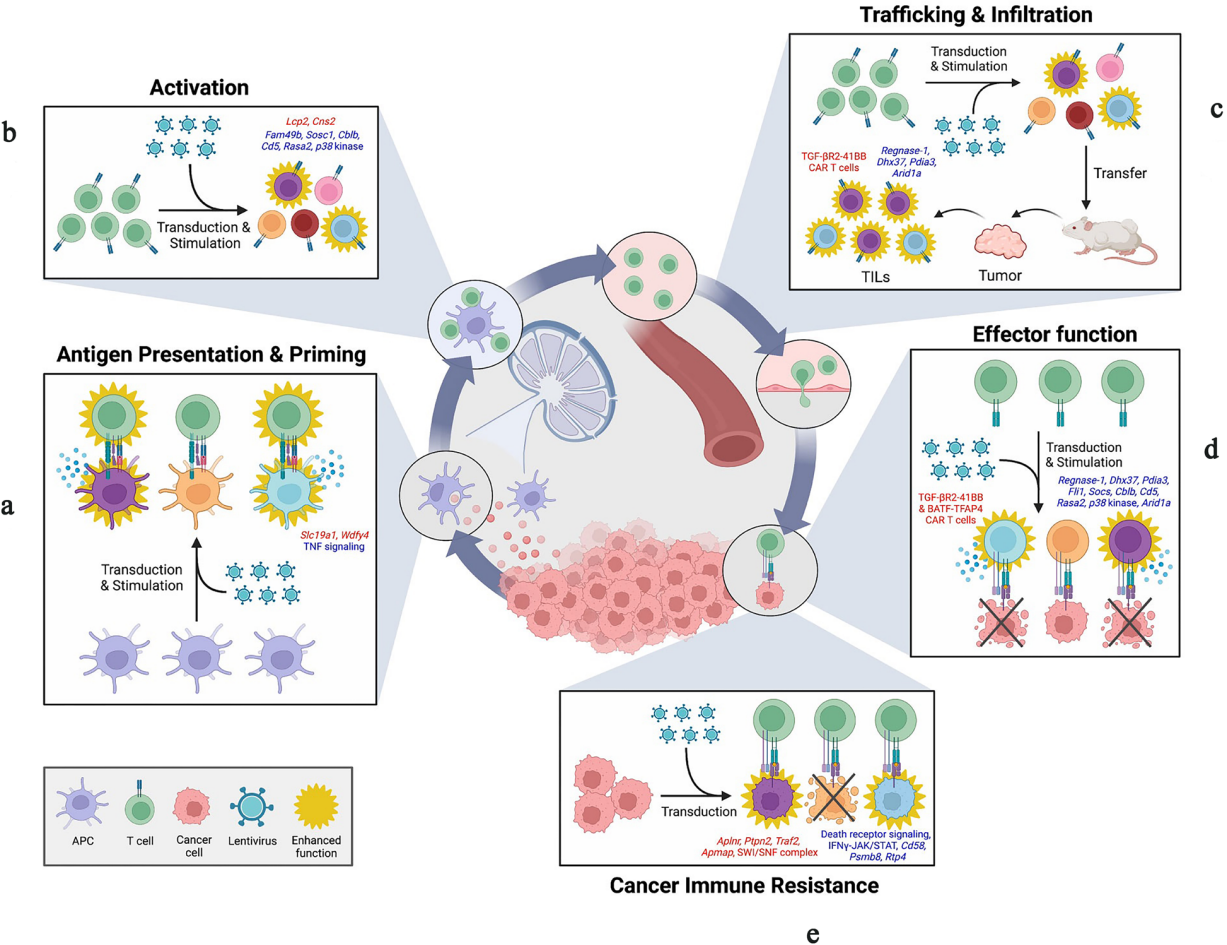
Identifying cancer-immunity cycle regulators through CRISPR screens. The cancer-immunity cycle outlines the step-by-step development of immune responses against tumors. CRISPR screening can be employed to examine cells at each stage of this cycle, identifying regulatory genes and the resulting phenotypic effects. **A** In the initial phase, tumor antigens are released by cancer cells and collected by APCs such as DCs, which may secrete cytokines when stimulated. APCs process and present the captured antigens using major histocompatibility complex proteins on their surface. APC trafficking to nearby lymph nodes enables cancer antigen presentation to naïve T cells, leading to T cell activation. Screens on APCs can reveal genes that regulate APC stimulation in response to tumor antigens and antigen presentation efficiency to T cells. **B** T cells exposed to antigens become primed and activated to target specific tumor antigens. T cell screens can pinpoint genes responsible for activation efficiency. **C** Primed T cells, including cytotoxic T lymphocytes, exit the lymph node, travel through the bloodstream, and infiltrate tumors as tumor-infiltrating lymphocytes (TILs). In vivo T cell screens can identify genes that facilitate TIL trafficking and infiltration. **D** Inside the tumor, T cells can finally recognize and respond to cancer-specific antigens, leading to tumor cell destruction. T cell screens can detect genes that improve tumor-killing activity. **E** Concurrently, screening tumor cells can uncover genes involved in resistance to T cell killing. CRISPR screens can identify positive regulators (shown in red) and negative regulators (shown in blue) at each step of the process. Reprinted from [657] with permission from Springer Nature

Figure 15 illustrates how CRISPR screening can be utilized to identify genes involved in the regulation of the cancer-immunity cycle, a process that describes the step-by-step development of immune responses against tumors. The cycle begins with the release of tumor antigens by cancer cells, which are collected by APCs such as DCs that process and present the antigens to naïve T cells, leading to their activation. CRISPR screening can be performed on APCs to identify genes that regulate antigen presentation efficiency and APC stimulation in response to tumor antigens. Similarly, T cell screens can identify genes responsible for activation efficiency, trafficking, infiltration, and tumor-killing activity. In vivo T cell screens can uncover genes that facilitate TIL trafficking and infiltration, while screening tumor cells can reveal genes involved in resistance to T cell killing. CRISPR screens can detect both positive and negative regulators at each stage of the cycle, enabling a better understanding of the complex mechanisms underlying the immune response against cancer. One pioneer player in this field is eTheRNA immunotherapies [656].

The company has developed a TriMix mRNA-based adjuvant that consists of three naked mRNA molecules, encoding the costimulatory molecule CD70 to induce activation of CD8^+^ T cells, the activation stimulator CD40 ligand (CD40L) to activate CD4^+^ T cells, and the constitutively active TLR4 (caTLR4) to facilitate DC antigen presentation [658]. The naked TriMix mRNA and ex-vivo DC loaded TriMix mRNA evaluated in multiple clinical trials are generally well tolerated and immunogenic [658]. Delivery of mRNA encoding TAAs (e.g. MAGE-A3, tyrosinase, gp-100 and melano-A/MART-1) and TriMix mRNA to DCs, ex vivo or in situ, can reprograms them to mature APCs, and subsequently prime the function of T cells [659]. In two Phase II studies for treating patients with stage III/IV melanoma, either as standalone product (TriMix mRNA plus TAA mRNA, so called TriMixDC-MEL) or combined with a CTLA-blocking monoclonal antibody ipilimumab checkpoint inhibitor, the products were able to elicit powerful immune response, in turn resulted in promising clinical response and prolonged disease-free survival rate (NCT01676779, NCT01302496) [660]. Another pioneer player Moderna has developed two mRNA products encapsulated in the LNP platform for intratumorally immunostimulatory activities [487]. These two products are currently evaluated in Phase I clinical trials to determine the safety and tolerability of repeated dosing [639]. One product is mRNA-2416, using mRNA encoding OX40L, either dosed alone or in combination with IV administered PD-L1 inhibitor durvalumab for treatment of lymphoma and metastatic ovarian cancer (NCT03323398) [639]. The other product is mRNA-2752, which is composed of OX40L/IL-23/IL-36Ƴ mRNAs for treatment of lymphoma (NCT03739931) [661]. In mRNA-2752 cocktail, OX40L composes the positive secondary signals to enhance T cell effector function, expansion and survival [661]. IL-36Ƴ functions as proinflammatory cytokines to further boost anticancer responses [662]. IL-36 Ƴ also correlates with good prognosis in cancer patients, and induces a favorable T helper 1 type TME change [663]. IL-23 (IL-12 family members) can act as the central coordinators and bridge innate and adaptive immunities [664]. Besides IL-23, IL-12 mRNA is also commonly used for improved antitumor immunity [664]. Moderna is collaborating with AstraZeneca, and planning to develop MED I1191 (IL-12 mRNA) through intratumorally injection [665]. Meanwhile, BioNtech’s cationic lipoplexes loaded BNT151 (nucleoside modified IL-12 mRNA) was also under pre-clinical evaluation for amplification of vaccine induced T cell response through IV administration [666]. These two products are planned for initiation of Phase I clinical studies in 2021 [666]. It should be noted that several small molecule drugs, especially the kinase inhibitors such as sunitinib, are potent modifiers of the suppressive tumor microenvironment [667]. Sunitinib base formulated in a nanoemulsion, when administered i.v., significantly decreased the content of regulatory T cells (Treg) and myeloid-derived suppressor cells (MDSC) and increased T-cells in the melanoma, and enhanced the tumor growth inhibition of a therapeutic vaccine [668].

## mRNA vaccine encoding tumor associated antigens

One of the key obstacles to the development of an effective cancer vaccine is the difficulties in antigen selection [669]. Cancer vaccines can be designed to target TAAs that are preferentially expressed in malignant cells [670]. For instance, tyrosinase, gp100, MAGE-A3, MAGE-C2 have been identified as TAAs for melanoma [239]. A cocktail of mRNA vaccines encoding all the TAAs have been used to treat metastatic melanoma in multiple clinical studies [239]. Table 12 illustrates the expression levels of tumor-specific antigens in various cancer types and their potential for targeting with mRNA vaccines. For example, in breast cancer, the HER2/neu antigen has a high expression level with 20–30% frequency of expression, which makes it a suitable target for mRNA vaccines. Similarly, the CEA antigen is highly expressed in colorectal cancer, with a frequency of 70–80%, making it another promising target. Table 12 goes on to present several other cancer types such as lung, melanoma, ovarian, pancreatic, and prostate, all with unique antigens that can be potentially targeted by mRNA vaccines. The frequency of expression, tissue specificity, and expression level of these antigens vary, which may influence the efficacy of targeted therapies. This data offers valuable insights for researchers working on personalized cancer treatments using mRNA vaccines.

**Table 12:** Expression levels of tumor-specific antigens in various cancer types

Cancer Type	Antigen Name	Expression Level	Tissue Specificity	Frequency of Expression	Potential for Targeting with mRNA Vaccines	Reference
Breast	HER2/neu	High	Breast	20–30%	Yes	[671]
Colorectal	CEA	High	Intestine	70–80%	Yes	[672]
Lung	EGFR	High	Lung	10–20%	Yes	[673]
Melanoma	MART-1	High	Skin	60–70%	Yes	[674]
Ovarian	CA125	High	Ovary	80–90%	Yes	[675]
Pancreatic	MUC1	High	Pancreas	90–100%	Yes	[676]
Prostate	PSA	High	Prostate	80–90%	Yes	[677]
Renal	CAIX	High	Kidney	85–95%	Yes	[678]
Ovarian	NY-ESO-1	Medium	Ovary	10–15%	Yes	[679]
Breast	MAGE-A1	Medium	Breast	15–20%	Yes	[44]
Melanoma	gp100	Medium	Skin	30–40%	Yes	[680]
Prostate	PSCA	Medium	Prostate	20–30%	Yes	[681]
Colorectal	MAGE-A3	Low	Intestine	5–10%	Yes	[682]
Lung	NY-ESO-1	Low	Lung	5–10%	Yes	[683]
Pancreatic	Survivin	Low	Pancreas	5–10%	Yes	[684]
Bladder	MAGE-A12	Low	Bladder	5–10%	Yes	[685]
Brain	EGFRvIII	Low	Brain	20–30%	Yes	[686]
Breast	BRCA1	Low	Breast	10–15%	Yes	[687]
Colorectal	TP53	Low	Intestine	15–20%	Yes	[688]
Kidney	CD105	Low	Kidney	10–15%	Yes	[689]
Liver	AFP	Low	Liver	60–70%	Yes	[690]
Lung	SLC34A2	Low	Lung	5–10%	Yes	[691]
Ovarian	TP53	Low	Ovary	20–30%	Yes	[692]
Pancreatic	KIF20A	Low	Pancreas	10–15%	Yes	[693]
Prostate	PSMA	Low	Prostate	5–10%	Yes	[694]
Sarcoma	NY-ESO-1	Low	Bone and Soft Tissue	5–10%	Yes	[695]
Stomach	HER2/neu	Low	Stomach	10–15%	Yes	[696]
Thyroid	Thyroglobulin	Low	Thyroid	80–90%	Yes	[697]
Uterine	p16	Low	Uterus	15–20%	Yes	[698]
Esophageal	MAGE-A4	Low	Esophagus	5–10%	Yes	[699]
Head and Neck	p16	Low	Head and Neck	25–30%	Yes	[700]
Leukemia	WT1	Low	Blood	75–85%	Yes	[701]
Lymphoma	CD19	Low	Lymphatic System	100%	Yes	[702]
Mesothelioma	Mesothelin	Low	Lungs	70–80%	Yes	[703]
Multiple Myeloma	NY-ESO-1	Low	Blood	20–30%	Yes	[704]
Neuroblastoma	GD2	Low	Nervous System	95–100%	Yes	[705]
Pancreatic	WT1	Low	Pancreas	10–15%	Yes	[706]
Prostate	MUC1	Low	Prostate	5–10%	Yes	[707]
Renal	WT1	Low	Kidney	15–20%	Yes	[708]
Sarcoma	SSX2	Low	Bone and Soft Tissue	5–10%	Yes	[709]
Stomach	MAGE-A3	Low	Stomach	10–15%	Yes	[710]
Testicular	MAGE-A4	Low	Testis	5–10%	Yes	[711]
Uterine	NY-ESO-1	Low	Uterus	5–10%	Yes	[698]
Acute Myeloid Leukemia	PR1	Medium	Blood	60–70%	Yes	[712]
Bladder	NY-ESO-1	Medium	Bladder	20–30%	Yes	[713]
Brain	Survivin	Medium	Brain	50–60%	Yes	[714]
Breast	HER2/neu	Medium	Breast	20–30%	Yes	[715]
Colorectal	CEA	Medium	Intestine	50–60%	Yes	[716]
Kidney	Renin	Medium	Kidney	50–60%	Yes	[717]
Liver	Glypican-3	Medium	Liver	50–60%	Yes	[718]
Lung	MUC1	Medium	Lung	50–60%	Yes	[719]
Ovarian	MUC1	Medium	Ovary	50–60%	Yes	[720]
Pancreatic	MUC1	Medium	Pancreas	50–60%	Yes	[721]
Prostate	PSA	Medium	Prostate	50–60%	Yes	[222]
Sarcoma	MAGE-A4	Medium	Bone and Soft Tissue	20–30%	Yes	[722]
Stomach	Survivin	Medium	Stomach	50–60%	Yes	[723]
Thyroid	Thyroid Peroxidase	Medium	Thyroid	50–60%	Yes	[724]

One well-known example of mRNA vaccine platform falls into this category is Lipo-MERIT [725]. As mentioned earlier, Lipo-MERIT is fabricated by complexing mRNA with cationic lipid such as DOTMA or DOTAP [725]. The lipoplexes with a cationic lipid: DOPE (helper lipid): mRNA ratio of 1.3:2 (≈250 nm in size and ≈30 mV in zeta potential) were shown to efficiently target the splenic DCs in mice and led to strong activation of NK, B, CD4^+^, CD8^+^ T cells, subsequently resulting in potent immunotherapeutic efficacy in multiple mouse cancer models and was translated into clinics [726]. In one clinical study (NCT02410733), the mRNA vaccine (BNT111) encoding four TAAs (NY-ESO-1, MAGE-A3, tyrosinase, and TPTE) was evaluated in patients bearing advanced melanoma [239]. Results demonstrated that three patients generated T cell responses against NY-ESO-1, two of which also showed responses against MAGE-A3 [727]. Recently, BioNTech announced a strategic collaboration with Regeneron to initiate the phase II clinical trial combining BNT111 with Regeneron Libtayo (cemiplimab), a fully humanized anti-PD-1 therapy in patients with anti-PD1-refractory/relapsed, unresectable Stage III or IV cutaneous melanoma [351]. Another player in the campaign is CureVac AG.

CureVac have developed mRNA vaccine CV9202, containing mRNAs encoding 6 different NSCLC TAAs (MUC-1, surviving, Trophoblast Glycoprotein, NY-ESO-1, MAGE-C1 and MAGE-C2) [728]. The naked TAA mRNA vaccines were co-delivered with protamine/mRNA complexes, which are known to have self-adjuvant properties as discussed earlier [74]. The new collaboration focused on CureVac’s CV9202 in early clinical development, in combination with afatinib for patients with advanced or metastatic epidermal growth factor mutated NSCLC, and in combination with chemo-radiation therapy in patients with unresectable stage III NSCLC [728]. For the first study, the vaccine treatment was well tolerated, with observations of only some inject site reactions and flu-like symptoms [729]. Increased antigen-specific immune response was observed in majority of the patients (84%). Antigen specific antibody and T cells are both increased, supporting further investigation of mRNA-based therapy with check-point inhibitors in treating NSCLC [647]. Moreover, Immunomic Therapeutics is collaborating with Dr. Duane Mitchell at the University of Florida on a Phase II proof of concept study using a pp65-lysosomal-associated membrane protein (LAMP)-based mRNA DC vaccine to treat patients bearing glioblastoma [730]. pp65 is a major cytomegalovirus (CMV) protein that provides exceptional tumor specificity for glioblastoma and is designed to stimulate pp65-specific CD4^+^ and CD8^+^ T cell response [731]. The previous phase I study showed a median overall survival of 35 months and progression-free survival of 31 months [731]. Table 13 highlights the different types of mRNA vaccines encoding tumor-associated antigens (TAAs) and their respective properties.

**Table 13:** The different types of mRNA vaccines encoding tumor-associated antigens (TAAs)

TAA Type	Advantages	Disadvantages	Immunogenicity	Efficacy	Safety	Stability	Reference
Shared TAAs	Target a broad range of tumors, useful for prevention of cancers	Low specificity and efficacy against certain cancers	Moderate to high, depending on TAA specificity	Varies depending on TAA and cancer type	Generally safe but may cause autoimmune reactions	Short half-life and may require frequent dosing	[732]
Differentiated TAAs	High specificity against individual cancer cells	Only effective against certain types of cancer cells	High	High, with potential for complete tumor regression	Generally safe but may cause autoimmune reactions	Short half-life and may require frequent dosing	[733]
Cancer-testis antigens	Specific to tumor cells and not found in normal adult tissues	Expression may vary between patients and be low in some cases	High in patients expressing the TAA	Varies depending on TAA and cancer type	Generally safe but may cause autoimmune reactions	Short half-life and may require frequent dosing	[508]
Overexpressed TAAs	Overexpressed in a broad range of cancers	May also be expressed in normal tissues, leading to toxicity	Moderate to high, depending on TAA specificity	Varies depending on TAA and cancer type, but potential for complete tumor regression	Generally safe but may cause autoimmune reactions	Short half-life and may require frequent dosing	[172]

Shared TAAs target a broad range of tumors, offering potential for cancer prevention, but exhibit low specificity and efficacy against certain cancers. Differentiated TAAs provide high specificity against individual cancer cells but are only effective against specific types of cancer cells. Cancer-testis antigens are specific to tumor cells and not found in normal adult tissues, although their expression may vary between patients. Overexpressed TAAs are present in a wide range of cancers but can also be expressed in normal tissues, leading to potential toxicity. In general, immunogenicity ranges from moderate to high, with efficacy varying depending on the TAA and cancer type. While these mRNA vaccines are generally considered safe, they may cause autoimmune reactions. All TAA types have a short half-life, which necessitates frequent dosing.

## mRNA vaccine encoding neoantigen, personalized vaccine

According to Table 14, there are various types of tumor-associated antigens (TAAs) being utilized in mRNA cancer vaccines. Shared antigens, differentiation antigens, and cancer-testis antigens are common to many tumor types, expressed in certain tumors, or highly immunogenic, respectively, and have been used in multiple trials. Neoantigens, viral antigens, oncofetal antigens, tumor-specific antigens, mutated self-antigens, shared mutated antigens, overexpressed antigens, and glycopeptide antigens are all in early-stage trials. Some TAAs have the potential for autoimmunity, while others are unique to specific tumors or patients. The immunogenicity of these antigens varies, with some having high immunogenicity and others having a more limited understanding. The stability of these antigens is generally unstable, and their expression levels range from low to high.

**Table 14:** The different types of tumor-associated antigens (TAA) used in mRNA cancer vaccines

Shared antigens	Common to many tumor types	Potential for autoimmunity	Variable	Moderate to High	Unstable	Moderate	Multiple trials	Reference
Differentiation antigens	Expressed in certain tumor types	Not present in all tumors	Variable	Moderate to High	Unstable	Moderate	Multiple trials	[732]
Cancer-testis antigens	Highly immunogenic	Restricted to certain tumor types	Variable	High	Unstable	Moderate to High	Multiple trials	[733]
Neoantigens	Patient-specific	Unique to each tumor	High	High	Unstable	Low to Moderate	Early-stage trials	[58]
Viral antigens	Easily recognized by immune system	Limited to virus-associated cancers	Variable	Moderate to High	Unstable	Moderate	Early-stage trials	[430]
Oncofetal antigens	High expression in tumors	Also expressed in some normal tissues	Variable	Moderate to High	Unstable	Moderate	Early-stage trials	[734]
Tumor-specific antigens	Unique to tumors	Low expression levels	High	High	Unstable	Low to Moderate	Early-stage trials	[735]
Mutated self-antigens	Patient-specific	Potential for off-target effects	Variable	High	Unstable	Low to Moderate	Early-stage trials	[736]
Shared mutated antigens	Common mutations in tumors	Potential for autoimmunity	Variable	Moderate to High	Unstable	Moderate	Early-stage trials	[638]
Overexpressed antigens	High expression in tumors	Also expressed in normal tissues	Variable	Moderate to High	Unstable	Moderate	Early-stage trials	[626]
Glycopeptide antigens	Unique glycosylation patterns in tumors	Limited understanding of immunogenicity	Variable	Moderate to High	Unstable	Moderate to High	Early-stage trials	[737]

The utilization of TAAs in mRNA cancer vaccines has the potential to revolutionize cancer treatment and provide new therapeutic options for patients. These antigens can be targeted by the immune system to eliminate cancer cells. Several obstacles limit the further application of TAA vaccines, including: (1) only limited TAAs have been identified for certain solid tumors resulting in limits of applications, and (2) patients harboring extensive variability in TAAs that gives rise to evasion of immune effectors and generation of resistance, (3) TAAs are also present in normal tissues [239]. Vaccines against TTAs could potentially initiate central and peripheral tolerance responses, lowering vaccination efficiency [735].

Tumor-specific antigens, termed neoantigens, are now the core targets of mRNA vaccines. Neoantigens are derived from random somatic mutations in tumor cells and not present in normal cells [738]. Neoantigens could be recognized by the host immune system as a “non-self” motif and thus are an appealing target for cancer vaccine [738]. The first step in developing a personalized neoantigen vaccine is to identify and confirm patient-specific immunogenic non-synonymous somatic mutations expressed in the tumor [739]. A biopsy of tumor tissue is taken for whole-exome, RNA, or transcriptome sequencing [739]. Non-synonymous somatic mutations in cancer, such as point mutations and insertion-deletions, could be identified by comparing the sequences of the tumor and matched healthy tissues [740]. Next, mutations with the highest immunogenicity are screened, analyzed, and identified using MHC class I epitope prediction algorithms [741]. Ranked lists of candidate antigens are further confirmed based on in vitro binding assay results.

Various types of variant mutations can be targeted by neoantigen based vaccine [741]. Multiple delivery strategies have been developed for neoantigens, including synthetic long peptides (SLPs) and nucleic acid (DNA/mRNA) based vaccines, either through direct injection of unformulated antigens, DC-based autologous transfer, or biomaterial-based delivery system [742]. In a pioneered phase I clinical study, a selected pool of 20 SLPs were SC administered together with adjuvant polyICLC to 6 patients with advanced cutaneous melanoma [743]. These SLPs were shown to induce both CD4^+^ T cells and CD8^+^ T cells response [744]. Four of the six patients were cancer-free 25 months post-treatment, demonstrating the viability of neoantigen vaccination in anticancer treatment [743]. However, peptides have limited immunogenicity, rapid clearance, and different physical–chemical properties restricting their clinical applications [744].

Most recently, some researchers reported that immunizing advanced melanoma patients in a clinical study using IVT mRNA encoding neoantigens through intranodal (i.n.) injection [745]. The ultrasound-guided injection could maximize the capture of antigens by APCs [746]. Potent T cell responses against multiple neoantigens were achieved in all the patients after vaccination [219]. Despite the encouraging initial results, the wide application of IN injection may be limited by the viability of the techniques and the difficulties for repeated dosing [745]. Non-viral platforms have until recently been applied to the delivery of mRNA encoding neoantigens [58]. Multiple clinical trials investigating the safety and efficacy of mRNA vaccine encoding neoantigens are ongoing [219]. Moderna and Merck collaborated to develop mRNA-5671, a Kras personalized vaccine (encoding KRAS neoantigens), alone or in together with Merck’s PD-1 specific antibody KEYTRUDA (Pembrolizumab) to treat patients with pancreatic cancer in Phase I Trial. LNPs were utilized to deliver mRNA-5671 intramuscularly every 3 weeks, 9 cycles in total [747]. Results suggested that anti-tumoral immune response was developed and the formulation is overall well-tolerated [747]. Another product is mRNA-4157, a personalized vaccine encapsulated in LNPs, for treating patients with resected solid tumors including melanoma, bladder carcinoma and NSCLC, as monotherapy or in combination with pembrolizumab (NCT03313778) [70]. The mRNA-4157 based mono and combination therapy with pembrolizumab showed an acceptable safety profile along with remarkable neoantigen-specific T cell responses [174]. Twelve out of thirteen patients treated by monotherapy were reported to be disease-free [732]. BioNtech collaborated with Genentech to join the campaign and to evaluate the safety and efficacy of mRNA personalized vaccine, RO7198457 delivered by Lipo-MERIT platform in multiple phase I and II clinical trials [748].

## Conclusion and future perspectives

With the recent approval of two mRNA LNP vaccines to prevent COVID-19, mRNA vaccines are experiencing a considerable burst in preclinical and clinical research in both cancer and infectious disease fields. The challenges of developing cancer vaccines versus infectious disease vaccines lie in: firstly, most infectious disease vaccines are prophylactic, whereas cancer vaccines are therapeutic. The cases for preventive cancer vaccines are rare with only two FDA approved such vaccines, and these two vaccines are applied to prevent virus-induced malignancies (HPV and HBV). Though anti-cancer prophylactic vaccines are still under pre-clinical investigation, the clinical translation is limited by the difficulties of antigen predictions and the suboptimal immunogenicity. Secondly, most antigens for infectious disease (bacterial or virus-driven) are exogeneous motifs typically presented by the MHC_II_ molecule. Vaccines targeting these exogenous antigens induce neutralizing antibodies mediated humoral response. In some cases, CD4^+^ T cell-mediated immune response is partially involved and required, whereas CD8^+^ cytotoxic T cells play crucial roles in the clearance of malignant cells with somatic mutations. Thus, the anticancer therapeutic vaccine not only needs to boost humoral response, CD4 + T cell response but also needs to activate the MHC_I_ mediated CD^+^ 8 T cells responses, which further adds to the difficulties for efficient boosting of a robust antitumor immunity. Another major hurdle for efficient anticancer vaccine development is to identify and efficiently deliver highly immunogenic tumor-specific antigens. Tumor antigens are highly variable across different individuals, and some are less immunogenic and can invade the recognition by the host immune system. Even if the antigen is immunogenic, a suppressive microenvironment could prevent effective T cells’ infiltration and cause T cell exhaustion. Lastly, as a therapeutic vaccine for treating a chronic disease like cancer, multiple/repeatable dosing with higher dosage than prophylactic vaccines is required, raising the safety criteria for both mRNAs and the carriers. Among other cancer vaccines, including DC-based vaccines and protein-based vaccines, mRNA stands out for several reasons: (1) mRNA could simultaneously encode multiple antigens, or a full protein with both MHC_I_ and MHC_II_ binding epitopes to facilitate both humoral and cellular adaptive immune response, providing a more intensified anti-tumor immunity. (2) Compared with DNA vaccine, mRNA vaccines are non-integrating, highly degradable, with no insertional mutagenesis potentials. Compared to protein or cell-mediated vaccines, the IVT production of mRNA is free of cellular and pathogenic viral components, with no infectious possibilities. Most mRNA vaccines tested in ongoing clinical trials are generally well tolerated, with rare cases of injection site reactions. Systemic inflammation may be a major concern for mRNA vaccines due to its intrinsic immunostimulant-like function to activate the TLR7/8 pathway and to induce the type I IFN responses. However, type I IFN mediated innate immune response could be reduced by removal of the dsRNA contaminants, codon optimizations, and nucleotide modifications. The innate immune response could also be restricted to the local injection site by properly designing the delivery systems and changing the administration routes. The activation of type I interferon responses is not only associated with inflammation but also potentially with autoimmunity. Therefore, identifying individuals at an increased risk of autoimmune reactions before mRNA vaccination is another precautious step necessary to be taken. (3) Another advantage of mRNA cancer vaccine is the rapid and scalable manufacturing. The mature manufacturing process of mRNA and formulation platform allows productions of a same or a new type of vaccine within a very short period. Although identifying immunogenic TAAs/TSAs and overcoming suppressive tumor microenvironment still remain major hurdles for mRNA vaccine, the recent discovery and identification of neoantigens facilitate personalized vaccine treatment applications. mRNA encoded neoantigens have become the frontrunner in the personalized vaccine campaign. Multiple clinical studies led by the mRNA LNP pioneers BioNTech and Moderna, already presented promising results (with a readout of antitumor immunity) using personalized vaccines in several clinical trials treating multiple solid tumors, including metastatic melanoma and aggressive pancreatic cancers, opening a new era for therapeutic cancer vaccines.

To further improve the potency of mRNA anticancer vaccines, multiple clinical trials are ongoing to evaluate the combination of mRNA vaccines with either cytokine therapies or checkpoint inhibitor therapies. mRNA is a powerful and versatile cancer vaccine platform. Its successful development towards clinical translation will remarkably strengthen our ability to combat cancers. Future investigations should continue focusing on (but not limited to) understanding and utilizing the paradoxical inherent innate immunity of mRNA, improving the efficiency of antigen expression and presentation by designing advanced and tolerable delivery systems, and modifying mRNA structures to achieve extended and controlled duration of expression.

## Data Availability

Not applicable.

## References

[1] Lu YC, Robbins PF. Cancer immunotherapy targeting neoantigens. Semin Immunol. 2016;28(1):22–7. 10.1016/j.smim.2015.11.002 PMC4862880 26653770

[2] Miao L, Zhang Y, Huang L. mRNA vaccine for cancer immunotherapy. Mol Cancer. 2021;20(1):1–23. 10.1186/s12943-021-01335-5 PMC7905014 33632261

[3] Hegde PS, Chen DS. Top 10 Challenges in Cancer Immunotherapy. Immunity. 2020;52(1):17–35. 10.1016/j.immuni.2019.12.011 31940268

[4] Li W, Deng Y, Chu Q, Zhang P. Gut microbiome and cancer immunotherapy. Cancer Lett. 2019;447:41–7. 10.1016/j.canlet.2019.01.015 30684593

[5] Dobosz P, Dzieciątkowski T. The Intriguing History of Cancer Immunotherapy. Front Immunol. 2019;2965. 10.3389/fimmu.2019.02965 PMC6928196 31921205

[6] Liu S, Galat V, Galat4 Y, Lee YKA, Wainwright D, Wu J. NK cell-based cancer immunotherapy: from basic biology to clinical development. J Hematol Oncol. 2021;14:1–7. 10.1186/s13045-020-01014-w PMC7788999 33407739

[7] Tan S, Li D, Zhu X. Cancer immunotherapy: Pros, cons and beyond. Biomed Pharmacother. 2020;124:109821. 10.1016/j.biopha.2020.109821 31962285

[8] Xu Z, Wang X, Zeng S, Ren X, Yan Y, Gong Z. Applying artificial intelligence for cancer immunotherapy. Acta Pharm Sin B. 2021;11(11):3393–405. 10.1016/j.apsb.2021.02.007 PMC8642413 34900525

[9] Stasiak M, Zawadzka‐starczewska K, Lewiński A. Significance of HLA Haplotypes in Two Patients with Subacute Thyroiditis Triggered by mRNA‐Based COVID‐19 Vaccine. Vaccines. 2022;10(2):280. 10.3390/vaccines10020280 PMC8879821 35214738

[10] Quatrini L, Mariotti FR, Munari E, Tumino N, Vacca P, Moretta L. The immune checkpoint PD-1 in natural killer cells: Expression, function and targeting in tumour immunotherapy. Cancers (Basel). 2020;12(11):3285. 10.3390/cancers12113285 PMC7694632 33172030

[11] Beck JD, Reidenbach D, Salomon N, Sahin U, Türeci Ö, Vormehr M, et al. mRNA therapeutics in cancer immunotherapy. Mol Cancer. 2021;20(1):1–24. 10.1186/s12943-021-01348-0 PMC8047518 33858437

[12] Zhao X, Pan X, Wang Y, Zhang Y. Targeting neoantigens for cancer immunotherapy. Biomark Res. 2021;28(7):365–70. 10.1186/s40364-021-00315-7 PMC8317330 34321091

[13] Bergman PJ. Cancer Immunotherapies. Vet Clin North Am - Small Anim Pract. 2019;49(5):881–902. 10.1016/j.cvsm.2019.04.010 31186125

[14] Schirrmacher V. From chemotherapy to biological therapy: A review of novel concepts to reduce the side effects of systemic cancer treatment (Review). Int J Oncol. 2019;54(2):407–19. 10.3892/ijo.2018.4661 PMC6317661 30570109

[15] Rallis KS, Hillyar CRT, Sideris M, Davies JK. T-cell-based immunotherapies for haematological cancers, Part A: A SWOT analysis of immune checkpoint inhibitors (ICIs) and bispecific T-Cell engagers (BiTEs). Anticancer Res. 2021;41(3):1143–56. 10.21873/anticanres.14870 33788704

[16] Wu P, Geng B, Chen Q, Zhao E, Liu J, Sun C, et al. Tumor cell-derived TGFb1 attenuates antitumor immune activity of T cells via regulation of PD-1 mRNA. Cancer Immunol Res. 2020;8(12):1470–84. 10.1158/2326-6066.CIR-20-0113 32999004

[17] Jung EH, Jang HR, Kim SH, Suh KJ, Kim YJ, Lee JH, et al. Tumor LAG-3 and NY-ESO-1 expression predict durable clinical benefits of immune checkpoint inhibitors in advanced non-small cell lung cancer. Thorac Cancer. 2021;12(5):619–30. 10.1111/1759-7714.13834 PMC7919166 33458968

[18] Hwang WL, Pike LRG, Royce TJ, Mahal BA, Loeffler JS. Safety of combining radiotherapy with immune-checkpoint inhibition. Nat Rev Clin Oncol. 2018;15(8):477–94. 10.1038/s41571-018-0046-7 29872177

[19] Liu J, Chen Z, Li Y, Zhao W, Wu JB, Zhang Z. PD-1/PD-L1 Checkpoint Inhibitors in Tumor Immunotherapy. Front Pharmacol. 2021;12:731798. 10.3389/fphar.2021.731798 PMC8440961 34539412

[20] Matlung HL, Szilagyi K, Barclay NA, van den Berg TK. The CD47-SIRPα signaling axis as an innate immune checkpoint in cancer. Immunol Rev. 2017;276(1):145–64. 10.1111/imr.12527 28258703

[21] Lin WY, Wang HH, Chen YW, Lin CF, Fan HC, Lee YY. Gene modified car-t cellular therapy for hematologic malignancies. Int J Mol Sci. 2020;21(22):8655. 10.3390/ijms21228655 PMC7697548 33212810

[22] Lee YG, Marks I, Srinivasarao M, Kanduluru AK, Mahalingam SM, Liu X, et al. Use of a single CAR T cell and several bispecific adapters facilitates eradication of multiple antigenically different solid tumors. Cancer Res. 2019;79(2):387–96. 10.1158/0008-5472.CAN-18-1834 30482775

[23] Fuca G, Reppel L, Landoni E, Savoldo B, Dotti G. Enhancing Chimeric Antigen Receptor T-Cell Efficacy in Solid Tumors. Clin Cancer Res. 2020;26(11):2444–51. 10.1158/1078-0432.CCR-19-1835 PMC7269829 32015021

[24] Liu D. CAR-T “the living drugs”, immune checkpoint inhibitors, and precision medicine: A new era of cancer therapy. J Hematol Oncol. 2019;12:1–5. 10.1186/s13045-019-0819-1 PMC6842223 31703740

[25] Chen Z, Pan H, Luo Y, Yin T, Zhang B, Liao J, et al. Nanoengineered CAR-T Biohybrids for Solid Tumor Immunotherapy with Microenvironment Photothermal-Remodeling Strategy. Small. 2021;17(14):2007494. 10.1002/smll.202007494 33711191

[26] Posey AD, Schwab RD, Boesteanu AC, Steentoft C, Mandel U, Engels B, et al. Engineered CAR T Cells Targeting the Cancer-Associated Tn-Glycoform of the Membrane Mucin MUC1 Control Adenocarcinoma. Immunity. 2016;44(6):1444–54. 10.1016/j.immuni.2016.05.014 PMC5358667 27332733

[27] Wang M, Munoz J, Goy A, Locke FL, Jacobson CA, Hill BT, et al. KTE-X19 CAR T-Cell Therapy in Relapsed or Refractory Mantle-Cell Lymphoma. N Engl J Med. 2020;382(14):1331–42. 10.1056/NEJMoa1914347 PMC7731441 32242358

[28] Poschke IC, Hassel JC, Rodriguez-Ehrenfried A, Lindner KAM, Heras-Murillo I, Appel LM, et al. The Outcome of Ex Vivo TIL Expansion Is Highly Influenced by Spatial Heterogeneity of the Tumor T-Cell Repertoire and Differences in Intrinsic in Vitro Growth Capacity between T-Cell Clones. Clin Cancer Res. 2020;26(16):4289–301. 10.1158/1078-0432.CCR-19-3845 32303540

[29] Liu S, Wang F, Tan W, Zhang L, Dai F, Wang Y, et al. CTLA4 has a profound impact on the landscape of tumor-infiltrating lymphocytes with a high prognosis value in clear cell renal cell carcinoma (ccRCC). Cancer Cell Int. 2020;20(1):519. 10.1186/s12935-020-01603-2 PMC7590466 33117084

[30] Kristensen NP, Heeke C, Tvingsholm SA, Borch A, Draghi A, Crowther MD, et al. Neoantigen-reactive CD8+ T cells affect clinical outcome of adoptive cell therapy with tumor-infiltrating lymphocytes in melanoma. J Clin Invest. 2022;132(2). 10.1172/JCI150535 PMC8759789 34813506

[31] Puntigam LK, Jeske SS, Götz M, Greiner J, Laban S, Theodoraki MN, et al. Immune checkpoint expression on immune cells of hnscc patients and modulation by chemo-and immunotherapy. Int J Mol Sci. 2020;21(15):5181. 10.3390/ijms21155181 PMC7432918 32707816

[32] Bianchi V, Harari A, Coukos G. Neoantigen-Specific Adoptive Cell Therapies for Cancer: Making T-Cell Products More Personal. Front Immunol. 2020;11:1215. 10.3389/fimmu.2020.01215 PMC7333784 32695101

[33] Stevanovic S, Helman SR, Wunderlich JR, Langhan MM, Doran SL, Kwong MLM, et al. A Phase II Study of Tumor-infiltrating Lymphocyte Therapy for Human Papillomavirus–associated Epithelial Cancers. Clin Cancer Res. 2019;25(5):1486–93. 10.1158/1078-0432.CCR-18-2722 PMC6397671 30518633

[34] Page DB, Yuan J, Redmond D, Wen YH, Durack JC, Emerson R, et al. Deep sequencing of T-cell receptor DNA as a biomarker of clonally expanded TILs in breast cancer after immunotherapy. Cancer Immunol Res. 2016;4(10):835-44. 10.1158/2326-6066.CIR-16-0013 PMC5064839 27587469

[35] Saxena M, van der Burg SH, Melief CJM, Bhardwaj N. Therapeutic cancer vaccines. Nat Rev Cancer. 2021;21(6):360-78. 10.1038/s41568-021-00346-0 33907315

[36] Xiao B, Li D, Xu H, Zhou X, Xu X, Qian Y, et al. An MRI-trackable therapeutic nanovaccine preventing cancer liver metastasis. Biomaterials. 2021;274:120893. 10.1016/j.biomaterials.2021.120893 34029913

[37] Islam MA, Rice J, Reesor E, Zope H, Tao W, Lim M, et al. Adjuvant-pulsed mRNA vaccine nanoparticle for immunoprophylactic and therapeutic tumor suppression in mice. Biomaterials. 2021;266:120431. 10.1016/j.biomaterials.2020.120431 PMC7528902 33099060

[38] Le Moignic A, Malard V, Benvegnu T, Lemiègre L, Berchel M, Jaffrès PA (2018). Preclinical evaluation of mRNA trimannosylated lipopolyplexes as therapeutic cancer vaccines targeting dendritic cells. J Control Release.

[39] Xie X, Hu Y, Ye T, Chen Y, Zhou L, Li F (2021). Therapeutic vaccination against leukaemia via the sustained release of co-encapsulated anti-PD-1 and a leukaemia-associated antigen. Nat Biomed Eng.

[40] Hollingsworth RE, Jansen K. Turning the corner on therapeutic cancer vaccines. NPJ Vacc. 2019;4(1):7. 10.1038/s41541-019-0103-y PMC6368616 30774998

[41] Feliciello I, Procino A (2021). mRNA vaccines: Why and how they should be modified. J Biol Res.

[42] Richards RM, Sotillo E, Majzner RG. CAR T cell therapy for neuroblastoma. Front Immunol. 2018;9:2380. 10.3389/fimmu.2018.02380 PMC6232778 30459759

[43] Huang H, Xiang J. Synergistic effect of lymphotactin and interferon γ-inducible protein-10 transgene expression in T-cell localization and adoptive T-cell therapy of tumors. Int J Cancer. 2004;109(6):817-25. 10.1002/ijc.20043 15027114

[44] Zhou D, Zheng H, Liu Q, Lu X, Deng X, Jiang L (2019). Attenuated plasmodium sporozoite expressing MAGE-A3 induces antigen-specific CD8+ T cell response against lung cancer in mice. Cancer Biol Med.

[45] Chandran SS, Klebanoff CA. T cell receptor-based cancer immunotherapy: Emerging efficacy and pathways of resistance. Immunol Rev. 2019;290(1):127-47. 10.1111/imr.12772 PMC7027847 31355495

[46] Rosenberg SA. Abstract IA14: Cell transfer immunotherapy targeting unique somatic mutations in cancer. 2019;7(2_Supplement):IA14.

[47] Lu YC, Jia L, Zheng Z, Tran E, Robbins PF, Rosenberg SA (2019). Single-cell transcriptome analysis reveals gene signatures associated with T-cell persistence following adoptive cell therapy. Cancer Immunol Res.

[48] June CH, O’Connor RS, Kawalekar OU, Ghassemi S, Milone MC. CAR T cell immunotherapy for human cancer. Science. 2018;359(6382):1361-5. 10.1126/science.aar6711 29567707

[49] Klampatsa A, Leibowitz MS, Sun J, Liousia M, Arguiri E, Albelda SM. Analysis and Augmentation of the Immunologic Bystander Effects of CAR T Cell Therapy in a Syngeneic Mouse Cancer Model. Mol Ther - Oncolytics. 2020;18:360-71. 10.1016/j.omto.2020.07.005 PMC7417672 32802940

[50] Xiao P, Wang J, Fang L, Zhao Z, Sun X, Liu X, et al. Nanovaccine-Mediated Cell Selective Delivery of Neoantigens Potentiating Adoptive Dendritic Cell Transfer for Personalized Immunization. Adv Funct Mater. 2021;31(36):2104068.

[51] Sahin U, Karikó K, Türeci Ö. MRNA-based therapeutics-developing a new class of drugs. Nat Rev Drug Discov. 2014;13(10):759-80. 10.1038/nrd4278 25233993

[52] Qin S, Tang X, Chen Y, Chen K, Fan N, Xiao W, et al. mRNA-based therapeutics: powerful and versatile tools to combat diseases. Signal Transduct Target Ther. 2022;7:1–35. 10.1038/s41392-022-01007-w PMC9123296 35597779

[53] McDonald I, Murray SM, Reynolds CJ, Altmann DM, Boyton RJ. Comparative systematic review and meta-analysis of reactogenicity, immunogenicity and efficacy of vaccines against SARS-CoV-2. NPJ Vacc. 2021;6(1):74. 10.1038/s41541-021-00336-1 PMC8116645 33986272

[54] Cruz-Zaragoza LD, Dennerlein S, Linden A, Yousefi R, Lavdovskaia E, Aich A (2021). An in vitro system to silence mitochondrial gene expression. Cell.

[55] Boudewijns S, Bloemendal M, de Haas N, Westdorp H, Bol KF, Schreibelt G (2020). Autologous monocyte-derived DC vaccination combined with cisplatin in stage III and IV melanoma patients: a prospective, randomized phase 2 trial. Cancer Immunol Immunother.

[56] Arya S, Lin Q, Zhou N, Gao X, Huang JD (2020). Strong Immune Responses Induced by Direct Local Injections of Modified mRNA-Lipid Nanocomplexes. Mol Ther - Nucleic Acids.

[57] Verbeke R, Lentacker I, Breckpot K, Van CS, De SSC, Dewitte H (2019). Abstract B136: Optimized messenger RNA immunolipoplexes for cancer immunotherapy: Balancing immunogenicity and adjuvancy. Cancer Immunol Res.

[58] Cafri G, Gartner JJ, Hopson K, Meehan RS, Zaks TZ, Robbins P (2019). Immunogenicity and tolerability of personalized mRNA vaccine mRNA-4650 encoding defined neoantigens expressed by the autologous cancer. J Clin Oncol.

[59] Webb C, Ip S, Bathula NV, Popova P, Soriano SKV, Ly HH, et al. Current Status and Future Perspectives on MRNA Drug Manufacturing. Mol Pharm. 2022;19(4):1047-58. 10.1021/acs.molpharmaceut.2c00010 35238565

[60] Persano S, Guevara ML, Li Z, Mai J, Ferrari M, Pompa PP (2017). Lipopolyplex potentiates anti-tumor immunity of mRNA-based vaccination. Biomaterials.

[61] Donaldson LJ, Rockville W, Sorra J, Gray L, Streagle S, Famolaro T (2020). Critical Appraisal Checklist. Expert Opin Pharmacother.

[62] Bhattacharya M, Sharma AR, Ghosh P, Patra P, Patra BC, Lee SS (2022). Bioengineering of Novel Non-Replicating mRNA (NRM) and Self-Amplifying mRNA (SAM) Vaccine Candidates Against SARS-CoV-2 Using Immunoinformatics Approach. Mol Biotechnol.

[63] Guo Y, Lei K, Tang L. Neoantigen vaccine delivery for personalized anticancer immunotherapy. Front Immunol. 2018;17. 10.3389/fimmu.2018.01499 PMC6036114 30013560

[64] Au L, Fendler A, Shepherd STC, Rzeniewicz K, Cerrone M, Byrne F (2021). Cytokine release syndrome in a patient with colorectal cancer after vaccination with BNT162b2. Nat Med.

[65] Gómez-Aguado I, Rodríguez-Castejón J, Vicente-Pascual M, Rodríguez-Gascón A, Solinís MÁ, Del Pozo-Rodríguez A. Nanomedicines to deliver mRNA: State of the art and future perspectives. Nanomaterials. 2020;10(2):364. 10.3390/nano10020364 PMC7075285 32093140

[66] Mourenza Á, Lorente-Torres B, Durante E, Llano-Verdeja J, Aparicio JF, Fernández-López A, et al. Understanding microRNAs in the Context of Infection to Find New Treatments against Human Bacterial Pathogens. Antibiotics. 2022;11(3):356. 10.3390/antibiotics11030356 PMC8944844 35326819

[67] Lei J, Jacobus EJ, Taverner WK, Fisher KD, Hemmi S, West K, et al. Expression of human CD46 and trans-complementation by murine adenovirus 1 fails to allow productive infection by a group B oncolytic adenovirusin murine cancer cells. J Immunother Cancer. 2018;6:1-6. 10.1186/s40425-018-0350-x PMC6000980 29898782

[68] Hao S, Inamdar VV, Sigmund EC, Zhang F, Stephan SB, Watson C (2022). BiTE secretion from in situ-programmed myeloid cells results in tumor-retained pharmacology. J Control Release.

[69] Huang X, Zhang G, Tang T, Liang T. Identification of tumor antigens and immune subtypes of pancreatic adenocarcinoma for mRNA vaccine development. Mol Cancer. 2021;20:1-8. 10.1186/s12943-021-01310-0 PMC7917175 33648511

[70] Zhong S, Breton B, Zheng W, McFadyen I, Hopson K, Frederick J, et al. Abstract 6539: Bioinformatics algorithm of mRNA-4157 identifies neoantigens with pre-existing TIL reactivities in colorectal tumors. Cancer Res. 2020;80:6539.

[71] Bol K, Aarntzen E, Pots J, Olde Nordkamp M, van de Rakt M, et al. Prophylactic vaccines are potent activators of monocyte-derived dendritic cells and drive effective anti-tumor responses in melanoma patients at the cost of toxicity. Cancer Immunol. Immunother. 2016;65:327-39. 10.1007/s00262-016-1796-7 PMC4779136 26861670

[72] Markov O, Oshchepkova A, Mironova N. Immunotherapy based on dendritic cell-targeted/-derived extracellular vesicles — A novel strategy for enhancement of the anti-tumor immune response. Front Pharmacol. 2019;10:1152. 10.3389/fphar.2019.01152 PMC6798004 31680949

[73] Pascolo S. The messenger’s great message for vaccination. Expert Rev Vaccines. 2014;14(2):153-6. 10.1586/14760584.2015.1000871 25586101

[74] Li WH, Li YM. Chemical strategies to boost cancer vaccines. Chem Rev. 2020;120(20):11420-78. 10.1021/acs.chemrev.9b00833 32914967

[75] Morozova O V., Isaeva EI, Klinov D V. Protein nanoparticles with enzymatic and antigen-binding activities induce th1 cytokine gene expression. Mater Sci Forum. 2020;995:109–13.

[76] Monslow MA, Elbashir S, Sullivan NL, Thiriot DS, Ahl P, Smith J (2020). Immunogenicity generated by mRNA vaccine encoding VZV gE antigen is comparable to adjuvanted subunit vaccine and better than live attenuated vaccine in nonhuman primates. Vaccine.

[77] Castillo-Hair SM, Seelig G (2022). Machine Learning for Designing Next-Generation mRNA Therapeutics. Acc Chem Res.

[78] Zhang X, Lu N, Wang L, Wang Y, Li M, Zhou Y, et al. Recent advances of m6A methylation modification in esophageal squamous cell carcinoma. Cancer Cell Int. 2021;21(1):1-9. 10.1186/s12935-021-02132-2 PMC8353866 34376206

[79] Jia L, Mao Y, Ji Q, Dersh D, Yewdell JW, Qian SB (2020). Decoding mRNA translatability and stability from the 5′ UTR. Nat Struct Mol Biol.

[80] Fuchs AL, Neu A, Sprangers R (2016). A general method for rapid and cost-efficient large-scale production of 5’ capped RNA. RNA.

[81] Griesbach E, Schlackow M, Marzluff WF, Proudfoot NJ. Dual RNA 3’-end processing of H2A.X messenger RNA maintains DNA damage repair throughout the cell cycle. Nat Commun. 2021;12(1):359. 10.1038/s41467-020-20520-6 PMC7807067 33441544

[82] Bhandari BK, Lim CS, Remus DM, Chen A, van Dolleweerd C, Gardner PP. Analysis of 11,430 recombinant protein production experiments reveals that protein yield is tunable by synonymous codon changes of translation initiation sites. PLoS Comput Biol. 2021;17(10):e1009461. 10.1371/journal.pcbi.1009461 PMC8519471 34610008

[83] Garin S, Levi O, Forrest ME, Antonellis A, Arava YS (2021). Comprehensive characterization of mRNAs associated with yeast cytosolic aminoacyl-tRNA synthetases. RNA Biol.

[84] Wehrkamp CJ, Natarajan SK, Mohr AM, Phillippi MA, Mott JL (2018). miR-106b-responsive gene landscape identifies regulation of Kruppel-like factor family. RNA Biol.

[85] Herzog VA, Fasching N, Ameres SL (2020). Determining mRNA Stability by Metabolic RNA Labeling and Chemical Nucleoside Conversion. Methods Mol Biol.

[86] Tangye SG, Burnett DL, Bull RA. Getting to the (germinal) center of humoral immune responses to SARS-CoV-2. Cell. 2022;185(6):945-8. 10.1016/j.cell.2022.02.018 PMC8928419 35303427

[87] Leppek K, Byeon GW, Kladwang W, Wayment-Steele HK, Kerr CH, Xu AF, et al. Combinatorial optimization of mRNA structure, stability, and translation for RNA-based therapeutics. Nat Commun. 2022;13(1):1536. 10.1038/s41467-022-28776-w PMC8940940 35318324

[88] Ozay EI, Dunbar P, Volk K, Maloney MF, Yee C, Mosaheb M, et al. Abstract 1525: Enhancing potency of antigen presenting cells via signal 2/3 mRNA engineering through Cell Squeeze® technology. Cancer Res. 2021;81:1525.

[89] Watermann C, Pasternack H, Idel C, Ribbat-Idel J, Bragelmann J, Kuppler P (2021). Recurrent HNSCC harbor an immunosuppressive tumor immune microenvironment suggesting successful tumor immune evasion. Clin Cancer Res.

[90] Xu L, Tudor D, Bomsel M. The Protective HIV-1 Envelope gp41 Antigen P1 Acts as a Mucosal Adjuvant Stimulating the Innate Immunity. Front Immunol. 2021;11:599278. 10.3389/fimmu.2020.599278 PMC7886812 33613520

[91] Loughrey D, Dahlman JE (2022). Non-liver mRNA Delivery. Acc Chem Res.

[92] Levanova AA, Lampi M, Kalke K, Hukkanen V, Poranen MM, Eskelin K. Native RNA Purification Method for Small RNA Molecules Based on Asymmetrical Flow Field-Flow Fractionation. Pharmaceuticals. 2022;15(2):261. 10.3390/ph15020261 PMC8876226 35215370

[93] Devarkar SC, Schweibenz B, Wang C, Marcotrigiano J, Patel SS (2018). RIG-I Uses an ATPase-Powered Translocation-Throttling Mechanism for Kinetic Proofreading of RNAs and Oligomerization. Mol Cell.

[94] Bosso M, Kirchhoff F. Emerging Role of PYHIN Proteins as Antiviral Restriction Factors. Viruses. 2020;12(12):1464. 10.3390/v12121464 PMC7767131 33353088

[95] Mu X, Hur S (2021). Immunogenicity of in Vitro-Transcribed RNA. Acc Chem Res.

[96] Bresson S, Shchepachev V, Spanos C, Turowski TW, Rappsilber J, Tollervey D (2020). Stress-Induced Translation Inhibition through Rapid Displacement of Scanning Initiation Factors. Mol Cell.

[97] Vlatkovic I, Ludwig J, Boros G, Szabó GT, Reichert J, Buff M, et al. Ribozyme Assays to Quantify the Capping Efficiency of In Vitro-Transcribed mRNA. Pharmaceutics. 2022;14(2):328. 10.3390/pharmaceutics14020328 PMC8879150 35214060

[98] Koyakutty M, Ramesh NA, Nambiar A, Anna NNA, Abraham MC, Anoop M, et al. Abstract B116: Nano-neo-mRNA vaccine: A novel platform technology for cancer-immunotherapy. 2019;7(2_Supplement):B116.

[99] Jackson NAC, Kester KE, Casimiro D, Gurunathan S, DeRosa F. The promise of mRNA vaccines: a biotech and industrial perspective. NPJ Vacc. 2020;5(1):11. 10.1038/s41541-020-0159-8 PMC7000814 32047656

[100] Ji X, Meng W, Liu Z, Mu X. Emerging Roles of lncRNAs Regulating RNA-Mediated Type-I Interferon Signaling Pathway. Front Immunol. 2022;13. 10.3389/fimmu.2022.811122 PMC8914027 35280983

[101] Obermann HL, Lederbogen II, Steele J, Dorna J, Sander LE, Engelhardt K, et al. RNA-Cholesterol Nanoparticles Function as Potent Immune Activators via TLR7 and TLR8. Front Immunol. 2022;12:5936. 10.3389/fimmu.2021.658895 PMC8814444 35126343

[102] Uematsu A, Kajino R, Maeda Y, Ueno Y (2020). Synthesis and characterization of 4′-C-guanidinomethyl-2′-O-methyl-modified RNA oligomers. Nucleosides, Nucleotides Nucleic Acids.

[103] Dörrie J, Schaft N, Schuler G, Schuler-Thurner B. Therapeutic cancer vaccination with ex vivo rna-transfected dendritic cells—an update. Pharmaceutics. 2020;12(2):92. 10.3390/pharmaceutics12020092 PMC7076681 31979205

[104] MacNamara KC (2019). The Role of Interferon, Inflammation and Infection in Aplastic Anemia. Blood..

[105] Kiddane AT, Kang MJ, Ho TC, Getachew AT, Patil MP, Chun BS (2022). Anticancer and Apoptotic Activity in Cervical Adenocarcinoma HeLa Using Crude Extract of Ganoderma applanatum. Curr Issues Mol Biol.

[106] Zhang T, Zhang SW, Zhang SY, Gao SJ, Chen Y, Huang Y. M6A-express: Uncovering complex and condition-specific m6A regulation of gene expression. Nucleic Acids Res. 2021;49(20):e116. 10.1093/nar/gkab714 PMC8599805 34417605

[107] Chen X, Liu Z, Gu Y, Zhang Y, Liu Y, Wang L, et al. A hexokinase from the oyster Crassostrea gigas is involved in immune recognition as a pattern recognition receptor. Dev Comp Immunol. 2021;122:104083. 10.1016/j.dci.2021.104083 33930456

[108] Salleh MZ, Norazmi MN, Deris ZZ. Immunogenicity mechanism of mRNA vaccines and their limitations in promoting adaptive protection against SARS-CoV-2. PeerJ. 2022;10:e13083. 10.7717/peerj.13083 PMC8917804 35287350

[109] Lee Y, Maes R, Tai SHS, Soboll HG (2019). Viral replication and innate immunity of feline herpesvirus-1 virulence-associated genes in feline respiratory epithelial cells. Virus Res.

[110] Park HJ, Ko HL, Won DH, Hwang D Bin, Shin YS, Kwak HW, et al. Comprehensive analysis of the safety profile of a single-stranded RNA nano-structure adjuvant. Pharmaceutics. 2019;11(9):464. 10.3390/pharmaceutics11090464 PMC6781302 31500241

[111] De Jong W, Aerts J, Allard S, Brander C, Buyze J, Florence E, et al. IHIVARNA phase IIa, a randomized, placebo-controlled, double-blinded trial to evaluate the safety and immunogenicity of iHIVARNA-01 in chronically HIV-infected patients under stable combined antiretroviral therapy. Trials. 2019;20:1-0. 10.1186/s13063-019-3409-1 PMC6580477 31208472

[112] Lombardi A, Bozzi G, Ungaro R, Villa S, Castelli V, Mangioni D, et al. Mini Review Immunological Consequences of Immunization With COVID-19 mRNA Vaccines: Preliminary Results. Front. Immunol. 2021;12:657711. 10.3389/fimmu.2021.657711 PMC7994748 33777055

[113] Mund SJK, Macphee DJ, Campbell J, Honaramooz A, Wobeser B, Barber SM. Macroscopic, histologic, and immunomodulatory response of limb wounds following intravenous allogeneic cord blood-derived multipotent mesenchymal stromal cell therapy in horses. Cells. 2021;10(11):2972. 10.3390/cells10112972 PMC8616408 34831196

[114] Liu Q, Zhu H, Tiruthani K, Shen L, Chen F, Gao K (2018). Nanoparticle-Mediated Trapping of Wnt Family Member 5A in Tumor Microenvironments Enhances Immunotherapy for B-Raf Proto-Oncogene Mutant Melanoma. ACS Nano.

[115] Gregg JR, Thompson TC. Considering the potential for gene-based therapy in prostate cancer. Nat. Rev. Urol. 2021;18:170–84. 10.1038/s41585-021-00431-x 33637962

[116] Medina-Magües LG, Gergen J, Jasny E, Petsch B, Lopera-Madrid J, Medina-Magües ES, et al. Mrna vaccine protects against zika virus. Vaccines. 2021;9(12):1464. 10.3390/vaccines9121464 PMC8707647 34960211

[117] Kim BJ, Jeong H, Seo H, Lee MH, Shin HM, Kim BJ. Recombinant Mycobacterium paragordonae Expressing SARS-CoV-2 Receptor-Binding Domain as a Vaccine Candidate Against SARS-CoV-2 Infections. Front Immunol. 2021;12:712274. 10.3389/fimmu.2021.712274 PMC8432291 34512635

[118] Chatterjee SK, Saha S, Munoz MNM. Activation of mucosal immunity and novel prophylactic and therapeutic strategy in combating COVID-19. Explor Immunol. 2021;1:374–97.

[119] Mauriello A, Manolio C, Cavalluzzo B, Avallone A, Borrelli M, Morabito A, et al. Immunological effects of adjuvants in subsets of antigen presenting cells of cancer patients undergoing chemotherapy. J Transl Med. 2020;18:1-7. 10.1186/s12967-020-02218-x PMC6977281 31973714

[120] Chen Q, Zhang Y, Yin H. Recent advances in chemical modifications of guide RNA, mRNA and donor template for CRISPR-mediated genome editing. Adv. Drug Deliv. Rev. 2021;168:246-58. 10.1016/j.addr.2020.10.014 33122087

[121] Ramachandran S, Satapathy SR, Dutta T. Delivery Strategies for mRNA Vaccines. Pharmaceut. Med. 2022;36(1):11-20. 10.1007/s40290-021-00417-5 PMC8801198 35094366

[122] Wei R, Qi G, Zeng Z, Shen N, Wang Z, Shen H, et al. IMUP and GPRC5A: two newly identified risk score indicators in pancreatic ductal adenocarcinoma. Cancer Cell Int. 2021;21:1-9. 10.1186/s12935-021-02324-w PMC8613923 34819098

[123] Heine A, Juranek S, Brossart P. Clinical and immunological effects of mRNA vaccines in malignant diseases. Mol Cancer. 2021;20(1):1-20. 10.1186/s12943-021-01339-1 PMC7957288 33722265

[124] Vogt A, Sadeghlar F, Ayub TH, Schneider C, Möhring C, Zhou T, et al. Alpha-fetoprotein-and CD40ligand-expressing dendritic cells for immunotherapy of hepatocellular carcinoma. Cancers (Basel). 2021;13(13):3375. 10.3390/cancers13133375 PMC8269346 34282787

[125] Taniguchi T, Endo K ichi, Tanioka H, Sasaoka M, Tashiro K, Kinoshita S, et al. Novel use of a chemically modified siRNA for robust and sustainable in vivo gene silencing in the retina. Sci Rep. 2020;10(1):1-9. 10.1038/s41598-020-79242-w PMC7749170 33339841

[126] Pardi N, Hogan MJ, Porter FW, Weissman D. mRNA vaccines-a new era in vaccinology. Nat. Rev. Drug Discov. 2018;17(4):261-79. 10.1038/nrd.2017.243 PMC5906799 29326426

[127] DiTommaso T, Cole JM, Cassereau L, Buggé JA, Sikora Hanson JL, Bridgen DT (2018). Cell engineering with microfluidic squeezing preserves functionality of primary immune cells in vivo. Proc Natl Acad Sci U S A.

[128] Aldosari BN, Alfagih IM, Almurshedi AS. Lipid nanoparticles as delivery systems for RNA-based vaccines. Pharmaceutics. 2021;13(2):206. 10.3390/pharmaceutics13020206 PMC7913163 33540942

[129] Ritthipichai K, Machin M, Juillerat A, Poirot L, Fardis M, Chartier C (2020). 1052P Genetic modification of Iovance’s TIL through TALEN-mediated knockout of PD-1 as a strategy to empower TIL therapy for cancer. Ann Oncol.

[130] Dewitte H, Verbeke R, Breckpot K, Vandenbroucke RE, Libert C, De Smedt SC (2014). Choose your models wisely: How different murine bone marrow-derived dendritic cell protocols influence the success of nanoparticulate vaccines in vitro. J Control Release.

[131] Fornaguera C, Díaz-Caballero M, García-Fernandez C, Olmo L, Pinto MSL, Navalón-López M, et al. Synthesis and characterization of mrna-loaded poly(Beta aminoesters) nanoparticles for vaccination purposes. J Vis Exp. 2021;13(174):e62889. 10.3791/62889 34459811

[132] Mai Y, Guo J, Zhao Y, Ma S, Hou Y, Yang J. Intranasal delivery of cationic liposome-protamine complex mRNA vaccine elicits effective anti-tumor immunity. Cell Immunol. 2020;354:104143. 10.1016/j.cellimm.2020.104143 32563850

[133] Mbatha LS, Maiyo F, Daniels A, Singh M. Dendrimer-Coated Gold Nanoparticles for Efficient Folate-Targeted mRNA Delivery In Vitro. Pharmaceutics. 2021;13(6):900. 10.3390/pharmaceutics13060900 PMC8235267 34204271

[134] He S, Xia S, Song X, Huang H, Wang X, Jiang X (2020). Investigating the Fate of MP1000-LPX In Vivo by Adding Serum to Transfection Medium. Pharm Nanotechnol.

[135] Shiga M, Saito M, Hattori M, Torii C, Kosaki K, Kiyono T (2007). Characteristic phenotype of immortalized periodontal cells isolated from a Marfan syndrome type I patient\rAssessing ageing of individual T lymphocytes: Mission impossible?\rFacile Synthesis of Naphthoquinone Spiroketals by Diastereoselective Oxidative [3. Cell Tissue Res.

[136] Chitosan TG, Response PSTLRDI (2015). Turkish. J Immunol.

[137] Simpson I. Therapeutic delivery: Industry update covering January 2019. Ther. Deliv. 2019;10(5):273-80.

[138] El Khatib M, Mauro A, Di Mattia M, Wyrwa R, Schweder M, Ancora M, et al. Electrospun PLGA Fiber Diameter and Alignment of Tendon Biomimetic Fleece Potentiate Tenogenic Differentiation and Immunomodulatory Function of Amniotic Epithelial Stem Cells. Cells. 2020;9(5):1207. 10.3390/cells9051207 PMC7290802 32413998

[139] Du GQ, Shao ZB, Wu J, Yin WJ, Li SH, Wu J, et al. Targeted myocardial delivery of GDF11 gene rejuvenates the aged mouse heart and enhances myocardial regeneration after ischemia–reperfusion injury. Basic Res Cardiol. 2017;112:1-4. 10.1007/s00395-016-0593-y 28004242

[140] Mair L, Ford K, Alam MR, Kole R, Fisher M, Superfine R (2009). Size-uniform 200 nm particles: Fabrication and application to magnetofection. J Biomed Nanotechnol.

[141] Davis ME, Zuckerman JE, Choi CHJ, Seligson D, Tolcher A, Alabi CA, et al. Evidence of RNAi in humans from systemically administered siRNA via targeted nanoparticles. Nature. 2010;464(7291):1067-70. 10.1038/nature08956 PMC2855406 20305636

[142] Zhang B, Li A, Zuo F, Yu R, Zeng Z, Ma H, Chen S. Recombinant Lactococcus lactis NZ9000 secretes a bioactive kisspeptin that inhibits proliferation and migration of human colon carcinoma HT-29 cells. Microb Cell Fact. 2016;15(1):1-1. 10.1186/s12934-016-0506-7 PMC4901401 27287327

[143] Soliman OY, Alameh MG, De Cresenzo G, Buschmann MD, Lavertu M (2020). Efficiency of Chitosan/Hyaluronan-Based mRNA Delivery Systems In Vitro: Influence of Composition and Structure. J Pharm Sci.

[144] Kim J, Vaughan HJ, Zamboni CG, Sunshine JC, Green JJ (2021). High-throughput evaluation of polymeric nanoparticles for tissue-targeted gene expression using barcoded plasmid DNA. J Control Release.

[145] Balachander GM, Rajashekar B, Sarashetti PM, Rangarajan A, Chatterjee K (2018). MiRNomics Reveals Breast Cancer Cells Cultured on 3D Scaffolds Better Mimic Tumors in Vivo than Conventional 2D Culture. ACS Biomater Sci Eng..

[146] Kiesgen S, Linot C, Quach HT, Saini J, Bellis R, Banerjee S (2020). Abstract LB-378: Regional delivery of clinical-grade mesothelin-targeted CAR T cells with cell-intrinsic PD-1 checkpoint blockade: Translation to a phase I trial. Cancer Res..

[147] Wilbie D, Walther J, Mastrobattista E (2019). Delivery Aspects of CRISPR/Cas for in Vivo Genome Editing. Acc Chem Res.

[148] Woess K, Sun Y, Morio H, Stierschneider A, Kaufmann A, Hainzl S, et al. Evaluating a Targeted Cancer Therapy Approach Mediated by RNA trans-Splicing In Vitro and in a Xenograft Model for Epidermolysis Bullosa-Associated Skin Cancer. Int J Mol Sci. 2022;23(1):575. 10.3390/ijms23010575 PMC8745581 35008999

[149] Carpi S, Fogli S, Giannetti A, Adinolfi B, Tombelli S, Da Pozzo E, et al. Theranostic properties of a survivin-directed molecular beacon in human melanoma cells. PLoS One. 2014;9(12):e114588. 10.1371/journal.pone.0114588 PMC4263748 25501971

[150] Jarrell JA, Twite AA, Lau KHWJ, Kashani MN, Lievano AA, Acevedo J, et al. Intracellular delivery of mRNA to human primary T cells with microfluidic vortex shedding. Sci Rep. 2019;9(1):3214. 10.1038/s41598-019-40147-y PMC6397276 30824814

[151] Ho TD, Starnbach MN, Ho S, Winkler-Lowen B, Morrish DW, Dakour J (2004). Interferon-alpha-mediated prevention of in vitro apoptosis of chronic lymphocytic leukemia B cells: role of bcl-2 and c-myc. Infect Immun.

[152] Chernikov IV, Vlassov VV, Chernolovskaya EL. Current development of siRNA bioconjugates: From research to the clinic. Front. Pharmacol. 2019:444. 10.3389/fphar.2019.00444 PMC6498891 31105570

[153] Jenneman GE, McInerney MJ, Crocker ME, Knapp RM, Alumina S, Smirnova I (2014). Preparation of Silica Aerogel by Ambient Pressure Drying Process using Rice Husk Ash as Raw Material BOILING IN SODIUM FILTRATION. Biomaterials..

[154] Kim EJ, Shim G, Kim K, Kwon IC, Oh YK, Shim CK (2009). Hyaluronic acid complexed to biodegradable poly L-arginine for targeted delivery of siRNAs. J Gene Med.

[155] Gaspar R, Coelho F, Silva BFB. Lipid-nucleic acid complexes: physicochemical aspects and prospects for cancer treatment. Molecules. 2020;25(21):5006. 10.3390/molecules25215006 PMC7662579 33126767

[156] Subhan MA, Torchilin VP. Efficient nanocarriers of siRNA therapeutics for cancer treatment. Transl. Res. 2019;214:62-91. 10.1016/j.trsl.2019.07.006 31369717

[157] Billingsley MM, Singh N, Ravikumar P, Zhang R, June CH, Mitchell MJ (2020). Ionizable Lipid Nanoparticle-Mediated mRNA Delivery for Human CAR T Cell Engineering. Nano Lett.

[158] Nitzlaff S, Bangel-Ruland N, Hoffmann S, Goycoolea FM, Weber WM. Tracking the uptake of labeled chitosan wtCFTR-mRNA complexes in human bronchial epithelial cells. Acta Physiol. 2017;219:112–3.

[159] Lou G, Song X, Yang F, Wu S, Wang J, Chen Z, et al. Exosomes derived from MIR-122-modified adipose tissue-derived MSCs increase chemosensitivity of hepatocellular carcinoma. J Hematol Oncol. 2015;8(1):1-1. 10.1186/s13045-015-0220-7 PMC4627430 26514126

[160] Sun W, Xing C, Zhao L, Zhao P, Yang G, Yuan L (2020). Ultrasound Assisted Exosomal Delivery of Tissue Responsive mRNA for Enhanced Efficacy and Minimized Off-Target Effects. Mol Ther - Nucleic Acids.

[161] Vu V, Liu Y, Sen S, Xu A, Sweeney G. Delivery of adiponectin gene to skeletal muscle using ultrasound targeted microbubbles improves insulin sensitivity and whole body glucose homeostasis. Am J Physiol - Endocrinol Metab. 2013;304(2):E168-75. 10.1152/ajpendo.00493.2012 PMC3543570 23132298

[162] Yang Z, Shi J, Xie J, Wang Y, Sun J, Liu T (2020). Large-scale generation of functional mRNA-encapsulating exosomes via cellular nanoporation. Nat Biomed Eng.

[163] Wang Y, Huang G, Yang H, Zhang XN. Advances on nonviral vectors of CRISPR/Cas9 system for genome editing. Yaoxue Xuebao. 2020:2606-17.

[164] Quijano E, Liu Y, Squinto S, Turner BC, Glazer PM (2021). Abstract LB169: Systemic Administration of an antibody/RNA complex results in tumor specific delivery of immunostimulatory RNAs and tumor growth suppression in a mouse model of melanoma. Cancer Res..

[165] Jing H, Cheng W, Li S, Wu B, Leng X, Xu S (2016). Novel cell-penetrating peptide-loaded nanobubbles synergized with ultrasound irradiation enhance EGFR siRNA delivery for triple negative Breast cancer therapy. Colloids Surfaces B Biointerfaces.

[166] Schetters STT, Rodriguez E, Kruijssen LJW, Crommentuijn MHW, Boon L, Van Den Bossche J, et al. Monocyte-derived APCs are central to the response of PD1 checkpoint blockade and provide a therapeutic target for combination therapy. J Immunother Cancer. 2020;8(2). 10.1136/jitc-2020-000588 PMC7371367 32690667

[167] Lacher MD, Bauer G, Fury B, Graeve S, Fledderman EL, Petrie TD, et al. SV-BR-1-GM, a clinically effective GM-CSF-secreting breast cancer cell line, expresses an immune signature and directly activates CD4+ T lymphocytes. Front Immunol. 2018;9:776. 10.3389/fimmu.2018.00776 PMC5962696 29867922

[168] Bošnjak B, Do KTH, Förster R, Hammerschmidt SI. Imaging dendritic cell functions*. Immunol. Rev. 2022;306(1):137-63. 10.1111/imr.13050 34859450

[169] Shi Y, Lu Y, Qin B, Jiang M, Guo X, Li X, et al. Antigen transfer from non-APCs to APCs impacts the efficacy and safety of protein- and mRNA- based vaccines. Nano Today. 2021;41:101326.

[170] Copur MS. Messenger RNA Vaccines: Beckoning of a New Era in Cancer Immunotherapy. Oncol. (United States). 2021;35(4):190–8. 10.46883/ONC.2021.3504.0198 33893760

[171] Ayad C, Libeau P, Lacroix-Gimon C, Ladavière C, Verrier B. Lipoparticles: Lipid-coated pla nanoparticles enhanced in vitro mrna transfection compared to liposomes. Pharmaceutics. 2021;13(3):377. 10.3390/pharmaceutics13030377 PMC7999670 33809164

[172] Wang QT, Nie Y, Sun SN, Lin T, Han RJ, Jiang J (2020). Tumor-associated antigen-based personalized dendritic cell vaccine in solid tumor patients. Cancer Immunol Immunother.

[173] Zhong Z, Portela Catani JP, McCafferty S, Couck L, Van Den Broeck W, Gorlé N, et al. Immunogenicity and protection efficacy of a naked self-replicating mrna-based zika virus vaccine. Vaccines. 2019;7(3):96. 10.3390/vaccines7030096 PMC6789535 31450775

[174] Burris HA, Patel MR, Cho DC, Clarke JM, Gutierrez M, Zaks TZ, et al. A phase I multicenter study to assess the safety, tolerability, and immunogenicity of mRNA-4157 alone in patients with resected solid tumors and in combination with pembrolizumab in patients with unresectable solid tumors. J Clin Oncol. 2019;37:2523.

[175] Zhang G, Chitkushev L, Olsen LR, Keskin DB, Brusic V. TANTIGEN 2.0: a knowledge base of tumor T cell antigens and epitopes. BMC Bioinformatics. 2021;22:1-8. 10.1186/s12859-021-03962-7 PMC8045306 33849445

[176] Gopanenko A V., Kosobokova EN, Kosorukov VS. Main strategies for the identification of neoantigens. Cancers (Basel). 2020;12(10):2879. 10.3390/cancers12102879 PMC7600129 33036391

[177] Cheng F, Liang H, Butte AJ, Eng C, Nussinov R. Personal mutanomes meet modern oncology drug discovery and precision health. Pharmacol. Rev. 2019;71(1):1-9. 10.1124/pr.118.016253 PMC6294046 30545954

[178] Leclerc M, Mezquita L, De Nerville GG, Tihy I, Malenica I, Chouaib S, et al. Recent advances in lung cancer immunotherapy: Input of T-cell epitopes associated with impaired peptide processing. Front Immunol. 2019;10:1505. 10.3389/fimmu.2019.01505 PMC6616108 31333652

[179] Hanada K, Zhao C, Gil-Hoyos R, Gartner J, Chow-Parmer C, Lowery F, et al. Abstract 1509: A signature for tumor neoantigen-reactive T-cells in fresh human lung cancers allows rapid cloning of their receptors. Cancer Res. 2021;81:1509.

[180] Liang Y, Zhang J, Yuan RY, Wang MY, He P, Su JG, et al. Design of a mutation-integrated trimeric RBD with broad protection against SARS-CoV-2. Cell Discov. 2022;8(1):17. 10.1038/s41421-022-00383-5 PMC8847466 35169113

[181] Driscoll CB, Schuelke MR, Kottke T, Thompson JM, Wongthida P, Tonne JM, et al. APOBEC3B-mediated corruption of the tumor cell immunopeptidome induces heteroclitic neoepitopes for cancer immunotherapy. Nat Commun. 2020;11(1):790. 10.1038/s41467-020-14568-7 PMC7005822 32034147

[182] Cafri G, Gartner JJ, Zaks T, Hopson K, Levin N, Paria BC (2020). mRNA vaccine–induced neoantigen-specific T cell immunity in patients with gastrointestinal cancer. J Clin Invest.

[183] Stukan AI, Goryainova AY, Riger NA, Sharov SV, Shatokhina AS, Chukhray OY (2021). Germinal BRCA-mutation significance in the tumor microenvironment formation Efficacy of PARP inhibition in late-line therapy of metastatic castration-resistant prostate cancer. Onkourologiya.

[184] Advani D, Sharma S, Kumari S, Ambasta RK, Kumar P (2021). Precision Oncology, Signaling, and Anticancer Agents in Cancer Therapeutics. Anticancer Agents Med Chem.

[185] Aghaei H, Farhadi E, Akhtari M, Shahba S, Mostafaei S, Jamshidi A, et al. Copy number variation of IL17RA gene and its association with the ankylosing spondylitis risk in Iranian patients: a case-control study. BMC Med Genet. 2020;21:1-8. 10.1186/s12881-020-01078-y PMC7350761 32650733

[186] Weissenberger M, Weissenberger MH, Gilbert F, Groll J, Evans CH, Steinert AF. Reduced hypertrophy in vitro after chondrogenic differentiation of adult human mesenchymal stem cells following adenoviral SOX9 gene delivery. BMC Musculoskelet Disord. 2020;21:1-4. 10.1186/s12891-020-3137-4 PMC7026978 32066427

[187] Trempe F, Rossotti MA, Maqbool T, MacKenzie CR, Arbabi-Ghahroudi M. Llama DNA Immunization and Isolation of Functional Single-Domain Antibody Binders. Methods Mol Biol. 2022;37–70. 10.1007/978-1-0716-2075-5_3 35157268

[188] Schmidt T, Klemis V, Schub D, Mihm J, Hielscher F, Marx S (2021). Immunogenicity and reactogenicity of heterologous ChAdOx1 nCoV-19/mRNA vaccination. Nat Med.

[189] Riwaldt S, Corydon TJ, Pantalone D, Sahana J, Wise P, Wehland M, et al. Role of Apoptosis in Wound Healing and Apoptosis Alterations in Microgravity. Front. Bioeng. Biotechnol. 2021;9:679650. 10.3389/fbioe.2021.679650 PMC8248797 34222218

[190] Siewert CD, Haas H, Cornet V, Nogueira SS, Nawroth T, Uebbing L, et al. Hybrid Biopolymer and Lipid Nanoparticles with Improved Transfection Efficacy for mRNA. Cells. 2020;9(9):2034. 10.3390/cells9092034 PMC7563888 32899484

[191] Rahman MM, Zhou N, Huang J. An overview on the development of mrna-based vaccines and their formulation strategies for improved antigen expression in vivo. Vaccines. 2021;9(3):244. 10.3390/vaccines9030244 PMC8001631 33799516

[192] Babaee N, Talebkhan Garoosi Y, Karimipoor M, Davami F, Bayat E, Safarpour H (2020). DARPin Ec1-LMWP protein scaffold in targeted delivery of siRNA molecules through EpCAM cancer stem cell marker. Mol Biol Rep.

[193] Hansen SV, Blum MK, Abildgaard N, Nyvold CG (2020). Efficient, Non-Viral and Reproducible Protocol for Stable Knockdown of Genes in Mantle Cell Lymphoma Cell Lines. Blood.

[194] Leventhal SS, Clancy C, Erasmus J, Feldmann H, Hawman DW. An intramuscular dna vaccine for sars-cov-2 decreases viral lung load but not lung pathology in syrian hamsters. Microorganisms. 2021;9(5):1040. 10.3390/microorganisms9051040 PMC8151856 34065996

[195] Lakshmikanthan S, Jedinak A, Maxwell M, Kadiyala M, Yan D, Cardia J, et al. Abstract 2239: Intratumoral delivery of mPH-804 (TIGIT targeting INTASYL compound) inhibits tumor growth and confers an inflammatory tumor microenvironment. Cancer Res. 2020;80:2239.

[196] Lokugamage MP, Vanover D, Beyersdorf J, Hatit MZC, Rotolo L, Echeverri ES (2021). Optimization of lipid nanoparticles for the delivery of nebulized therapeutic mRNA to the lungs. Nat Biomed Eng.

[197] Paramasivam P, Stöter M, Corradi E, Costa ID, Höijer A, Bartesaghi S (2022). Quantitative intracellular retention of delivered RNAs through optimized cell fixation and immunostaining. RNA.

[198] Carvalheiro M, Ferreira-Silva M, Holovanchuk D, Marinho HS, Moreira JN, Soares H, et al. Antagonist G-targeted liposomes for improved delivery of anticancer drugs in small cell lung carcinoma. Int J Pharm. 2022;612:121380. 10.1016/j.ijpharm.2021.121380 34915142

[199] Scicchitano P, Milo M, Mallamaci R, De Palo M, Caldarola P, Massari F, et al. Inclisiran in lipid management: A Literature overview and future perspectives. Biomed Pharmacother. 2021;143:112227. 10.1016/j.biopha.2021.112227 34563953

[200] Lokugamage MP, Gan Z, Zurla C, Levin J, Islam FZ, Kalathoor S, et al. Mild Innate Immune Activation Overrides Efficient Nanoparticle-Mediated RNA Delivery. Adv Mater. 2020;32(1):1904905. 10.1002/adma.201904905 PMC7029413 31743531

[201] Saunders KO, Pardi N, Parks R, Santra S, Mu Z, Sutherland L, et al. Lipid nanoparticle encapsulated nucleoside-modified mRNA vaccines elicit polyfunctional HIV-1 antibodies comparable to proteins in nonhuman primates. NPJ Vaccines. 2021;6(1):50. 10.1038/s41541-021-00307-6 PMC8035178 33837212

[202] Chen CY, Tran DM, Cavedon A, Cai X, Rajendran R, Lyle MJ (2020). Treatment of Hemophilia A Using Factor VIII Messenger RNA Lipid Nanoparticles. Mol Ther - Nucleic Acids.

[203] Lewis PE, Poteet EC, Liu D, Chen C, Labranche CC, Stanfield-Oakley SA (2020). Ctla-4 blockade, during hiv virus-like particles immunization, alters hiv-specific b-cell responses. Vaccines.

[204] Cui D, Tang Y, Jiang Q, Jiang D, Zhang Y, Lv Y, et al. Follicular Helper T Cells in the Immunopathogenesis of SARS-CoV-2 Infection. Front. Immunol. 2021;12:731100. 10.3389/fimmu.2021.731100 PMC8481693 34603308

[205] Fang E, Liu X, Li M, Zhang Z, Song L, Zhu B, et al. Advances in COVID-19 mRNA vaccine development. Signal Transduct Target Ther. 2022;7(1):94. 10.1038/s41392-022-00950-y PMC8940982 35322018

[206] Giacobino C, Canta M, Fornaguera C, Borrós S, Cauda V. Extracellular vesicles and their current role in cancer immunotherapy. Cancers (Basel). 2021;13(9):2280. 10.3390/cancers13092280 PMC8126043 34068657

[207] Punta M, Jennings VA, Melcher AA, Lise S. The Immunogenic Potential of Recurrent Cancer Drug Resistance Mutations: An In Silico Study. Front Immunol. 2020;11:2576. 10.3389/fimmu.2020.524968 PMC7578429 33133066

[208] Huang Y, Chen Y, Zhou S, Chen L, Wang J, Pei Y, et al. Dual-mechanism based CTLs infiltration enhancement initiated by Nano-sapper potentiates immunotherapy against immune-excluded tumors. Nat Commun. 2020;11;11(1):622. 10.1038/s41467-020-14425-7 PMC6992734 32001695

[209] Tombácz I, Weissman D, Pardi N. Vaccination with Messenger RNA: A Promising Alternative to DNA Vaccination. Methods Mol Biol. 2021;13–31. 10.1007/978-1-0716-0872-2_2 32827130

[210] Hirayama M, Nishimura Y. The present status and future prospects of peptide-based cancer vaccines. Int Immunol. 2016;28(7):319-28. 10.1093/intimm/dxw027 27235694

[211] Khong H, Overwijk WW. Adjuvants for peptide-based cancer vaccines. J. Immunother Cancer. 2016;4:1-1. 10.1186/s40425-016-0160-y PMC5028954 27660710

[212] Li SY, Liu Y, Xu CF, Shen S, Sun R, Du XJ, et al. Restoring anti-tumor functions of T cells via nanoparticle-mediated immune checkpoint modulation. J Control Release. 2016;231:17-28. 10.1016/j.jconrel.2016.01.044 26829099

[213] Trajanoski Z, Maccalli C, Mennonna D, Casorati G, Parmiani G, Dellabona P. Somatically mutated tumor antigens in the quest for a more efficacious patient-oriented immunotherapy of cancer. Cancer Immunol Immunother. 2015;64:99-104. 10.1007/s00262-014-1599-7 PMC11028785 25164877

[214] Peng M, Mo Y, Wang Y, Wu P, Zhang Y, Xiong F, et al. Neoantigen vaccine: An emerging tumor immunotherapy. Mol Cancer. 2019;18(1):1-4. 10.1186/s12943-019-1055-6 PMC6708248 31443694

[215] Cheng S, Xu C, Jin Y, Li Y, Zhong C, Ma J, et al. Artificial Mini Dendritic Cells Boost T Cell–Based Immunotherapy for Ovarian Cancer. Adv Sci. 2020;7(7):1903301. 10.1002/advs.201903301 PMC7141030 32274314

[216] Song H, Su Q, Huang P, Zhang C, Wang W. Self-assembling, self-adjuvanting and fully synthetic peptide nanovaccine for cancer immunotherapy. Smart Mater. Med. 2021;2:237-49.

[217] Tornesello AL, Tagliamonte M, Tornesello ML, Buonaguro FM, Buonaguro L. Nanoparticles to improve the efficacy of peptide-based cancer vaccines. Cancers (Basel). 2020;12(4):1049. 10.3390/cancers12041049 PMC7226445 32340356

[218] Dorosti H, Eslami M, Negahdaripour M, Ghoshoon MB, Gholami A, Heidari R (2019). Vaccinomics approach for developing multi-epitope peptide pneumococcal vaccine. J Biomol Struct Dyn.

[219] Lynn GM, Sedlik C, Baharom F, Zhu Y, Ramirez-Valdez RA, Coble VL (2020). Peptide–TLR-7/8a conjugate vaccines chemically programmed for nanoparticle self-assembly enhance CD8 T-cell immunity to tumor antigens. Nat Biotechnol.

[220] Hainline KM, Shores LS, Votaw NL, Bernstein ZJ, Kelly SH, Fries CN, Madhira MS, Gilroy CA, Chilkoti A, Collier JH. Modular complement assemblies for mitigating inflammatory conditions. Proceedings of the National Academy of Sciences. 2021;118(15):e2018627118. 10.1073/pnas.2018627118 PMC8054013 33876753

[221] Li L, Goedegebuure SP, Gillanders WE. Preclinical and clinical development of neoantigen vaccines. Ann. Oncol. 2017;28:xii11-7. 10.1093/annonc/mdx681 PMC5834106 29253113

[222] Kantoff PW, Schuetz TJ, Blumenstein BA, Michael Glode L, Bilhartz DL, Wyand M (2010). Overall survival analysis of a phase II randomized controlled trial of a poxviral-based PSA-targeted immunotherapy in metastatic castration-resistant prostate cancer. J Clin Oncol.

[223] Yoshikawa T. Third-generation smallpox vaccine strain-based recombinant vaccines for viral hemorrhagic fevers. Vaccine. 2021;39(41):6174-81. 10.1016/j.vaccine.2021.09.001 34521550

[224] Piccaluga PP, Di Guardo A, Lagni A, Lotti V, Diani E, Navari M, et al. COVID-19 Vaccine: Between Myth and Truth. Vaccines. 2022;10(3):349. 10.3390/vaccines10030349 PMC8950941 35334981

[225] Zabaleta N, Dai W, Bhatt U, Hérate C, Maisonnasse P, Chichester JA (2021). An AAV-based, room-temperature-stable, single-dose COVID-19 vaccine provides durable immunogenicity and protection in non-human primates. Cell Host Microbe.

[226] Manara C, Brazzoli M, Piccioli D, Taccone M, D’Oro U, Maione D (2019). Co-administration of GM-CSF expressing RNA is a powerful tool to enhance potency of SAM-based vaccines. Vaccine.

[227] Abbasi S, Uchida S. Multifunctional immunoadjuvants for use in minimalist nucleic acid vaccines. Pharmaceutics. 2021;13(5):644. 10.3390/pharmaceutics13050644 PMC8147386 34062771

[228] Broderick KE, Humeau LM. Enhanced delivery of dna or rna vaccines by electroporation. Methods Mol Biol. 2017:193-200. 10.1007/978-1-4939-6481-9_12 27987151

[229] Lopes A, Bastiancich C, Bausart M, Ligot S, Lambricht L, Vanvarenberg K, et al. New generation of DNA-based immunotherapy induces a potent immune response and increases the survival in different tumor models. J Immunother Cancer. 2021;9(4). 10.1136/jitc-2020-001243 PMC8021892 33795383

[230] Starostina E V., Sharabrin S V., Antropov DN, Stepanov GA, Shevelev GY, Lemza AE, et al. Construction and immunogenicity of modified mrna-vaccine variants encoding influenza virus antigens. Vaccines. 2021;9(5):452. 10.3390/vaccines9050452 PMC8147809 34063689

[231] Papukashvili D, Rcheulishvili N, Liu C, Wang X, He Y, Wang PG. Strategy of developing nucleic acid-based universal monkeypox vaccine candidates. Front. Immunol. 2022;13. 10.3389/fimmu.2022.1050309 PMC9646902 36389680

[232] Eygeris Y, Gupta M, Kim J, Sahay G (2022). Chemistry of Lipid Nanoparticles for RNA Delivery. Acc Chem Res.

[233] Haroun F, Alharbi M, Hong A. Case series on the safety of mRNA COVID19 vaccines in cancer patients undergoing treatment. J Clin Oncol. 2021;39:e14562.

[234] Sharbi-Yunger A, Grees M, Cafri G, Bassan D, Eichmüller SB, Tzehoval E (2019). A universal anti-cancer vaccine: Chimeric invariant chain potentiates the inhibition of melanoma progression and the improvement of survival. Int J Cancer.

[235] Bandola-Simon JM, Roche PA (2019). Dendritic cells dysfunction in tumor-draining lymph nodes. J Immunol..

[236] Moreira A, Erdmann M, Uslu U, Vass V, Schuler G, Schuler-Thurner B. Blood eosinophilia is an on-treatment biomarker in patients with solid tumors undergoing dendritic cell vaccination with autologous tumor-RNA. Pharmaceutics. 2020;12(3):210. 10.3390/pharmaceutics12030210 PMC7150785 32121531

[237] Morrissey ME, Byrne R, Nulty C, McCabe NH, Lynam-Lennon N, Butler CT, et al. The tumour microenvironment of the upper and lower gastrointestinal tract differentially influences dendritic cell maturation. BMC Cancer. 2020;20(1):1-3. 10.1186/s12885-020-07012-y PMC7302160 32552799

[238] Song HY, Han JM, Byun EH, Kim WS, Seo HS, Byun EB. Bombyx batryticatus protein-rich extract induces maturation of dendritic cells and th1 polarization: A potential immunological adjuvant for cancer vaccine. Molecules. 2021;26(2):476. 10.3390/molecules26020476 PMC7831066 33477499

[239] Arance Fernandez AM, Baurain J-F, Vulsteke C, Rutten A, Soria A, Carrasco J, et al. A phase I study (E011-MEL) of a TriMix-based mRNA immunotherapy (ECI-006) in resected melanoma patients: Analysis of safety and immunogenicity. J Clin Oncol. 2019;37:2641.

[240] Li X, Zheng J, Chen S, Meng F dong, Ning J, Sun S lan. Oleandrin, a cardiac glycoside, induces immunogenic cell death via the PERK/elF2α/ATF4/CHOP pathway in breast cancer. Cell Death Dis. 2021;12(4):314. 10.1038/s41419-021-03605-y PMC7990929 33762577

[241] Hu Z, Ott PA, Wu CJ. Towards personalized, tumour-specific, therapeutic vaccines for cancer. Nat. Rev. Immunol. 2018;18(3):168-82. 10.1038/nri.2017.131 PMC6508552 29226910

[242] Liu S, Sirohi K, Verma M, McKay J, Michalec L, Sripada A (2020). Optimal identification of human conventional and nonconventional (CRTH2–IL7Rα–) ILC2s using additional surface markers. J Allergy Clin Immunol.

[243] Gumińska N, Zakryś B, Milanowski R. A New Type of Circular RNA derived from Nonconventional Introns in Nuclear Genes of Euglenids. J Mol Biol. 2021;433(3):166758. 10.1016/j.jmb.2020.166758 33316270

[244] Weide B, Carralot JP, Reese A, Scheel B, Eigentler TK, Hoerr I (2008). Results of the first phase I/II clinical vaccination trial with direct injection of mRNA. J Immunother.

[245] Hervent A-S, Vandekerckhove L, De Keulenaer GW. Neuregulin-1 antagonizes myocardial fibrosis and diastolic dysfunction in angiotensin-II treated mice. Eur Heart J. 2013;34:P2434.

[246] Khranovska N, Skachkova O, Ganul A, Gorbach O, Sovenko V, Svergun N (2013). Results from phase iii trial of dendritic cell based vaccine immunotherapy in patients with-iiia stage non-small-cell lung cancer. J Thorac Oncol.

[247] Pepini T, Pulichino A-M, Carsillo T, Carlson AL, Sari-Sarraf F, Ramsauer K (2017). Induction of an IFN-Mediated Antiviral Response by a Self-Amplifying RNA Vaccine: Implications for Vaccine Design. J Immunol.

[248] Rotterdam J, Thiaucourt M, Schwaab J, Reiter A, Kreil S, Steiner L, et al. Antibody Response to Vaccination with BNT162b2, mRNA-1273, and ChADOx1 in Patients with Myeloid and Lymphoid Neoplasms. Blood. 2021;138:218.

[249] Bai X, Wong CC, Pan Y, Chen H, Liu W, Zhai J, et al. Loss of YTHDF1 in gastric tumors restores sensitivity to antitumor immunity by recruiting mature dendritic cells. J Immunother Cancer. 2022;10(2). 10.1136/jitc-2021-003663 PMC9066370 35193930

[250] Lee M, Shim SY. Inhibitory effects of eriodictyol-7-O-β-d-glucuronide and 5,7-dihydroxy-4-chromene isolated from chrysanthemum zawadskii var. latilobum in FcϵRI-mediated human basophilic KU812F cell activation. Molecules. 2020;25(4):994. 10.3390/molecules25040994 PMC7070965 32102220

[251] Mulder FJ, Klufah F, Janssen FME, Farshadpour F, Willems SM, de Bree R, et al. Presence of Human Papillomavirus and Epstein–Barr Virus, but Absence of Merkel Cell Polyomavirus, in Head and Neck Cancer of Non-Smokers and Non-Drinkers. Front Oncol. 2021;10:560434. 10.3389/fonc.2020.560434 PMC7855709 33552950

[252] Ogasawara S, Kanzaki H, Koroki K, Kanayama K, Maruta S, Kobayashi K, et al. Phase I study of a new concept cancer vaccine composed artificial intelligence (AI)-designed shared-antigen peptides plus combined synergistically activating antigen-specific CTL reaction (CYT001) in patients with advanced hepatocellular carcinoma (CRESCE. J Clin Oncol. 2020;38:TPS595.

[253] Song X, Jiang Y, Zhang W, Elfawal G, Wang K, Jiang D (2022). Transcutaneous tumor vaccination combined with anti-programmed death-1 monoclonal antibody treatment produces a synergistic antitumor effect. Acta Biomater.

[254] Fehervari Z (2021). Intranasal vaccination. Nat Immunol.

[255] Harda K, Szabo Z, Juhasz E, Dezso B, Kiss C, Schally A V., et al. Expression of somatostatin receptor subtypes (SSTR-1–SSTR-5) in pediatric hematological and oncological disorders. Molecules. 2020;25(23):5775. 10.3390/molecules25235775 PMC7730851 33297556

[256] Braun DA, Bakouny Z, Hirsch L, Flippot R, Van Allen EM, Wu CJ, et al. Beyond conventional immune-checkpoint inhibition — novel immunotherapies for renal cell carcinoma. Nat Rev Clin Oncol. 2021;199–214. 10.1038/s41571-020-00455-z PMC8317018 33437048

[257] Liu S, Feng Y, Huang Y, Jiang X, Tang C, Tang F (2021). A GM1 gangliosidosis mutant mouse model exhibits activated microglia and disturbed autophagy. Exp Biol Med.

[258] Chen D, Li X, Li H, Wang K, Tian X. Identification of Immune-Related Prognostic mRNA and lncRNA in Patients with Hepatocellular Carcinoma. J Oncol. 2022;2022. 10.1155/2022/5313149 PMC8752260 35027925

[259] Miloradovic D, Miloradovic D, Markovic BS, Acovic A, Harrell CR, Djonov V, et al. The Effects of Mesenchymal Stem Cells on Antimelanoma Immunity Depend on the Timing of Their Administration. Stem Cells Int. 2020;2020. 10.1155/2020/8842659 PMC7368936 32695181

[260] Miyashita Y, Yoshida T, Takagi Y, Tsukamoto H, Takashima K, Kouwaki T, et al. Circulating extracellular vesicle microRNAs associated with adverse reactions, proinflammatory cytokine, and antibody production after COVID-19 vaccination. NPJ Vaccines. 2022;7(1):16. 10.1038/s41541-022-00439-3 PMC8826357 35136071

[261] Lee MJ, Lee I, Wang K. Recent Advances in RNA Therapy and Its Carriers to Treat the Single-Gene Neurological Disorders. Biomedicines. 2022;10(1):158. 10.3390/biomedicines10010158 PMC8773368 35052837

[262] Lou G, Anderluzzi G, Schmidt ST, Woods S, Gallorini S, Brazzoli M (2020). Delivery of self-amplifying mRNA vaccines by cationic lipid nanoparticles: The impact of cationic lipid selection. J Control Release.

[263] Hattori Y, Saito H, Oku T, Ozaki KI. Effects of sterol derivatives in cationic liposomes on biodistribution and gene-knockdown in the lungs of mice systemically injected with siRNA lipoplexes. Mol Med Rep. 2021;24(2):1-9. 10.3892/mmr.2021.12237 PMC8240178 34165169

[264] Wang WD, Yang XR, Guo MF, Pan ZF, Shang M, Qiu MJ (2021). Up-regulation of BTLA expression in myeloid dendritic cells associated with the treatment outcome of neonatal sepsis. Mol Immunol..

[265] Nikpoor AR, Jaafari MR, Zamani P, Teymouri M, Gouklani H, Saburi E, et al. Cell cytotoxicity, immunostimulatory and antitumor effects of lipid content of liposomal delivery platforms in cancer immunotherapies. A comprehensive in-vivo and in-vitro study. Int J Pharm. 2019;567:118492. 10.1016/j.ijpharm.2019.118492 31271815

[266] Chittamuru S, Murphy TM, Little SA, Taylor AA, Wexler R, Desai L. Abstract P39: Pre-clinical evaluation of NEOS-223, an (S)-valine-thiazole derived peptidomimetic N-heterocycle, as an anticancer agent and P-glycoprotein inhibitor. Clin Cancer Res. 2021;27:P39.

[267] Krzywkowski T, Kühnemund M, Nilsson M (2019). Chimeric padlock and iLock probes for increased efficiency of targeted RNA detection. RNA.

[268] Koopaei NN, Schmittgen TD, Reynolds BA, Azari H. Method for Isolating Extracellular Vesicles from Human Neural Stem Cells Expanded Under Neurosphere Culture. Methods Mol Biol. 2022;87–94. 10.1007/978-1-0716-1783-0_8 34558004

[269] Khawar MB, Hamid SE, Jan T, Abbasi MH, Idnan M, Sheikh N. Diagnostic, prognostic and therapeutic potential of long noncoding RNAs in cancer. Mol Biol Rep. 2022;49(3):2311-9. 10.1007/s11033-022-07180-z 35072835

[270] López-Camacho C, De Lorenzo G, Slon-Campos JL, Dowall S, Abbink P, Larocca RA (2020). Immunogenicity and efficacy of zika virus envelope domain III in DNA, protein, and ChAdOx1 adenoviral-vectored vaccines. Vaccines.

[271] Janssens I, Campillo Davó D, Van den Bos J, De Reu H, Berneman ZN, Wens I (2022). Engineering of regulatory T cells by means of mRNA electroporation in a GMP-compliant manner. Cytotherapy.

[272] Kapoor I, Emam EAF, Shaw A, Varshneya U. Nucleoside diphosphate kinase escalates A-to-C mutations in MutT-deficient strains of Escherichia coli. J Bacteriol. 2020;202(1):e00567-19. 10.1128/JB.00567-19 PMC6932243 31591275

[273] Gurney M, O’Reilly E, Corcoran S, Brophy S, Hardwicke D, Krawczyk J, et al. Tc Buster Transposon Engineered CLL-1 CAR-NK Cells Efficiently Target Acute Myeloid Leukemia. Blood. 2021;138:1725.

[274] Guan S, Darmstädter M, Xu C, Rosenecker J. In vitro investigations on optimizing and nebulization of ivt-mrna formulations for potential pulmonary-based alpha-1-antitrypsin deficiency treatment. Pharmaceutics. 2021;13(8):1281. 10.3390/pharmaceutics13081281 PMC8399093 34452241

[275] Melamed JR, Hajj KA, Chaudhary N, Strelkova D, Arral ML, Pardi N (2022). Lipid nanoparticle chemistry determines how nucleoside base modifications alter mRNA delivery. J Control Release.

[276] Dwijayanti A, Storch M, Stan GB, Baldwin GS (2022). A modular RNA interference system for multiplexed gene regulation. Nucleic Acids Res.

[277] Widom JR, Rai V, Rohlman CE, Walter NG (2019). Versatile transcription control based on reversible dCas9 binding. RNA.

[278] Glez-Vaz J, Azpilikueta A, Olivera I, Cirella A, Teijeira A, Ochoa MC, et al. Soluble CD137 as a dynamic biomarker to monitor agonist CD137 immunotherapies. J Immunother cancer. 2022;82(12_Supplement):628. 10.1136/jitc-2021-003532 PMC8896037 35236742

[279] Moreno H, Kunz S. The protein kinase receptor modulates the innate immune response against tacaribe virus. Viruses. 2021;13. 10.3390/v13071313 PMC8310291 34372519

[280] Acharya D, Sullivan MJ, Duell BL, Eveno T, Schembri MA, Ulett GC. Physical extraction and fast protein liquid chromatography for purifying flagella filament from uropathogenic Escherichia coli for immune assay. Front Cell Infect Microbiol. 2019;9:118. 10.3389/fcimb.2019.00118 PMC6491459 31069177

[281] Taina-González L, de la Fuente M. The Potential of Nanomedicine to Unlock the Limitless Applications of mRNA. Pharmaceutics. 2022;14(2):460. 10.3390/pharmaceutics14020460 PMC8879057 35214191

[282] Platts-Mills JA, Houpt ER, Liu J, Zhang J, Guindo O, Sayinzoga-Makombe N (2021). Etiology and Incidence of Moderate-to-Severe Diarrhea in Young Children in Niger. J Pediatric Infect Dis Soc.

[283] Bajrovic I, Schafer SC, Romanovicz DK, Croyle MA. Novel technology for storage and distribution of live vaccines and other biological medicines at ambient temperature. Sci Adv. 2020;6(10):eaau4819. 10.1126/sciadv.aau4819 PMC7056310 32181330

[284] Zheng Z, Yang X, Yu Q, Li L, Qiao L (2021). The regulating role of miR-494 on HCCR1 in cervical cancer cells. Cell Mol Biol.

[285] Packer M, Gyawali D, Yerabolu R, Schariter J, White P. A novel mechanism for the loss of mRNA activity in lipid nanoparticle delivery systems. Nat Commun. 2021;12(1):6777. 10.1038/s41467-021-26926-0 PMC8608879 34811367

[286] Bharath BC, Balamuralidhara V, Venkatesh MP (2019). Veterinary medicine: Drug approval process in Europe and USA. J Pharm Sci Res.

[287] Bilotta C, Perrone G, Adelfio V, Spatola GF, Uzzo ML, Argo A, et al. COVID-19 Vaccine-Related Thrombosis: A Systematic Review and Exploratory Analysis. Front Immunol. 2021;12:729251. 10.3389/fimmu.2021.729251 PMC8666479 34912330

[288] Levine-Tiefenbrun M, Yelin I, Katz R, Herzel E, Golan Z, Schreiber L (2021). Initial report of decreased SARS-CoV-2 viral load after inoculation with the BNT162b2 vaccine. Nat Med.

[289] Borgognone A, Serna G, Noguera-Julian M, Alonso L, Parera M, Català-Moll F, et al. Performance of 16s metagenomic profiling in formalin-fixed paraffin-embedded versus fresh-frozen colorectal cancer tissues. Cancers (Basel). 2021;13(21):5421. 10.3390/cancers13215421 PMC8582506 34771584

[290] Frati P, La Russa R, Di Fazio N, Del Fante Z, Delogu G, Fineschi V. Compulsory vaccination for healthcare workers in italy for the prevention of sars-cov-2 infection. Vaccines. 2021;9(9):966. 10.3390/vaccines9090966 PMC8473178 34579203

[291] Robertson JS, Loizides U, Adisa A, López de la Rica Manjavacas A, Rodilla V, Strnadova C, et al. International Nonproprietary Names (INN) for novel vaccine substances: A matter of safety. Vaccine. 2022;40(1):21-7. 10.1016/j.vaccine.2021.11.054 PMC8625196 34844820

[292] Ait Benichou S, Jauvin D, De Serres-Bérard T, Pierre M, Ling KK, Bennett CF, et al. Antisense oligonucleotides as a potential treatment for brain deficits observed in myotonic dystrophy type 1. Gene Ther. 2022;29(12):698-709. 10.1038/s41434-022-00316-7 PMC9750879 35075265

[293] Marchini T, Mitre LS, Wolf D. Inflammatory Cell Recruitment in Cardiovascular Disease. Front. Cell Dev Biol. 2021;9:635527. 10.3389/fcell.2021.635527 PMC7930487 33681219

[294] van der Meijden PEJ, Heemskerk JWM. Platelet biology and functions: new concepts and clinical perspectives. Nat. Rev. Cardiol. 2019;16(3):166-79. 10.1038/s41569-018-0110-0 30429532

[295] Zhang P, Narayanan E, Liu Q, Tsybovsky Y, Boswell K, Ding S (2021). A multiclade env–gag VLP mRNA vaccine elicits tier-2 HIV-1-neutralizing antibodies and reduces the risk of heterologous SHIV infection in macaques. Nat Med.

[296] Setlow P, Christie G. Bacterial Spore mRNA – What’s Up With That? Front. Microbiol. 2020;11:596092. 10.3389/fmicb.2020.596092 PMC7649253 33193276

[297] D’haese S, Lacroix C, Garcia F, Plana M, Ruta S, Vanham G, et al. Off the beaten path: Novel mRNA-nanoformulations for therapeutic vaccination against HIV J Control. Release. 2021. 1016–33. 10.1016/j.jconrel.2020.11.009 33181204

[298] Haabeth OAW, Lohmeyer JJK, Sallets A, Blake TR, Sagiv-Barfi I, Czerwinski DK (2021). An mRNA SARS-CoV-2 Vaccine Employing Charge-Altering Releasable Transporters with a TLR-9 Agonist Induces Neutralizing Antibodies and T Cell Memory. ACS Cent Sci.

[299] Shin MD, Shukla S, Chung YH, Beiss V, Chan SK, Ortega-Rivera OA, et al. COVID-19 vaccine development and a potential nanomaterial path forward. Nat Nanotechnol. 2020;15(8):646-55. 10.1038/s41565-020-0737-y 32669664

[300] Zhou A, Shi G, Kang GJ, Xie A, Liu H, Jiang N, et al. RNA binding protein, HuR, regulates SCN5A expression through stabilizing MEF2C transcription factor mRNA. J Am Heart Assoc. 2018;7(9):e007802. 10.1161/JAHA.117.007802 PMC6015277 29678826

[301] Zainol MI Bin, Kawasaki T, Monwan W, Murase M, Sueyoshi T, Kawai T. Innate immune responses through Toll-like receptor 3 require human-antigen-R-mediated Atp6v0d2 mRNA stabilization. Sci Rep. 2019;9(1):1-1. 10.1038/s41598-019-56914-w PMC6938500 31892731

[302] Lambermon MHL, Simpson GG, Wieczorek Kirk DA, Hemmings-Mieszczak M, Klahre U, Filipowicz W (2000). UBP1, a novel hnRNP-like protein that functions at multiple steps of higher plant nuclear pre-mRNA maturation. EMBO J.

[303] Liu L, Yang S, Chen F, Cheng KW. Polysaccharide-Zein Composite Nanoparticles for Enhancing Cellular Uptake and Oral Bioavailability of Curcumin: Characterization, Anti-colorectal Cancer Effect, and Pharmacokinetics. Front Nutr. 2022;9. 10.3389/fnut.2022.846282 PMC8924582 35308263

[304] Zhai J, Bao L, Walduck AK, Dyett BP, Cai X, Li M (2022). Enhancing the photoluminescence and cellular uptake of fluorescent carbon nanodots via cubosome lipid nanocarriers. Nanoscale.

[305] Ma R, Shi L. Trade-off effect of polymeric nano-medicine in anti-cancer drug delivery. Giant. 2021;8:100074.

[306] Meier JL, Montgomery DC, Dervan PB (2012). Enhancing the cellular uptake of Py-Im polyamides through next-generation aryl turns. Nucleic Acids Res.

[307] Kocabas BB, Almacioglu K, Bulut EA, Gucluler G, Tincer G, Bayik D (2020). Dual-adjuvant effect of pH-sensitive liposomes loaded with STING and TLR9 agonists regress tumor development by enhancing Th1 immune response. J Control Release.

[308] Li Z, Zhang L, Tang C, Yin C (2017). Co-Delivery of Doxorubicin and Survivin shRNA-Expressing Plasmid Via Microenvironment-Responsive Dendritic Mesoporous Silica Nanoparticles for Synergistic Cancer Therapy. Pharm Res..

[309] Lacaille-Dubois MA. Updated insights into the mechanism of action and clinical profile of the immunoadjuvant QS-21: A review. Phytomedicine. 2019;60:152905. 10.1016/j.phymed.2019.152905 PMC7127804 31182297

[310] Jensen-Jarolim E, Roth-Walter F, Jordakieva G, Pali-Schöll I (2021). Allergens and Adjuvants in Allergen Immunotherapy for Immune Activation, Tolerance, and Resilience. J Allergy Clin Immunol Pract.

[311] Gu P, Wusiman A, Zhang Y, Cai G, Xu S, Zhu S, et al. Polyethylenimine-coated PLGA nanoparticles-encapsulated Angelica sinensis polysaccharide as an adjuvant for H9N2 vaccine to improve immune responses in chickens compared to Alum and oil-based adjuvants. Vet Microbiol. 2020;251:108894. 10.1016/j.vetmic.2020.108894 33096470

[312] Wang Y, Xie Y, Luo J, Guo M, Hu X, Chen X, et al. Engineering a self-navigated MnARK nanovaccine for inducing potent protective immunity against novel coronavirus. Nano Today. 2021;38:101139. 10.1016/j.nantod.2021.101139 PMC7972805 33758593

[313] Cohen Tervaert JW, Mohazab N, Redmond D, van Eeden C, Osman M. Breast implant illness: scientific evidence of its existence. Expert Rev Clin Immunol. 2022;18(1):15-29. 10.1080/1744666X.2022.2010546 34882509

[314] Nagy NA, de Haas AM, Geijtenbeek TBH, van Ree R, Tas SW, van Kooyk Y, et al. Therapeutic Liposomal Vaccines for Dendritic Cell Activation or Tolerance. Front Immunol. 2021;12:674048. 10.3389/fimmu.2021.674048 PMC8155586 34054859

[315] Li A, Yi M, Qin S, Song Y, Chu Q, Wu K. Activating cGAS-STING pathway for the optimal effect of cancer immunotherapy. J Hematol Oncol. 2019;12(1):1-2. 10.1186/s13045-019-0721-x PMC6444510 30935414

[316] Goggi JL, Hartimath S V., Khanapur S, Ramasamy B, Chin ZF, Cheng P, et al. Imaging Memory T-Cells Stratifies Response to Adjuvant Metformin Combined with αPD-1 Therapy. Int J Mol Sci. 2022;23(21):12892. 10.3390/ijms232112892 PMC9654631 36361684

[317] Webb ES, Liu P, Baleeiro R, Lemoine NR, Yuan M, Wang Y (2018). Immune checkpoint inhibitors in cancer therapy. J Biomed Res.

[318] Zhu Y, Yang Z, Dong Z, Gong Y, Hao Y, Tian L, et al. CaCO3-Assisted Preparation of pH-Responsive Immune-Modulating Nanoparticles for Augmented Chemo-Immunotherapy. Nano-Micro Lett. 2021;13:1-8. 10.1007/s40820-020-00549-4 PMC8187673 34138248

[319] Liu C, Zhang YS, Chen F, Wu XY, Zhang BB, Wu ZD, et al. Immunopathology in schistosomiasis is regulated by TLR2,4- A nd IFN-γ-activated MSC through modulating Th1/Th2 responses. Stem Cell Res Ther. 2020;11:1-0. 10.1186/s13287-020-01735-2 PMC7275460 32503644

[320] Wang RF, Wang HY (2002). Enhancement of antitumor immunity by prolonging antigen presentation on dendritic cells. Nat Biotechnol.

[321] Wang RF (2002). Enhancing antitumor immune responses: Intracellular peptide delivery and identification of MHC class II-restricted tumor antigens. Immunol Rev.

[322] Huang X, Kong N, Zhang X, Cao Y, Langer R, Tao W. The landscape of mRNA nanomedicine. Nat Med 2022;28:2273–87. 10.1038/s41591-022-02061-1 36357682

[323] Hoernes TP, Heimdörfer D, Köstner D, Faserl K, Nußbaumer F, Plangger R, et al. Eukaryotic translation elongation is modulated by single natural nucleotide derivatives in the coding sequences of mRNAs. Genes (Basel). 2019;10(2):84. 10.3390/genes10020084 PMC6409545 30691071

[324] 837. Improving the Specificity of RNAi-Based Therapeutics for Huntington’s Disease. Mol Ther. 2011;19:S320.

[325] Dewi KS, Kusharyoto W (2020). Strategy for Designing the Synthetic Gene Encoding Human papillomavirus Major Capsid L1 Protein for Heterologous Expression in Escherichia coli System. Biog J Ilm Biol.

[326] Tudek A, Krawczyk PS, Mroczek S, Tomecki R, Turtola M, Matylla-Kulińska K, et al. Global view on the metabolism of RNA poly(A) tails in yeast Saccharomyces cerevisiae. Nat Commun. 2021;12(1):4951. 10.1038/s41467-021-25251-w PMC8367983 34400637

[327] Zhou S, Hill C, Sarkar S, Tse V, Sheahan T, Baric R (2021). RNHC inhibits SARS-CoV-2 in vitro but is mutagenic in mammalian cells. Top Antivir Med.

[328] Baiersdörfer M, Boros G, Muramatsu H, Mahiny A, Vlatkovic I, Sahin U (2019). A Facile Method for the Removal of dsRNA Contaminant from In Vitro-Transcribed mRNA. Mol Ther - Nucleic Acids.

[329] Dilley KA, Voorhies AA, Luthra P, Puri V, Stockwell TB, Lorenzi H, et al. The Ebola virus VP35 protein binds viral immunostimulatory and host RNAs identified through deep sequencing. PLoS One. 2017;12. 10.1371/journal.pone.0178717 PMC5479518 28636653

[330] Javaheri B, Stern A, Lara N, Dallas M, Zhao H, Liu YY-BY (2005). Rubio D: Spontaneous human adult stem cell transformation. Cancer Res..

[331] Gehring NH, Roignant JY. Anything but Ordinary – Emerging Splicing Mechanisms in Eukaryotic Gene Regulation. Trends Genet. 2021;37(4):355-72. 10.1016/j.tig.2020.10.008 33203572

[332] Kanagasabai R, Serdar L, Karmahapatra S, Kientz CA, Ellis J, Ritke MK (2017). Alternative RNA processing of topoisomerase IIα in etoposide-resistant human leukemia K562 cells: Intron retention results in a novel C-terminal truncated 90-kDa isoform. J Pharmacol Exp Ther.

[333] Wei L, Zhang Q, Zhong C, Aubé J, Welch DR, Wu X (2022). Abstract P4–01–16: Overcome chemoresistance of triple-negative breast cancer by inhibiting the RNA-binding protein HuR. Cancer Res..

[334] Su D, Chan CTY, Gu C, Lim KS, Chionh YH, McBee ME (2014). Quantitative analysis of ribonucleoside modifications in tRNA by HPLC-coupled mass spectrometry. Nat Protoc.

[335] Giri P, Kumar G (2009). Molecular Aspects of Small Molecules-Poly(A) Interaction: An Approach to RNA Based Drug Design. Curr Med Chem.

[336] Moudgil A, Wilkinson MN, Chen X, He J, Cammack AJ, Vasek MJ (2020). Self-Reporting Transposons Enable Simultaneous Readout of Gene Expression and Transcription Factor Binding in Single Cells. Cell.

[337] Anderluzzi G, Lou G, Woods S, Schmidt ST, Gallorini S, Brazzoli M (2022). The role of nanoparticle format and route of administration on self-amplifying mRNA vaccine potency. J Control Release.

[338] Tsiambas E, Chrysovergis A, Papanikolaou V, Mastronikolis N, Ragos V, Batistatou A, et al. Impact of Ribosome Activity on SARS-CoV-2 LNP – Based mRNA Vaccines. Front Mol Biosci. 2021;8:654866. 10.3389/fmolb.2021.654866 PMC8093617 33959636

[339] Beaudoin JD, Perreault JP (2010). 5′-UTR G-quadruplex structures acting as translational repressors. Nucleic Acids Res.

[340] Jungers CF, Djuranovic S. Modulation of miRISC-Mediated Gene Silencing in Eukaryotes. Front Mol Biosci. 2022;9:832916. 10.3389/fmolb.2022.832916 PMC8882679 35237661

[341] Tomé I, Francisco V, Fernandes H, Ferreira L. High-throughput screening of nanoparticles in drug delivery. APL Bioeng. 2021;5(3):031511. 10.1063/5.0057204 PMC8397474 34476328

[342] Byszewska M, Śmietański M, Purta E, Bujnicki JM. RNA methyltransferases involved in 5′ cap biosynthesis. RNA Biol. 2014;11(12):1597-607. 10.1080/15476286.2015.1004955 PMC4615557 25626080

[343] Lauridsen LH, Doessing HB, Long KS, Nielsen AT. A capture-SELEX strategy for multiplexed selection of RNA aptamers against small molecules. Methods Mol Biol. 2018;291–306. 10.1007/978-1-4939-7295-1_18 29170966

[344] Schott JW, Morgan M, Galla M, Schambach A. Viral and synthetic RNA vector technologies and applications. Mol Ther. 2016;24(9):1513-27. 10.1038/mt.2016.143 PMC5113109 27377044

[345] Su S, Wang Q, Jiang S. Facing the challenge of viral mutations in the age of pandemic: Developing highly potent, broad‐spectrum, and safe COVID‐19 vaccines and therapeutics. Clin Transl Med. 2021;11(1). 10.1002/ctm2.284 PMC7803365 33463059

[346] Zouridis H, Hatzimanikatis V, Zhu K, Chan W, Heymach J, Wilkinson M (2015). Regulation of G(1) arrest and apoptosis in hypoxia by PERK and GCN2-mediated eIF2alpha phosphorylation. J Biol Chem.

[347] Goto Y, Suga H (2021). The RaPID Platform for the Discovery of Pseudo-Natural Macrocyclic Peptides. Acc Chem Res.

[348] Filonov GS, Piatkevich KD, Ting L-M, Zhang J, Kim K, Verkhusha VV (2011). Green fluorescent protein specified small interfering RNA-cross-linked iron oxide nanoparticles-Cy5.5. Nat Biotechnol..

[349] Ratajczak-Wielgomas K, Grzegrzolka J, Piotrowska A, Gomulkiewicz A, Witkiewicz W, Dziegiel P (2016). Periostin expression in cancer-associated fibroblasts of invasive ductal breast carcinoma. Oncol Rep.

[350] Deliu LP, Ghosh A, Grewal SS (2017). Investigation of protein synthesis in Drosophila larvae using puromycin labelling. Biol Open.

[351] Mohammed MEA (2021). SARS-CoV-2 Proteins: Are They Useful as Targets for COVID-19 Drugs and Vaccines?. Curr Mol Med.

[352] Qi Y, Zhang Y, Mu Q, Zheng G, Zhang M, Chen B, et al. RNA Secondary Structurome Revealed Distinct Thermoregulation in Plasmodium falciparum. Front Cell Dev Biol. 2022;9:3496. 10.3389/fcell.2021.766532 PMC8763798 35059397

[353] Mehta D, Chirmade T, Tungekar AA, Gani K, Bhambure R. Cloning and expression of antibody fragment (Fab) I: Effect of expression construct and induction strategies on light and heavy chain gene expression. Biochem Eng J. 2021;176:108189.

[354] Yan L, Yang Y, Li M, Zhang Y, Zheng L, Ge J (2021). Coupling of N7-methyltransferase and 3′-5′ exoribonuclease with SARS-CoV-2 polymerase reveals mechanisms for capping and proofreading. Cell.

[355] Sohi AN, Kiani J, Arefian E, Khosrojerdi A, Fekrirad Z, Ghaemi S, et al. Development of an mrna-lnp vaccine against sars-cov-2: Evaluation of immune response in mouse and rhesus macaque. Vaccines. 2021;9(9):1007. 10.3390/vaccines9091007 PMC8472963 34579244

[356] Zagoskin M V., Wang J, Neff AT, Veronezi GMB, Davis RE. Small RNA pathways in the nematode Ascaris in the absence of piRNAs. Nat Commun. 2022;13(1):837. 10.1038/s41467-022-28482-7 PMC8837657 35149688

[357] Metz PJ, Ching KA, Xie T, Delgado Cuenca P, Niessen S, Tatlock JH (2020). Symmetric Arginine Dimethylation Is Selectively Required for mRNA Splicing and the Initiation of Type I and Type III Interferon Signaling. Cell Rep.

[358] Burgaz S, García C, Gómez-Cañas M, Rolland A, Muñoz E, Fernández-Ruiz J. Neuroprotection with the cannabidiol quinone derivative VCE-004.8 (EHP-101) against 6-hydroxydopamine in cell and murine models of Parkinson’s disease. Molecules. 2021;26(11):3245. 10.3390/molecules26113245 PMC8198479 34071302

[359] Srivastava A, Shukla P. Tightening the Screws on PsbA in Cyanobacteria. Trends Genet. 2021;37(3):211-5. 10.1016/j.tig.2020.08.018 32977998

[360] Hillen HS, Bartuli J, Grimm C, Dienemann C, Bedenk K, Szalay AA (2019). Structural Basis of Poxvirus Transcription: Transcribing and Capping Vaccinia Complexes. Cell.

[361] Suskiewicz MJ, Palazzo L, Hughes R, Ahel I (2021). Progress and outlook in studying the substrate specificities of PARPs and related enzymes. FEBS J.

[362] Moradian H, Roch T, Anthofer L, Lendlein A, Gossen M (2022). Chemical modification of uridine modulates mRNA-mediated proinflammatory and antiviral response in primary human macrophages. Mol Ther - Nucleic Acids.

[363] Zhou Y, Pu Q, Chen J, Hao G, Gao R, Ali A, et al. Thiol-based functional mimicry of phosphorylation of the two-component system response regulator ArcA promotes pathogenesis in enteric pathogens. Cell Rep. 2021;37(12):110147. 10.1016/j.celrep.2021.110147 PMC8728512 34936880

[364] Warminski M, Kowalska J, Nowak E, Kubacka D, Tibble R, Kasprzyk R (2021). Structural Insights into the Interaction of Clinically Relevant Phosphorothioate mRNA Cap Analogs with Translation Initiation Factor 4E Reveal Stabilization via Electrostatic Thio-Effect. ACS Chem Biol.

[365] Nogueira-De-Almeida CA, Ued FDV, Del Ciampo LA, Martinez EZ, Ferraz IS, Contini AA, et al. Prevalence of childhood anaemia in Brazil: still a serious health problem: a systematic review and meta-analysis. Public Health Nutr. 2021;24(18):6450-65. 10.1017/S136898002100286X PMC11148596 34212834

[366] Hu H, Flynn N, Zhang H, You C, Hang R, Wang X, et al. SPAAC-NAD-seq, a sensitive and accurate method to profile NAD+-capped transcripts. Proc Natl Acad Sci U S A. 2021;118(13):e2025595118. 10.1073/pnas.2025595118 PMC8020637 33753511

[367] Kis Z, Kontoravdi C, Shattock R, Shah N (2021). Correction: Resources, production scales and time required for producing RNA vaccines for the global pandemic demand. Vaccines..

[368] Nair P, Sapre SU. Significance of RNA sensors in activating immune system in emerging viral diseases. Dyn Immune Act Viral Dis. 2020. 229–42.

[369] Jiang Y, Chang M, Lan Y, Huang S (2019). Mechanism of CAP1-mediated apical actin polymerization in pollen tubes. Proc Natl Acad Sci U S A.

[370] Blanchard EL, Loomis KH, Bhosle SM, Vanover D, Baumhof P, Pitard B (2019). Proximity Ligation Assays for In Situ Detection of Innate Immune Activation: Focus on In Vitro-Transcribed mRNA. Mol Ther - Nucleic Acids.

[371] Poonia T, Silva WGDP, van Wijngaarden J. Derivation of an accurate geometry of 2-fluoroaniline from rotational spectroscopy and computational chemistry. J Mol Struct. 2021;1225.

[372] Mangkalaphiban K, He F, Ganesan R, Wu C, Baker R, Jacobson A. Transcriptome-wide investigation of stop codon readthrough in Saccharomyces cerevisiae. PLoS Genet. 2021;17(4):e1009538. 10.1371/journal.pgen.1009538 PMC8087045 33878104

[373] Karollus A, Avsec Ž, Gagneur J. Predicting mean ribosome load for 5’UTR of any length using deep learning. PLoS Comput Biol. 2021;17(5):e1008982. 10.1371/journal.pcbi.1008982 PMC8136849 33970899

[374] Vasquez G, Migawa MT, Wan WB, Low A, Tanowitz M, Swayze EE (2022). Evaluation of Phosphorus and Non-Phosphorus Neutral Oligonucleotide Backbones for Enhancing Therapeutic Index of Gapmer Antisense Oligonucleotides. Nucleic Acid Ther.

[375] Cui Z, Shen X, Zhang X, Li F, Amevor FK, Zhu Q (2021). A functional polymorphism of inhibin alpha subunit at miR-181b-1-3p-binding site regulates proliferation and apoptosis of chicken ovarian granular cells. Cell Tissue Res.

[376] Kim SC, Sekhon SS, Shin WR, Ahn G, Cho BK, Ahn JY, et al. Modifications of mRNA vaccine structural elements for improving mRNA stability and translation efficiency. Mol Cell. Toxicol. 2022;1-18. 10.1007/s13273-021-00171-4 PMC8450916 34567201

[377] Wishka DG, Lopez OD, Rudchenko VF, Huang G, Bahde R, Kumar V (2020). The development of β-selective glycosylation reactions with benzyl substituted 2-deoxy-1,4-dithio-D-erythro-pentofuranosides: enabling practical multi-gram syntheses of 4’-Thio-2’-deoxycytidine (T-dCyd) and 5-aza-4’-thio-2’-deoxycytidine (aza-T-dCyd) to s. Nucleosides, Nucleotides Nucleic Acids.

[378] Liu C, Chen F, Zhang J, Liu L, Lei H, Li H (2019). Metabolic Changes of Fusarium graminearum Induced by TPS Gene Deletion. J Proteome Res.

[379] Berry N, Suspène R, Caval V, Khalfi P, Beauclair G, Rigaud S, et al. Herpes Simplex Virus Type 1 Infection Disturbs the Mitochondrial Network, Leading to Type I Interferon Production through the RNA Polymerase III/RIG-I Pathway. MBio. 2021;12(6):e02557-21. 10.1128/mBio.02557-21 PMC8609356 34809467

[380] Passarelli MC, Pinzaru AM, Asgharian H, Liberti MV, Heissel S, Molina H (2022). Leucyl-tRNA synthetase is a tumour suppressor in breast cancer and regulates codon-dependent translation dynamics. Nat Cell Biol.

[381] Staudacher J, Rebnegger C, Dohnal T, Landes N, Mattanovich D, Gasser B (2022). Going beyond the limit: Increasing global translation activity leads to increased productivity of recombinant secreted proteins in Pichia pastoris. Metab Eng.

[382] Seymour BJ, Singh S, Certo HM, Sommer K, Sather BD, Khim S (2021). Effective, safe, and sustained correction of murine XLA using a UCOE-BTK promoter-based lentiviral vector. Mol Ther - Methods Clin Dev.

[383] Hernandez SM, Tikhonova EB, Baca KR, Zhao F, Zhu X, Karamyshev AL. Unexpected implication of srp and ago2 in parkinson’s disease: Involvement in alpha-synuclein biogenesis. Cells. 2021;10(10):2792. 10.3390/cells10102792 PMC8534902 34685771

[384] Nisar S, Bhat AA, Singh M, Karedath T, Rizwan A, Hashem S, et al. Insights Into the Role of CircRNAs: Biogenesis, Characterization, Functional, and Clinical Impact in Human Malignancies. Front Cell Dev Biol. 2021;9:617281. 10.3389/fcell.2021.617281 PMC7894079 33614648

[385] Shukla S, Bjerke GA, Muhlrad D, Yi R, Parker R (2019). The RNase PARN Controls the Levels of Specific miRNAs that Contribute to p53 Regulation. Mol Cell.

[386] Gu S, Jeon HM, Nam SW, Hong KY, Rahman MS, Lee JB (2022). The flip-flop configuration of the PABP-dimer leads to switching of the translation function. Nucleic Acids Res.

[387] Wu MZ, Asahara H, Tzertzinis G, Roy B (2020). Synthesis of low immunogenicity RNA with high-temperature in vitro transcription. RNA.

[388] Georgiadis C, Rasaiyaah J, Gkazi SA, Preece R, Etuk A, Christi A (2019). Universal’ Fratricide-Resistant CAR T Cells Against T Cell Leukemia Generated By Coupled & Uncoupled Deamination Mediated Base Editing. Blood.

[389] Arango D, Sturgill D, Alhusaini N, Dillman AA, Sweet TJ, Hanson G (2018). Acetylation of Cytidine in mRNA Promotes Translation Efficiency. Cell.

[390] Khavari B, Cairns MJ. Epigenomic Dysregulation in Schizophrenia: In Search of Disease Etiology and Biomarkers. Cells. 2020;9(8):1837. 10.3390/cells9081837 PMC7463953 32764320

[391] Zhu QC, Li S, Yuan LX, Chen RA, Liu DX, Fung TS. Induction of the proinflammatory chemokine interleukin-8 is regulated by integrated stress response and ap-1 family proteins activated during coronavirus infection. Int J Mol Sci. 2021;22(11):5646. 10.3390/ijms22115646 PMC8198748 34073283

[392] Papies J, Emanuel J, Heinemann N, Kulić Ž, Schroeder S, Tenner B (2021). Corrigendum: Antiviral and Immunomodulatory Effects of Pelargonium sidoides DC. Root Extract EPs® 7630 in SARS-CoV-2-Infected Human Lung Cells. Front Pharmacol.

[393] Takahashi-Nakaguchi A, Shishido E, Yahara M, Urayama SI, Ninomiya A, Chiba Y, et al. Phenotypic and Molecular Biological Analysis of Polymycovirus AfuPmV-1M From Aspergillus fumigatus: Reduced Fungal Virulence in a Mouse Infection Model. Front Microbiol. 2020;11:607795. 10.3389/fmicb.2020.607795 PMC7794001 33424809

[394] Lášek T, Petrová M, Košiová I, Šimák O, Buděšínský M, Kozák J, et al. 5′-Phosphonate modified oligoadenylates as potent activators of human RNase L. Bioorganic Med Chem. 2022;56:116632. 10.1016/j.bmc.2022.116632 35078032

[395] Taemaitree L, Shivalingam A, El-Sagheer AH, Brown T. “Split-and-Click” sgRNA. Methods Mol Biol. 2021;61–78. 10.1007/978-1-0716-0687-2_5 32926378

[396] Shemesh CS, Hsu JC, Hosseini I, Shen BQ, Rotte A, Twomey P, et al. Personalized Cancer Vaccines: Clinical Landscape, Challenges, and Opportunities. Mol Ther. 2021;29(2):555-70. 10.1016/j.ymthe.2020.09.038 PMC7854282 33038322

[397] Kremsner P, Mann P, Bosch J, Fendel R, Gabor JJ, Kreidenweiss A, et al. Phase 1 Assessment of the Safety and Immunogenicity of an mRNA- Lipid Nanoparticle Vaccine Candidate Against SARS-CoV-2 in Human Volunteers. medRxiv. 2020;2020.11.09.20228551.

[398] Hewitt SL, Bailey D, Zielinski J, Apte A, Musenge F, Karp R (2020). Intratumoral IL12 mRNA therapy promotes TH1 transformation of the tumor microenvironment. Clin Cancer Res.

[399] Taylor D, Korrer M (2021). 581 Multi-dimensional Synergy of Combinations (MuSYC) Algorithm Optimizes Combinatorial STING and TLR Adjuvant Cancer Vaccines. J Immunother Cancer.

[400] Nakamura T, Sato T, Endo R, Sasaki S, Takahashi N, Sato Y, et al. STING agonist loaded lipid nanoparticles overcome anti-PD-1 resistance in melanoma lung metastasis via NK cell activation. J Immunother Cancer. 2021;9(7). 10.1136/jitc-2021-002852 PMC8256839 34215690

[401] Pollack KE, Meneveau MO, Melssen MM, Lynch KT, Koeppel AF, Young SJ, et al. Incomplete freund’s adjuvant reduces arginase and enhances th1 dominance, tlr signaling and cd40 ligand expression in the vaccine site microenvironment. J Immunother Cancer. 2020;8(1). 10.1136/jitc-2020-000544 PMC7213888 32350119

[402] Carlino MS, Larkin J, Long GV. Immune checkpoint inhibitors in melanoma. Lancet. 2021;398(10304):1002-14. 10.1016/S0140-6736(21)01206-X 34509219

[403] Stravokefalou V, Stellas D, Karaliota S, Nagy B, Guerin T, Kozlov S (2021). Abstract 2727: Heterodimeric IL-15 (hetIL-15) affects conventional dendritic cells and a distinct novel dendritic cell population in different mouse cancer models of breast and pancreatic cancer. Cancer Res.

[404] Murrieta-Coxca JM, Gutiérrez-Samudio RN, El-Shorafa HM, Groten T, Rodríguez-Martínez S, Cancino-Diaz ME (2021). Role of IL-36 cytokines in the regulation of angiogenesis potential of trophoblast cells. Int J Mol Sci.

[405] Panelli MC, Wang E, Monsurro V, Marincola FM. The role of quantitative PCR for the immune monitoring of cancer patients. Expert Opin. Biol. Ther. 2002;2(5):557-64. 10.1517/14712598.2.5.557 12079490

[406] Yu H, Bruneau RC, Brennan G, Rothenburg S. Battle royale: Innate recognition of poxviruses and viral immune evasion. Biomedicines. 2021;9(7):765. 10.3390/biomedicines9070765 PMC8301327 34356829

[407] Yang Z, Mitländer H, Vuorinen T, Finotto S. Mechanism of Rhinovirus Immunity and Asthma. Front Immunol. 2021;12:731846. 10.3389/fimmu.2021.731846 PMC8526928 34691038

[408] Qiao Y, Zhu S, Deng S, Zou SS, Gao B, Zang G, et al. Human Cancer Cells Sense Cytosolic Nucleic Acids Through the RIG-I–MAVS Pathway and cGAS–STING Pathway. Front Cell Dev Biol. 2021;8:606001. 10.3389/fcell.2020.606001 PMC7820189 33490069

[409] Dela Justina V, Giachini FR, Priviero F, Webb RC. COVID-19 and hypertension: Is there a role for dsRNA and activation of Toll-like receptor 3? Vascul Pharmacol. 2021;140:106861. 10.1016/j.vph.2021.106861 PMC8061373 33845201

[410] Cesaro T, Hayashi Y, Borghese F, Vertommen D, Wavreil F, Michiels T. PKR activity modulation by phosphomimetic mutations of serine residues located three aminoacids upstream of double-stranded RNA binding motifs. Sci Rep. 2021;11(1):1-6. 10.1038/s41598-021-88610-z PMC8080564 33911136

[411] Qasem A, Naser AE, Naser SA. Enteropathogenic infections modulate intestinal serotonin transporter (SERT) function by activating Toll-like receptor 2 (TLR-2) in Crohn’s disease. Sci Rep. 2021;11(1):22624. 10.1038/s41598-021-02050-3 PMC8604993 34799637

[412] Appelberg S, John L, Pardi N, Végvári Á, Bereczky S, Ahlén G, et al. Nucleoside-Modified mRNA Vaccines Protect IFNAR –/– Mice against Crimean-Congo Hemorrhagic Fever Virus Infection . J Virol. 2022;96(3):e01568-21. 10.1128/jvi.01568-21 PMC8826901 34817199

[413] Buharideen SM, Hassan MSH, Najimudeen SM, Niu D, Czub M, Gomis S, et al. Immune responses in laying hens after an infectious bronchitis vaccination of pullets: A comparison of two vaccination strategies. Vaccines. 2021;9(5):531. 10.3390/vaccines9050531 PMC8161194 34065415

[414] Ngono AE, Syed T, Nguyen AV, Regla-Nava JA, Susantono M, Spasova D, et al. CD8+ T cells mediate protection against Zika virus induced by an NS3-based vaccine. Sci Adv. 2020;6(45):eabb2154. 10.1126/sciadv.abb2154 PMC7673678 33148638

[415] Wu H, Li L, Chen KM, Homolka D, Gos P, Fleury-Olela F (2019). Decapping Enzyme NUDT12 Partners with BLMH for Cytoplasmic Surveillance of NAD-Capped RNAs. Cell Rep.

[416] Maruggi G, Mallett CP, Westerbeck JW, Chen T, Lofano G, Friedrich K (2022). A self-amplifying mRNA SARS-CoV-2 vaccine candidate induces safe and robust protective immunity in preclinical models. Mol Ther.

[417] Yeh JX, Schultz KLW, Calvert V, Petricoin EF, Griffin DE (2020). The NF-κB/leukemia inhibitory factor/STAT3 signaling pathway in antibody-mediated suppression of Sindbis virus replication in neurons. Proc Natl Acad Sci U S A.

[418] Johanning FW, Conry RM, Lobuglio AF, Wright M, Sumerel LA, Pike MJ (1995). A sindbis virus mRNA polynucleotide vector achieves prolonged and high level heterologous gene expression in vivo. Nucleic Acids Res.

[419] Deb S, Tammi K, Gao XZ, Kalita K, Mahanta P, Cross S (2022). A Robust Two-Stage Planning Model for the Charging Station Placement Problem Considering Road Traffic Uncertainty. IEEE Trans Intell Transp Syst.

[420] Dalmann A, Reimann I, Wernike K, Beer M. Autonomously Replicating RNAs of Bungowannah Pestivirus: E RNS Is Not Essential for the Generation of Infectious Particles. J Virol. 2020;94(14):e00436-20. 10.1128/JVI.00436-20 PMC7343217 32404522

[421] Yang Z, Guo L, Yang Z (2019). Emergency logistics for wildfire suppression based on forecasted disaster evolution. Ann Oper Res.

[422] Malicoat J, Manivasagam S, Zuñiga S, Sola I, McCabe D, Rong L, et al. Development of a Single-Cycle Infectious SARS-CoV-2 Virus Replicon Particle System for Use in Biosafety Level 2 Laboratories. J Virol. 2022;96(3):e01837-21. 10.1128/jvi.01837-21 PMC8826801 34851142

[423] Goswami R, O'hagan DT, Adamo R, Baudner BC (2021). Conjugation of mannans to enhance the potency of liposome nanoparticles for the delivery of rna vaccines. Pharmaceutics..

[424] Blakney AK, McKay PF, Hu K, Samnuan K, Jain N, Brown A (2021). Polymeric and lipid nanoparticles for delivery of self-amplifying RNA vaccines. J Control Release.

[425] Geall AJ, Verma A, Otten GR, Shaw CA, Hekele A, Banerjee K (2012). Nonviral delivery of self-amplifying RNA vaccines. Proc Natl Acad Sci U S A.

[426] Zhang NN, Li XF, Deng YQ, Zhao H, Huang YJ, Yang G (2020). A Thermostable mRNA Vaccine against COVID-19. Cell.

[427] Antunes KH, Fachi JL, de Paula R, da Silva EF, Pral LP, dos Santos AÁ, et al. Microbiota-derived acetate protects against respiratory syncytial virus infection through a GPR43-type 1 interferon response. Nat Commun. 2019;10(1):3273. 10.1038/s41467-019-11152-6 PMC6646332 31332169

[428] Stokes A, Pion J, Binazon O, Laffont B, Bigras M, Dubois G, et al. Nonclinical safety assessment of repeated administration and biodistribution of a novel rabies self-amplifying mRNA vaccine in rats: Toxicity and biodistribution of rabies SAM vaccine. Regul Toxicol Pharmacol. 2020;113:104648. 10.1016/j.yrtph.2020.104648 32240713

[429] Hunt C, Hartford SA, White D, Pefanis E, Hanna T, Herman C, et al. Tissue-specific activation of gene expression by the Synergistic Activation Mediator (SAM) CRISPRa system in mice. Nat Commun. 2021;12(1):2770. 10.1038/s41467-021-22932-4 PMC8119962 33986266

[430] Kim YS, Lim J, Sung J, Cheong Y, Lee EY, Kim J (2020). Built-in RNA-mediated chaperone (chaperna) for antigen folding tailored to immunized hosts. Biotechnol Bioeng.

[431] Tong DC, Bloom JE, Quinn S, Nasis A, Hiew C, Roberts-Thomson P (2021). Colchicine in Patients With Acute Coronary Syndrome: Two-Year Follow-Up of the Australian COPS Randomized Clinical Trial. Circulation.

[432] Inbaraj S, Sejian V, Ramasamy S. Role of environmental stressor-host immune system–pathogen interactions in development of infectious disease in farm animals. Biol. Rhythm Res. 2022;53(5):707-24.

[433] Dewitte H, Van Lint S, Heirman C, Thielemans K, De Smedt SC, Breckpot K (2014). The potential of antigen and TriMix sonoporation using mRNA-loaded microbubbles for ultrasound-triggered cancer immunotherapy. J Control Release.

[434] Terhorst D, Fossum E, Baranska A, Tamoutounour S, Malosse C, Garbani M (2015). Laser-Assisted Intradermal Delivery of Adjuvant-Free Vaccines Targeting XCR1+ Dendritic Cells Induces Potent Antitumoral Responses. J Immunol.

[435] Kim YC, Prausnitz MR. Enabling skin vaccination using new delivery technologies. Drug Deliv Transl Res. 2011;1:7-12. 10.1007/s13346-010-0005-z PMC3143039 21799951

[436] Teixeira L, Medioni J, Garibal J, Adotevi O, Doucet L, Durey MAD (2020). A first-in-human phase I study of INVAC-1, an optimized human telomerase DNA vaccine in patients with advanced solid tumors. Clin Cancer Res.

[437] Lin CC, Yen MC, Lin CM, Huang SS, Yang HJ, Chow NH (2008). Delivery of noncarrier naked DNA vaccine into the skin by supersonic flow induces a polarized T helper type 1 immune response to cancer. J Gene Med.

[438] Isaguliants M, Ljungberg K, editors. Advances in DNA Vaccines. Adv DNA Vacc. 2021.

[439] Corbett HJ, Fernando GJP, Chen X, Frazer IH, Kendall MAF. Skin vaccination against cervical cancer associated human papillomavirus with a novel micro-projection array in a mouse model. PLoS One. 2010;5(10):e13460. 10.1371/journal.pone.0013460 PMC2956639 20976136

[440] Li T, Wang Y, Chen J, Gao X, Pan S, Su Y (2020). Co-delivery of brinzolamide and miRNA-124 by biodegradable nanoparticles as a strategy for glaucoma therapy. Drug Deliv.

[441] Wan X, Sun R, Bao Y, Zhang C, Wu Y, Gong Y (2021). In Vivo Delivery of siRNAs Targeting EGFR and BRD4 Expression by Peptide-Modified Redox Responsive PEG-PEI Nanoparticles for the Treatment of Triple-Negative Breast Cancer. Mol Pharm.

[442] Karpenko LI, Rudometov AP, Sharabrin SV, Shcherbakov DN, Borgoyakova MB, Bazhan SI (2021). Delivery of mrna vaccine against sars-cov-2 using a polyglucin: Spermidine conjugate. Vaccines.

[443] Piperno A, Sciortino MT, Giusto E, Montesi M, Panseri S, Scala A. Recent advances and challenges in gene delivery mediated by polyester-based nanoparticles. Int J Nanomedicine. 2021;16:5981. 10.2147/IJN.S321329 PMC8418317 34511901

[444] Bai S, Sun Y, Cheng Y, Ye W, Jiang C, Liu M, et al. MCP mediated active targeting calcium phosphate hybrid nanoparticles for the treatment of orthotopic drug-resistant colon cancer. J Nanobiotechnology. 2021;19(1):1-20. 10.1186/s12951-021-01115-9 PMC8600743 34789268

[445] Liszewski MK, Atkinson JP. Membrane cofactor protein (MCP; CD46): deficiency states and pathogen connections. Curr Opin Immunol. 2021;72:126-34. 10.1016/j.coi.2021.04.005 PMC8123722 34004375

[446] Šošić L, Selbo PK, Kotkowska ZK, Kündig TM, Høgset A, Johansen P. Photochemical internalization: Light paves way for new cancer chemotherapies and vaccines. Cancers (Basel). 2020;12(1):165. 10.3390/cancers12010165 PMC7016662 31936595

[447] Sun X, Ni Y, He Y, Yang M, Tani T, Kitajima S, et al. Engineering the Immune Adaptor Protein STING as a Functional Carrier. Adv Ther. 2021;4(8):2100066.

[448] Tsai SJ, Amerman A, Jewell CM. Altering Antigen Charge to Control Self-Assembly and Processing of Immune Signals During Cancer Vaccination. Front Immunol. 2021;11:3340. 10.3389/fimmu.2020.613830 PMC7815530 33488621

[449] Rahman S, Kumar V, Kumar A, Abdullah TS, Rather IA, Jan AT. Molecular perspective of nanoparticle mediated therapeutic targeting in breast cancer: An odyssey of endoplasmic reticulum unfolded protein response (UPRER) and beyond. Biomedicines. 2021;9(6):635. 10.3390/biomedicines9060635 PMC8229605 34199484

[450] Akhtar B, Muhammad F, Aslam B, Saleemi MK, Sharif A (2020). Pharmacokinetic profile of chitosan modified poly lactic co-glycolic acid biodegradable nanoparticles following oral delivery of gentamicin in rabbits. Int J Biol Macromol.

[451] Yokoo H, Oba M, Uchida S. Cell-Penetrating Peptides: Emerging Tools for mRNA Delivery. Pharmaceutics. 2022;14(1):78. 10.3390/pharmaceutics14010078 PMC8781296 35056974

[452] Brimmo A, Menachery A, Qasaimeh M (2019). 3D printed micro-electro-fluidic probe (MeFP) for single cell electroporation.

[453] Bosch NC, Martin LM, Voskens CJ, Berking C, Seliger B, Schuler G, et al. A chimeric il-15/il-15rα molecule expressed on nfκb-activated dendritic cells supports their capability to activate natural killer cells†. Int J Mol Sci. 2021;22(19):10227. 10.3390/ijms221910227 PMC8508776 34638566

[454] Chariou PL, Beiss V, Ma Y, Steinmetz NF (2021). In situ vaccine application of inactivated CPMV nanoparticles for cancer immunotherapy. Mater Adv.

[455] Abudula T, Bhatt K, Eggermont LJ, O’Hare N, Memic A, Bencherif SA. Supramolecular Self-Assembled Peptide-Based Vaccines: Current State and Future Perspectives. Front Chem. 2020;8:598160. 10.3389/fchem.2020.598160 PMC7662149 33195107

[456] Waryono T (2019). RINGKASAN Puding merah (Gruptophyllum pictum L Griff). J Farm Galen (Galenika J Pharmacy).

[457] Singh V, Kesharwani P (2021). Recent advances in microneedles-based drug delivery device in the diagnosis and treatment of cancer. J Control Release.

[458] Andersson HA, Kim YS, O’Neill BE, Shi ZZ, Serda RE (2014). HSP70 promoter-driven activation of gene expression for immunotherapy using gold nanorods and near infrared light. Vaccines.

[459] Patel SG, Sayers EJ, He L, Narayan R, Williams TL, Mills EM, et al. Cell-penetrating peptide sequence and modification dependent uptake and subcellular distribution of green florescent protein in different cell lines. Sci Rep. 2019;9(1):6298. 10.1038/s41598-019-42456-8 PMC6472342 31000738

[460] Bell M, Rooks CP, Agrahari V. Drug Delivery Approaches and Imaging Techniques for Brain Tumor. Neuromethods. 2021. 109–26.

[461] Prajapati R, Garcia-Garrido E, Somoza Á. Albumin-based nanoparticles for the delivery of doxorubicin in breast cancer. Cancers (Basel). 2021;13(12):3011 10.3390/cancers13123011 PMC8235501 34208533

[462] Sohrabi B, Dayeri B, Zahedi E, Khoshbakht S, Nezamabadi Pour N, Ranjbar H, et al. Mesenchymal stem cell (MSC)-derived exosomes as novel vehicles for delivery of miRNAs in cancer therapy. Cancer Gene Ther. 2022;29(8-9):1105-16. 10.1038/s41417-022-00427-8 35082400

[463] Webster DM, Sundaram P, Byrne ME. Injectable nanomaterials for drug delivery: Carriers, targeting moieties, and therapeutics. Eur. J. Pharm. Biopharm. 2013;84(1):1-20. 10.1016/j.ejpb.2012.12.009 23313176

[464] Dhungel K, Narayan J. Nanoparticle: Significance as smart material in therapeutic strategies in drug delivery in biological systems. Appl Biomed Eng Neurosci. 2019. 327–39.

[465] Bariwal J, Ma H, Altenberg GA, Liang H. Nanodiscs: a versatile nanocarrier platform for cancer diagnosis and treatment. Chem Soc Rev. 2022. 1702–28. 10.1039/d1cs01074c 35156110

[466] Fisher RK, West PC, Mattern‐schain SI, Best MD, Kirkpatrick SS, Dieter RA, et al. Advances in the formulation and assembly of non‐cationic lipid nanoparticles for the medical application of gene therapeutics. Nanomaterials. 2021;11(3):825. 10.3390/nano11030825 PMC8004789 33807086

[467] Niazvand F, Fathinezhad Z, Alfuraiji N, Etajuri EA, Amini-Chermahini F, Chehelgerdi M, Ranjbar R. Clustered regularly interspaced short palindromic repeats system of genome engineering in embryos to repair genes. Journal of Biomedical Nanotechnology. 2021;17(3):331-56. 10.1166/jbn.2021.3045 33875070

[468] Nagasaka M (2021). ES28.04 Emerging Mechanisms to Target KRAS Directly. J Thorac Oncol..

[469] Brenneman RJ. Potentiating anti-tumor immunity using aptamer-siRNA conjugates. ProQuest Diss Theses. 2013.

[470] Simpson I (2021). Industry news update covering June 2021. Ther Deliv.

[471] Coolen AL, Lacroix C, Mercier-Gouy P, Delaune E, Monge C, Exposito JY (2019). Poly(lactic acid) nanoparticles and cell-penetrating peptide potentiate mRNA-based vaccine expression in dendritic cells triggering their activation. Biomaterials.

[472] Hang C, Xu S, Wu Q, Zhang P, Zhang Y, Xu Y. mRNA-based therapies and their clinical prospects. Kexue Tongbao/Chinese Sci Bull. 2021. 3649–66.

[473] Yang S, Wang D, Zhang X, Sun Y, Zheng B (2021). cRGD peptide-conjugated polyethylenimine-based lipid nanoparticle for intracellular delivery of siRNA in hepatocarcinoma therapy. Drug Deliv.

[474] Heller R, Heller LC (2015). Gene Electrotransfer Clinical Trials. Adv Genet.

[475] Shariatinia Z. Big family of nano- and microscale drug delivery systems ranging from inorganic materials to polymeric and stimuli-responsive carriers as well as drug-conjugates. J. Drug Deliv. Sci. Technol. 2021;66:102790.

[476] Paunovska K, Loughrey D, Dahlman JE, Coulter WH. Drug delivery systems for RNA therapeutics NATuRe ReVIewS | Genetics. Nat Rev Gennetic. 2022;23(5):265-80. 10.1038/s41576-021-00439-4 PMC8724758 34983972

[477] Patel S, Ryals RC, Weller KK, Pennesi ME, Sahay G (2019). Lipid nanoparticles for delivery of messenger RNA to the back of the eye. J Control Release.

[478] Paramasivam P, Franke C, Stöter M, Höijer A, Bartesaghi S, Sabirsh A, et al. Endosomal escape of delivered mRNA from endosomal recycling tubules visualized at the nanoscale. J Cell Biol. 2022;221(2):e202110137. 10.1083/jcb.202110137 PMC8666849 34882187

[479] Obuobi S, Ngoc Phung A, Julin K, Johannessen M, Škalko-Basnet N (2022). Biofilm Responsive Zwitterionic Antimicrobial Nanoparticles to Treat Cutaneous Infection. Biomacromol.

[480] Park S, Choi YK, Kim S, Lee J, Im W (2021). CHARMM-GUI Membrane Builder for Lipid Nanoparticles with Ionizable Cationic Lipids and PEGylated Lipids. J Chem Inf Model.

[481] Cui L, Hunter MR, Sonzini S, Pereira S, Romanelli SM, Liu K, et al. Mechanistic Studies of an Automated Lipid Nanoparticle Reveal Critical Pharmaceutical Properties Associated with Enhanced mRNA Functional Delivery In Vitro and In Vivo. Small. 2022;18(9):2105832. 10.1002/smll.202105832 34914866

[482] Schlich M, Palomba R, Costabile G, Mizrahy S, Pannuzzo M, Peer D, et al. Cytosolic delivery of nucleic acids: The case of ionizable lipid nanoparticles. Bioeng Transl Med. 2021;6(2):e10213. 10.1002/btm2.10213 PMC7995196 33786376

[483] Nakajima T, Nagano K, Fukuda Y, Ishima Y, Shibata H, Isaka R (2022). Subvisible Particles Derived by Dropping Stress Enhance Anti-PEG Antibody Production and Clearance of PEGylated Proteins in Mice. J Pharm Sci.

[484] Mehanny M, Lehr CM, Fuhrmann G. Extracellular vesicles as antigen carriers for novel vaccination avenues. Adv Drug Deliv Rev. 2021;173:164-80. 10.1016/j.addr.2021.03.016 33775707

[485] Van Der Jeught K, De Koker S, Bialkowski L, Heirman C, Tjok Joe P, Perche F (2018). Dendritic Cell Targeting mRNA Lipopolyplexes Combine Strong Antitumor T-Cell Immunity with Improved Inflammatory Safety. ACS Nano.

[486] Zhou X, Pan Y, Yu L, Wu J, Li Z, Li H (2021). Feasibility of cRGD conjugation at 5′-antisense strand of siRNA by phosphodiester linkage extension. Mol Ther - Nucleic Acids.

[487] Garrido C, Curtis AD, Dennis M, Pathak SH, Gao H, Montefiori D, et al. SARS-CoV-2 vaccines elicit durable immune responses in infant rhesus macaques. Sci Immunol. 2021;6(60):eabj3684. 10.1126/sciimmunol.abj3684 PMC8774290 34131024

[488] Sabnis RW (2021). Novel Substituted Pyrazines as Splicing Modulators for Modulating Splicing of mRNA and Uses Thereof for Treating Diseases. ACS Med Chem Lett.

[489] Zia SR. Role of water in the determination of protonation states of titratable residues. J Mol Model. 2021;27(2):61. 10.1007/s00894-021-04677-5 33517493

[490] Perini DA, Alcaraz A, Queralt-Martín M. Lipid headgroup charge and acyl chain composition modulate closure of bacterial β-barrel channels. Int J Mol Sci. 2019;20(3):674. 10.3390/ijms20030674 PMC6386941 30764475

[491] Cornebise M, Narayanan E, Xia Y, Acosta E, Ci L, Koch H, et al. Discovery of a Novel Amino Lipid That Improves Lipid Nanoparticle Performance through Specific Interactions with mRNA. Adv Funct Mater. 2022;32(8):2106727.

[492] Uddin MN, Roni MA. Challenges of storage and stability of mrna-based covid-19 vaccines. Vaccines. 2021;9(9):1033. 10.3390/vaccines9091033 PMC8473088 34579270

[493] Schoenmaker L, Witzigmann D, Kulkarni JA, Verbeke R, Kersten G, Jiskoot W, et al. mRNA-lipid nanoparticle COVID-19 vaccines: Structure and stability. Int J Pharm. 2021;601:120586. 10.1016/j.ijpharm.2021.120586 PMC8032477 33839230

[494] Selig M, Lauer JC, Hart ML, Rolauffs B. Mechanotransduction and stiffness-sensing: Mechanisms and opportunities to control multiple molecular aspects of cell phenotype as a design cornerstone of cell-instructive biomaterials for articular cartilage repair. Int J Mol Sci. 2020;21(15):5399. 10.3390/ijms21155399 PMC7432012 32751354

[495] Liu S, Cheng Q, Wei T, Yu X, Johnson LT, Farbiak L (2021). Membrane-destabilizing ionizable phospholipids for organ-selective mRNA delivery and CRISPR–Cas gene editing. Nat Mater.

[496] Moreno-Ulloa A, Sicairos Diaz V, Tejeda-Mora JA, Macias Contreras MI, Castillo FD, Guerrero A, et al. Chemical Profiling Provides Insights into the Metabolic Machinery of Hydrocarbon-Degrading Deep-Sea Microbes. mSystems. 2020;5(6):e00824-20. 10.1128/mSystems.00824-20 PMC7657597 33172970

[497] Megeressa M, Siraj B, Zarina S, Ahmed A. Structural characterization and in vitro lipid binding studies of non-specific lipid transfer protein 1 (nsLTP1) from fennel (Foeniculum vulgare) seeds. Sci Rep. 2020;10(1):1-3. 10.1038/s41598-020-77278-6 PMC7718255 33277525

[498] Che S, Liang Y, Chen Y, Wu W, Liu R, Zhang Q (2022). Structure of Pseudomonas aeruginosa spermidine dehydrogenase: a polyamine oxidase with a novel heme-binding fold. FEBS J.

[499] Mortazavi M, Shokrgozar MA, Sardari S, Azadmanesh K, Mahdian R, Kaghazian H (2019). Using chemical chaperones to increase recombinant human erythropoietin secretion in CHO cell line. Prep Biochem Biotechnol.

[500] Zhao S, Li J, Zhou Y, Huang L, Li Y, Xu J, et al. Lipid nanoparticles-encapsulated YF4: A potential therapeutic oral peptide delivery system for hypertension treatment. Front Pharmacol. 2019;10:102. 10.3389/fphar.2019.00102 PMC6401629 30873021

[501] Muñoz-Úbeda M, Semenzato M, Franco-Romero A, Junquera E, Aicart E, Scorrano L, et al. Transgene expression in mice of the Opa1 mitochondrial transmembrane protein through bicontinuous cubic lipoplexes containing gemini imidazolium surfactants. J Nanobiotechnology. 2021;19(1):425. 10.1186/s12951-021-01167-x PMC8684174 34922554

[502] El-Shabrawi MHF, Sherief LM, Yakoot M, Kamal NM, Almalky MA, AbdElgawad MM (2019). Effects of dual sofosbuvir/daclatasvir therapy on, chronic hepatitis C infected, survivors of childhood malignancy. World J Clin Cases.

[503] Shoma Suresh K, Bhat S, Guru BR, Muttigi MS, Seetharam RN. A nanocomposite hydrogel delivery system for mesenchymal stromal cell secretome. Stem Cell Res Ther. 2020;11(1):1-4. 10.1186/s13287-020-01712-9 PMC7251860 32460846

[504] Li MY, Liu LZ, Dong M. Progress on pivotal role and application of exosome in lung cancer carcinogenesis, diagnosis, therapy and prognosis. Mol Cancer. 2021;20:1-22. 10.1186/s12943-021-01312-y PMC7839206 33504342

[505] Nakamura S, Sayama M, Uwamizu A, Jung S, Ikubo M, Otani Y (2020). Non-naturally Occurring Regio Isomer of Lysophosphatidylserine Exhibits Potent Agonistic Activity toward G Protein-Coupled Receptors. J Med Chem.

[506] Park M, Kim Y, Lee H. Design of 2D massless Dirac fermion systems and quantum spin Hall insulators based on sp–sp2 carbon sheets. NPJ Comput Mater. 2018;4(1):54.

[507] Aouey B, Fares E, Chtourou Y, Bouchard M, Fetoui H. Lambda-cyhalothrin exposure alters purine nucleotide hydrolysis and nucleotidase gene expression pattern in platelets and liver of rats. Chem Biol Interact. 2019;311:108796. 10.1016/j.cbi.2019.108796 31421116

[508] Bost JP, Barriga H, Holme MN, Gallud A, Maugeri M, Gupta D, Erratum: Delivery of Oligonucleotide Therapeutics: Chemical Modifications, Lipid Nanoparticles, and Extracellular Vesicles (ACS Nano,  (2021). 15:9 (13993–14021) DOI: 10.1021/acsnano.1c05099). ACS Nano.

[509] Krhač Levačić A, Berger S, Müller J, Wegner A, Lächelt U, Dohmen C (2021). Dynamic mRNA polyplexes benefit from bioreducible cleavage sites for in vitro and in vivo transfer. J Control Release.

[510] Handayani A. Pengaruh Corporate Governance, Leverage, Dan Manajemen Laba Terhadap Agresivitas Pajak (Pada Perusahaan Propertydan Real Estate Yang Terdaftar Di Bursa Efek Indonesia Tahun 2016-2018). Molecules. 2019;9:148–62.

[511] Liu X, Zhao Z, Wu F, Chen Y, Yin L. Tailoring Hyperbranched Poly(β-amino ester) as a Robust and Universal Platform for Cytosolic Protein Delivery. Adv Mater. 2022;34(8):2108116. 10.1002/adma.202108116 34894367

[512] Li J, Liu S, Shi J, Zhu HJ. Activation of tenofovir alafenamide and sofosbuvir in the human lung and its implications in the development of nucleoside/nucleotide prodrugs for treating sars-cov-2 pulmonary infection. Pharmaceutics. 2021;13(10):1656. 10.3390/pharmaceutics13101656 PMC8540046 34683949

[513] Han Y-C, Lin C-M, Chen TT. RNA-Seq analysis of differentially expressed genes relevant to innate and adaptive immunity in cecropin P1 transgenic rainbow trout (Oncorhynchus mykiss). BMC Genomics. 2018;19:1-2. 10.1186/s12864-018-5141-8 PMC6195682 30340506

[514] Swingle KL, Billingsley MM, Bose SK, White B, Palanki R, Dave A (2022). Amniotic fluid stabilized lipid nanoparticles for in utero intra-amniotic mRNA delivery. J Control Release.

[515] Hattori Y, Nakamura M, Takeuchi N, Tamaki K, Shimizu S, Yoshiike Y (2019). Effect of cationic lipid in cationic liposomes on siRNA delivery into the lung by intravenous injection of cationic lipoplex. J Drug Target.

[516] Chen D, Ganesh S, Wang W, Amiji M (2019). The role of surface chemistry in serum protein corona-mediated cellular delivery and gene silencing with lipid nanoparticles. Nanoscale.

[517] Carrasco MJ, Alishetty S, Alameh MG, Said H, Wright L, Paige M, et al. Ionization and structural properties of mRNA lipid nanoparticles influence expression in intramuscular and intravascular administration. Commun Biol. 2021;4(1):956. 10.1038/s42003-021-02441-2 PMC8358000 34381159

[518] Surma MA, Gerl MJ, Herzog R, Helppi J, Simons K, Klose C. Mouse lipidomics reveals inherent flexibility of a mammalian lipidome. Sci Rep. 2021;11(1):19364. 10.1038/s41598-021-98702-5 PMC8481471 34588529

[519] Firdessa-Fite R, Creusot RJ (2020). Nanoparticles versus Dendritic Cells as Vehicles to Deliver mRNA Encoding Multiple Epitopes for Immunotherapy. Mol Ther - Methods Clin Dev.

[520] Wang Y, Zhang L, Xu Z, Miao L, Huang L (2018). mRNA Vaccine with Antigen-Specific Checkpoint Blockade Induces an Enhanced Immune Response against Established Melanoma. Mol Ther.

[521] Paloncýová M, Čechová P, Šrejber M, Kührová P, Otyepka M (2021). Role of Ionizable Lipids in SARS-CoV-2 Vaccines As Revealed by Molecular Dynamics Simulations: From Membrane Structure to Interaction with mRNA Fragments. J Phys Chem Lett.

[522] Miao L, Li L, Huang Y, Delcassian D, Chahal J, Han J (2019). Delivery of mRNA vaccines with heterocyclic lipids increases anti-tumor efficacy by STING-mediated immune cell activation. Nat Biotechnol.

[523] Kinsey C, Lu T, Deiss A, Vuolo K, Klein L, Rustandi RR (2022). Determination of lipid content and stability in lipid nanoparticles using ultra high-performance liquid chromatography in combination with a Corona Charged Aerosol Detector. Electrophoresis.

[524] Yen C, Abbasi AZ, He C, Amini MA, Lip H, Rauth M (2021). Abstract PO-100: Theragnostic tumor-targeted manganese dioxide-loaded polymer-lipid nanoparticles for magnetic resonance image-guided radiation therapy. Clin Cancer Res..

[525] Sharma A, Kontodimas K, Bosmann M. Nanomedicine: A Diagnostic and Therapeutic Approach to COVID-19. Front. Med. 2021;8:648005. 10.3389/fmed.2021.648005 PMC8211875 34150793

[526] Drakes D, Abbas A, Shields J, DeMuth P (2021). 157 Lymph node targeted boosting with cognate amphiphile-peptide vaccines enhances TCR-T Cell therapy to eradicate solid tumors. J Immunother Cancer.

[527] Zamani P, Momtazi-Borojeni AA, Nik ME, Oskuee RK, Sahebkar A. Nanoliposomes as the adjuvant delivery systems in cancer immunotherapy. J. Cell. Physiol. 2018;233(7):5189-99. 10.1002/jcp.26361 29215747

[528] Yeow YL, Wu J, Wang X, Winteringham L, Feindel KW, Tirnitz-Parker JEE, et al. ECM Depletion Is Required to Improve the Intratumoral Uptake of Iron Oxide Nanoparticles in Poorly Perfused Hepatocellular Carcinoma. Front Oncol. 2022;12:335. 10.3389/fonc.2022.837234 PMC8902243 35273916

[529] Hagino Y, Khalil IA, Kimura S, Kusumoto K, Harashima H (2021). GALA-Modified Lipid Nanoparticles for the Targeted Delivery of Plasmid DNA to the Lungs. Mol Pharm.

[530] Gao Y, Men K, Pan C, Li J, Wu J, Chen X (2021). Functionalized dmp-039 hybrid nanoparticle as a novel mrna vector for efficient cancer suicide gene therapy. Int J Nanomedicine.

[531] Živojević K, Mladenović M, Djisalov M, Mundzic M, Ruiz-Hernandez E, Gadjanski I, et al. Advanced mesoporous silica nanocarriers in cancer theranostics and gene editing applications. J Control Release. 2021;337:193-211. 10.1016/j.jconrel.2021.07.029 34293320

[532] Yang R, Deng Y, Huang B, Huang L, Lin A, Li Y, et al. A core-shell structured COVID-19 mRNA vaccine with favorable biodistribution pattern and promising immunity. Signal Transduct Target Ther. 2021;6(1):213. 10.1038/s41392-021-00634-z PMC8165147 34059617

[533] Brown DW, Raturi A, Bhandari P, Sosnowski D, Grin L, Wee P (2020). Safe and Effective Delivery of Nucleic Acids Using LNPs Formulated with Fusion-Associated Small Transmembrane Proteins. Mol Ther.

[534] Zhang R, El-Mayta R, Murdoch TJ, Warzecha CC, Billingsley MM, Shepherd SJ (2021). Helper lipid structure influences protein adsorption and delivery of lipid nanoparticles to spleen and liver. Biomater Sci.

[535] Evers MJW, Du W, Yang Q, Kooijmans SAA, Vink A, van Steenbergen M (2022). Delivery of modified mRNA to damaged myocardium by systemic administration of lipid nanoparticles. J Control Release.

[536] Lin PJC, Tam YK. Controlling protein expression by delivery of RNA therapeutics using lipid nanoparticles. Nucleic Acid Nanotheranostics Biomed Appl. 2019. 277–310.

[537] Sardo C, Bassi B, Craparo EF, Scialabba C, Cabrini E, Dacarro G (2017). Gold nanostar–polymer hybrids for siRNA delivery: Polymer design towards colloidal stability and in vitro studies on breast cancer cells. Int J Pharm.

[538] Blakney AK, McKay PF, Yus BI, Aldon Y, Shattock RJ (2019). Inside out: optimization of lipid nanoparticle formulations for exterior complexation and in vivo delivery of saRNA. Gene Ther.

[539] Suzuki Y, Ishihara H. Difference in the lipid nanoparticle technology employed in three approved siRNA (Patisiran) and mRNA (COVID-19 vaccine) drugs. Drug Metab. Pharmacokinet. 2021;41:100424. 10.1016/j.dmpk.2021.100424 PMC8502116 34757287

[540] Lutz J, Lazzaro S, Habbeddine M, Schmidt KE, Baumhof P, Mui BL, et al. Unmodified mRNA in LNPs constitutes a competitive technology for prophylactic vaccines. npj Vaccines. 2017;2(1):29. 10.1038/s41541-017-0032-6 PMC5648897 29263884

[541] Mok DZL, Chan KR. The effects of pre-existing antibodies on live-attenuated viral vaccines. Viruses. 2020;12(5):520. 10.3390/v12050520 PMC7290594 32397218

[542] Chiu TW, Peng CJ, Chen MC, Hsu MH, Liang YH, Chiu CH, et al. Constructing conjugate vaccine against Salmonella Typhimurium using lipid-A free lipopolysaccharide. J Biomed Sci. 2020;27(1):1-4. 10.1186/s12929-020-00681-8 PMC7443816 32831077

[543] Pylypchuk I V., Suo H, Chucheepchuenkamol C, Jedicke N, Lindén PA, Lindström ME, et al. High-Molecular-Weight Fractions of Spruce and Eucalyptus Lignin as a Perspective Nanoparticle-Based Platform for a Therapy Delivery in Liver Cancer. Front Bioeng Biotechnol. 2022;9:1467. 10.3389/fbioe.2021.817768 PMC8860172 35198551

[544] Xu R, Zhao S, Nie L, Deng C, Hao S, Zhao X (2021). Study on the technology of monodisperse droplets by a high-throughput and instant-mixing droplet microfluidic system. Materials (Basel).

[545] Cullis PR, Hope MJ. Lipid Nanoparticle Systems for Enabling Gene Therapies. Mol Ther. 2017;25(7):1467-75. 10.1016/j.ymthe.2017.03.013 PMC5498813 28412170

[546] Reátegui E, Van Der Vos KE, Lai CP, Zeinali M, Atai NA, Aldikacti B, et al. Engineered nanointerfaces for microfluidic isolation and molecular profiling of tumor-specific extracellular vesicles. Nat Commun. 2018;9(1):175. 10.1038/s41467-017-02261-1 PMC5766611 29330365

[547] Webb C, Forbes N, Roces CB, Anderluzzi G, Lou G, Abraham S, et al. Using microfluidics for scalable manufacturing of nanomedicines from bench to GMP: A case study using protein-loaded liposomes. Int J Pharm. 2020;582:119266. 10.1016/j.ijpharm.2020.119266 32251694

[548] Newswire PR (2020). CanSino Biologics and Precision NanoSystems Announce Collaboration to Co-Develop a COVID-19 RNA Vaccine. PNI-CanSino-COVID19.

[549] Meo SA, Bukhari IA, Akram J, Meo AS, Klonoff DC (2021). COVID-19 vaccines: Comparison of biological, pharmacological characteristics and adverse effects of pfizer/BioNTech and moderna vaccines. Eur Rev Med Pharmacol Sci.

[550] Munro APS, Janani L, Cornelius V, Aley PK, Babbage G, Baxter D (2021). Safety and immunogenicity of seven COVID-19 vaccines as a third dose (booster) following two doses of ChAdOx1 nCov-19 or BNT162b2 in the UK (COV-BOOST): a blinded, multicentre, randomised, controlled, phase 2 trial. Lancet.

[551] Hou X, Zaks T, Langer R, Dong Y. Lipid nanoparticles for mRNA delivery. Nat Rev Mater. 2021;6(12):1078-94. 10.1038/s41578-021-00358-0 PMC8353930 34394960

[552] Zhang D, Atochina-Vasserman EN, Lu J, Maurya DS, Xiao Q, Liu M (2022). The Unexpected Importance of the Primary Structure of the Hydrophobic Part of One-Component Ionizable Amphiphilic Janus Dendrimers in Targeted mRNA Delivery Activity. J Am Chem Soc.

[553] Shi D, Beasock D, Fessler A, Szebeni J, Ljubimova JY, Afonin KA, et al. To PEGylate or not to PEGylate: Immunological properties of nanomedicine’s most popular component, polyethylene glycol and its alternatives. Adv. Drug Deliv Rev. 2022;180:114079. 10.1016/j.addr.2021.114079 PMC8899923 34902516

[554] Mohanty A, Uthaman S, Park IK. Utilization of polymer-lipid hybrid nanoparticles for targeted anti-cancer therapy. Molecules. 2020;25(19):4377. 10.3390/molecules25194377 PMC7582728 32977707

[555] Riley RS, June CH, Langer R, Mitchell MJ. Delivery technologies for cancer immunotherapy. Nat Rev Drug Discov. 2019;18(3):175-96. 10.1038/s41573-018-0006-z PMC6410566 30622344

[556] Ren J, Cao Y, Li L, Wang X, Lu H, Yang J (2021). Self-assembled polymeric micelle as a novel mRNA delivery carrier. J Control Release.

[557] Chiper M, Tounsi N, Kole R, Kichler A, Zuber G (2017). Self-aggregating 1.8 kDa polyethylenimines with dissolution switch at endosomal acidic pH are delivery carriers for plasmid DNA, mRNA, siRNA and exon-skipping oligonucleotides. J Control Release..

[558] Sturm L, Schwemberger B, Menzel U, Häckel S, Albers CE, Plank C, et al. In vitro evaluation of a nanoparticle-based mrna delivery system for cells in the joint. Biomedicines. 2021;9(7):794. 10.3390/biomedicines9070794 PMC8301349 34356857

[559] Sezlev Bilecen D, Uludag H, Hasirci V (2019). Development of PEI-RANK siRNA Complex Loaded PLGA Nanocapsules for the Treatment of Osteoporosis. Tissue Eng - Part A.

[560] Pacheco-Torres J, Penet MF, Krishnamachary B, Mironchik Y, Chen Z, Bhujwalla ZM. PD-L1 siRNA Theranostics With a Dextran Nanoparticle Highlights the Importance of Nanoparticle Delivery for Effective Tumor PD-L1 Downregulation. Front Oncol. 2021;10:614365. 10.3389/fonc.2020.614365 PMC7947807 33718115

[561] Hamada E, Kurosaki T, Hashizume J, Harasawa H, Nakagawa H, Nakamura T (2021). Anionic complex with efficient expression and good safety profile for mRNA delivery. Pharmaceutics.

[562] Paris JL, Coelho F, Teixeira A, Diéguez L, Silva BFB, Abalde-Cela S. In vitro evaluation of lipopolyplexes for gene transfection: Comparing 2D, 3D and microdroplet-enabled cell culture. Molecules. 2020;25(14):3277. 10.3390/molecules25143277 PMC7397275 32708478

[563] Xu X, Duan J, Lan Q, Kuang Y, Liao T, Liu Y, et al. A dual-sensitive poly(amino acid)/hollow mesoporous silica nanoparticle-based anticancer drug delivery system with a rapid charge-reversal property. J Drug Deliv Sci Technol. 2021;66:102817.

[564] Zheng X, Pang X, Yang P, Wan X, Wei Y, Guo Q (2017). A hybrid siRNA delivery complex for enhanced brain penetration and precise amyloid plaque targeting in Alzheimer’s disease mice. Acta Biomater.

[565] Timin AS, Muslimov AR, Lepik KV, Epifanovskaya OS, Shakirova AI, Mock U (2018). Efficient gene editing via non-viral delivery of CRISPR–Cas9 system using polymeric and hybrid microcarriers. Nanomedicine Nanotechnology, Biol Med.

[566] Fenton OS, Kauffman KJ, Kaczmarek JC, McClellan RL, Jhunjhunwala S, Tibbitt MW, et al. Synthesis and Biological Evaluation of Ionizable Lipid Materials for the In Vivo Delivery of Messenger RNA to B Lymphocytes. Adv Mater. 2017;29(33):1606944. 10.1002/adma.201606944 28681930

[567] Zohra FT, Chowdhury EH, Tada S, Hoshiba T, Akaike T (2007). Effective delivery with enhanced translational activity synergistically accelerates mRNA-based transfection. Biochem Biophys Res Commun.

[568] Ebeid K, Geary SM, Salem AK. Preparation and Characterization of a Liver Targeted, Poly(amidoamine) Based, Gene Delivery System. Methods Mol Biol. 2022. 319–32. 10.1007/978-1-0716-2128-8_24 PMC9670859 35213004

[569] Bellis AD, Peňalver-Bernabé B, Weiss MS, Yarrington ME, Barbolina MV, Pannier AK (2011). Cellular arrays for large-scale analysis of transcription factor activity. Biotechnol Bioeng.

[570] Shah V, Taratula O, Garbuzenko OB, Taratula OR, Rodriguez-Rodriguez L, Minko T (2013). Targeted nanomedicine for suppression of CD44 and simultaneous cell death induction in ovarian cancer: An optimal delivery of siRNA and anticancer drug. Clin Cancer Res.

[571] Golan M, Feinshtein V, David A (2016). Conjugates of HA2 with octaarginine-grafted HPMA copolymer offer effective siRNA delivery and gene silencing in cancer cells. Eur J Pharm Biopharm.

[572] Arami S, Mahdavi M, Rashidi MR, Fathi M, Hejazi MS, Samadi N. Multifunctional Superparamagnetic Nanoparticles: From Synthesis to siRNA Delivery. Curr Pharm Des. 2016;;23(16):2400-9. 10.2174/1381612822666161031153159 27799034

[573] Yao S, Huang P, Liu F, Zeng F, Zeng W, He S (2020). Preparation of a polymer-modified folate-targeted magnetic nano-delivery system and its inhibitory effect on nasopharyngeal carcinoma. Mater Express.

[574] Xiong XB, Uludaĝ H, Lavasanifar A (2010). Virus-mimetic polymeric micelles for targeted siRNA delivery. Biomaterials.

[575] Tack F, Noppe M, Van Dijck A, Dekeyzer N, Van Der Leede BJ, Bakker A (2008). Delivery of a DNAzyme targeting c-myc to HT29 colon carcinoma cells using a gold nanoparticulate approach. Pharmazie.

[576] Maksimowski NA, Song X, Bae EH, Reich H, John R, Pei Y, et al. Follistatin-like-1 (Fstl1) is a fibroblast-derived growth factor that contributes to progression of chronic kidney disease. Int J Mol Sci. 2021;22(17):9513. 10.3390/ijms22179513 PMC8431028 34502419

[577] Isakova-Sivak I, Stepanova E, Mezhenskaya D, Matyushenko V, Prokopenko P, Sychev I, et al. Influenza vaccine: progress in a vaccine that elicits a broad immune response. Expert Rev. Vaccines. 2021;20(9):1097-112. 10.1080/14760584.2021.1964961 34348561

[578] Wu XR, Zhang J, Zhang JH, Xiao YP, He X, Liu YH, et al. Amino acid-linked low molecular weight polyethylenimine for improved gene delivery and biocompatibility. Molecules. 2020;25(4):975. 10.3390/molecules25040975 PMC7070781 32098282

[579] Lan B, Wu J, Li N, Pan C, Yan L, Yang C (2020). Hyperbranched cationic polysaccharide derivatives for efficient siRNA delivery and diabetic wound healing enhancement. Int J Biol Macromol.

[580] Galiano M, Miah S, Akinbami O, Gonzalez Gonoggia S, Ellis J, Zambon M. A29 Genetic heterogeneity of influenza A (H3N2) viruses in the United Kingdom, 2016–8. Virus Evol. 2019;5(Supplement_1):vez002-028.

[581] Iyoda T, Shimoyama S, Liu K, Omatsu Y, Akiyama Y, Maeda Y (2002). The CD8+ dendritic cell subset selectively endocytoses dying cells in culture and in vivo. J Exp Med.

[582] Son S, Nam J, Zenkov I, Ochyl LJ, Xu Y, Scheetz L (2020). Sugar-Nanocapsules Imprinted with Microbial Molecular Patterns for mRNA Vaccination. Nano Lett.

[583] Białkowska K, Miłowska K, Michlewska S, Sokołowska P, Komorowski P, Lozano-Cruz T, et al. Interaction of cationic carbosilane dendrimers and their siRNA complexes with MCF-7 cells. Int J Mol Sci. 2021;22(13):7097. 10.3390/ijms22137097 PMC8269323 34281151

[584] Khan MK, Nigavekar SS, Minc LD, Kariapper MST, Nair BM, Lesniak WG, et al. In vivo biodistribution of dendrimers and dendrimer nanocomposites - Implications for cancer imaging and therapy. Technol. Cancer Res. Treat. 2005;4(6):603-13. 10.1177/153303460500400604 16292880

[585] Pastor-Ibáñez R, Díez-Fuertes F, Sánchez-Palomino S, Alcamí J, Plana M, Torrents D, et al. Impact of transcriptome and gut microbiome on the response of hiv-1 infected individuals to a dendritic cell-based hiv therapeutic vaccine. Vaccines. 2021;9(7):694. 10.3390/vaccines9070694 PMC8310021 34202658

[586] Thomas TJ, Tajmir-Riahi HA, Pillai CKS. Biodegradable polymers for gene delivery. Molecules. 2019;24(20):3744. 10.3390/molecules24203744 PMC6832905 31627389

[587] Wu H, Zhou J, Zhu T, Cohen I, Dictenberg J (2020). A kinesin adapter directly mediates dendritic mRNA localization during neural development in mice. J Biol Chem.

[588] Zhang H, Leal J, Soto MR, Smyth HDC, Ghosh D (2020). Aerosolizable lipid nanoparticles for pulmonary delivery of mRNA through design of experiments. Pharmaceutics.

[589] Kowalski PS, Capasso Palmiero U, Huang Y, Rudra A, Langer R, Anderson DG. Ionizable amino-polyesters synthesized via ring opening polymerization of tertiary amino-alcohols for tissue selective mRNA delivery. Adv Mater. 2018;30(34):1801151. 10.1002/adma.201801151 PMC6320729 29975801

[590] Haabeth OAW, Blake TR, McKinlay CJ, Tveita AA, Sallets A, Waymouth RM (2019). Local delivery of OX40L, CD80, and CD86 mRNA kindles global anticancer immunity. Cancer Res.

[591] McKinlay CJ, Benner NL, Haabeth OA, Waymouth RM, Wender PA (2018). Enhanced mRNA delivery into lymphocytes enabled by lipid-varied libraries of charge-altering releasable transporters. Proc Natl Acad Sci U S A.

[592] Nasr SS, Lee S, Thiyagarajan D, Boese A, Loretz B, Lehr CM. Co-delivery of mRNA and pDNA using thermally stabilized coacervate-based core-shell nanosystems. Pharmaceutics. 2021;13;13(11):1924. 10.3390/pharmaceutics13111924 PMC8619316 34834339

[593] Amjad S, Mushtaq S, Rehman R, Munir A, Zahid N, Siddique PQR. Protamine 1/Protamine 2 mRNA ratio in nonobstructive azoospermic patients. Andrologia. 2021;53(3):e13936. 10.1111/and.13936 33427330

[594] Tong J, Wang X, Liu Y, Ren X, Wang A, Chen Z, et al. Pooled CRISPR screening identifies m6A as a positive regulator of macrophage activation. Sci Adv. 2021;7(18):eabd4742. 10.1126/sciadv.abd4742 PMC8081357 33910903

[595] Bahiri Elitzur S, Cohen-Kupiec R, Yacobi D, Fine L, Apt B, Diament A (2021). Prokaryotic rRNA-mRNA interactions are involved in all translation steps and shape bacterial transcripts. RNA Biol.

[596] Armbruster N, Jasny E, Petsch B. Advances in rna vaccines for preventive indications: A case study of a vaccine against rabies. Vaccines. 2019;7(4):132. 10.3390/vaccines7040132 PMC6963972 31569785

[597] Zhang H, Men K, Pan C, Gao Y, Li J, Lei S, et al. Treatment of colon cancer by degradable rrppc nano-conjugates delivered stat3 sirna. Int J Nanomedicine. 2020;9875-90. 10.2147/IJN.S277845 PMC7732178 33324056

[598] Udhayakumar VK, De Beuckelaer A, McCaffrey J, McCrudden CM, Kirschman JL, Vanover D, et al. Arginine-Rich Peptide-Based mRNA Nanocomplexes Efficiently Instigate Cytotoxic T Cell Immunity Dependent on the Amphipathic Organization of the Peptide. Adv Healthc Mater. 2017;6(13):1601412. 10.1002/adhm.201601412 28436620

[599] Zhang R, Tang L, Tian Y, Ji X, Hu Q, Zhou B (2020). DP7-C-modified liposomes enhance immune responses and the antitumor effect of a neoantigen-based mRNA vaccine. J Control Release.

[600] Tateshita N, Miura N, Tanaka H, Masuda T, Ohtsuki S, Tange K (2019). Development of a lipoplex-type mRNA carrier composed of an ionizable lipid with a vitamin E scaffold and the KALA peptide for use as an ex vivo dendritic cell-based cancer vaccine. J Control Release.

[601] Bellefroid C, Reusch C, Lechanteur A, Evrard B, Debacq-Chainiaux F, Mottet D, et al. Systematic study of liposomes composition towards efficient delivery of plasmid DNA as potential application of dermal fibroblasts targeting. Int J Pharm. 2021;593:120122. 10.1016/j.ijpharm.2020.120122 33307161

[602] Samsa MM, Dupuy LC, Beard CW, Six CM, Schmaljohn CS, Mason PW (2019). Self-Amplifying RNA Vaccines for Venezuelan Equine Encephalitis Virus Induce Robust Protective Immunogenicity in Mice. Mol Ther.

[603] Luisi K, Morabito KM, Burgomaster KE, Sharma M, Kong WP, Foreman BM, et al. Development of a potent Zika virus vaccine using self-amplifying messenger RNA. Sci Adv. 2020;6(32):eaba5068. 10.1126/sciadv.aba5068 PMC7413734 32821824

[604] Wang X, Chen Z, Huang Y, Ye X, Wang J, Yang Y (2020). Liquid crystallinity and thermal properties of polyhedral oligomeric silsesquioxane/side-chain azobenzene hybrid copolymer. Nanotechnol Rev.

[605] Dudzik P, Trojan SE, Ostrowska B, Lasota M, Dulinska-Litewka J, Laidler P (2019). Aberrant promoter methylation may be responsible for the control of CD146 (MCAM) gene expression during breast cancer progression. Acta Biochim Pol.

[606] Persano F, Leporatti S (2020). Current Overview of Inorganic Nanoparticles for the Treatment of Central Nervous System (CNS) Diseases. Curr Nanomater.

[607] Wang P, Perche F, Midoux P, Cabral Cátia SD, Malard V, Correia IJ (2021). In Vivo bone tissue induction by freeze-dried collagen-nanohydroxyapatite matrix loaded with BMP2/NS1 mRNAs lipopolyplexes. J Control Release..

[608] Meng C, Chen Z, Mai J, Shi Q, Tian S, Hinkle L, et al. Virus-Mimic mRNA Vaccine for Cancer Treatment. Adv Ther. 2021;4(11):2100144. 10.1002/adtp.202100144 PMC8646380 34901386

[609] Revon-Riviere G, Ninove L, Min V, Rome A, Coze C, Verschuur A (2021). The BNT162b2 mRNA COVID-19 vaccine in adolescents and young adults with cancer: A monocentric experience. Eur J Cancer.

[610] Van Hoecke L, Verbeke R, De Vlieger D, Dewitte H, Roose K, Van Nevel S (2020). mRNA Encoding a Bispecific Single Domain Antibody Construct Protects against Influenza A Virus Infection in Mice. Mol Ther - Nucleic Acids.

[611] De Beuckelaer A, Pollard C, Van Lint S, Roose K, Van Hoecke L, Naessens T (2016). Type I interferons interfere with the capacity of mRNA lipoplex vaccines to elicit Cytolytic T cell responses. Mol Ther.

[612] Rahamimov N, Baturov V, Shani A, Ben Zoor I, Fischer D, Chernihovsky A (2021). Inadequate deltoid muscle penetration and concerns of improper COVID mRNA vaccine administration can be avoided by injection technique modification. Vaccine.

[613] Ols S, Yang L, Thompson EA, Pushparaj P, Tran K, Liang F (2020). Route of Vaccine Administration Alters Antigen Trafficking but Not Innate or Adaptive Immunity. Cell Rep.

[614] Dalla Pietà A, Carpanese D, Grigoletto A, Tosi A, Dalla Santa S, Pedersen GK (2021). Hyaluronan is a natural and effective immunological adjuvant for protein-based vaccines. Cell Mol Immunol.

[615] Szőke D, Kovács G, Kemecsei É, Bálint L, Szoták-Ajtay K, Aradi P, et al. Nucleoside-modified VEGFC mRNA induces organ-specific lymphatic growth and reverses experimental lymphedema. Nat Commun. 2021;12(1):3460 10.1038/s41467-021-23546-6 PMC8187400 34103491

[616] León Y, Zapata L, Molina RE, Okanovič G, Gómez LA, Daza-Castro C (2020). Intranasal immunization of mice with multiepitope chimeric vaccine candidate based on conserved autotransporters SiGa, Pic and Sap, confers protection against Shigella Flexneri. Vaccines.

[617] Huang L, Shi Y, Gong B, Jiang L, Zhang Z, Liu X, et al. Dynamic blood single-cell immune responses in patients with COVID-19. Signal Transduct Target Ther. 2021;6(1):110. 10.1038/s41392-021-00526-2 PMC7936231 33677468

[618] Irvine EB, O’Neil A, Darrah PA, Shin S, Choudhary A, Li W (2021). Robust IgM responses following intravenous vaccination with Bacille Calmette-Guérin associate with prevention of Mycobacterium tuberculosis infection in macaques. Nat Immunol.

[619] Lee K, Kim TS, Seo Y, Kim SY, Lee H (2020). Combined hybrid structure of siRNA tailed IVT mRNA (ChriST mRNA) for enhancing DC maturation and subsequent anticancer T cell immunity. J Control Release.

[620] Vlatkovic I. Non-immunotherapy application of lnp-mrna: Maximizing efficacy and safety. Biomedicines. 2021;9(5):530. 10.3390/biomedicines9050530 PMC8151051 34068715

[621] Román JJM, Del Campo M, Villar J, Paolini F, Curzio G, Venuti A, et al. Immunotherapeutic potential of mollusk hemocyanins in combination with human vaccine adjuvants in murine models of oral cancer. J Immunol Res. 2019;2019. 10.1155/2019/7076942 PMC6362480 30847353

[622] Tusup M, Läuchli S, Jarzebska NT, French LE, Chang YT, Vonow-Eisenring M, et al. mRNA-based anti-TCR CDR3 tumour vaccine for T-cell lymphoma. Pharmaceutics. 2021;13(7):1040. 10.3390/pharmaceutics13071040 PMC8308944 34371731

[623] Gruell H, Vanshylla K, Tober-Lau P, Hillus D, Schommers P, Lehmann C (2022). mRNA booster immunization elicits potent neutralizing serum activity against the SARS-CoV-2 Omicron variant. Nat Med.

[624] Omar R, Yang J, Alrushaid S, Burczynski FJ, Minuk GY, Gong Y (2019). Inhibition of BMP4 and Alpha Smooth Muscle Actin Expression in LX-2 Hepatic Stellate Cells by BMP4-siRNA Lipid Based Nanoparticle. J Pharm Pharm Sci.

[625] Amatu A, Pani A, Patelli G, Gagliardi OM, Loparco M, Piscazzi D (2022). Impaired seroconversion after SARS-CoV-2 mRNA vaccines in patients with solid tumours receiving anticancer treatment. Eur J Cancer.

[626] Lin H, Wang K, Xiong Y, Zhou L, Yang Y, Chen S, et al. Identification of Tumor Antigens and Immune Subtypes of Glioblastoma for mRNA Vaccine Development. Front Immunol. 2022;13:249. 10.3389/fimmu.2022.773264 PMC8847306 35185876

[627] Chen X, Zhang Y, Fu Y. The critical role of Toll-like receptor-mediated signaling in cancer immunotherapy. Med Drug Discov. 2022;100122.

[628] Sahin I, George A, Seyhan AA. Therapeutic targeting of alternative rna splicing in gastrointestinal malignancies and other cancers. Int. J Mol Sci. 2021;22(21):11790. 10.3390/ijms222111790 PMC8583749 34769221

[629] Polack FP, Thomas SJ, Kitchin N, Absalon J, Gurtman A, Lockhart S (2020). Safety and Efficacy of the BNT162b2 mRNA Covid-19 Vaccine. N Engl J Med.

[630] Baden LR, El Sahly HM, Essink B, Kotloff K, Frey S, Novak R (2021). Efficacy and Safety of the mRNA-1273 SARS-CoV-2 Vaccine. N Engl J Med.

[631] Sahin U, Derhovanessian E, Miller M, Kloke BP, Simon P, Löwer M (2017). Personalized RNA mutanome vaccines mobilize poly-specific therapeutic immunity against cancer. Nature.

[632] Jackson LA, Anderson EJ, Rouphael NG, Roberts PC, Makhene M, Coler RN (2020). An mRNA Vaccine against SARS-CoV-2 — Preliminary Report. N Engl J Med.

[633] Pardi N, Hogan MJ, Pelc RS, Muramatsu H, Andersen H, DeMaso CR (2017). Zika virus protection by a single low-dose nucleoside-modified mRNA vaccination. Nature.

[634] Mulligan MJ, Lyke KE, Kitchin N, Absalon J, Gurtman A, Lockhart S (2020). Phase I/II study of COVID-19 RNA vaccine BNT162b1 in adults. Nature.

[635] Ott PA, Hu Z, Keskin DB, Shukla SA, Sun J, Bozym DJ (2017). An immunogenic personal neoantigen vaccine for patients with melanoma. Nature.

[636] Rittig SM, Haentschel M, Weimer KJ, Heine A, Muller MR, Brugger W (2011). Intradermal vaccinations with RNA coding for TAA generate CD8 and CD4 immune responses and induce clinical benefit in vaccinated patients. Mol Ther.

[637] Tanyi JL, Bobisse S, Ophir E, Tuyaerts S, Roberti A, Genolet R, et al. Personalized cancer vaccine effectively mobilizes antitumor T cell immunity in ovarian cancer. Sci Transl Med. 2018;10(436):eaao5931. 10.1126/scitranslmed.aao5931 29643231

[638] Sahin U, Oehm P, Derhovanessian E, Jabulowsky RA, Vormehr M, Gold M (2020). An RNA vaccine drives immunity in checkpoint-inhibitor-treated melanoma. Nature.

[639] Jimeno A, Gupta S, Sullivan R, Do KT, Akerley WL, Wang D (2020). Abstract CT032: A phase 1/2, open-label, multicenter, dose escalation and efficacy study of mRNA-2416, a lipid nanoparticle encapsulated mRNA encoding human OX40L, for intratumoral injection alone or in combination with durvalumab for patients with advanc. Cancer Res..

[640] NCT04486378. A Phase II Clinical Trial Comparing the Efficacy of RO7198457 Versus Watchful Waiting in Patients With ctDNA-positive, Resected Stage II (High Risk) and Stage III Colorectal Cancer. https://clinicaltrials.gov/show/NCT04486378. 2020.

[641] Anderson BR, Muramatsu H, Nallagatla SR, Bevilacqua PC, Sansing LH, Weissman D (2010). Incorporation of pseudouridine into mRNA enhances translation by diminishing PKR activation. Nucleic Acids Res.

[642] Kim SH, Kim Y, Jung JY, Park NY, Jang H, Hyun JW (2019). Erratum: High seroprevalence and index of anti-john-cunningham virus antibodies in korean patients with multiple sclerosis. Clin Neurol..

[643] Kranz LM, Diken M, Haas H, Kreiter S, Loquai C, Reuter KC (2016). Systemic RNA delivery to dendritic cells exploits antiviral defence for cancer immunotherapy. Nature.

[644] Ebert LM, MacRaild SE, Zanker D, Davis ID, Cebon J, Chen W. A Cancer Vaccine Induces Expansion of NY-ESO-1-Specific Regulatory T Cells in Patients with Advanced Melanoma. PLoS One. 2012;7(10):e48424. 10.1371/journal.pone.0048424 PMC3482213 23110239

[645] Adenoviral Tumor-specific Neoantigen Priming Vaccine GRT-C901. Definitions. 2020;14(17):4255.

[646] Van Nuffel AMT, Benteyn D, Wilgenhof S, Pierret L, Corthals J, Heirman C (2012). Dendritic cells loaded with mRNA encoding full-length tumor antigens prime CD4+ and CD8+ T cells in melanoma patients. Mol Ther.

[647] Papachristofilou A, Hipp MM, Klinkhardt U, Früh M, Sebastian M, Weiss C, et al. Phase Ib evaluation of a self-adjuvanted protamine formulated mRNA-based active cancer immunotherapy, BI1361849 (CV9202), combined with local radiation treatment in patients with stage IV non-small cell lung cancer. J Immunother Cancer. 2019;7:1-4. 10.1186/s40425-019-0520-5 PMC6368815 30736848

[648] Gay CL, Kuruc JD, Falcinelli SD, Warren JA, Reifeis SA, Kirchherr JL, et al. Assessing the impact of AGS-004, a dendritic cell-based immunotherapy, and vorinostat on persistent HIV-1 Infection. Sci Rep. 2020;10(1):1-3. 10.1038/s41598-020-61878-3 PMC7083965 32198428

[649] Wilgenhof S, Van Nuffel AMT, Benteyn D, Corthals J, Aerts C, Heirman C (2013). A phase IB study on intravenous synthetic mRNA electroporated dendritic cell immunotherapy in pretreated advanced melanoma patients. Ann Oncol.

[650] Sanmamed MF, Rodriguez I, Schalper KA, Oñate C, Azpilikueta A, Rodriguez-Ruiz ME (2015). Nivolumab and urelumab enhance antitumor activity of human T lymphocytes engrafted in Rag2-/-IL2Rγnull immunodeficient mice. Cancer Res.

[651] Amin A, Plimack ER, Infante JR, Ernstoff MS, Rini BI, McDermott DF (2014). Nivolumab (anti-PD-1; BMS-936558, ONO-4538) in combination with sunitinib or pazopanib in patients (pts) with metastatic renal cell carcinoma (mRCC). J Clin Oncol.

[652] Toso JF, Gill VJ, Hwu P, Marincola FM, Restifo NP, Schwartzentruber DJ (2002). Phase I study of the intravenous administration of attenuated Salmonella typhimurium to patients with metastatic melanoma. J Clin Oncol..

[653] Srivastava RM, Trivedi S, Concha-Benavente F, Gibson SP, Reeder C, Ferrone S (2017). CD137 Stimulation Enhances Cetuximab-Induced Natural Killer: Dendritic Cell Priming of Antitumor T-Cell Immunity in Patients with Head and Neck Cancer. Clin Cancer Res..

[654] Krombach J, Hennel R, Brix N, Orth M, Schoetz U, Ernst A, et al. Priming anti-tumor immunity by radiotherapy: Dying tumor cell-derived DAMPs trigger endothelial cell activation and recruitment of myeloid cells. Oncoimmunology. 2019;8(1):e1523097. 10.1080/2162402X.2018.1523097 PMC6287777 30546963

[655] Ishiura N, Yanaba K, Nashiro K, Kudo M, Goto T, Okochi H (2021). Inhibitory role of interleukin-10 in the cutaneous reverse Arthus reaction. J Dermatol.

[656] Philippidis A (2019). eTheRNA Advances mRNA-Based Melanoma Immunotherapy ECI-006. GEN Edge.

[657] Holcomb EA, Pearson AN, Jungles KM, Tate A, James J, Jiang L (2022). High-content CRISPR screening in tumor immunology. Front Immunol..

[658] De Keersmaecker B, Claerhout S, Carrasco J, Bar I, Corthals J, Wilgenhof S, et al. TriMix and tumor antigen mRNA electroporated dendritic cell vaccination plus ipilimumab: Link between T-cell activation and clinical responses in advanced melanoma. J Immunother Cancer. 2020;8(1). 10.1136/jitc-2019-000329 PMC7057443 32114500

[659] Salah A, Wang H, Li Y, Ji M, Ou W Bin, Qi N, et al. Insights Into Dendritic Cells in Cancer Immunotherapy: From Bench to Clinical Applications. Front Cell Dev Biol. 2021;9:686544. 10.3389/fcell.2021.686544 PMC8273339 34262904

[660] Jameson-Lee M, Ott PA, Luke JJ, Postow MA, Poklepovic AS (2021). Multicenter phase I/II trial of encorafenib with and without binimetinib in combination with nivolumab and low-dose ipilimumab in metastatic BRAF-mutant melanoma. J Clin Oncol..

[661] Patel M, Jimeno A, Wang D, Stemmer S, Bauer T, Sweis R (2021). 539 Phase 1 study of mRNA-2752, a lipid nanoparticle encapsulating mRNAs encoding human OX40L/IL-23/IL-36γ, for intratumoral (ITu) injection +/- durvalumab in advanced solid tumors and lymphoma. J Immunother Cancer.

[662] Byrne J, Baker K, Houston A, Brint E. IL-36 cytokines in inflammatory and malignant diseases: not the new kid on the block anymore. Cell Mol Life Sci. 2021;78(17-18):6215-27. 10.1007/s00018-021-03909-4 PMC8429149 34365521

[663] Dummer R, Biette K, Gusenleitner D, Ramesh R, Lebbe C, Atkinson V (2020). Effect of first-line spartalizumab + dabrafenib + trametinib on immunosuppressive features detected in peripheral blood and clinical outcome in patients (pts) with advanced BRAF V600–mutant melanoma. J Clin Oncol.

[664] Bauer T, Patel M, Jimeno A, Wang D, McDermott J, Zacharek S (2019). Abstract CT210: A Phase I, open-label, multicenter, dose escalation study of mRNA-2752, a lipid nanoparticle encapsulating mRNAs encoding human OX40L, IL-23, and IL-36γ, for intratumoral injection alone and in combination with immune checkpoint blockade. Cancer Res..

[665] Alexander JL, Moran GW, Gaya DR, Raine T, Hart A, Kennedy NA, et al. SARS-CoV-2 vaccination for patients with inflammatory bowel disease: a British Society of Gastroenterology Inflammatory Bowel Disease section and IBD Clinical Research Group position statement. Lancet Gastroenterol. Hepatol. 2021;6(3):218-24. 10.1016/S2468-1253(21)00024-8 PMC7834976 33508241

[666] Stuart ASV, Shaw RH, Liu X, Greenland M, Aley PK, Andrews NJ (2022). Immunogenicity, safety, and reactogenicity of heterologous COVID-19 primary vaccination incorporating mRNA, viral-vector, and protein-adjuvant vaccines in the UK (Com-COV2): a single-blind, randomised, phase 2, non-inferiority trial. Lancet.

[667] Longo V, Brunetti O, Azzariti A, Galetta D, Nardulli P, Leonetti F, et al. Strategies to improve cancer immune checkpoint inhibitors efficacy, other than abscopal effect: A systematic review. Cancers (Basel). 2019;11(4):539. 10.3390/cancers11040539 PMC6521062 30991686

[668] Bianchini F, Portioli E, Ferlenghi F, Vacondio F, Andreucci E, Biagioni A (2019). Cell-targeted c(AmpRGD)-sunitinib molecular conjugates impair tumor growth of melanoma. Cancer Lett.

[669] Jou J, Harrington KJ, Zocca MB, Ehrnrooth E, Cohen EEW. The changing landscape of therapeutic cancer vaccines-novel platforms and neoantigen identification. Clin. Cancer Res. 2021;27(3):689-703. 10.1158/1078-0432.CCR-20-0245 33122346

[670] Zhao Y, Baldin A V., Isayev O, Werner J, Zamyatnin AA, Bazhin A V. Cancer vaccines: Antigen selection strategy. Vaccines. 2021;9(2):85. 10.3390/vaccines9020085 PMC7911511 33503926

[671] Vogel CL, Cobleigh MA, Tripathy D, Gutheil JC, Harris LN, Fehrenbacher L (2002). Efficacy and safety of trastuzumab as a single agent in first-line treatment of HER2-overexpressing metastatic breast cancer. J Clin Oncol.

[672] Liu W, Liu Y, Zhu J, Wright E, Ding I, Rodgers GP (2008). Reduced hGC-1 protein expression is associated with malignant progression of colon carcinoma. Clin Cancer Res.

[673] Reck M, Kaiser R, Mellemgaard A, Douillard JY, Orlov S, Krzakowski M (2014). Docetaxel plus nintedanib versus docetaxel plus placebo in patients with previously treated non-small-cell lung cancer (LUME-Lung 1): A phase 3, double-blind, randomised controlled trial. Lancet Oncol.

[674] Nguyen LT, Saibil SD, Sotov V, Le MX, Khoja L, Ghazarian D (2019). Phase II clinical trial of adoptive cell therapy for patients with metastatic melanoma with autologous tumor-infiltrating lymphocytes and low-dose interleukin-2. Cancer Immunol Immunother.

[675] Gu Z, He Y, Zhang Y, Chen M, Song K, Huang Y, et al. Postprandial increase in serum CA125 as a surrogate biomarker for early diagnosis of ovarian cancer. J Transl Med. 2018;16:1-3. 10.1186/s12967-018-1489-4 PMC5930842 29716620

[676] Kunk PR, Bauer TW, Slingluff CL, Rahma OE. From bench to bedside a comprehensive review of pancreatic cancer immunotherapy. J. Immunother. Cancer. 2016;4:1-2. 10.1186/s40425-016-0119-z PMC4791889 26981244

[677] Bruno SM, Falagario UG, d’Altilia N, Recchia M, Mancini V, Selvaggio O, et al. PSA Density Help to Identify Patients With Elevated PSA Due to Prostate Cancer Rather Than Intraprostatic Inflammation: A Prospective Single Center Study. Front Oncol. 2021;11:693684. 10.3389/fonc.2021.693684 PMC8173030 34094990

[678] Bui MHT, Seligson D, Han KR, Pantuck AJ, Dorey FJ, Huang Y (2003). Carbonic anhydrase IX is an independent predictor of survival in advanced renal clear cell carcinoma: Implications for prognosis and therapy. Clin Cancer Res..

[679] Szender JB, Papanicolau-Sengos A, Eng KH, Miliotto AJ, Lugade AA, Gnjatic S (2017). NY-ESO-1 expression predicts an aggressive phenotype of ovarian cancer. Gynecol Oncol.

[680] de Vries TJ, Smeets M, de Graaf R, Hou‐Jensen K, Bröcker EB, Renard N (2001). Expression of gp100, MART‐1, tyrosinase, and S100 in paraffin‐embedded primary melanomas and locoregional, lymph node, and visceral metastases: implications for diagnosis and immunotherapy. A study conducted by the EORTC Melanoma Cooperative Group. J Pathol.

[681] Murad JP, Tilakawardane D, Park AK, Lopez LS, Young CA, Gibson J, et al. Pre-conditioning modifies the TME to enhance solid tumor CAR T cell efficacy and endogenous protective immunity. Mol Ther. 2021;29(7):2335-49. 10.1016/j.ymthe.2021.02.024 PMC8261088 33647456

[682] Han M, Choong TL, Hong WZ, Chao S, Zheng R, Kok TY (2008). Novel blood-based, five-gene biomarker set for the detection of colorectal cancer. Clin Cancer Res.

[683] Horeweg N, van Rosmalen J, Heuvelmans MA, van der Aalst CM, Vliegenthart R, Scholten ET (2014). Lung cancer probability in patients with CT-detected pulmonary nodules: A prespecified analysis of data from the NELSON trial of low-dose CT screening. Lancet Oncol.

[684] Abu-Sbeih H, Tang T, Lu Y, Thirumurthi S, Altan M, Jazaeri AA, et al. Clinical characteristics and outcomes of immune checkpoint inhibitor-induced pancreatic injury. J Immunother Cancer. 2019;7(1):1-2. 10.1186/s40425-019-0502-7 PMC6364483 30728076

[685] Black PC, Brown GA, Dinney CPN. The impact of variant histology on the outcome of bladder cancer treated with curative intent. Urol. Oncol. Semin. Orig. Investig. 2009;27:3–7. 10.1016/j.urolonc.2007.07.010 18367107

[686] Fabi A, Felici A, Metro G, Mirri A, Bria E, Telera S, et al. Brain metastases from solid tumors: Disease outcome according to type of treatment and therapeutic resources of the treating center. J Exp Clin Cancer Res. 2011;30(1):1-7. 10.1186/1756-9966-30-10 PMC3033846 21244695

[687] Masuda H, Baggerly KA, Wang Y, Zhang Y, Gonzalez-Angulo AM, Meric-Bernstam F (2013). Differential response to neoadjuvant chemotherapy among 7 triple-negative breast cancer molecular subtypes. Clin Cancer Res.

[688] Swisher SK, Vila J, Tucker SL, Bedrosian I, Shaitelman SF, Litton JK (2016). Locoregional Control According to Breast Cancer Subtype and Response to Neoadjuvant Chemotherapy in Breast Cancer Patients Undergoing Breast-conserving Therapy. Ann Surg Oncol.

[689] Patard JJ, Pignot G, Escudier B, Eisen T, Bex A, Sternberg C, ICUD-EAU international consultation on kidney cancer,  (2010). Treatment of metastatic disease. Eur Urol.

[690] Tao L, Huang G, Song H, Chen Y, Chen L. Cancer associated fibroblasts: An essential role in the tumor microenvironment (review). Oncol Lett. 2017;14(3):2611-20. 10.3892/ol.2017.6497 PMC5588104 28927027

[691] Herbst RS, Gandara DR, Hirsch FR, Redman MW, LeBlanc M, Mack PC (2015). Lung Master Protocol (Lung-MAP) - A biomarker-driven protocol for accelerating development of therapies for squamous cell lung cancer: SWOG S1400. Clin Cancer Res.

[692] Martinez A, Ngo C, Leblanc E, Gouy S, Luyckx M, Darai E (2016). Surgical Complexity Impact on Survival After Complete Cytoreductive Surgery for Advanced Ovarian Cancer. Ann Surg Oncol.

[693] Wittel UA, Lubgan D, Ghadimi M, Belyaev O, Uhl W, Bechstein WO, et al. Consensus in determining the resectability of locally progressed pancreatic ductal adenocarcinoma-results of the Conko-007 multicenter trial. BMC Cancer. 2019;19(1):1-9. 10.1186/s12885-019-6148-5 PMC6805375 31640628

[694] Karzai F, VanderWeele D, Madan RA, Owens H, Cordes LM, Hankin A, et al. Activity of durvalumab plus olaparib in metastatic castration-resistant prostate cancer in men with and without DNA damage repair mutations. J Immunother Cancer. 2018;6:1-2. 10.1186/s40425-018-0463-2 PMC6280368 30514390

[695] Sankar S, Theisen ER, Bearss J, Mulvihill T, Hoffman LM, Sorna V (2014). Reversible LSD1 inhibition interferes with global EWS/ETS transcriptional activity and impedes Ewing sarcoma tumor growth. Clin Cancer Res.

[696] Ashraf N, Hoffe S, Kim R (2013). Adjuvant Treatment for Gastric Cancer: Chemotherapy Versus Radiation. Oncologist.

[697] Schlumberger M, Jarzab B, Cabanillas ME, Robinson B, Pacini F, Ball DW (2016). A phase II trial of the multitargeted tyrosine kinase inhibitor lenvatinib (E7080) in advanced medullary thyroid cancer. Clin Cancer Res.

[698] Cantrell LA, Blank S V., Duska LR. Uterine carcinosarcoma: A review of the literature. Gynecol Oncol. 2015;137(3):581-8. 10.1016/j.ygyno.2015.03.041 25805398

[699] Li B, Xu WW, Lam AKY, Wang Y, Hu HF, Guan XY (2017). Significance of PI3K/AKT signaling pathway in metastasis of esophageal squamous cell carcinoma and its potential as a target for anti-metastasis therapy. Oncotarget.

[700] Ferris RL. Immunology and immunotherapy of head and neck cancer. J Clin Oncol. 2015;33(29):3293-304. 10.1200/JCO.2015.61.1509 PMC4586169 26351330

[701] Jiménez-Morales S, Aranda-Uribe IS, Pérez-Amado CJ, Ramírez-Bello J, Hidalgo-Miranda A. Mechanisms of Immunosuppressive Tumor Evasion: Focus on Acute Lymphoblastic Leukemia. Front Immunol. 2021;4787. 10.3389/fimmu.2021.737340 PMC8636671 34867958

[702] Solal-Céligny P, Roy P, Colombat P, White J, Armitage JO, Arranz-Saez R (2004). Follicular lymphoma international prognostic index. Blood.

[703] Nicolini F, Bocchini M, Bronte G, Delmonte A, Guidoboni M, Crinò L, et al. Malignant Pleural Mesothelioma: State-of-the-Art on Current Therapies and Promises for the Future. Front Oncol. 2020;9:1519. 10.3389/fonc.2019.01519 PMC6992646 32039010

[704] Fonseca R, Debes-Marun CS, Picken EB, Dewald GW, Bryant SC, Winkler JM (2003). The recurrent IgH translocations are highly associated with nonhyperdiploid variant multiple myeloma. Blood.

[705] Park JR, Bagatell R, Cohn SL, Pearson AD, Villablanca JG, Berthold F (2017). Revisions to the international neuroblastoma response criteria: A consensus statement from the National Cancer Institute clinical trials planning meeting. J Clin Oncol.

[706] Hiraoka N, Onozato K, Kosuge T, Hirohashi S (2006). Prevalence of FOXP3+ regulatory T cells increases during the progression of pancreatic ductal adenocarcinoma and its premalignant lesions. Clin Cancer Res.

[707] Chen PC, Tai HC, Lin TH, Wang SW, Lin CY, Chao CC (2017). CCN3 promotes epithelial-mesenchymal transition in prostate cancer via FAK/Akt/HIF-1α-induced twist expression. Oncotarget.

[708] Yu DS, Ma CP, Chang SY (2000). Establishment and characterization of renal cell carcinoma cell lines with multidrug resistance. Urol Res.

[709] Garg V, Zhang W, Gidwani P, Kim M, Kolb EA (2007). Preclinical analysis of tasidotin HCl in Ewing’s sarcoma, rhabdomyosarcoma, synovial sarcoma, and osteosarcoma. Clin Cancer Res.

[710] Apicella M, Corso S, Giordano S. Targeted therapies for gastric cancer: Failures and hopes from clinical trials. Oncotarget. 2017;8(34):57654. 10.18632/oncotarget.14825 PMC5593674 28915702

[711] Gure AO, Chua R, Williamson B, Gonen M, Ferrera CA, Gnjatic S (2005). Cancer-testis genes are coordinately expressed and are markers of poor outcome in non-small cell lung cancer. Clin Cancer Res.

[712] Appelbaum FR, Gundacker H, Head DR, Slovak ML, Willman CL, Godwin JE (2006). Age and acute myeloid leukemia. Blood.

[713] Wang JP, Leng JY, Zhang RK, Zhang L, Zhang B, Jiang WY (2018). Functional analysis of gene expression profiling-based prediction in bladder cancer. Oncol Lett.

[714] Skrap M, Marin D, Ius T, Fabbro F, Tomasino B (2016). Brain mapping: A novel intraoperative neuropsychological approach. J Neurosurg.

[715] Nathan MR, Schmid P (2018). The emerging world of breast cancer immunotherapy. Breast.

[716] Qaderi SM, Wijffels NAT, Bremers AJA, De Wilt JHW. Major differences in follow-up practice of patients with colorectal cancer; Results of a national survey in the Netherlands. BMC Cancer. 2020;20(1):1-8. 10.1186/s12885-019-6509-0 PMC6945647 31906899

[717] Wilson BE, Routy B, Nagrial A, Chin VT (2020). The effect of antibiotics on clinical outcomes in immune-checkpoint blockade: a systematic review and meta-analysis of observational studies. Cancer Immunol Immunother.

[718] Bakalian S, Marshall JC, Logan P, Faingold D, Maloney S, Di Cesare S, et al. Molecular pathways mediating liver metastasis in patients with uveal melanoma. Clin. Cancer Res. 2008;14(4):951-6. 10.1158/1078-0432.CCR-06-2630 18281525

[719] Babatz J, Röllig C, Löbel B, Folprecht G, Haack M, Günther H (2006). Induction of cellular immune responses against carcinoembryonic antigen in patients with metastatic tumors after vaccination with altered peptide ligand-loaded dendritic cells. Cancer Immunol Immunother.

[720] Imai Y, Hasegawa K, Matsushita H, Fujieda N, Sato S, Miyagi E (2018). Expression of multiple immune checkpoint molecules on t cells in malignant ascites from epithelial ovarian carcinoma. Oncol Lett.

[721] McCormick KA, Coveler AL, Rossi GR, Vahanian NN, Link C, Chiorean EG (2016). Pancreatic cancer: Update on immunotherapies and algenpantucel-L. Hum Vaccines Immunother.

[722] Italiano A, Dinart D, Soubeyran I, Bellera C, Espérou H, Delmas C, et al. Molecular profiling of advanced soft-tissue sarcomas: the MULTISARC randomized trial. BMC Cancer. 2021;21(1):1-0. 10.1186/s12885-021-08878-2 PMC8570026 34740331

[723] Ilan-Ber T, Ilan Y (2019). The role of microtubules in the immune system and as potential targets for gut-based immunotherapy. Mol Immunol.

[724] Stenner F, Liewen H, Zweifel M, Weber A, Tchinda J, Bode B (2008). Targeted therapeutic approach for an anaplastic thyroid cancer in vitro and in vivo. Cancer Sci.

[725] Jabulowsky RA, Loquai C, Mitzel-Rink H, Utikal J, Gebhardt C, Hassel JC (2018). Abstract CT156: A first-in-human phase I/II clinical trial assessing novel mRNA-lipoplex nanoparticles encoding shared tumor antigens for immunotherapy of malignant melanoma. Cancer Res..

[726] Sun M, Yuan Y, Lu F, Di Pasqua AJ (2021). Physicochemical factors that influence the biocompatibility of cationic liposomes and their ability to deliver DNA to the nuclei of ovarian cancer SK-OV-3 cells. Materials (Basel).

[727] Froning KJ, Sereno A, Huang F, Demarest SJ. Generalizable design parameters for soluble T cell receptor-based T cell engagers. J Immunother Cancer. 2022;10(3). 10.1136/jitc-2021-004281 PMC8905924 35260435

[728] Gebre MS, Rauch S, Roth N, Yu J, Chandrashekar A, Mercado NB (2022). Optimization of non-coding regions for a non-modified mRNA COVID-19 vaccine. Nature.

[729] Dolgin E. CureVac COVID vaccine let-down spotlights mRNA design challenges. Nature. 2021;594:483. 10.1038/d41586-021-01661-0 34145413

[730] Rahman M, Ghiaseddin A, Yegorov O, Yang C, Dechkovskaia A, Tran D (2019). ATIM-15 ATIM-15. sustained complete radiographic response and prolonged systemic immune activation in a patient with mgmt unmethylated midline glioblastoma receiving CMV pp65-LAMP RNA-pulsed dendritic cell vaccines. Neuro Oncol..

[731] Ballegaard V, Pedersen KK, Brændstrup P, Kirkby N, Stryhn A, Ryder LP, et al. Cytomegalovirus-specific CD8+ T-cell responses are associated with arterial blood pressure in people living with HIV. PLoS One. 2020;15(1):e0226182. 10.1371/journal.pone.0226182 PMC6957152 31929537

[732] Wong YY, Ng SP, Ng MH, Si SH, Yao SZ, Fung YS (2002). Immunosensor for the differentiation and detection of Salmonella species based on a quartz crystal microbalance. Biosens Bioelectron.

[733] Wachowska M, Muchowicz A, Golab J. Targeting epigenetic processes in photodynamic therapy-induced anticancer immunity. Front Oncol. 2015;5:176. 10.3389/fonc.2015.00176 PMC4519687 26284197

[734] Martínez-Cortés F, Servín-Blanco R, Domínguez-Romero AN, Munguía ME, Guzman Valle J, Odales J (2021). Generation of cancer vaccine immunogens derived from Oncofetal antigen (OFA/iLRP) using variable epitope libraries tested in an aggressive breast cancer model. Mol Immunol.

[735] Leuchte K, Staib E, Thelen M, Gödel P, Lechner A, Zentis P (2021). Microwave ablation enhances tumor-specific immune response in patients with hepatocellular carcinoma. Cancer Immunol Immunother.

[736] Buonaguro L, Tagliamonte M. Selecting target antigens for cancer vaccine development. Vaccines. 2020;8(4):615. 10.3390/vaccines8040615 PMC7711972 33080888

[737] Stergiou N, Gaidzik N, Heimes AS, Dietzen S, Besenius P, Jäkel J (2019). Reduced breast tumor growth after immunization with a tumor-restricted MUC1 glycopeptide conjugated to tetanus toxoid. Cancer Immunol Res.

[738] Yamamoto TN, Kishton RJ, Restifo NP. Developing neoantigen-targeted T cell–based treatments for solid tumors. Nat Med. 2019;25(10):1488-99. 10.1038/s41591-019-0596-y 31591590

[739] Garcia-Garijo A, Fajardo CA, Gros A. Determinants for neoantigen identification. Front Immunol. 2019;10:1392. 10.3389/fimmu.2019.01392 PMC6601353 31293573

[740] Gerami SM, Somi MH, Vahedi L, Farassati F, Dolatkhah R (2020). The APC gene rs41115 polymorphism is associated with survival in Iranian colorectal cancer patients. Biomed Res Ther..

[741] Pourseif MM, Parvizpour S, Jafari B, Dehghani J, Naghili B, Omidi Y (2021). A domain-based vaccine construct against SARS-CoV-2, the causative agent of COVID-19 pandemic: Development of self-amplifying mRNA and peptide vaccines. BioImpacts.

[742] Zhan X, Wang B, Wang Y, Chen L, Peng X, Li J (2020). Phase I trial of personalized mRNA vaccine encoding neoantigen in patients with advanced digestive system neoplasms. J Clin Oncol.

[743] Valdivia L, García-Hevia L, Bañobre-López M, Gallo J, Valiente R, Fanarraga ML (2021). Solid lipid particles for lung metastasis treatment. Pharmaceutics.

[744] Shibata H, Xu N, Saito S, Zhou L, Ozgenc I, Webb J, et al. Integrating CD4+ T cell help for therapeutic cancer vaccination in a preclinical head and neck cancer model. Oncoimmunology. 2021;10(1):1958589. 10.1080/2162402X.2021.1958589 PMC8366550 34408919

[745] Rajan TS, Gugliandolo A, Bramanti P, Mazzon E. In vitro-transcribed mrna chimeric antigen receptor T cell (IVT mrna car T) therapy in hematologic and solid tumor management: A preclinical update. Int J Mol Sci. 2020;21(18):6514. 10.3390/ijms21186514 PMC7556036 32899932

[746] Sabbagh A, Beccaria K, Ling X, Marisetty A, Ott M, Kong L-Y (2021). Abstract 1489: Deposition of genetically engineered T cell attracting antigen presenting cells in the glioma microenvironment with low intensity pulsed ultrasound-based blood-brain barrier opening triggers therapeutic responses in preclinical glioma model. Cancer Res.

[747] Tabernero J, Bendell J, Corcoran R, Kopetz S, Lee J, Davis M (2021). P-71 KRYSTAL-10: A randomized phase 3 study of adagrasib (MRTX849) in combination with cetuximab vs chemotherapy in patients with previously treated advanced colorectal cancer with KRASG12C mutation. Ann Oncol.

[748] Marabelle A, Cassier P, Delord J-P, Jungles C, Champiat S, Vinceneux A (2019). 162TiP A phase I study evaluating BI 765063, a first in class selective myeloid SIRPa inhibitor, as standalone and in combination with BI 754091, a programmed death-1 (PD-1) inhibitor, in patients with advanced solid tumours. Ann Oncol..

